# Towards a membrane proteome in *Drosophila*: a method for the isolation of plasma membrane

**DOI:** 10.1186/1471-2164-11-302

**Published:** 2010-05-12

**Authors:** Mansi R Khanna, Bruce A Stanley, Graham H Thomas

**Affiliations:** 1 Departments of Biology and of Biochemistry and Molecular Biology, The Pennsylvania State University, University Park, PA 16802 USA; 2 Section of Research Resources, Pennsylvania State University College of Medicine, Hershey, PA 17033, USA

## Abstract

**Background:**

The plasma membrane (PM) is a compartment of significant interest because cell surface proteins influence the way in which a cell interacts with its neighbours and its extracellular environment. However, PM is hard to isolate because of its low abundance. Aqueous two-phase affinity purification (2PAP), based on PEG/Dextran two-phase fractionation and lectin affinity for PM-derived microsomes, is an emerging method for the isolation of high purity plasma membranes from several vertebrate sources. In contrast, PM isolation techniques in important invertebrate genetic model systems, such as *Drosophila melanogaster*, have relied upon enrichment by density gradient centrifugation. To facilitate genetic investigation of activities contributing to the content of the PM sub-proteome, we sought to adapt 2PAP to this invertebrate model to provide a robust PM isolation technique for *Drosophila*.

**Results:**

We show that 2PAP alone does not completely remove contaminating endoplasmic reticulum and mitochondrial membrane. However, a novel combination of density gradient centrifugation plus 2PAP results in a robust PM preparation. To demonstrate the utility of this technique we isolated PM from fly heads and successfully identified 432 proteins using MudPIT, of which 37% are integral membrane proteins from all compartments. Of the 432 proteins, 22% have been previously assigned to the PM compartment, and a further 34% are currently unassigned to any compartment and represent candidates for assignment to the PM. The remainder have previous assignments to other compartments.

**Conclusion:**

A combination of density gradient centrifugation and 2PAP results in a robust, high purity PM preparation from *Drosophila*, something neither technique can achieve on its own. This novel preparation should lay the groundwork for the proteomic investigation of the PM in different genetic backgrounds in *Drosophila*. Our results also identify two key steps in this procedure: The optimization of membrane partitioning in the PEG/Dextran mixture, and careful choice of the correct lectin for the affinity purification step in light of variations in bulk membrane lipid composition and glycosylation patterns respectively. This points the way for further adaptations into other systems.

## Background

The plasma membrane (PM) and its associated proteins play an important role in determining how a cell interacts with its neighbours as well as how it responds to components of, and conditions in its extracellular environment. As a reflection of this, more than 50% of the current drug targets lie at the cell surface [1]. The amount of a protein at the cell surface is determined by its rate of delivery, internalization, recycling and degradation. All these parameters are subject to change during normal physiological adjustments, development, varying environmental influences and pathological conditions [2]. Obviously, to monitor such changes *via *total protein level, when the surface pool is the active population, would mask key regulatory changes that arise from movement to and from other sub-cellular compartments. Thus, it is essential to develop techniques that permit the effective study of the surface pool specifically.

The challenge for isolation of the PM is its low abundance - 10% or less of the cellular membrane, depending on the tissue type - that is easily overwhelmed by high abundance compartments such as the endoplasmic reticulum (ER). Various techniques to isolate plasma membranes exist, and each has its strengths and weaknesses. Density gradient centrifugation separates biomolecules and organelles on the basis of their buoyant densities. Although this results in fractionation, similarities in membrane density inevitably lead to an overlap between cellular compartments (reviewed in [3]). Immunoaffinity purification using antibodies against cell surface proteins has been used to isolate plasma membranes from rat liver [4] and mouse livers with relatively low contamination from other compartments [5]. However, this method depends on the availability of a good antibody to a single protein and is thus likely to be tissue specific and biased towards a specific PM domain in polarized cells. Global surface labeling with biotin and then isolation of biotinylated surface proteins with the use of streptavidin has been used before (reviewed in [6]). However, this is not a practical technique for plasma membrane isolation from a whole organ or organism. Recently, aqueous two-phase affinity partitioning (2PAP) has emerged as a useful technique to isolate plasma membranes from several sources [7-11]. In this method, plasma membrane is first partitioned into the polyethylene glycol (PEG) layer of a two-phase PEG/Dex system, then selectively pulled into the dextran phase by the use of the lectin wheat germ agglutinin (WGA) coupled to the dextran to select for membrane containing glycosylated proteins.

It is a desirable goal to combine 2PAP with a genetic approach to facilitate regulatory studies of global cell surface protein population. However, the currently established method for this depends upon the use of the lectin wheat germ agglutinin (WGA), which has specificity for N-acetylhexosamines or sialic acid in certain linkages [12]. This limits the utility of this phase separation for the isolation of plasma membrane from invertebrate genetic models, such as *Drosophila*, where glycoyslation patterns are much simpler, and have a high mannose content [13,14]. Simply changing lectin to Conconavalin A (ConA), which has a high affinity for mannose, creates a new problem as this will now efficiently isolate endoplasmic reticulum and Golgi resident proteins because oligomannosidic proteins are extensively present in the ER. Here, we present a novel combination of density gradient centrifugation and affinity purification using 2PAP to isolate plasma membranes from *Drosophila melanogaster*. We show that 2PAP alone, is not sufficient to eliminate contaminating membranes, and that prior enrichment of plasma membranes is required. The addition of an initial gradient fractionation permits the efficient removal of the ER and Golgi membranes. Using this new protocol we identified 432 *Drosophila *head proteins by MudPIT analysis, 22-56% of which are likely to be PM proteins. Our results provide an initial PM proteome for this largely neuronal tissue source.

## Methods

### Extract Preparation

The wild-type *Drosophila *line Oregon R was used for all extractions. *Embryos*: 1.5-3.0 g of embryos (*Oregon R*; 0-15 hours old) were dechorionated in 50% bleach, thoroughly rinsed, and carefully homogenized in 10 volumes of homogenization buffer I (HB-I; 0.22 M sucrose, 0.12 M mannitol, 1 mM EDTA and 10 mM tricine, pH 7.2) [15] containing 1× protease inhibitor cocktail (10 μM benzamidine, 1 μg/ml phenanthroline and 10 μg/ml each of aprotinin, leupeptin and pepstatin A) [16] by 15 strokes of Pestle A in a Kontes homogenizer on ice. *Heads*: Heads were isolated from 15-20 g of 4-5 day old flies using a modification of the standard freezing protocol [17]: Flies were frozen at -80°C for a minimum of one hour in a 200 ml centrifuge bottle, shaking briskly to decapitate and passing through a stack of frozen metal sieves of decreasing pore size (850 μm, 600 μm and 355 μm) and homogenized with 5 ml of HB-I by 3 strokes in a Kontes homogenizer and motor driven ground glass pestle. This was followed by 10 strokes of Pestle A in a Kontes homogenizer on ice in a total volume of 20 ml. All subsequent steps were carried out at 4°C. Use of liquid N_2 _for head isolation [17] is to be avoided as this leads to a catastrophic mixing of membrane compartments. The initial homogenate was centrifuged: at 3,000 × *g *for 10 min at 4°C to pellet nuclei; at 10,000 × *g *for 30 min at 4°C to pellet mitochondria; and at 100,000 × *g *for 1 h to obtain a microsome pellet.

To proceed directly with 2PAP, the pellet was washed once by resuspending and repelleting in HB-II (0.25 M sucrose, 15 mM Tris, pH 7.4; [11]) containing 0.1× protease inhibitor cocktail. The pellet thus obtained was then resuspended in 400 μl of HB-II and used for affinity partitioning. To proceed with density gradient centrifugation, the microsome pellet was resuspended in 5 ml HB-I.

Protein estimations were performed using the Advanced Protein Assay Reagent (Cytoskeleton Inc., Denver, CO) according to the manufacturer's instructions.

### Preparation of ConA-Dextran

ConA was coupled to tresyl-dextran as previously described for coupling of WGA to dextran [8]. All solvents used to activate dextran were dried using silica gel (0.02 g/ml). All glassware to be used for activation was dried overnight at 65°C. The tresyl-dextran should be used as soon as possible after preparation for coupling to avoid loss of capacity.

### Aqueous two-phase affinity partitioning

**Figure 1:**
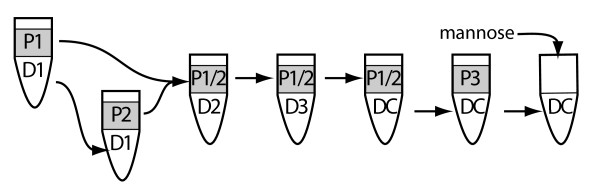
**Schematic representation of the two-phase affinity purification technique**. See Methods section for procedure. P1-3 - PEG phases; D1-3 - Dextran phases; DC - Conconavalin A-coupled dextran; mannose - sugar used for elution from ConA.

Aqueous two-phase affinity partitioning was done essentially as previously described [11]; Figure 1). In brief, 100 μl of the microsomes in HB-II (~3 mg protein) were added to 0.9 g of a two-phase system (6.3% or 5.7% (w/w) dextran plus 6.3% or 5.7% (w/w) PEG, respectively, in 15 mM Tris/H_2_SO_4_, pH 7.8) to complete a 1-g system. This was mixed by 20 inversions, vortexing for 10 sec, followed by another 20 inversions. Phase separation was assisted by centrifugation at 150 × *g *for 5 min, to give a top phase (P1) and a bottom phase (D1). The bottom phase (D1) was re-extracted with an equal volume of fresh top phase (P2) from a pre-equilibrated 1-g two-phase system and combined with (P1). (P1 + P2) were combined and layered onto a fresh bottom phase (D2) that had been pre-equilibrated against PEG. After extraction, the resulting top layer was extracted once again with a fresh bottom phase (D3) as before. The top phase was then subjected to affinity purification with the bottom phase of a 2-g two-phase system (6.3% or 5.7% (w/w) dextran, 6.3% or 5.7% (w/w) PEG, 200 μg of ConA as ConA-dextran, 2 mM LiSO_4_, 15 mM Tris borate, pH 7.8). The top phase (P1+P2) was kept aside and the bottom phase was re-extracted with the same volume of a fresh top phase (P3) from a new 2-g two-phase system (6.3% or 5.7% (w/w) dextran, 6.3% or 5.7% (w/w) PEG, 2 mM LiSO_4_, 15 mM Tris borate, pH 7.8). The resulting bottom phase (ConA) was diluted with 10 volumes of elution solution (0.1 M mannose, 0.25 M sucrose). All other phases (D1, D2, D3, P1+P2 and P3) were diluted 10-fold in HB-II. All samples were centrifuged at 100,000 × *g *for 1.5 h at 4°C to pellet membranes. The pellets thus obtained were resuspended in 200 μl of HB-II prior to further analysis.

### Density gradient centrifugation

Density gradient centrifugation was done essentially as previously described [16]. Briefly, the initial 100,000 × *g *microsome pellet (see above) was resuspended and mixed with OptiPrep (Accurate Chemical and Scientific Corp., Westbury, NY) such that a 10-30% gradient was created with a total protein content of 12-15 mg per gradient. Centrifugation was at 286,675 × *g *for 3.6 h. 0.25 ml fractions were collected from the bottom of the tube for further analysis.

### Combined density gradient centrifugation and aqueous two-phase separation

The uppermost fractions (15-20) from the density gradient contains the bulk of the plasma membrane and were pooled, diluted in 5 volumes HB-II and pelleted by centrifugation at 100,000 × *g *for 1.5 h at 4°C. The pellet was resuspended in HB-II (= 'Pool') and subjected to 2PAP (see above).

### Antibodies

The following primary antibodies were used for immunoblotting: mouse anti-α-Spectrin (1:75,000; ascites #N3 from Dr. D. Branton, Harvard University, Cambridge, MA); rat anti-BiP (1:20,000; Babraham Institute, Cambridge, UK); mouse anti-ATP synthase (1:100,000; MitoSciences, Eugene, OR), mouse anti-Nervana (1:10,000; Developmental Studies Hybridoma Bank, Iowa City, IA), rabbit anti-HRP (1:2000; a gift from Dr. Richard Ordway), rabbit anti-Lava lamp (1:50,000 gift from Dr. John Sisson). HRP-conjugated anti-mouse, anti-rat and anti-rabbit secondary antibodies (1:2500) were all purchased from Jackson Immunoresearch (West Grove, PA).

### SDS-PAGE and Immunoblotting

Proteins were separated on SDS-polyacrylamide gels according to standard (12% for Nervana, ATP synthase, BiP and HRP; [18]) or high molecular weight (7.5% for α-Spectrin, ATP synthase and BiP and 6% for Lava lamp; [19]) protocols. For direct visualization of proteins, gels were stained with Colloidal Coomassie Blue (Invitrogen, Carlsbad, CA). For immunoblotting, proteins were transferred to Hybond ECL nitrocellulose membrane (Amersham Biosciences, Piscataway, NJ) and probed using standard protocols with final detection by 'normal sensitivity' chemiluminescence (~10 pg detection limit; ECL, Amersham Biosciences, Piscataway, NJ) or 'high sensitivity' chemiluminescence (low fg detection limit; SuperSignal West Femto, Pierce Biotechnology, Rockford, IL) using Pierce CL-Xposure Film (Thermo Scientific, Rockford, IL).

### Enzyme Assays

The following marker enzyme assays were used to detect specific subcellular compartments: Alkaline phosphatase for the plasma membrane [20], Succinate dehydrogenase for mitochondria [21] and Cytochrome c reductase (NADPH) for the endoplasmic reticulum (Cytochrome c Reductase (NADPH) Assay Kit, Sigma-Aldrich, St.Louis, MO).

### Preparation of samples for Mass Spectrometry

The ConA pellet was subjected to in-solution proteolysis essentially as previously described [22]. Eluted membrane pellets were resuspended and reduced (2.5 mM DTT, 50 mM ammonium bicarbonate, pH 8) at 50°C for 30 min with sonication. This was followed by alkylation (10 mM iodoacetamide, 50 mM ammonium bicarbonate, pH 8) in the dark for 30 min at 37°C and quenching with 11 mM DTT at 37°C for 30 min. The reduced and alkylated proteins were then digested with 1 μg of Promega Gold Trypsin (Promega Corp., Madison, WI) in 200 μl 50 mM ammonium bicarbonate and 37% acetonitrile at 48°C for 3 h, followed by 37°C for 16 h. Following digestion, the reaction was dried to remove solvents and buffers, and resuspended in 200 μl of distilled water. Drying and resuspension were done two more times and the pellet was finally resuspended in 10 μl of 0.1% trifluoroacetic acid.

### Mass Spectrometry and data interpretation

Mass spectrometry and data acquisition was done at The Mass Spectrometry Core Research Facility at Penn State, Hershey as described [22]. Peptides were separated using LC-MALDI techniques through two sequential columns: strong cation exchange (SCX) and C18 nanoflow chromatography. The samples were dried down, loaded in SCX loading buffer and SCX separations were carried out on a passivated Waters 600E HPLC system, using a 4.6 × 250 mm PolySulfoethyl Aspartamide column (PolyLC, Columbia, MD) at a flow rate of 1 ml/min. Buffers used were Buffer A (10 mM ammonium formate, pH 3.6, in 20% acetonitrile/80% water) and Buffer B (666 mM ammonium formate, pH 3.6, in 20% acetonitrile/80% water). The gradient was Buffer A at 100% (0-30 minutes following sample injection), 0% → 35% Buffer B (30-48 min), 35% → 100% Buffer B (48-49 min), 100% Buffer B (49-56 min), then at 56 min reverted to 100% A to re-equilibrate for the next injection. The first 28 ml of eluant (containing all flow-through fractions) were combined into one fraction, then 14 additional 2-ml fractions were collected. All 15 of these SCX fractions were dried down completely to reduce volume and to remove the volatile ammonium formate salts, resuspended in 15 μl of 2% (v/v) acetonitrile, 0.1% (v/v) trifluoroacetic acid and filtered before reverse phase C18 nanoflow-LC separation. For reverse phase nanoflow-LC, each SCX fraction was autoinjected onto a Chromolith CapRod column (150 × 0.1 mm; EMD Chemicals Inc., Gibbstown, NJ) using a 5 μl injector loop on a Tempo LC MALDI Spotting system (ABI-MDS/Sciex). Buffers used were Buffer C (2% acetonitrile, 0.1% trifluoroacetic acid) and Buffer D (98% acetonitrile, 0.1% trifluoroacetic acid). The elution gradient was {95% C/5% D (0-8 min)}, ^®^40% D (8.1-40 min), ^®^80% D (41-44 min), ^®^5% D (44-49 min) (initial conditions). Flow rate was 2.5 μl/min, and an equal flow of MALDI matrix solution was added post-column (7 mg/ml recrystallized CHCA (α-cyano-hydroxycinnamic acid), 2 mg/ml ammonium phosphate, 0.1% trifluoroacetic acid, 80% acetonitrile). The combined eluant was automatically spotted onto a stainless steel MALDI target plate every 6 seconds (0.6 μl per spot), for a total of 370 spots per original SCX fraction. The resulting 5500 MALDI spots were analyzed and MS and MS/MS spectra were obtained using an ABI 4800 MALDI TOF-TOF analyzer. Peptide and protein identification was performed with the Paragon "Sequence Temperature Value" algorithm [23] contained in Protein Pilot software version 2.01 (Applied Biosystems/MDS Sciex), and the ProGroup algorithm for protein inference and grouping from tandem mass spectrometry (MS/MS) spectral/peptide data. Search criteria were trypsin-cleaved peptides; iodoacetamide-modified cysteines; ID Focus = Biological Modifications; Thorough Search setting; and Detected Protein Threshold = 0.05 (10.0%). Protein Pilot automatically searches for a series of potential biological and sample preparation-induced modifications once a suitable sequence tag of 3-4 amino acids has been found within an MS/MS spectrum.

MS/MS data from 2D LC MALDI MudPIT experiments were analyzed using both Mascot and Protein Pilot software version 2.0. For both algorithms, protein identification acceptance criteria were C.I ≥ 98% (equal to a Protein Pilot Unused Score of 1.7) for proteins identified with multiple peptides, and C.I ≥ 99.9% for proteins detected from a single peptide, plus acceptable false discovery rates (FDRs). In the Protein Pilot analyses, proteins identified through MudPIT were accepted only if they met our C.I. criterion and also had an estimated FDR < 0.05. The decisions about how to arrive at the minimal protein list which accounts for all the observed spectral evidence are calculated by the ProFound algorithm also contained in the Protein Pilot software. All identified proteins had an Unused Score of 1.7 or higher, which corresponds to a confidence of 98% or higher. A second requirement was that all identified proteins have an estimated local FDR of 5% or less, based on the number of IDs at any cutoff Unused Score from a "normal" database (database searched was the NCBInr *Drosophila *Protein Sequences as of 11/26/2008, containing 89,592 protein sequences) compared to the number of IDs from a concatenated forward and decoy database plus a list of known (ConA) or common potential contaminants such as keratins, common laboratory reagents such as BSA, and trypsin autolysis peaks. The decoy database was a randomized version of the same NCBInr database, where amino acid frequencies in the database were kept the same as in the normal database. The FDR used as a cutoff for accepting Protein Pilot identified proteins was a local (sometimes called "instantaneous") FDR of 5% or lower, meaning that the protein with the lowest accepted score still had an estimated probability of less than 5% of being a false positive, based on the rate of increase in the accumulation of decoy database hits at that particular cutoff score. This local FDR was calculated using the Proteomics System Performance Evaluation Pipeline (PSPEP) tool [24]. Proteins appearing in databases under different names and accession numbers is taken care of by the ProGroup algorithm embedded in ProteinPilot software, which groups all homologous proteins with different names in the database under one Protein Family, selecting only one of these equivalent protein IDs for inclusion on the list of 432 proteins.

## Results

### Aqueous two-phase affinity partitioning I (6.3%PEG/Dextran)

Aqueous two-phase affinity partitioning (2PAP) separates membranes through their differing affinities for the polyethylene glycol and dextran phases coupled with the affinity of glycosylated surface proteins for lectins, and represents a quick and easy method to isolate plasma membrane (PM) proteins. We first attempted to isolate plasma membrane from *Drosophila melanogaster *using the 2PAP method previously used to enrich for PM from rat brains [11]. To adapt the protocol for fly tissue, we made two changes to this previously described protocol: First, we changed the lectin used from wheat germ agglutinin (WGA) to Concanavalin A (ConA). This was an essential change because the pattern of *Drosophila *protein glycosylation is simpler than that in vertebrates, containing a high proportion of mannose [13,25]. WGA has specificity for N-acetylhexosamines or sialic acid in certain linkages, and while there is some tissue reactivity with WGA [14], this lectin is primarily used as a nuclear stain in fly embryos and does not react with the PM, for example [26]. In adapting this methodology to other model systems similar consideration of species-specific glycosylation patterns should be made. Second, we used a slightly different isolation buffer to try and maintain the integrity of the mitochondria during the initial stages of the preparation [15]. The basic procedure is laid out in figure 1.

Evaluating PM purity is not straightforward, because few PM proteins are completely absent from any internal compartment due to continuous biosynthesis and turnover from the cell surface. Previous studies using 2PAP have reported plasma membrane enrichment and removal of contaminating compartments with the help of marker enzyme activities [7,10,11]. However, the PM marker Alkaline Phosphatase (ALP) also has a significant internal pool [27], and only ~5% is recovered in the PM fraction [11]. Clearly, yield and enrichment calculations become less dependable when they are based on a tiny fraction of the marker activity. Moreover, enzyme activity must survive the preparation to be fully accurate, and in our preparations the additional complication of significant levels of ConA in the final pellets (see below) leads to false estimates of total protein and therefore final specific activities. Immunoblotting is an obvious alternative but has similar problems (see discussion in [11]; results presented below). We performed both evaluations with our initial samples but eventually selected immunoblotting, and define our optimal PM fraction to have the maximum yield of a known PM marker with the simultaneous absence of the ER marker BiP.

We chose two tissue sources for our experiments: Embryos, which are easy to produce in large amounts, but are heterogeneous in tissue content, and heads, which are relatively homogeneous (~85% neuronal - brain plus optic lobe) and thus provide a tissue type that is readily isolated en masse (see Methods). Typically, heads from ~5 g of flies yielded ~7.5 mg of protein, of which ~2 mg of protein was used for a single 1-g phase separation system, that was set up as previously described [11]. ~1.5 g of embryos yielded ~15 mg of protein, of which ~3.5 mg of protein was used for a single 1-g phase separation system. To track the isolation of different membrane compartments we performed immunoblot analyses. As plasma membrane markers we eventually settled on Nervana (Nrv; the brain-specific isoform of the β subunit of the Na^+^/K^+ ^ATPase; [28]) for head extracts, and α-Spectrin in embryo extracts [16]. In addition, we used the chaperone BiP and the α subunit of ATP synthase for both tissue sources as markers for the endoplasmic reticulum (ER) and mitochondrial membrane, respectively. We also characterized our preparations from embryos using enzyme assays for Alkaline phosphatase (PM) and Succinate dehydrogenase (SDH; mitochondria) as previously suggested [11] and Cytochrome c reductase (CCR; ER), all of which have homologues in the fly. However, these results are expressed as yields rather than relative specific activities [7,11] because the protein concentration cannot be accurately estimated in our final eluate due to significant leaching of ConA from the dextran. This release arises because not all subunits of the ConA tetramers are cross-linked to the dextran and some disassembly occurs at the elution step.

**Figure 2:**
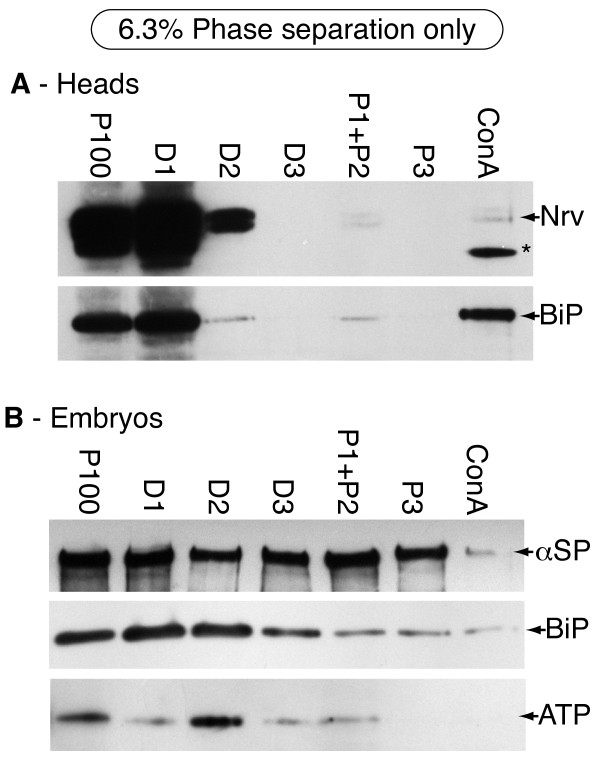
**Immunoblot analysis of samples fractionated by two phase affinity partitioning using 6.3%PEG/Dextran**. **A - **Fractionation of microsomes prepared from heads. Only small amounts of Nervana are recovered in the ConA fraction, and these contain significant amounts of the ER marker BiP. *Labeling*: P100 - input microsomes from the 100,000 × g pellet; D1-3 - Dextran fractions D1-3 in figure 1; P1+P2, P3 - PEG fractions P1-3 in figure 1; ConA - eluate released from the ConA-dextran fraction DC in figure 1. All samples represent remaining protein after each fractionation step. Asterisk - non-specific antibody binding to a large amount of ConA that coelutes from the dextran. Nrv - Nervana; BiP - ER chaperon BiP; ATP - α subunit of the mitochondrial F_1_F_0 _ATPase; αSp - α-Spectrin. *Loading: *All samples have equivalent loading, except P100, which was 1/5^th ^of the others. *Detection: *normal sensitivity chemiluminescence (see Methods). **B - **Fractionation of microsomes prepared from 0-15 hr embryos. Only small amounts of α-Spectrin are recovered in the ConA fraction. This fraction also contains readily detectable amounts of the ER marker BiP and small amounts of ATP synthase from mitochondria. Labels, loadings and detection are the same as A.

This basic 2PAP procedure removes a large amount of the ER and mitochondrial membrane through partitioning into the dextran phases (D1, D2 and D3 in Figure 2). However, the PM fraction (ConA in Figure 2) still contains detectable levels of ER and mitochondria. In preparations from both, heads and embryos, a significant amount of PM markers partition into the dextran phases perhaps representing an endomembrane pool of these proteins. The overall result is a relatively low yield in the final ConA fraction. To quantify these results the eluted ConA fraction was assessed using the marker enzyme assays, with the microsomal fraction for reference (Table 1). The results indicate a slight selectivity for the plasma membrane in the ConA phase: 0.55% of the ALP activity was recovered, compared to 0.37% for both the SDH and CCR activities (Table 1). Given the disproportionate ratio of ER to PM in a typical cell, this represents a significant level of contamination, and suggests that the 2PAP protocol alone is not sufficient to reduce contamination by these other fractions to the same extent as with vertebrate samples. This probably reflects the use of ConA, since mannose is extensively present in the ER.

**Table 1:** Relative yields of membrane compartments determined from marker enzymes.

Method	Enzyme	Microsomes	PM Pool	ConA
Affinity	ALP	100		0.55 ± 0.44
	
	SDH	100		0.37 ± 0.15
	
	CCR	100		0.37 ± 0.26

Combination	ALP	100	0.7 ± 0.14	0.4 ± 0.17
	
	SDH	100	0.8 ± 0.07	0.25 ± 0.04
	
	CCR	100	1.1 ± 0.52	0.1 ± 0.02

Gradient+2PAP (6.3%)	ALP		100	54.0 ± 23.4
	
	SDH		100	30.0 ± 5.31
	
	CCR		100	12.9 ± 1.7

Embryos were used as the starting material. Yields of Alkaline phosphatase (ALP; EC 3.1.3.1; PM marker), Succinate dehydrogenase (SDH; EC 1.3.5.1; mitochondrial marker) and Cytochrome c reductase (NADPH) (CCR; EC 1.6.2.4; ER marker). Because significant amounts of ConA elute in the final fraction, specific activities cannot be presented and these figures are raw activity yields, normalized to the activity present in the microsome pellets. Results represent mean ± 1 standard deviation of values obtained from three experiments after adjustment for differences in protein concentration in the microsomal input fraction. The addition of the pre-fractionation by density gradient centrifugation preferentially reduces the amount of contaminating ER in the ConA fraction.

### Density gradient centrifugation

**Figure 3:**
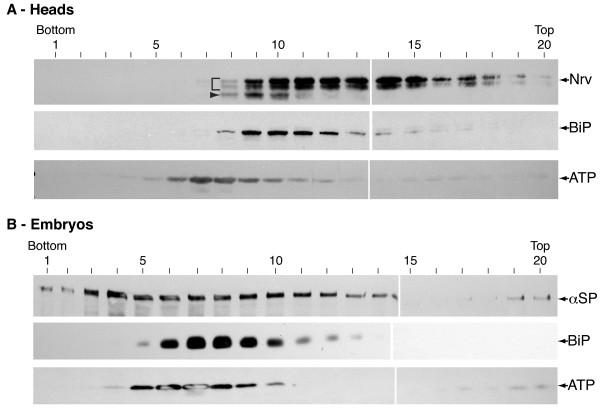
**Immunoblot analysis of samples fractionated on 10-30% Optiprep density gradients**. **A - **Fractionation of microsomes prepared from heads. BiP, marking the ER, peaks in the center of the gradient in fractions 9-11. ATP synthase shows that residual mitochondrial membrane is slightly heavier than the ER with a peak in fraction 7, but also shows a small presence in higher fractions at the top of the gradient. Nervana is seen as three prominent bands. The lowest (arrowhead) represents minimally processed protein and is restricted to the ER. The mature glycosylated forms (bracket) represent post-Golgi compartments and the plasma membrane. See [28] for a description of Nervana glycosylation patterns. **B - **Fractionation of microsomes prepared from 0-15 hr embryos. BiP, marking the ER, peaks slightly lower than the ER from head gradients in fractions 7-9. ATP synthase again peaks in fraction 7, and again has a small presence in higher fractions at the top of the gradient. α-Spectrin has a peak in the Golgi region (fractions 3-4), extends through the ER and shows a distinct peak at the top of the gradient. *Labeling: *1-20 - fraction number from bottom to top of tube; all other labels are as in figure 2. *Loading: *An equal volume of all fractions were loaded, except the peak protein fractions (4-6) where 50 μg of protein was loaded to prevent overloading.

The persistence of contaminating sub cellular compartments using 2PAP alone prompted us to first try to enrich for plasma membrane using density gradient centrifugation on the P100 microsomal fraction [16]. We began by characterizing the efficacy of Optiprep density centrifugation in isolation. Immunoblot analysis of the gradient fractions shows that different sub cellular compartments lie in different parts of the gradients as previously documented (Figure 3A, B; [16]). In gradient fractions with heads as the starting tissue (Figure 3A), the ER is predominantly found in fractions 8-14, while residual mitochondrial membrane lies mostly in fractions 6-12. Nrv is seen from fractions 8-20 overlapping with the ER. Based on the extent of this overlap and prior characterization of these gradients [16] we operationally define the PM pool in these gradients to be in fractions 15-20. In gradient fractions using embryos as the starting material (Figure 3B) similar results are obtained: The ER is predominantly found in fractions 6-13 while mitochondrial membrane lies in fractions 5-10. In both preparations, Golgi is typically in fractions 4-10 (Additional File 1, panel A, and [16]), while post Golgi compartments detected by anti-HRP staining extend from the Golgi region through to the PM fractions (Additional File 1, panel B). α-Spectrin can be seen throughout the gradient, although it shows a peak in the Golgi and PM fractions as previously described (4-8 and 15-20 respectively; [16]).

**Figure 4:**
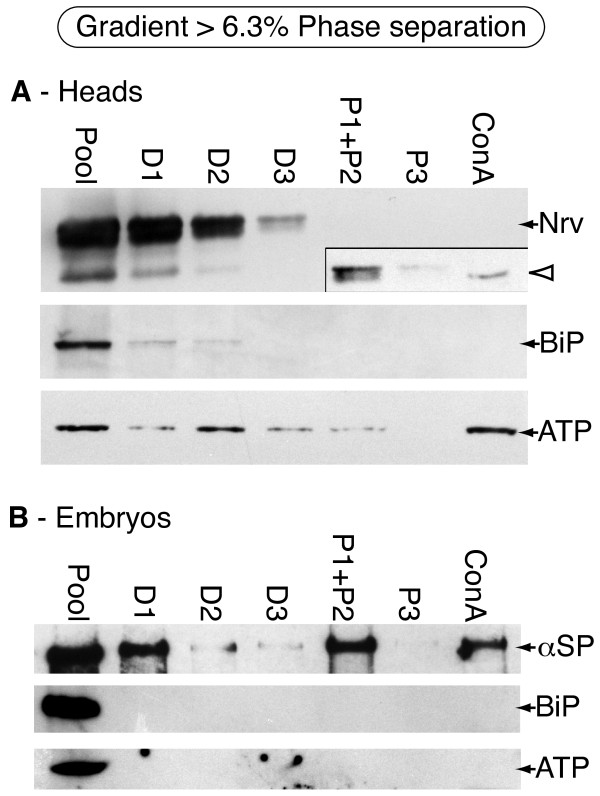
**Immunoblot analysis of samples fractionated by density gradient centrifugation followed by two phase affinity partitioning using 6.3%PEG/Dextran**. **A - **Fractionation of microsomes prepared from heads. Most Nervana is left behind in the dextran fractions, although a 60× exposure shows that a low yield of Nervana is found in the eluted ConA fraction (arrowhead in inset). BiP is no longer detectable in this combined preparation, demonstrating the utility of pre-fractionation on an Optiprep gradient. Residual ATP synthase is detected in the eluted fraction. *Labels*: Pool - pooled fractions 15-20 from the initial Optiprep gradient. Other labels are as described in figure 2A. *Loading*: Equivalent amounts of all fractions were loaded. *Detection*: normal sensitivity chemiluminescence (Nrv), high sensitivity chemiluminescence (BiP, ATP synthase). **B - **Fractionation of microsomes prepared from 0-15 hr embryos. Some α-Spectrin is recovered in the eluted fraction although some seems to have partitioned into dextran phase D1 and some has been excluded from the ConA as seen in the PEG phase (P1+P2). Neither BiP, nor ATP synthase is detectable in the eluted ConA fraction. *Labels*: same as A. *Loading*: Equivalent amounts of all fractions were loaded. *Detection: *high sensitivity chemiluminescence (α-Spectrin, BiP and ATP synthase). N.B. 'high' sensitivity detection reagents are 100-1000× more sensitive than the 'normal' sensitivity substrates and were used to detect even low-level residual contamination.

Immunoblot analysis of the pooled fractions 15-20 shows that contaminating BiP and ATP synthase are still present in low amounts (Pool, Figure 4A, B). Enzyme assays indicate that the yields of ALP, SDH and CCR in the pool are 0.7%, 0.8% and 1.1% respectively (Table 1). As expected, the persistence of the ER and mitochondrial membrane in plasma membrane fractions indicates that density gradient centrifugation alone is not the best method to obtain pure plasma membranes. However, the level of these compartments in the PM region is substantially reduced as seen by immunoblot analyses, suggesting that this might be a good preliminary enrichment for 2PAP.

### Combined density gradient centrifugation and aqueous two-phase affinity partitioning I (6.3% PEG/Dextran)

Since neither of these methods alone produced a satisfactory enrichment for PM, we decided to combine density gradient centrifugation and 2PAP. Thus the PM pool of fractions 15-20 from the gradient was subjected to 2PAP, and analyzed as before except that we switched to high sensitivity chemiluminescence substrates with 100-1000 times the sensitivity of the previous analyses to assess ER and mitochondrial contamination (Figure 4A, B). The level of BiP in the ConA eluate for both heads and embryos is now below the level of detection, suggesting that the combined preparation has significantly increased PM purity. Low levels of mitochondrial membrane persisted in head preparations but not embryos, perhaps reflecting slightly greater compartment mixing due to the head isolation protocol (ConA, Figure 4A, B). The enzyme yields corroborate these results with 50% of the PM (ALP activity) in the input pool being recovered in the ConA eluate, whereas the majority of the residual mitochondrial and ER membrane are eliminated (70% of the SDH and 87% of the CCR respectively; Table 1). Overall, we recover 0.4% of the ALP yield in the ConA pellet after combining the two methods, which is comparable to that obtained when 2PAP is used alone (0.55%); however, we observe a reduction in the yield of CCR. We conclude that pre-enrichment for PM by density gradient fractionation results in a significant increase in PM purity after 2PAP. However, significant amounts of both Nrv and α-Spectrin are still lost through partitioning into the dextran phases.

### Optimization of PEG/Dextran concentration for aqueous two-phase affinity partitioning

**Figure 5:**
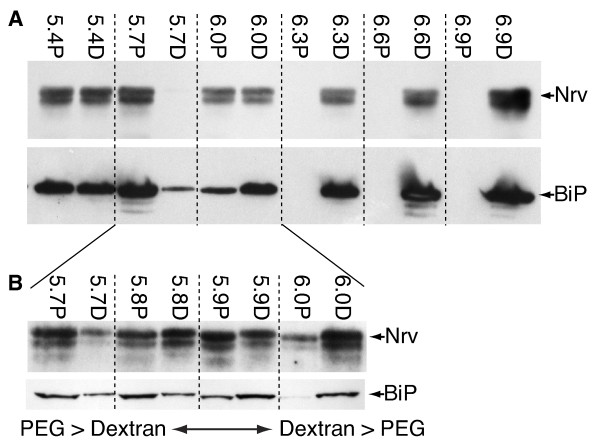
**Optimization of PEG/Dextran concentrations to be used for two phase affinity partitioning**. **A**. Immunoblot analysis of partitioning of Nervana and BiP into PEG (P) and Dextran (D) at different concentrations of PEG/Dextran (from 5.4% PEG/Dextran to 6.9% PEG/Dextran). At concentrations above 6.0-6.3% Nervana partitions into the dextran phase preferentially while at 6.0% and below Nervana is found in both phases. BiP behaves identically. The significant drop in microsome partitioning into dextran seen between 5.7% and 6.3% is seen in all preparations but can be variable in extent, as illustrated in B. **B**. The same experiment performed for a range of percentages from 5.7-6.0% PEG/Dextran reveals a fairly abrupt transition between 5.8% and 5.9%. While a majority of both markers is seen in the PEG fraction at 5.7% and in the Dextran phase at ≥ 6.3%, the precise fractionation behaviour in the transition zone is somewhat variable. The Nrv and BiP blots shown in this panel are from different preparations to illustrate this phenomenon (see text for further discussion).

Despite successfully eliminating the ER, substantial loss of PM markers by non-specific partitioning into the dextran phases during equilibration clearly reduced the yield in the final ConA fraction. In order to increase our PM yield, we decided to test the effect of various PEG/Dextran concentrations on the partitioning of the marker proteins for the PM and ER (Figure 5). Since the spectrum of membrane lipids in vertebrates and invertebrates are significantly different [29-31], the optimal concentrations of PEG and Dextran for 2PAP derived for vertebrate analyses may well be inappropriate for invertebrates. Moreover, because we are pre-enriching for PM through the use of a density gradient we need be less concerned about differential partitioning of PM from endomembane based upon their relative solubility in the PEG and dextran phases. Thus our goal need only be to maximize the fractionation of membrane into the PEG phase from which the ConA-Dextran could effect the final purification.

To optimize the yield of PM in the PEG phase, we performed an experiment on unfractionated P100 microsomes from heads, using a range of PEG/Dextran concentrations from 5.4-6.9%. To standardize the input a single P100 preparation was evenly split into individual 1-g systems made up with the indicated PEG/Dextran concentrations (Figure 5). Immunoblot analyses on the upper PEG (P) phases and lower Dextran (D) phases for each concentration used shows that at PEG/Dextran concentrations of 6.3% and above Nrv partitions exclusively into the dextran phase, whereas from 6.0% and below the prominent fraction is found in the PEG fraction (Figure 5A). Interestingly, at around 5.7% the majority of Nrv is always found in the PEG fraction. It is striking that the partitioning of the ER (BiP) mimics that of Nrv at all PEG/Dextran concentrations suggesting that the PEG/Dextran mixture does not significantly enrich for PM on its own as it does with vertebrate sources. To further investigate this transition we performed experiments interpolating values between 5.7% and 6.0%. There is always a transition between these two values, but the nominal percentage at which this occurs may vary between 5.8% and 6.0% (Figure 5B). This variability may arise from very slight differences in the actual percentage that can easily arise during weighing to make up each system, or perhaps from slight variation in protein content in each microsomal preparation. To maximize our final yield of PM in the PEG fraction, and to ensure maximum reproducibility we decided to use a 5.7% PEG/Dextran mixture.

### Aqueous two-phase affinity partitioning II (5.7% PEG/Dextran)

**Figure 6:**
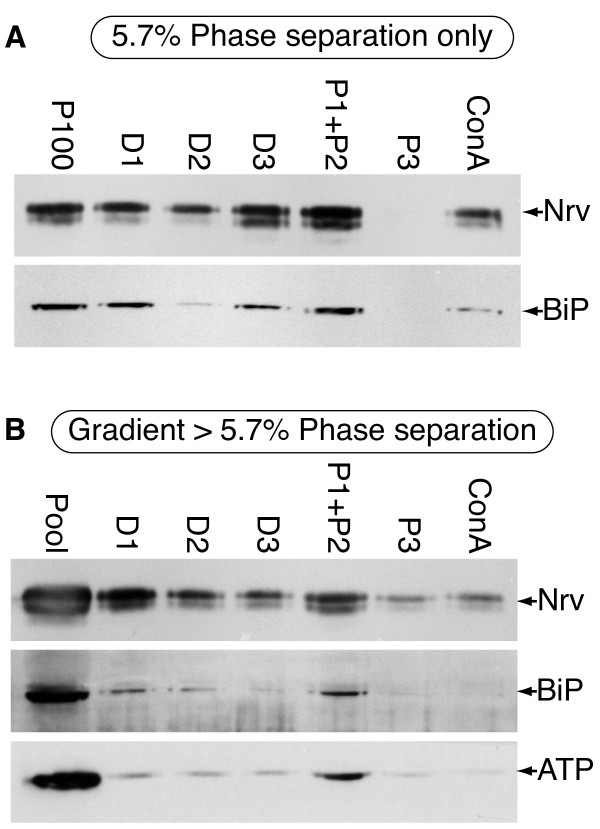
**Immunoblot analysis of head microsomes fractionated by two phase affinity partitioning alone or by density gradient centrifugation followed by two phase affinity partitioning at 5.7% PEG/Dextran**. **A **- Fractionation by two-phase affinity partitioning alone. The yield of Nervana is significantly improved (compare to Figure 4A.) However, BiP is still present in the ConA fraction indicating that 2PAP alone at the 5.7% concentration is still insufficient to produce high purity PM. *Labeling*: same as figure 2. *Loading*: All samples have equivalent loading, except P100, which was 1/5^th ^of the others. **B **- Fractionation by two phase affinity partitioning following an initial density gradient fractionation. The Nervana is still found in the ConA sample, but this fraction no longer contains detectable BiP. Small amounts of residual ATP synthase are still present. *Labeling*: same as figure 4. *Loading*: Equivalent amounts of all fractions were loaded.

We next applied the optimized 5.7% PEG/Dextran percentage to a 2PAP only protocol. This results in a significant increase in Nrv yield in the ConA fraction (Figure 6A; compare with Figure 4A); however, a readily detectable amount of BiP is still seen in this same fraction. Based upon these results, we decided to combine density gradient centrifugation with 2PAP, this time with 5.7% PEG/Dextran phase separation systems.

### Combined density gradient centrifugation and aqueous two-phase affinity partitioning II (5.7% PEG/Dextran)

To apply the optimized combined protocol, head microsomes were subjected to density gradient fractionation as before, and the pool of PM fractions (15-20) from the gradient was subjected to 2PAP with 5.7% PEG/Dextran (Figure 6B). With the optimized concentration the yield of Nrv is still satisfactory, while BiP is reduced to undetectable levels in the final ConA fraction. Probing phases with anti-HRP further corroborates the presence of post-Golgi glycosylation patterns in the final preparation (Additional File 1, panel C). Thus the overall yield and purity of the PM is considerably improved in comparison to that after performing 2PAP with 6.3%PEG/Dextran. Residual ATP synthase is still detected; however, we believe that this represents low levels of membrane mixing (see Discussion).

### Protein identification of affinity purified proteins from *Drosophila *heads

**Figure 7:**
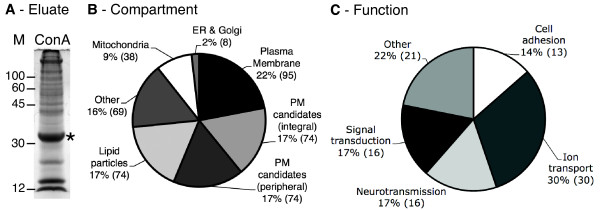
**Protein identification from head PM preparation**. **A - **SDS PAGE analysis of proteins eluted from the ConA in a head PM preparation from ~20 g of flies. Gel is stained with colloidal coomasie blue. Asterisk - prominent ConA band that coelutes from the column, probably due to tetramer disassembly. The presence of this band prevents us from an accurate protein determination from our preparation; however we estimate there to be 50-100 μg protein on this gel. Three such preparations were combined for our MudPIT analysis. **B - **Pie chart showing the breakdown of protein types and compartments identified with high reliability in our MudPIT analysis. Compartment assignments were taken from annotations at Flybase.org in conjunction with our hydropathy analsysis (see text and Table 2). Plasma Membrane -integral or peripheral plasma membrane proteins; PM candidates (integral) - integral membrane proteins with no current assignment to any compartment (see text); PM candidates (peripheral) - have no predicted transmembrane domain and no currently assigned compartment (see text); Other - ribosome, cytoskeleton, synaptic vesicle, cytoplasmic; Mitochondria - mitochondrial; ER & Golgi - endoplasmic reticulum and Golgi apparatus; Lipid particles - lipid particles. Some of these may also be present in mitochondria and/or ER (see text for discussion). **C - **Pie chart showing the functional annotation of proteins identified in the plasma membrane fraction in B. 78% are associated with cell surface activities (Cell adhesion, Ion transport, Neurotransmission, Signal Transduction). See Additional File 5 for the specific assignment of each protein.

In our initial (pre-optimisation) analyses, apparently satisfactory protein concentrations in the final ConA fraction failed to result in reliable protein identifications. Analysis of such preparations by SDS polyacrylamide gel electrophoresis (PAGE) and staining with colloidal coomasie blue revealed that ConA was the only band on the gel (not shown). This indicates that during elution a significant amount of ConA is released from the column. This probably results from the tetrameric structure of this lectin and that coupling to the dextran for each tetramer is *via *less than four subunits: Unattached subunits can dissociate and be released. However, when we analyzed a ConA fraction from an optimized combined preparation by SDS PAGE, a robust protein ladder was seen in addition to the ConA band (Figure 7A).

Proteins from an optimized ConA fraction were trypsin digested and identified by MudPit. 432 protein identifications were accepted with >95% confidence and a <5% false discovery rate (see Methods; Additional Files 2, 3). To evaluate this sub-proteome we performed two analyses. Since, integral membrane proteins can be hard to identify by MS due to poor peptide solubility we subjected the primary sequence of all identified proteins to hydropathy analysis by the method of Kyte and Doolittle [32] to estimate the total number of integral membrane proteins identified in our preparation. For this analysis we used the Protean tool in DNASTAR using a stringent 19 residue window, and only accepted results with at least one region scoring >1.6 that was not at the N-terminus (presumed signal sequence), and was long enough to represent a transmembrane domain, as recommended by Kyte and Doolittle ([32]; annotations are listed in Additional File 2 with cross reference to the hydropathy plots in Additional File 4 where appropriate). 159 out of the 432 proteins identified (37%) were positive in this assay indicating that our digestion and identification protocol is more than satisfactory for the identification of these challenging proteins.

Second, proteins were assigned to sub-cellular compartments based on their existing annotation in Flybase (Flybase.org) in conjunction with our hydropathy analysis. These assignments are broken down in Table 2 and are illustrated in Figure 7B. Satisfyingly, the largest single group of proteins at 22% are annotated in Flybase as 'Plasma membrane', of which a majority are predicted to be integral membrane proteins. Two of the next largest groups we have designated as 'Candidate Plasma Membrane Residents'. This group does not have a currently assigned compartment, but given the high frequency with which we have recovered *bona fide *PM proteins we feel that a majority of these may well reside at the PM. Obviously this will require future experimental verification, and we expect that some will eventually prove to reside elsewhere. This group has been broken down into those that are predicted to be integral membrane proteins (17%) and those with no evidence of a transmembrane domain in our analysis (17%). Together with the *bona fide *PM proteins this provides an upper limit of 56% (22+17+17) for the number of proteins in the PM compartment that we have identified. The remaining proteins have non-PM assignments in Flybase (Table 2; Figure 7): 17% were from lipid particles; 16% were from 'Other' compartments; Only 9% are mitochondrial; Just 2% are ER or Golgi resident. A few of the proteins that are assigned to "lipid particles" by Flybase (Flybase.org; [33]) are also residents of the ER and mitochondria. This reflects the possibility that lipid droplets may originate from either the ER [34] or mitochondria [35]. If we were to reassign these proteins to the ER and mitochondrial categories the percentages of these two compartments would not change dramatically (3% and 16% respectively).

**Table 2:** Membrane and compartment assignments for identified proteins.

Category	Compartment	Flybase annotation	Percent integral^1^	Number of proteins in category	Percentage of all proteins
Plasma Membrane	Plasma membrane	Plasma membrane	60% (57/95)	95	22%

Candidate Plasma Membrane Residents (Integral)	Not assigned	Membrane	50% (10/21)	74	17%
				
		Integral to membrane	100% (21/21)		
				
		None but we predict ≥ 1 transmembrane domain	100% (43/43)		

Candidate Plasma Membrane Residents (Peripheral)	Not assigned	Membrane	N/A^2 ^11 proteins	74	17%
				
		None with no predicted transmembrane domains	N/A^2 ^63 proteins		

Lipid particles	Lipid particles	Lipid particles	22% (16/74)	74	17%

Other	Cytoplasm and Nucleus	Cytoplasm Cytoskeleton Nucleus Ribosomes	6% (4/69)	69	16%

Mitochondrial	Mitochondrial	Mitochondrial	18% (5/38)	38	9%

Endoplasmic reticulum and Golgi	Endoplasmic reticulum and Golgi	Endoplasmic reticulum and Golgi	38% (3/8)	8	2%

All compartments			37% (159/432)	432	100%

^1^Proteins predicted by hydropathy analysis to have ≥ 1 transmembrane domain

^2^Not Applicable - neither category has any predicted integral membrane proteins.

The table shows the number of proteins assigned to compartments based on their Flybase annotation in conjunction with our hydropathy analysis. The biggest single category has been annotated in Flybase as 'Plasma membrane'. The next two categories we predict will also contain a substantial fraction, which are in the PM compartment; however, this will obviously require experimental verification by interested investigators (see text for discussion).

We further classified the definitive PM proteins on the basis of their cellular function (FlyBase; Figure 7C). The categories are: 'Cell adhesion', 'Neurotransmission' (includes proteins involved in neurotransmitter transport and secretion), 'Signal transduction', 'Ion transport', and 'Other' (includes proteins that are structural, involved in cell polarity, ion/protein binding, have roles in axogenesis and central nervous system development). The largest category of proteins are involved in ion transport (30%) and include the likes of excitatory amino acid transporter 1 and Na^+^/K^+ ^ATPase (α subunit). 17% each were involved in signal transduction and neurotransmission, and 13% in cell adhesion. Proteins involved in signal transduction include G-proteins as well as proteins in the InaD-signaling complex. Among those in the category 'neurotransmission' are Syntaxin 1 as well as Neurexins 1 and 4. Cell adhesion proteins include Fasciclins 1 and 3, N-cadherin and Contactin. Most of these proteins were also found in the rat-brain plasma membrane preparation by Schindler *et al*. [11]. A complete list of the plasma membrane proteins along with their functional categories is provided (Additional File 5).

## Discussion

The success of aqueous two-phase affinity purification (2PAP) to isolate high purity plasma membranes from rat livers, lungs and brain [7-11] suggested that this would be an excellent technique to combine with the sophisticated genetic approaches possible with the invertebrate *Drosophila melanogaster*. To date, plasma membrane isolation from *Drosophila *tissues has been based on density gradient centrifugation [36-38], and to the best of our knowledge the use of 2PAP has not yet been reported for this model. Here we adapt 2PAP to the fly model. In contrast to reports using 2PAP with vertebrate tissue sources, we found that the initial PEG/Dextran partitioning steps in the 2PAP technique are not effective in differentially partitioning PM from ER membranes. In addition, a necessary substitution with respect to most recent 2PAP protocols [11] is the substitution of the lectin Concanavalin A (ConA) for wheat germ agglutinin (WGA) as a method to select for glycoprotein-containing microsomes. This is because protein glycosylation patterns in insects are simple and rich in mannose [13]. However, this lectin is less specific for post-ER proteins. To overcome these two problems we extend the 2PAP technique through the use of a pre-enrichment density gradient centrifugation step to produce PM of high purity.

2PAP is reported to enrich for PM in part by preferential enrichment in the PEG fraction [6]; however, this was not our experience with *Drosophila *membranes. A key difference in partitioning behavior may arise from differences in lipid composition between vertebrates and insects and as well as differences in the way lipids are segregated between organelles. In mammals the plasma membrane is rich in phospholipids (sphingomyelin, phosphatidylcholine, phosphatidylethanolamine, phosphatidylserine and phosphatidylinositol) along with large amounts of cholesterol [39]. In contrast, intracellular compartments have a higher percentage of phosphatidylcholine and phosphatidylethanolamine, much-reduced sphingomyelin and phosphatidylserine, and much lower cholesterol levels (*ibid*). Whereas, plasma membranes from *Drosophila *contain similar lipid head groups to vertebrates, they have shorter fatty acid chains [30,31], and the major sterol is ergosterol [30]. We have found no reports comparing the lipid profile of PM and intracellular membranes in *Drosophila*; however, a study on mosquito *Aedes aegypti *(also of the order *Diptera*), suggests that sphingosine containing lipids are not significantly enriched in the PM, nor are phosphatidylcholine and phosphatidylethanolamine enriched on internal compartments [40]. If this lack of distinction between the types of bulk lipids in different compartments holds for *Drosophila *as well, this might explain the lowered efficiency of PM segregation by PEG/Dextran compared to mammalian tissue sources. This problem, coupled with the lower discrimination by ConA, fully explains our initial results and the continued presence of ER in the final affinity-selected fraction. To solve this problem we used density gradient centrifugation to separate the plasma membrane from intracellular membranes. Since this is based on density and not on differential solubility of the microsomes, this effectively removes the vast majority of ER. The small amount that does carry through with the PM fractions on the gradient is removed with the help of the affinity step in 2PAP. Because differential lipid solubility in the PEG/Dextran system is not an essential enrichment step in our implementation of 2PAP we further adjusted our method to increase the fraction of PM in the PEG phase (by changing from 6.3% to 5.7% PEG/Dextran mixtures), boosting our overall yield of PM. This is probably an important optimization step in adaptation to other models. In vertebrates, the use of PEG/Dextran concentrations above 5.7% can result in a slight enrichment of PM over ER in the PEG phase during the equilibration steps [11]; however, in our hands this reduces the PM yield by about 50%. Combining more fractions from the gradient could offset this, but only at the cost of PM purity. Thus we favour our current strategy.

Although our method eliminates almost all ER, low-level contamination with mitochondrial proteins remains (Figure 6B). In the hope of keeping most of the mitochondria intact we adjusted our extraction conditions buffer to one that should optimize *Drosophila *mitochondrial integrity [15]. Intact mitochondria should be removed by early low speed spins, and the major peak of residual mitochondrial membrane in the Optiprep gradients is well below our PM pool (see Figure 3). Thus, the presence of low levels of mitochondrial membranes in the lighter PM fractions is probably due to organelle fragmentation and mixing with PM during initial homogenization. However, we note that this is not the only possibility: ConA has been reported to have affinity for some mitochondrial membrane proteins [41,42] and [43], and some mitochondrial proteins have been reported to also reside at the plasma membrane (seen in rat livers [44]). Thus, we conclude that such contamination may be unavoidable or possibly of functional significance.

The original 2PAP technique gives a preparation containing 34-42% PM proteins from rat brains [11]. In comparison, 22% of our proteins are annotated in Flybase as 'Plasma membrane'. If we add to this the 34% of proteins in our preparation that have not yet been assigned to a compartment by other techniques this suggests we may have as many as 56% PM proteins in our preparation and conclude that that our likely yield for the PM proteome as a whole is in the 22-56% range. This is very comparable to these previous efforts.

Previous work to define the total *Drosophila *brain and eye proteome through 2D gel analysis on dissected tissues did not enrich for plasma membrane [45]. In comparison to our PM sub-proteome, only ~15% of the definitive plasma membrane proteins identified in our preparation were found in the brain/eye lists. Even allowing for the fact that perhaps 15% of the tissue in our whole head extracts is not from brain/eye tissue, the fact that we identified so many more proteins in this category emphasizes the value of enriching for the study of the PM.

Finally, in extending this method to whole flies and to other stages in the life cycle, additional lectins should perhaps be considered. While glycosylation in adult brains is mostly mannosidic or paucimannosidic [14], ~40% of N-glycans in adult flies are core α-1,6 fucosylated [46]. *Aleuria aurantia *lectin for example has affinity for α 1-2, -3, -4 and α 1-6 fucosylated glycans [12,47], and might be of some utility, perhaps in combination with ConA. However, we note that Nervana and Fasciclin 1, which are both known to have core fucosylation, are present in our preparation suggesting that other groups on such proteins still allow their purification by our method.

## Conclusion

2PAP has been reported as a simple and efficient technique to isolate pure plasma membranes from vertebrate tissues [7-11]. Our results demonstrate that 2PAP alone is not sufficient to purify plasma membranes from the invertebrate *Drosophila melanogaster*. In comparison, density gradient centrifugation, an established method of plasma membrane enrichment in *Drosophila *results in significant overlap between the plasma membrane fractions and those of the endoplasmic reticulum. However, we demonstrate that a combination of these two techniques is effective.

*Drosophila *is an established model system for developmental studies and an emerging model system for neurological disorders [48-50]. Our adaptation of the 2PAP technology provides the opportunity to focus on the cell surface proteome of the *Drosophila *at any stage of development, and to combine this with the elegant genetic techniques for which the fly is justly famous. In addition, our results emphasize the importance of optimizing two key steps: The optimization of membrane partitioning in the PEG/Dextran mixture in light of variations in bulk membrane lipid composition, and careful choice of the correct lectin for the affinity purification step in light of species-specific variation in glycosylation patterns. This points the way for further adaptations of this method to other models.

## Abbreviations

2PAP: Aqueous two-phase affinity purification; MudPIT: Multidimensional Protein Identification Technology; PM: Plasma membrane; PEG: Polyethylene glycol; WGA: Wheat germ agglutinin; ConA: ConcanavalinA; ER: Endoplasmic reticulum; Nrv: Nervana; HB: Homogenization buffer; ALP: Alkaline phosphatase; SDH: Succinate dehydrogenase; CCR: Cytochrome c reductase (NADPH); MS/MS: Tandem mass spectrometry.

## Authors' contributions

MRK performed all of the membrane purifications, prepared all samples for mass spectrometry and performed the functional annotation of the identified proteins. BAS performed the protein identification by Mass spectrometry. GHT is the principal investigator. All three authors participated in drafting the manuscript. All authors read and approved the final manuscript.

## Supplementary Material

Additional file 1 **Golgi and post-Golgi proteins in head microsomes fractionated by density gradient centrifugation followed by two phase affinity partitioning at 5.7% PEG/Dextran**. **A - **Fractionated microsomes prepared from heads and probed for the Golgi protein Lava lamp. Two isoforms of Lava lamp are detected (arrows) at ~170 kDa and ~315 kDa. On average Golgi membrane is heavier than the peak ER fractions as expected [16]); double headed arrow; see Figure 3), but some overlap is seen especially with the larger isoform which has a bimodal distribution. **B - **Fractionated microsomes prepared from heads and probed with anti-Horseradish Peroxidase (HRP). The epitopes recognized by anti-HRP depend on the presence of *N*-glycan core α1,3-linked fucose [46] and thus detects proteins in trans-Golgi and post-Golgi compartments. The prominent epitope at 42 kDa is thought to be our PM marker Nervana [28]. Trans- and post-Golgi proteins detected by anti-HRP extend from the Golgi fractions through to the lightest region of the gradient as seen with the fully glycosylated Nervana isoforms (see Figure 3A). **C **- Fractionation by two phase affinity partitioning following an initial density gradient fractionation and probed with anti-HRP. The most prominent band behaves the same way as Nervana (see Figure 6B) and probably *is *Nervana (see [28]). An overexposure of the final ConA eluate (ConA over) is included to show that other anti-HRP detectable proteins are also present in the PM fraction. *Labeling*: same as figure 3 for A and B and figure 4 for C. *Loading*: Equivalent amounts of all fractions were loaded. Click here for file

Additional file 2 **MudPIT identification of proteins purified by combination of density gradient centrifugation and 2PAP from *Drosophila *head microsomes**: This table includes a list of all the proteins purified by our optimized protocol and identified with > 95% confidence. The sub-cellular compartment in which each protein can be found is indicated, along with the number of peptides identified. For single-peptide identifications, the sequence, precursor m/z and score of the peptide have been provided. Cross-references to Additional Files 3 and 4 are also included. Click here for file

Additional file 3 **Spectra for proteins identified by single-peptide hit**: This table provides the matched peptide and spectrum for all single-peptide identifications. Click here for file

Additional file 4 **Hydropathy plots for proteins predicted to have transmembrane domains**: This table provides the hydropathy plots of all those proteins predicted to have transmembrane domains by the method of Kyte and Doolittle. Click here for file

Additional file 5 **Functional categorisation of proteins identified as residents of the plasma membrane**: This table classifies the plasma membrane proteins listed in Additional File 2 on the basis of their cellular function. Click here for file

## References

[B1] Overington JP Al-Lazikani B Hopkins AL How many drug targets are there? Nat Rev Drug Discov 2006 5 12 993 996 10.1038/nrd2199 17139284

[B2] Josic D Clifton JG Kovac S Hixson DC Membrane proteins as diagnostic biomarkers and targets for new therapies Curr Opin Mol Ther 2008 10 2 116 123 18386223

[B3] Josic D Clifton JG Mammalian plasma membrane proteomics Proteomics 2007 7 16 3010 3029 10.1002/pmic.200700139 17654460

[B4] Lawson EL Clifton JG Huang F Li X Hixson DC Josic D Use of magnetic beads with immobilized monoclonal antibodies for isolation of highly pure plasma membranes Electrophoresis 2006 27 13 2747 2758 10.1002/elps.200600059 16739230

[B5] Zhang L Wang X Peng X Wei Y Cao R Liu Z Xiong J Ying X Chen P Liang S Immunoaffinity purification of plasma membrane with secondary antibody superparamagnetic beads for proteomic analysis J Proteome Res 2007 6 1 34 43 10.1021/pr060069r 17203946

[B6] Schindler J Nothwang HG Aqueous polymer two-phase systems: effective tools for plasma membrane proteomics Proteomics 2006 6 20 5409 5417 10.1002/pmic.200600243 16972286

[B7] Persson A Johansson B Olsson H Jergil B Purification of rat liver plasma membranes by wheat-germ-agglutinin affinity partitioning Biochem J 1991 273 Pt 1 173 177 1703408 10.1042/bj2730173 PMC1149895

[B8] Persson A Jergil B Purification of plasma membranes by aqueous two-phase affinity partitioning Anal Biochem 1992 204 1 131 136 10.1016/0003-2697(92)90151-V 1381154

[B9] Ekblad L Jergil B Localization of phosphatidylinositol 4-kinase isoenzymes in rat liver plasma membrane domains Biochim Biophys Acta 2001 1531 3 209 221 11325612 10.1016/s1388-1981(01)00103-2

[B10] Abedinpour P Jergil B Isolation of a caveolae-enriched fraction from rat lung by affinity partitioning and sucrose gradient centrifugation Anal Biochem 2003 313 1 1 8 10.1016/S0003-2697(02)00561-4 12576051

[B11] Schindler J Lewandrowski U Sickmann A Friauf E Nothwang HG Proteomic analysis of brain plasma membranes isolated by affinity two-phase partitioning Mol Cell Proteomics 2006 5 2 390 400 16249173 10.1074/mcp.T500017-MCP200

[B12] Iskratsch T Braun A Paschinger K Wilson IB Specificity analysis of lectins and antibodies using remodeled glycoproteins Anal Biochem 2009 386 2 133 146 10.1016/j.ab.2008.12.005 19123999

[B13] Rendić D Wilson IBH Paschinger K The glycosylation capacity of insect cells Croatica Chemica Acta 2008 81 1 7 21

[B14] Koles K Lim JM Aoki K Porterfield M Tiemeyer M Wells L Panin V Identification of N-glycosylated proteins from the central nervous system of Drosophila melanogaster Glycobiology 2007 17 12 1388 1403 10.1093/glycob/cwm097 17893096

[B15] Beziat F Touraille S Debise R Morel F Petit N Lecher P Alziari S Biochemical and molecular consequences of massive mitochondrial gene loss in different tissues of a mutant strain of Drosophila subobscura J Biol Chem 1997 272 36 22583 22590 10.1074/jbc.272.36.22583 9278413

[B16] Papoulas O Hays TS Sisson JC The golgin Lava lamp mediates dynein-based Golgi movements during Drosophila cellularization Nat Cell Biol 2005 7 6 612 618 10.1038/ncb1264 15908943

[B17] Ashburner M Drosophila A Laboratory Handbook 1989 Woodbury: Cold Spring Harbor Laboratory Press

[B18] Laemmli UK Cleavage of structural proteins during the assembly of the head of bacteriophage T4 Nature 1970 227 5259 680 685 10.1038/227680a0 5432063

[B19] Fritz JD Swartz DR Greaser ML Factors affecting polyacrylamide gel electrophoresis and electroblotting of high-molecular-weight myofibrillar proteins Anal Biochem 1989 180 2 205 210 10.1016/0003-2697(89)90116-4 2817350

[B20] Akcakaya H Aroymak A Gokce S A quantitative colorimetric method of measuring alkaline phosphatase activity in eukaryotic cell membranes Cell Biol Int 2007 31 2 186 190 10.1016/j.cellbi.2006.11.014 17207647

[B21] Munujos P Coll-Canti J Gonzalez-Sastre F Gella FJ Assay of succinate dehydrogenase activity by a colorimetric-continuous method using iodonitrotetrazolium chloride as electron acceptor Anal Biochem 1993 212 2 506 509 10.1006/abio.1993.1360 8214593

[B22] Zhao Z Zhang W Stanley BA Assmann SM Functional proteomics of Arabidopsis thaliana guard cells uncovers new stomatal signaling pathways Plant Cell 2008 20 12 3210 3226 10.1105/tpc.108.063263 19114538 PMC2630442

[B23] Shilov IV Seymour SL Patel AA Loboda A Tang WH Keating SP Hunter CL Nuwaysir LM Schaeffer DA The Paragon Algorithm, a next generation search engine that uses sequence temperature values and feature probabilities to identify peptides from tandem mass spectra Mol Cell Proteomics 2007 6 9 1638 1655 10.1074/mcp.T600050-MCP200 17533153

[B24] Tang WH Shilov IV Seymour SL Nonlinear fitting method for determining local false discovery rates from decoy database searches J Proteome Res 2008 7 9 3661 3667 10.1021/pr070492f 18700793

[B25] Montreuil J Vliegenthart JFG Schachter H Glycoproteins 1995 Amsterdam; New York: Elsevier

[B26] Vaccari T Rusten TE Menut L Nezis IP Brech A Stenmark H Bilder D Comparative analysis of ESCRT-I, ESCRT-II and ESCRT-III function in Drosophila by efficient isolation of ESCRT mutants J Cell Sci 2009 122 Pt 14 2413 2423 10.1242/jcs.046391 19571114 PMC2704878

[B27] Rustin GJ Wilson PD Peters TJ Studies on the subcellular localization of human neutrophil alkaline phosphatase J Cell Sci 1979 36 401 412 457815 10.1242/jcs.36.1.401

[B28] Sun B Salvaterra PM Characterization of nervana, a Drosophila melanogaster neuron-specific glycoprotein antigen recognized by anti-horseradish peroxidase antibodies J Neurochem 1995 65 1 434 443 7540667 10.1046/j.1471-4159.1995.65010434.x

[B29] Zachowski A Phospholipids in animal eukaryotic membranes: transverse asymmetry and movement Biochem J 1993 294 Pt 1 1 14 8363559 10.1042/bj2940001 PMC1134557

[B30] Rietveld A Neutz S Simons K Eaton S Association of sterol- and glycosylphosphatidylinositol-linked proteins with Drosophila raft lipid microdomains J Biol Chem 1999 274 17 12049 12054 10.1074/jbc.274.17.12049 10207028

[B31] Stark WS Lin TN Brackhahn D Christianson JS Sun GY Phospholipids in Drosophila heads: effects of visual mutants and phototransduction manipulations Lipids 1993 28 1 23 28 10.1007/BF02536355 8446007

[B32] Kyte J Doolittle RF A simple method for displaying the hydropathic character of a protein J Mol Biol 1982 157 1 105 132 10.1016/0022-2836(82)90515-0 7108955

[B33] Beller M Riedel D Jansch L Dieterich G Wehland J Jackle H Kuhnlein RP Characterization of the Drosophila lipid droplet subproteome Mol Cell Proteomics 2006 5 6 1082 1094 10.1074/mcp.M600011-MCP200 16543254

[B34] Thiele C Spandl J Cell biology of lipid droplets Curr Opin Cell Biol 2008 20 4 378 385 10.1016/j.ceb.2008.05.009 18606534

[B35] Caetano FH Zara FJ Gregório EA The origin of lipid droplets in the post-pharyngeal gland of *Dinoponera australis *(Formicidae: Ponerinae) Cytologia 2002 67 301 308 10.1508/cytologia.67.301

[B36] Rao RP Yuan C Allegood JC Rawat SS Edwards MB Wang X Merrill AH Jr Acharya U Acharya JK Ceramide transfer protein function is essential for normal oxidative stress response and lifespan Proc Natl Acad Sci USA 2007 104 27 11364 11369 10.1073/pnas.0705049104 17592126 PMC1899189

[B37] Eroglu C Brugger B Wieland F Sinning I Glutamate-binding affinity of Drosophila metabotropic glutamate receptor is modulated by association with lipid rafts Proc Natl Acad Sci USA 2003 100 18 10219 10224 10.1073/pnas.1737042100 12923296 PMC193542

[B38] Jiang QY Gnagey A Tandler B Jacobs-Lorena M Isolation of plasma membranes from Drosophila embryos Mol Biol Rep 1986 11 1 19 24 10.1007/BF00417590 3003563

[B39] van Meer G Voelker DR Feigenson GW Membrane lipids: where they are and how they behave Nat Rev Mol Cell Biol 2008 9 2 112 124 10.1038/nrm2330 18216768 PMC2642958

[B40] Butters TD Hughes RC Phospholipids and glycolipids in subcellular fractions of mosquito *Aedes aegypti *cells In Vitro 1981 17 9 831 838 10.1007/BF02618451

[B41] Chandra NC Spiro MJ Spiro RG Identification of a glycoprotein from rat liver mitochondrial inner membrane and demonstration of its origin in the endoplasmic reticulum J Biol Chem 1998 273 31 19715 19721 10.1074/jbc.273.31.19715 9677401

[B42] Lopez MF Kristal BS Chernokalskaya E Lazarev A Shestopalov AI Bogdanova A Robinson M High-throughput profiling of the mitochondrial proteome using affinity fractionation and automation Electrophoresis 2000 21 16 3427 3440 10.1002/1522-2683(20001001)21:16<3427::AID-ELPS3427>3.0.CO;2-L 11079563

[B43] Distler AM Kerner J Hoppel CL Proteomics of mitochondrial inner and outer membranes Proteomics 2008 8 19 4066 4082 10.1002/pmic.200800102 18763707

[B44] Bae TJ Kim MS Kim JW Kim BW Choo HJ Lee JW Kim KB Lee CS Kim JH Chang SY Lipid raft proteome reveals ATP synthase complex in the cell surface Proteomics 2004 4 11 3536 3548 10.1002/pmic.200400952 15378739

[B45] Matsumoto H Takemori N Thompson JNj Yamamoto MT Komori N *Drosophila *proteome atlas Drosophila Information Service 2007 90 162 164

[B46] Fabini G Freilinger A Altmann F Wilson IB Identification of core alpha 1,3-fucosylated glycans and cloning of the requisite fucosyltransferase cDNA from Drosophila melanogaster. Potential basis of the neural anti-horseadish peroxidase epitope J Biol Chem 2001 276 30 28058 28067 10.1074/jbc.M100573200 11382750

[B47] Tateno H Nakamura-Tsuruta S Hirabayashi J Comparative analysis of core-fucose-binding lectins from Lens culinaris and Pisum sativum using frontal affinity chromatography Glycobiology 2009 19 5 527 536 10.1093/glycob/cwp016 19218400

[B48] Feany MB Bender WW A Drosophila model of Parkinson's disease Nature 2000 404 6776 394 398 10.1038/35006074 10746727

[B49] Link CD Invertebrate models of Alzheimer's disease Genes Brain Behav 2005 4 3 147 156 10.1111/j.1601-183X.2004.00105.x 15810903

[B50] Spradling A Ganetsky B Hieter P Johnston M Olson M Orr-Weaver T Rossant J Sanchez A Waterston R New roles for model genetic organisms in understanding and treating human disease: report from the 2006 Genetics Society of America meeting Genetics 2006 172 4 2025 2032 16636111 10.1093/genetics/172.4.2025 PMC1456383

