# Supplementary material for: Towards a membrane proteome in Drosophila: a method for the isolation of plasma membrane
Source: BMC Genomics. 2010 May 12;11:302. doi: 10.1186/1471-2164-11-302 (PMC2876126; doi:10.1186/1471-2164-11-302)
Supplement: Additional file 1 — Golgi and post-Golgi proteins in head microsomes fractionated by density gradient centrifugation followed by two phase affinity partitioning at 5.7% PEG/Dextran. A - Fractionated microsomes prepared from heads and probed for the Golgi protein Lava lamp. Two isoforms of Lava lamp are detected (arrows) at ~170 kDa and ~315 kDa. On average Golgi membrane is heavier than the peak ER fractions as expected [16]); double headed arrow; see Figure 3), but some overlap is seen especially with the larger isoform which has a bimodal distribution. B - Fractionated microsomes prepared from heads and probed with anti-Horseradish Peroxidase (HRP). The epitopes recognized by anti-HRP depend on the presence of N-glycan core α1,3-linked fucose [46] and thus detects proteins in trans-Golgi and post-Golgi compartments. The prominent epitope at 42 kDa is thought to be our PM marker Nervana [28]. Trans- and post-Golgi proteins detected by anti-HRP extend from the Golgi fractions through to the lightest region of the gradient as seen with the fully glycosylated Nervana isoforms (see Figure 3A). C - Fractionation by two phase affinity partitioning following an initial density gradient fractionation and probed with anti-HRP. The most prominent band behaves the same way as Nervana (see Figure 6B) and probably is Nervana (see [28]). An overexposure of the final ConA eluate (ConA over) is included to show that other anti-HRP detectable proteins are also present in the PM fraction. Labeling: same as figure 3 for A and B and figure 4 for C. Loading: Equivalent amounts of all fractions were loaded. [file 1471-2164-11-302-S1.PDF]

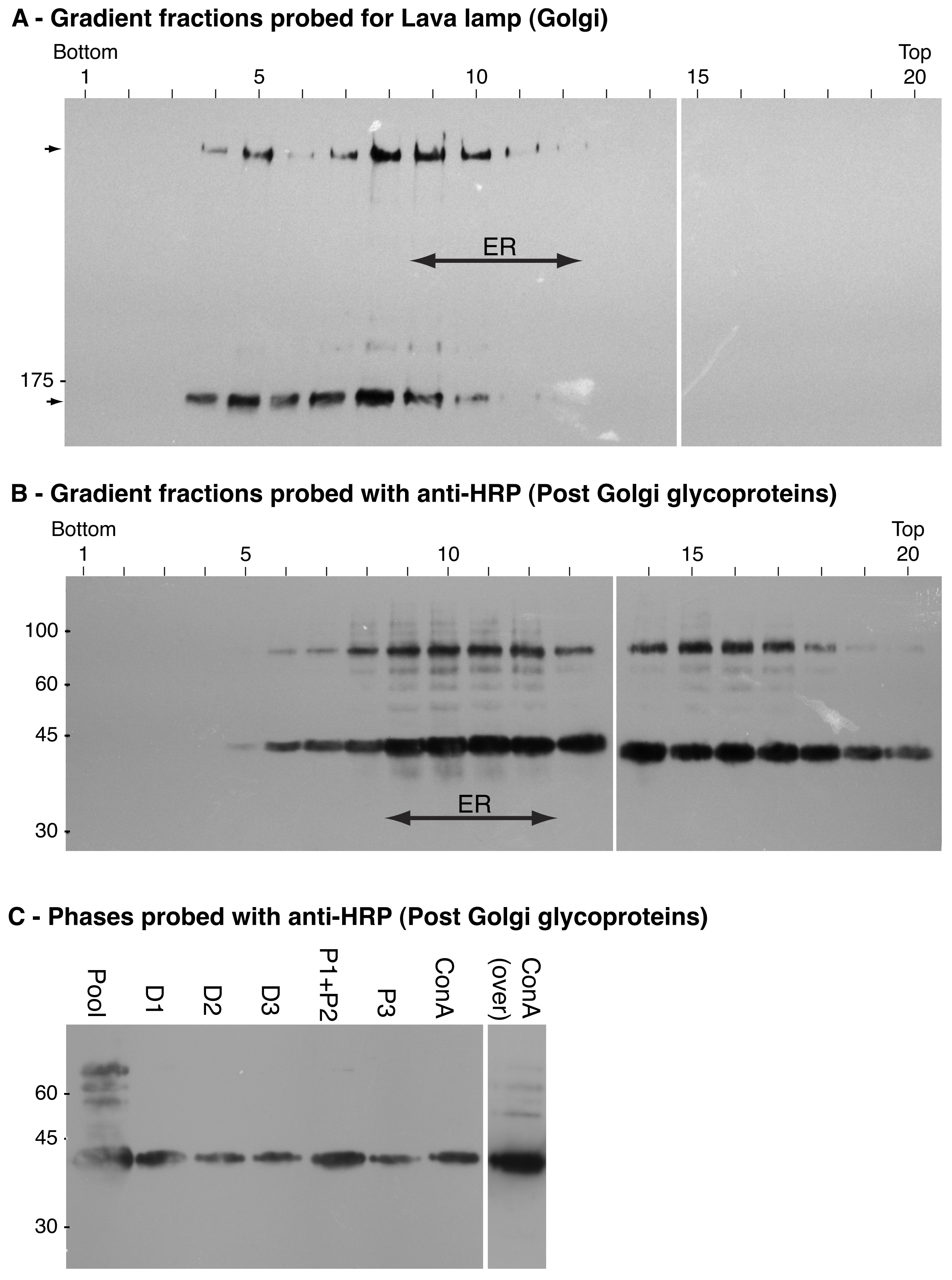

A - Gradient fractions probed for Lava lamp (Golgi)
Bottom
Top
1
5
10
15
20
175
ER
B - Gradient fractions probed with anti-HRP (Post Golgi glycoproteins)
Bottom
Top
1
5
10
15
20
100
60
45
30
ER
C - Phases probed with anti-HRP (Post Golgi glycoproteins)
Pool
D1
D2
D3
P1+P2
P3
ConA
(over)
ConA
60
45
30
