# Supplementary material for: Towards a membrane proteome in Drosophila: a method for the isolation of plasma membrane
Source: BMC Genomics. 2010 May 12;11:302. doi: 10.1186/1471-2164-11-302 (PMC2876126; doi:10.1186/1471-2164-11-302)
Supplement: Additional file 3 — Spectra for proteins identified by single-peptide hit: This table provides the matched peptide and spectrum for all single-peptide identifications. [file 1471-2164-11-302-S3.PDF]

Spectra for single peptide identifications

Spectrum Number	Precursor m/z	Spectrum
1	1566.75	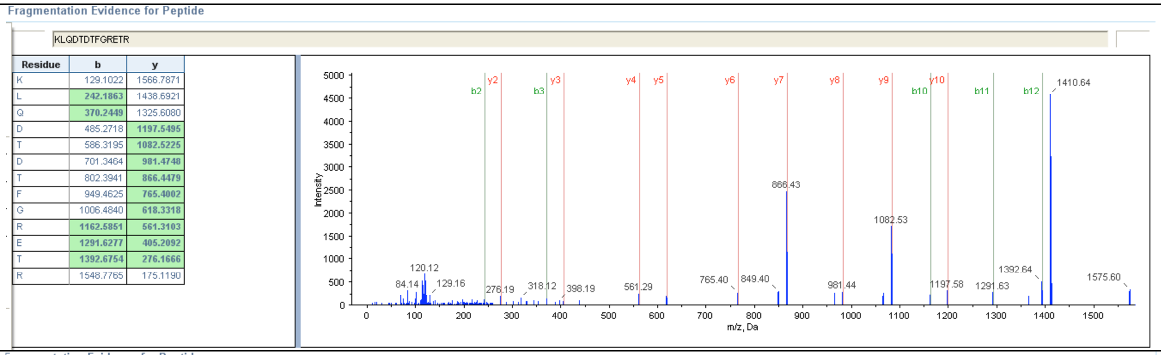
2	2895.373	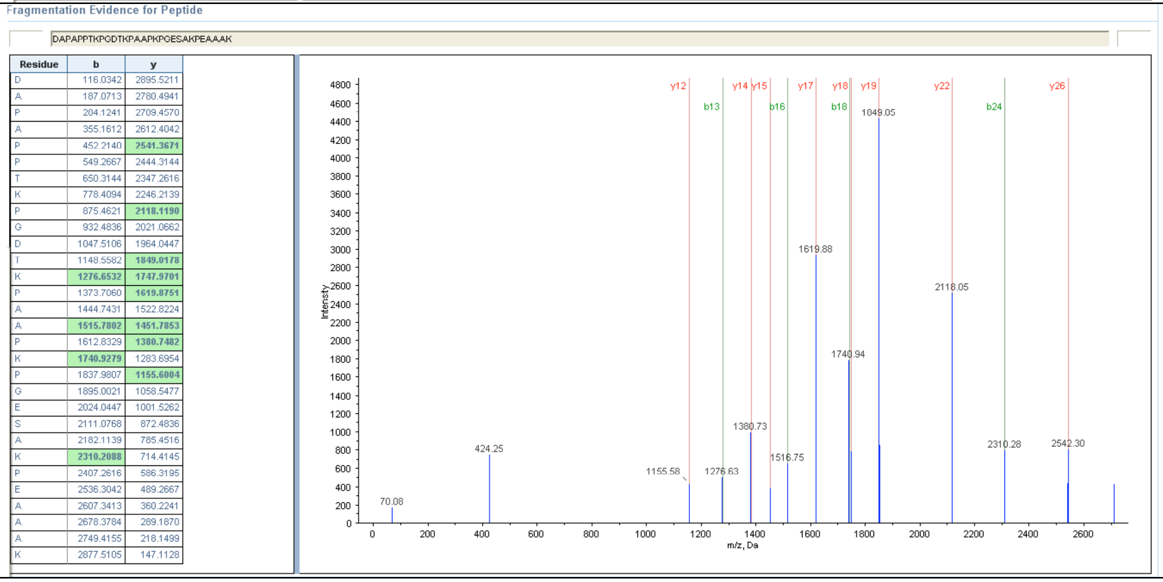

3	996.5359		Fragmentation Evidence for Peptide

AFLNIQYK

Residue	b	y
A	72.0444	996.5513
F	**219.1128**	925.5142
L	**332.1969**	**778.4458**
N	**446.2398**	**665.3617**
I	**559.3239**	**551.3188**
Q	687.3824	**438.2347**
Y	**850.4458**	310.1761
K	978.5407	**147.1128**

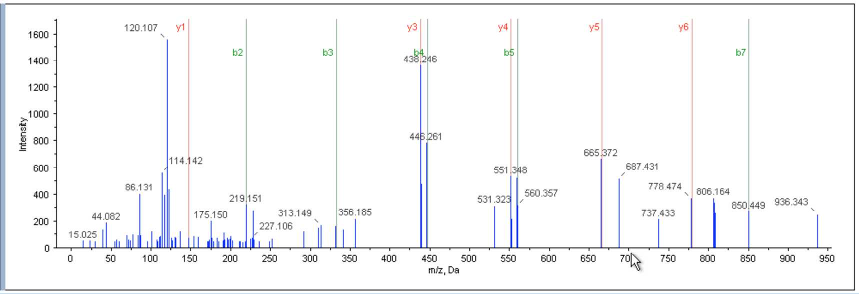

4	1617.679		Fragmentation Evidence for Peptide

SPFSEMSEGSEFQR

Residue	b	y
S	88.0393	1617.6850
P	**185.0921**	1530.6529
F	**332.1605**	**1433.6002**
S	**419.1925**	1286.5318
E	**548.2351**	**1199.4997**
M	**679.2756**	**1070.4571**
S	766.3076	939.4167
E	895.3502	**852.3846**
G	952.3717	**723.3420**
S	1039.4037	**666.3206**
E	1168.4463	**579.2885**
F	1315.5147	**450.2459**
Q	1443.5733	**303.1775**
R	1599.6744	**175.1190**

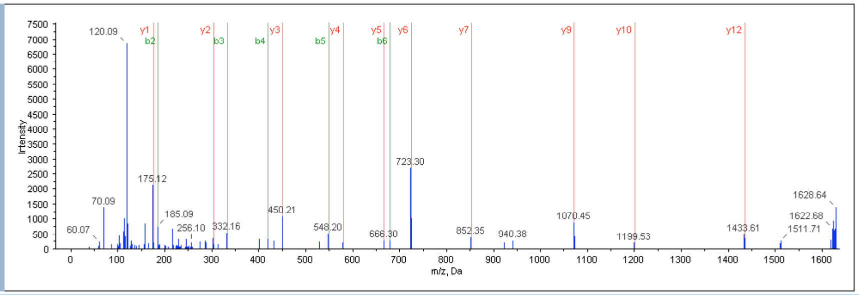

5	2431.168		Fragmentation Evidence for Peptide

QNSVYPDDPEQMVPLKNIESK

Residue	b	y
Q	**129.0659**	2431.1810
N	243.1088	2303.1224
S	**330.1408**	2189.0795
V	**429.2092**	2102.0474
Y	592.2726	2002.9790
P	689.3253	1839.9157
D	804.3523	1742.8629
D	919.3792	**1627.8360**
P	1016.4320	**1512.8090**
E	1145.4746	1415.7563
Q	1273.5331	**1286.7137**
M	1404.5736	1158.6551
V	1503.6420	1027.6146
P	1600.6948	928.5462
L	1713.7789	831.4934
K	1841.8738	718.4094
N	1955.9168	590.3144
I	2069.0008	476.2715
E	2198.0434	363.1874
S	2285.0754	234.1448
K	2413.1704	147.1128

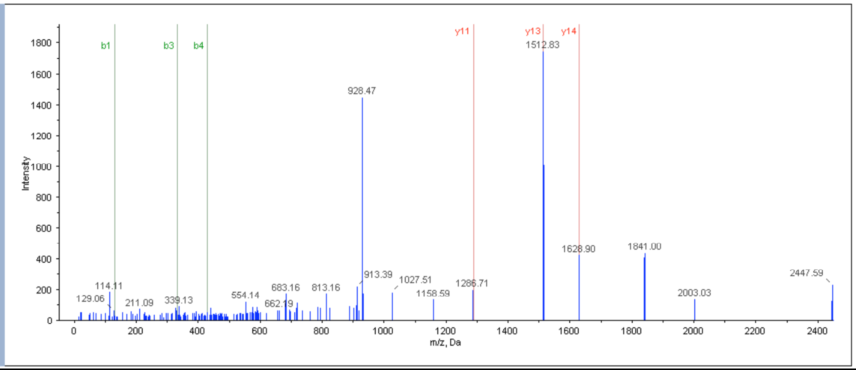

6	2302.078	

Fragmentation Evidence for Peptide

YITEHEQEDYLLTGFTSQK

Residue	b	y
Y	164.0706	2302.0874
I	277.1547	2139.0241
T	378.2023	2025.9400
E	507.2449	1924.8923
H	644.3039	1795.8497
E	773.3464	1658.7908
Q	901.4050	1529.7482
E	1030.4476	1401.6896
D	1145.4746	1272.6470
Y	1308.5379	1157.6201
L	1421.6220	994.5568
L	1534.7060	881.4727
T	1635.7537	768.3886
G	1692.7752	667.3410
F	1839.8436	610.3195
T	1940.8913	463.2511
S	2027.9233	362.2034
Q	2155.9819	275.1714
K	2284.0768	147.1128

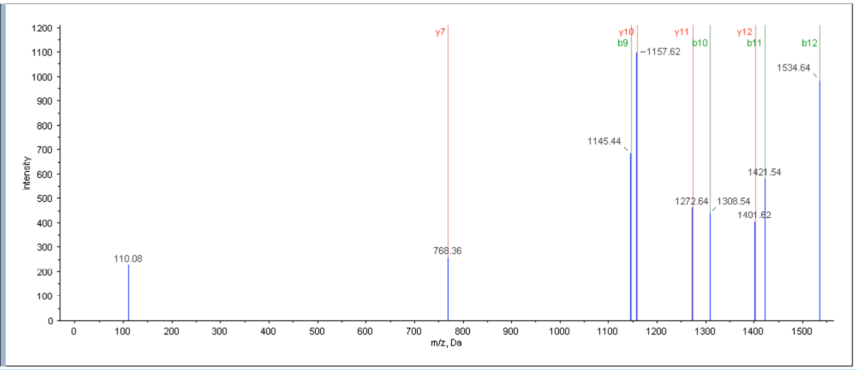

7	1739.857	

Fragmentation Evidence for Peptide

TTGPDVEEGALLPSPTR

Residue	b	y
T	102.0550	1739.8810
T	203.1026	1638.8333
G	260.1241	1537.7857
P	357.1769	1480.7642
D	472.2038	1383.7114
V	571.2722	1268.6845
E	700.3148	1169.6161
E	829.3574	1040.5735
G	886.3789	911.5309
A	957.4160	854.5094
L	1070.5000	783.4723
L	1183.5841	670.3883
P	1280.6369	557.3042
S	1367.6689	460.2514
P	1464.7217	373.2194
T	1565.7693	276.1666
R	1721.8705	175.1190

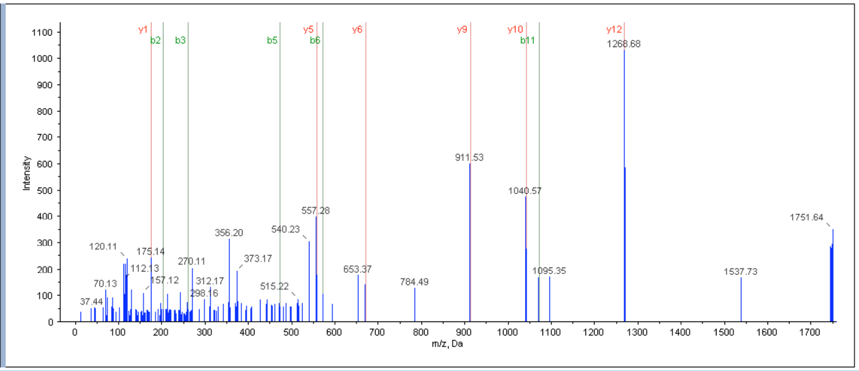

8	1417.698	

Fragmentation Evidence for Peptide

DPYRDNLVNRR

Residue	b	y
D	116.0342	1417.7295
P	213.0870	1302.7025
Y	376.1503	1205.6498
R	532.2514	1042.5865
D	647.2784	886.4853
N	761.3213	771.4584
L	874.4054	657.4155
V	973.4738	544.3314
N	1087.5167	445.2630
R	1243.6178	331.2201
R	1399.7189	175.1190

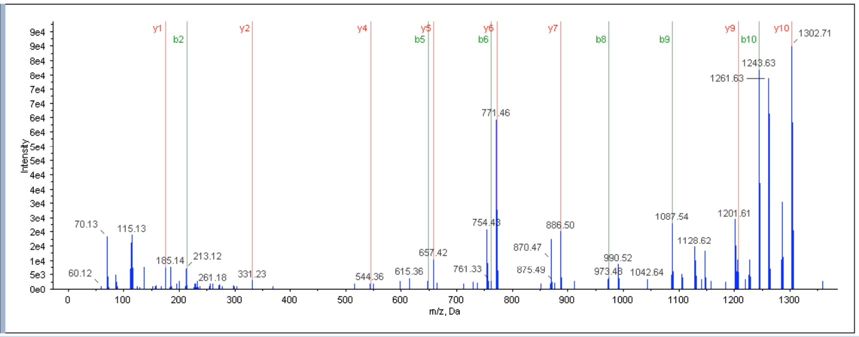

9	2723.576		

Fragmentation Evidence for Peptide

AAPAPAAAPKPAPPPPAAGAPKPPPPPPPK

Residue	b	y
A	72.0444	2723.5396
A	143.0815	2652.5024
P	**240.1343**	2581.4653
A	**311.1714**	2484.4126
P	408.2241	2413.3754
A	479.2613	2316.3227
A	550.2984	2245.2856
A	621.3355	2174.2485
P	718.3883	2103.2113
K	846.4832	2006.1586
P	943.5360	1878.0636
A	1014.5731	1781.0109
P	1111.6259	1709.9737
P	1208.6786	1612.9210
P	1305.7314	1515.8682
P	1402.7841	1418.8154
A	1473.8213	1321.7627
A	1544.8584	**1250.7256**
G	1601.8798	1179.6885
A	1672.9170	1122.6670
P	1769.9697	**1051.6299**
K	1898.0647	954.5771
P	**1995.1174**	826.4822
P	2092.1702	**729.4294**
P	2189.2230	**632.3766**
P	2286.2757	**535.3239**
P	2383.3285	**438.2711**
P	2480.3813	341.2183
P	2577.4340	244.1656
K	2705.5290	147.1128

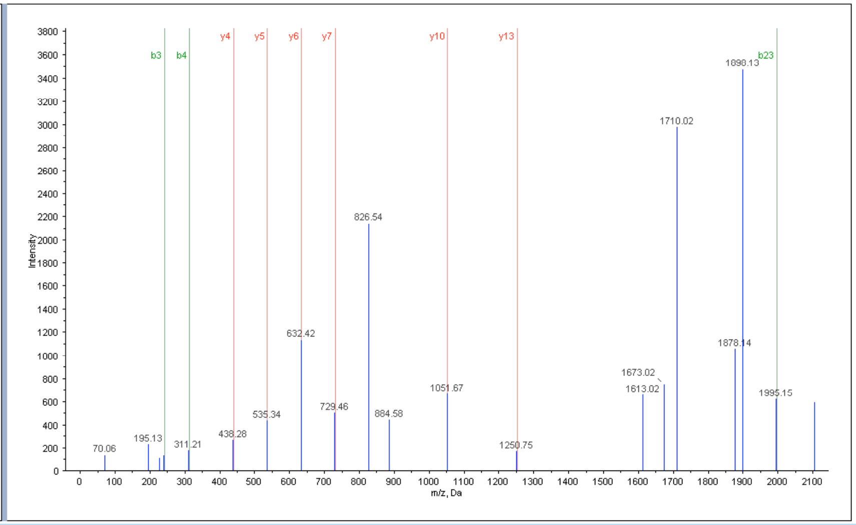

10			

Fragmentation Evidence for Peptide

MKEAQDEVQR

Residue	b	y
M	132.0478	1233.5892
K	260.1427	**1102.5487**
E	**389.1853**	974.4538
A	460.2224	845.4112
Q	588.2810	774.3741
D	703.3080	646.3155
E	832.3505	**531.2885**
V	931.4190	**402.2459**
Q	1059.4775	**303.1775**
R	1215.5786	**175.1190**

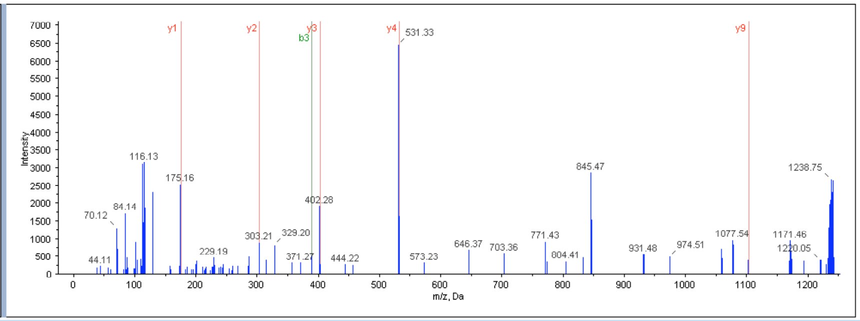

11	2862.181		Fragmentation Evidence for Peptide: YKLDKSENFDEYMKELGVGLVTRK
12	2068.002		Fragmentation Evidence for Peptide: GTDITEAFEAHHLTTAPEK

Fragmentation Evidence for Peptide

YKLDKSENFDEYMKELGVGLVTRK

Residue	b	y
Y	164.0706	2862.4706
K	292.1656	2699.4073
L	405.2496	2571.3123
D	520.2766	2458.2283
K	648.3715	**2343.2013**
S	735.4036	**2215.1063**
E	864.4462	2128.0743
N	978.4891	**1999.0317**
F	1125.5575	**1884.9888**
D	1240.5844	**1737.9204**
E	1369.6270	**1622.8934**
Y	1532.6904	**1493.8508**
M	1663.7309	**1330.7875**
K	1791.8258	**1199.7470**
E	1920.8684	**1071.6521**
L	2033.9525	**942.6095**
G	2090.9739	**829.5254**
V	2190.0423	**772.5039**
G	2247.0638	**673.4355**
L	2360.1479	616.4141
V	2459.2163	503.3300
T	2560.2640	404.2616
R	2716.3651	303.2139
K	2844.4600	147.1128

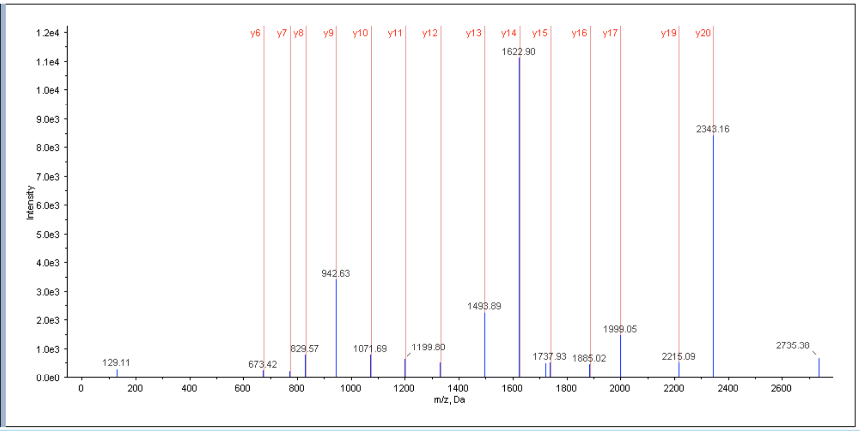

Fragmentation Evidence for Peptide

GTDITEAFEAHHLTTAPEK

Residue	b	y
G	58.0287	2067.9982
T	159.0764	2010.9767
D	274.1034	1909.9290
I	387.1874	1794.9021
T	488.2351	1681.8180
E	617.2777	1580.7703
A	**688.3148**	1451.7278
F	835.3832	1380.6906
E	964.4258	1233.6222
A	1035.4629	**1104.5796**
H	**1172.5218**	1033.5425
H	1309.5808	896.4836
L	1422.6648	759.4247
T	1523.7125	646.3406
T	1624.7602	545.2930
A	1695.7973	444.2453
P	1792.8501	373.2082
E	1921.8926	276.1554
K	2049.9876	147.1128

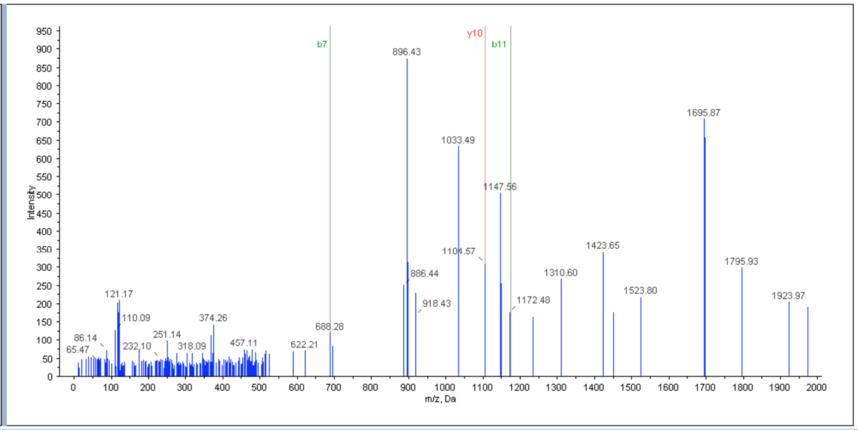

13	1459.699		Fragmentation Evidence for Peptide

Fragmentation Evidence for Peptide

TDSDPESNIVINR

Residue	b	y
T	102.0550	1459.7023
D	217.0819	1358.6546
S	304.1139	**1243.6277**
D	**419.1409**	1156.5957
P	516.1936	**1041.5687**
E	645.2362	944.5160
S	732.2683	**815.4734**
N	**846.3112**	**728.4413**
I	**959.3952**	**614.3984**
V	1058.4637	**501.3144**
I	1171.5477	**402.2459**
N	1285.5907	**289.1619**
R	1441.6918	**175.1190**

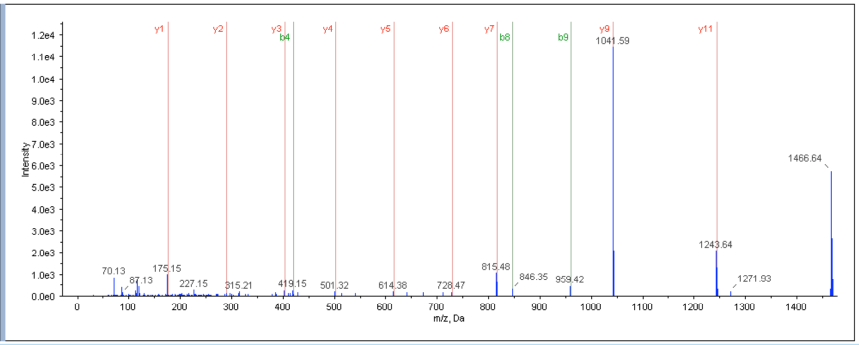

14	1955.079		Fragmentation Evidence for Peptide

Fragmentation Evidence for Peptide

NKQPQGSEEVKVPVFIR

Residue	b	y
N	115.0502	1955.0709
K	**243.1452**	1841.0280
Q	**371.2037**	1712.9330
P	468.2565	1584.8744
Q	596.3151	1487.8217
G	653.3365	1359.7631
S	740.3686	1302.7416
E	869.4112	1215.7096
E	998.4538	**1086.6670**
V	1097.5222	**957.6244**
K	1225.6171	**858.5560**
V	1324.6856	**730.4610**
P	1421.7383	**631.3926**
V	1520.8067	534.3398
F	1667.8751	**435.2714**
I	**1780.9592**	288.2030
R	1937.0603	**175.1190**

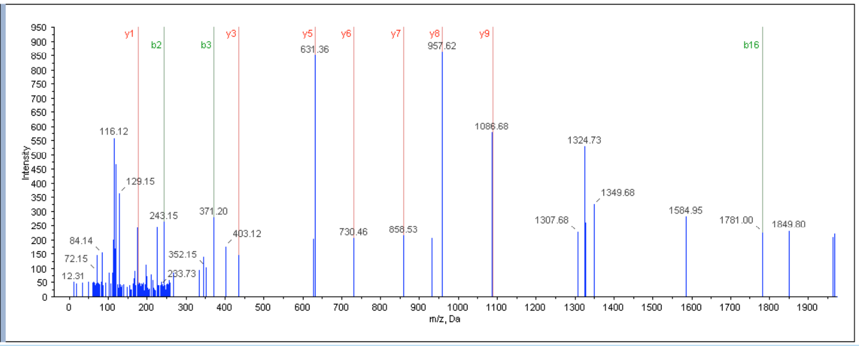

15	2051.011		Fragmentation Evidence for Peptide

Fragmentation Evidence for Peptide

NKQPQGSEEVKVPVFIR

Residue	b	y
N	115.0502	1955.0709
K	**243.1452**	1841.0280
Q	**371.2037**	1712.9330
P	468.2565	1584.8744
Q	596.3151	1487.8217
G	653.3365	1359.7631
S	740.3686	1302.7416
E	869.4112	1215.7096
E	998.4538	**1086.6670**
V	1097.5222	**957.6244**
K	1225.6171	**858.5560**
V	1324.6856	**730.4610**
P	1421.7383	**631.3926**
V	1520.8067	534.3398
F	1667.8751	**435.2714**
I	**1780.9592**	288.2030
R	1937.0603	**175.1190**

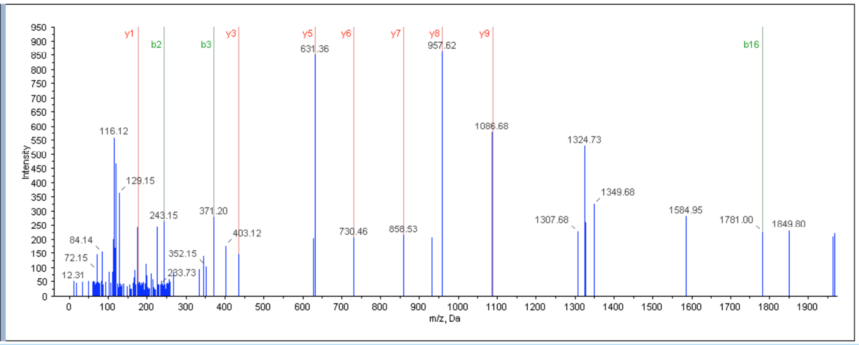

16	1239.632		Fragmentation Evidence for Peptide

LAEEFIPHQR

Residue	b	y
L	114.0913	1239.6480
A	**185.1285**	1126.5640
E	314.1710	1055.5269
E	443.2136	926.4843
F	**590.2821**	**797.4417**
I	703.3661	**650.3733**
P	800.4189	**537.2892**
H	937.4778	**440.2364**
Q	1065.5364	**303.1775**
R	1221.6375	**175.1190**

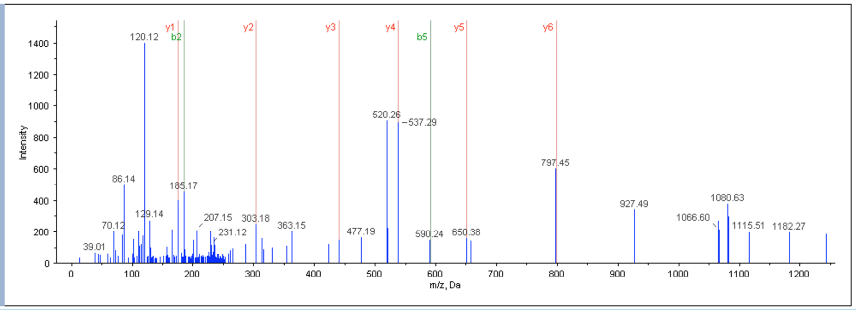

17	1559.762		Fragmentation Evidence for Peptide

GLQLTQPNTNNFGR

Residue	b	y
G	58.0287	1559.7925
L	**171.1128**	1502.7710
Q	**299.1714**	1389.6870
L	412.2554	1261.6284
T	513.3031	1148.5443
Q	641.3617	1047.4966
P	738.4145	919.4381
N	852.4574	822.3853
T	953.5051	708.3424
N	1067.5480	607.2947
N	1181.5909	493.2518
F	1328.6593	379.2088
G	1385.6808	**232.1404**
R	1541.7819	**175.1190**

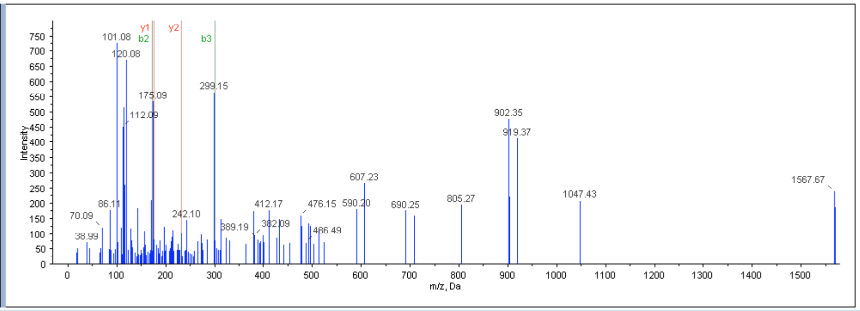

18	1411.747		Fragmentation Evidence for Peptide

THLADTVEELKR

Residue	b	y
T	102.0550	1411.7540
H	239.1139	1310.7063
L	**352.1979**	1173.6474
A	**423.2350**	1060.5633
D	**538.2620**	**989.5262**
T	639.3097	874.4993
V	738.3781	773.4516
E	867.4207	674.3832
E	996.4633	**545.3406**
L	1109.5473	**416.2980**
K	1237.6423	**303.2139**
R	1393.7434	**175.1190**

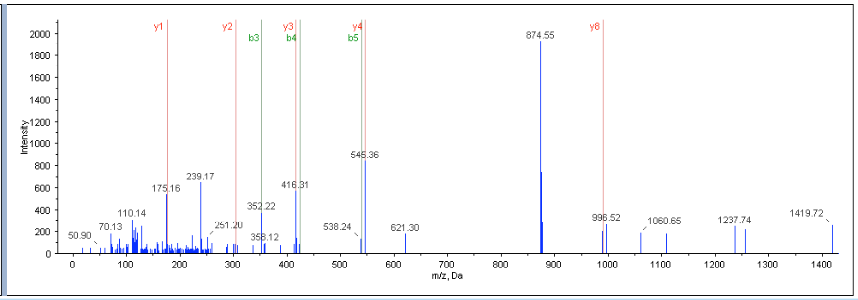

<table>
<tr><td>19</td><td>1899.845</td><td></td><td>
Fragmentation Evidence for Peptide

FC[CAM]QLDTGDSDAC[CAM]EILR

<table>
<tr><th>Residue</th><th>b</th><th>y</th></tr>
<tr><td>F</td><td>148.0757</td><td>1899.8211</td></tr>
<tr><td>C[CAM]</td><td>308.1063</td><td>1752.7527</td></tr>
<tr><td>Q</td><td>436.1649</td><td>1592.7221</td></tr>
<tr><td>L</td><td>549.2490</td><td>1464.6635</td></tr>
<tr><td>D</td><td>664.2759</td><td>1351.5794</td></tr>
<tr><td>T</td><td>765.3236</td><td>1236.5525</td></tr>
<tr><td>G</td><td>822.3451</td><td>1135.5048</td></tr>
<tr><td>D</td><td>937.3720</td><td>1078.4833</td></tr>
<tr><td>S</td><td>1024.4040</td><td>963.4564</td></tr>
<tr><td>D</td><td>1139.4310</td><td>876.4244</td></tr>
<tr><td>A</td><td>1210.4681</td><td>761.3974</td></tr>
<tr><td>C[CAM]</td><td>1370.4987</td><td>690.3603</td></tr>
<tr><td>E</td><td>1499.5413</td><td>530.3297</td></tr>
<tr><td>I</td><td>1612.6254</td><td>401.2871</td></tr>
<tr><td>L</td><td>1725.7095</td><td>288.2030</td></tr>
<tr><td>R</td><td>1881.8106</td><td>175.1190</td></tr>
</table>

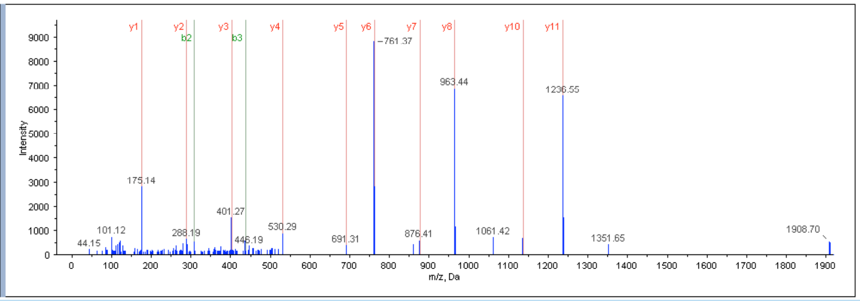

</td></tr>
<tr><td>20</td><td>1094.563</td><td></td><td>
Fragmentation Evidence for Peptide

MDRVGEFLK

<table>
<tr><th>Residue</th><th>b</th><th>y</th></tr>
<tr><td>M</td><td>132.0478</td><td>1094.5663</td></tr>
<tr><td>D</td><td>247.0747</td><td>963.5258</td></tr>
<tr><td>R</td><td>403.1758</td><td>848.4989</td></tr>
<tr><td>V</td><td>502.2442</td><td>692.3978</td></tr>
<tr><td>G</td><td>559.2657</td><td>593.3293</td></tr>
<tr><td>E</td><td>688.3083</td><td>536.3079</td></tr>
<tr><td>F</td><td>835.3767</td><td>407.2653</td></tr>
<tr><td>L</td><td>948.4608</td><td>260.1969</td></tr>
<tr><td>K</td><td>1076.5557</td><td>147.1128</td></tr>
</table>

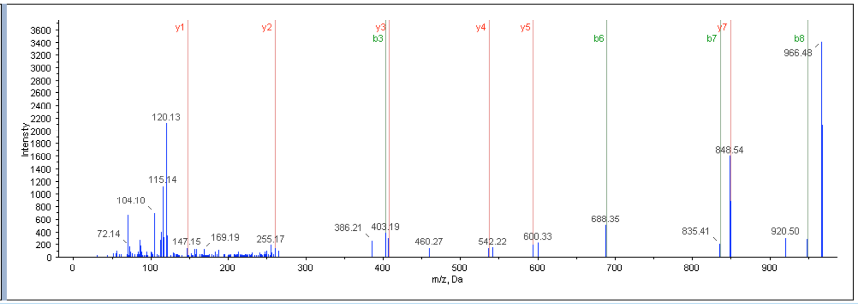

</td></tr>
<tr><td>21</td><td>1431.774</td><td></td><td>
Fragmentation Evidence for Peptide

NLTGENDGRSQIK

<table>
<tr><th>Residue</th><th>b</th><th>y</th></tr>
<tr><td>N</td><td>115.0502</td><td>1431.7186</td></tr>
<tr><td>L</td><td>228.1343</td><td>1317.6757</td></tr>
<tr><td>T</td><td>329.1819</td><td>1204.5917</td></tr>
<tr><td>G</td><td>386.2034</td><td>1103.5440</td></tr>
<tr><td>E</td><td>515.2460</td><td>1046.5225</td></tr>
<tr><td>N</td><td>629.2889</td><td>917.4799</td></tr>
<tr><td>D</td><td>744.3159</td><td>803.4370</td></tr>
<tr><td>G</td><td>801.3373</td><td>688.4100</td></tr>
<tr><td>R</td><td>957.4384</td><td>631.3886</td></tr>
<tr><td>S</td><td>1044.4705</td><td>475.2875</td></tr>
<tr><td>Q</td><td>1172.5291</td><td>388.2554</td></tr>
<tr><td>I</td><td>1285.6131</td><td>260.1969</td></tr>
<tr><td>K</td><td>1413.7081</td><td>147.1128</td></tr>
</table>

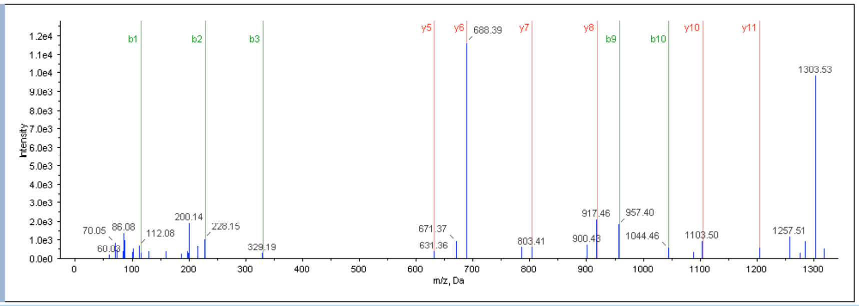

</td></tr>
</table>

22	2011.016		Fragmentation Evidence for Peptide

DASHVKFTPWAN[Dea]GKPAQR

Residue	b	y
D	116.0342	2011.0144
A	187.0713	1895.9875
S	274.1034	1824.9504
H	**411.1623**	1737.9183
V	**510.2307**	1600.8594
K	638.3257	1501.7910
F	785.3941	**1373.6961**
T	886.4417	1226.6276
P	983.4945	**1125.5800**
W	1169.5738	1028.5272
A	1240.6109	**842.4479**
N[Dea]	1355.6379	**771.4108**
G	1412.6593	**656.3838**
K	**1540.7543**	599.3624
P	1637.8071	**471.2674**
A	1708.8442	374.2146
Q	**1836.9028**	**303.1775**
R	1993.0039	**175.1190**

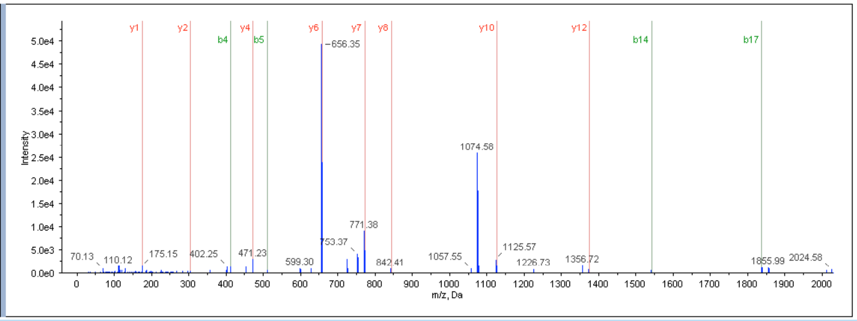

23	861.4528		Fragmentation Evidence for Peptide

AIESQWK

Residue	b	y
A	72.0444	861.4465
I	**185.1285**	790.4094
E	**314.1710**	**677.3253**
S	**401.2031**	**548.2827**
Q	**529.2617**	**461.2507**
W	**715.3410**	**333.1921**
K	843.4359	**147.1128**

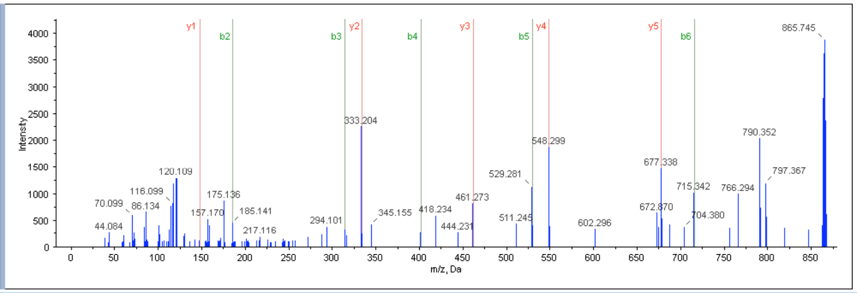

24	1712.843		Fragmentation Evidence for Peptide

AIESQWK

Residue	b	y
A	72.0444	861.4465
I	**185.1285**	790.4094
E	**314.1710**	**677.3253**
S	**401.2031**	**548.2827**
Q	**529.2617**	**461.2507**
W	**715.3410**	**333.1921**
K	843.4359	**147.1128**

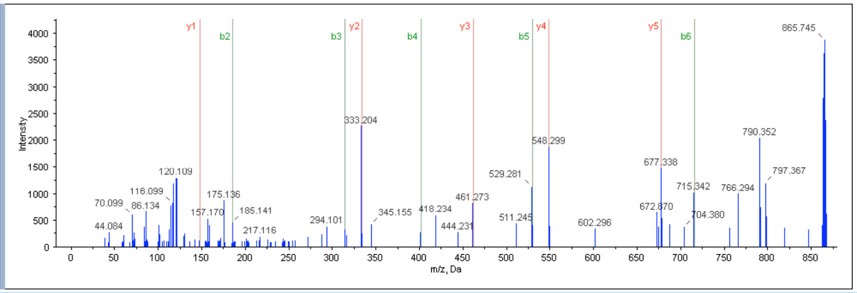

25	1366.664	

Fragmentation Evidence for Peptide

AQDNPWPQWPK

Residue	b	y
A	72.0444	1366.6539
Q	**200.1030**	1295.6167
D	**315.1299**	1167.5582
N	**429.1728**	1052.5312
P	526.2256	**938.4883**
W	**712.3049**	841.4355
P	**809.3577**	**655.3562**
Q	**937.4163**	558.3035
W	**1123.4956**	**430.2449**
P	1220.5483	**244.1656**
K	1348.6433	147.1128

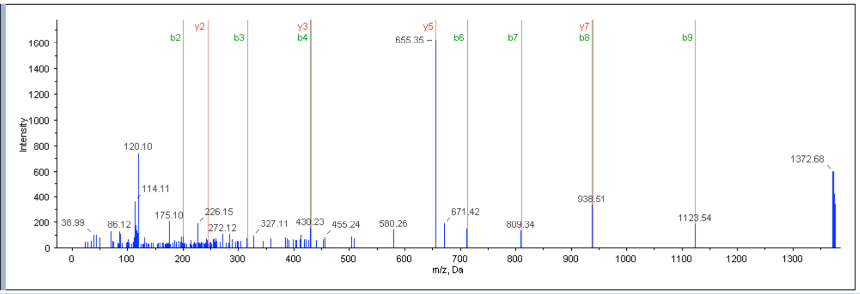

26	1294.681	

Fragmentation Evidence for Peptide

SIFSNFPQQVK

Residue	b	y
S	88.0393	1294.6790
I	**201.1234**	1207.6470
F	348.1918	1094.5629
S	435.2238	947.4945
N	549.2667	**860.4625**
F	696.3352	746.4196
P	793.3879	**599.3511**
Q	921.4465	502.2984
Q	1049.5051	**374.2398**
V	1148.5735	246.1812
K	1276.6684	**147.1128**

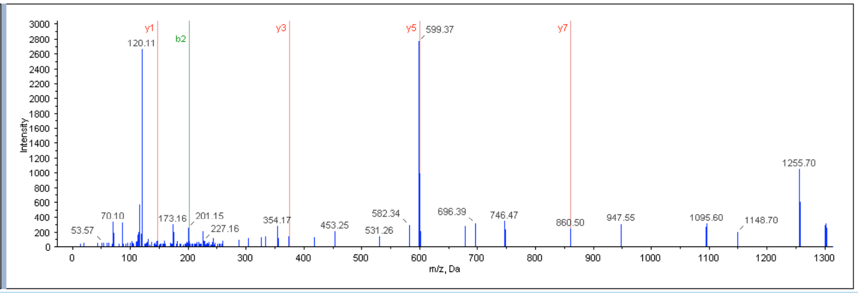

27	1786.863	

Fragmentation Evidence for Peptide

VSHDTTVVYELGNKPK

Residue	b	y
V	100.0757	1786.9334
S	187.1077	1687.8650
H	324.1666	1600.8329
D	439.1936	1463.7740
T	540.2413	**1348.7471**
T	**641.2889**	1247.6994
V	**740.3573**	1146.6517
V	**839.4258**	**1047.5833**
Y	**1002.4891**	**948.5149**
E	**1131.5317**	**785.4516**
L	**1244.6157**	**656.4090**
G	**1301.6372**	**543.3249**
N	**1415.6801**	486.3035
K	**1543.7751**	372.2605
P	1640.8279	244.1656
K	1768.9228	147.1128

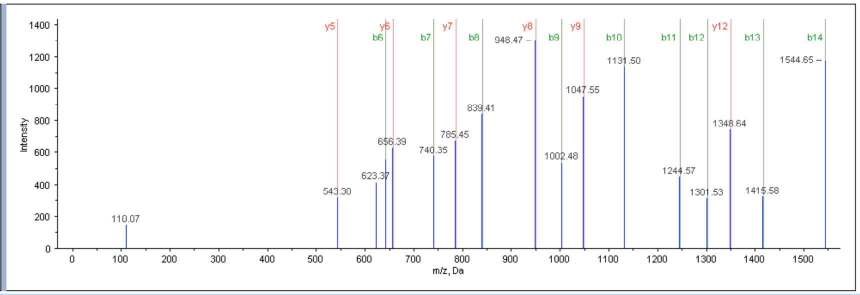

28	1414.761		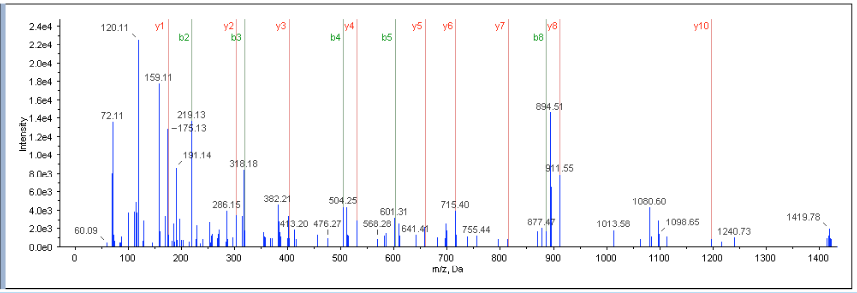
29	1525.715		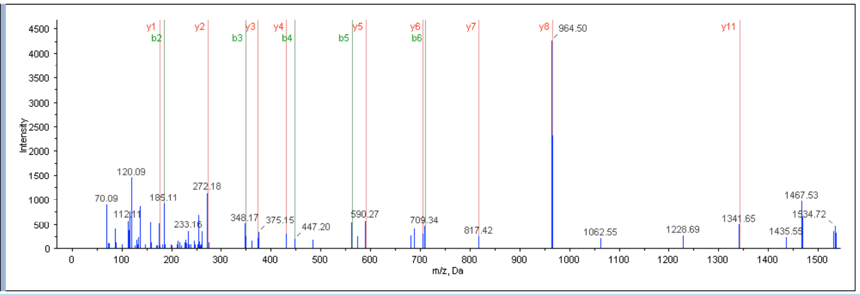
30	1950.982		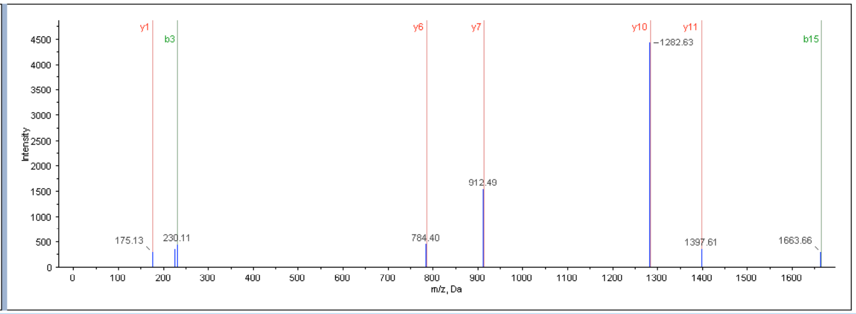

Row 28 — Fragmentation Evidence for Peptide

AFVWPVGQQVQR

Residue	b	y
A	72.0444	1414.7590
F	**219.1128**	1343.7219
V	**318.1812**	**1196.6535**
W	**504.2605**	1097.5851
P	**601.3133**	**911.5057**
V	700.3817	**814.4530**
G	757.4032	**715.3846**
Q	**885.4618**	**658.3631**
Q	1013.5203	**530.3045**
V	1112.5887	**402.2459**
Q	1240.6473	**303.1775**
R	1396.7484	**175.1190**

Row 29 — Fragmentation Evidence for Peptide

SPYVDFLNC[CAM]GTPR

Residue	b	y
S	88.0393	1525.7104
P	**185.0921**	1438.6784
Y	**348.1554**	**1341.6256**
V	**447.2238**	1178.5623
D	**562.2508**	1079.4939
F	**709.3192**	**964.4669**
L	822.4032	**817.3985**
N	936.4462	**704.3144**
C[CAM]	1096.4768	**590.2715**
G	1153.4983	**430.2409**
T	1254.5459	**373.2194**
P	1351.5987	**272.1717**
R	1507.6998	**175.1190**

Row 30 — Fragmentation Evidence for Peptide

SAAVPKDQIEKGYFEIR

Residue	b	y
S	88.0393	1951.0284
A	159.0764	1863.9963
A	**230.1135**	1792.9592
V	329.1819	1721.9221
P	426.2347	1622.8537
K	554.3297	1525.8009
D	669.3566	**1397.7060**
Q	797.4152	**1282.6790**
I	910.4993	1154.6204
E	1039.5419	1041.5364
K	1167.6368	**912.4938**
G	1224.6583	**784.3988**
Y	1387.7216	727.3774
F	1534.7900	564.3140
E	**1663.8326**	417.2456
I	1776.9167	288.2030
R	1933.0178	**175.1190**

31	2363.161		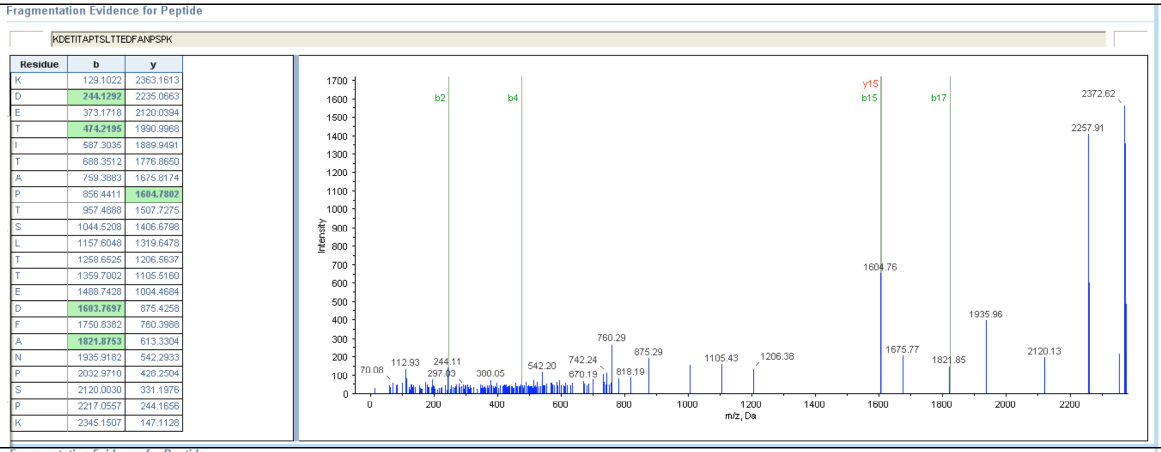

Residue	b	y
K	129.1022	2363.1613
D	**244.1292**	2235.0663
E	373.1718	2120.0394
T	**474.2195**	1990.9968
I	587.3035	1889.9491
T	688.3512	1776.8650
A	759.3883	1675.8174
P	856.4411	**1604.7802**
T	957.4888	1507.7275
S	1044.5208	1406.6798
L	1157.6048	1319.6478
T	1258.6525	1206.5637
T	1359.7002	1105.5160
E	1488.7428	1004.4684
D	**1603.7697**	875.4258
F	1750.8382	760.3988
A	**1821.8753**	613.3304
N	1935.9182	542.2933
P	2032.9710	428.2504
S	2120.0030	331.1976
P	2217.0557	244.1656
K	2345.1507	147.1128

32	1268.641		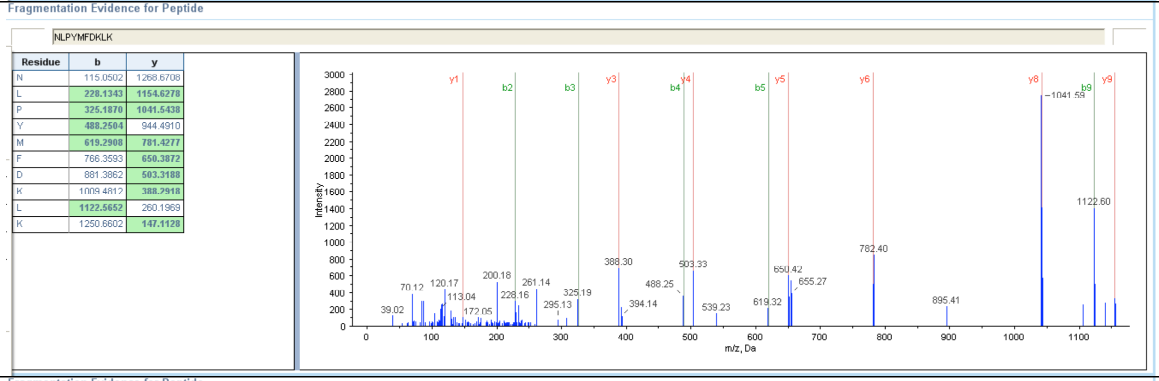

Residue	b	y
N	115.0502	1268.6708
L	**228.1343**	**1154.6278**
P	**325.1870**	**1041.5438**
Y	**488.2504**	944.4910
M	**619.2908**	**781.4277**
F	766.3593	**650.3872**
D	881.3862	**503.3188**
K	1009.4812	**388.2918**
L	**1122.5652**	260.1969
K	1250.6602	**147.1128**

33	1250.673		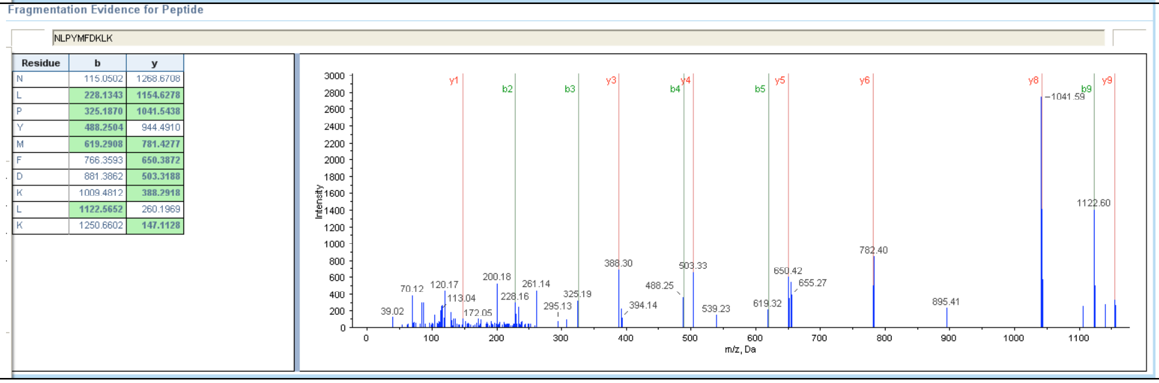

Residue	b	y
N	115.0502	1268.6708
L	**228.1343**	**1154.6278**
P	**325.1870**	**1041.5438**
Y	**488.2504**	944.4910
M	**619.2908**	**781.4277**
F	766.3593	**650.3872**
D	881.3862	**503.3188**
K	1009.4812	**388.2918**
L	**1122.5652**	260.1969
K	1250.6602	**147.1128**

<table>
<tr><td>34</td><td>1488.689</td><td></td><td>Fragmentation Evidence for Peptide
YVHQFFSQNYR
<table><tr><th>Residue</th><th>b</th><th>y</th></tr><tr><td>Y</td><td>164.0706</td><td>1488.7019</td></tr><tr><td>V</td><td>263.1390</td><td>1325.6385</td></tr><tr><td>H</td><td>400.1979</td><td>1226.5701</td></tr><tr><td>Q</td><td>528.2565</td><td>1089.5112</td></tr><tr><td>F</td><td>675.3249</td><td>961.4526</td></tr><tr><td>F</td><td>822.3933</td><td>814.3842</td></tr><tr><td>S</td><td>909.4254</td><td>667.3158</td></tr><tr><td>Q</td><td>1037.4839</td><td>580.2838</td></tr><tr><td>N</td><td>1151.5269</td><td>452.2252</td></tr><tr><td>Y</td><td>1314.5902</td><td>338.1823</td></tr><tr><td>R</td><td>1470.6913</td><td>175.1190</td></tr></table>
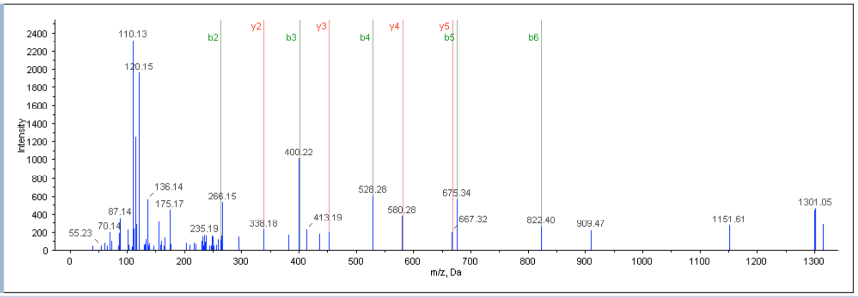
</td></tr>
<tr><td>35</td><td>1497.713</td><td></td><td>Fragmentation Evidence for Peptide
NAALEYYNQFHK
<table><tr><th>Residue</th><th>b</th><th>y</th></tr><tr><td>N</td><td>115.0502</td><td>1497.7121</td></tr><tr><td>A</td><td>186.0873</td><td>1383.6692</td></tr><tr><td>A</td><td>257.1244</td><td>1312.6321</td></tr><tr><td>L</td><td>370.2085</td><td>1241.5949</td></tr><tr><td>E</td><td>499.2511</td><td>1128.5109</td></tr><tr><td>Y</td><td>662.3144</td><td>999.4683</td></tr><tr><td>Y</td><td>825.3777</td><td>836.4050</td></tr><tr><td>N</td><td>939.4207</td><td>673.3416</td></tr><tr><td>Q</td><td>1067.4793</td><td>559.2987</td></tr><tr><td>F</td><td>1214.5477</td><td>431.2401</td></tr><tr><td>H</td><td>1351.6066</td><td>284.1717</td></tr><tr><td>K</td><td>1479.7015</td><td>147.1128</td></tr></table>
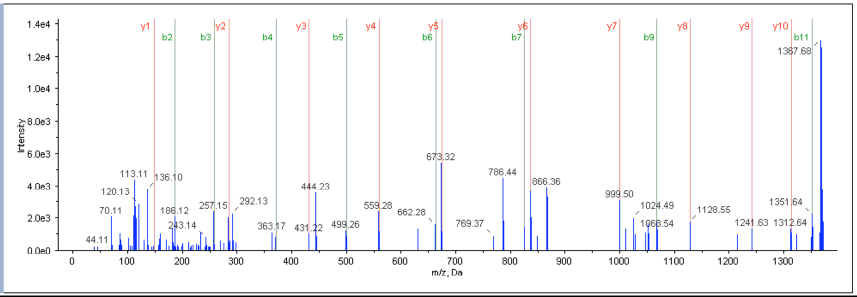
</td></tr>
<tr><td>36</td><td>920.3999</td><td></td><td>Fragmentation Evidence for Peptide
SQC[CAM]EHPY
<table><tr><th>Residue</th><th>b</th><th>y</th></tr><tr><td>S</td><td>88.0393</td><td>920.3567</td></tr><tr><td>Q</td><td>216.0979</td><td>833.3247</td></tr><tr><td>C[CAM]</td><td>376.1285</td><td>705.2661</td></tr><tr><td>E</td><td>505.1711</td><td>545.2354</td></tr><tr><td>H</td><td>642.2300</td><td>416.1928</td></tr><tr><td>P</td><td>739.2828</td><td>279.1339</td></tr><tr><td>Y</td><td>902.3461</td><td>182.0812</td></tr></table>
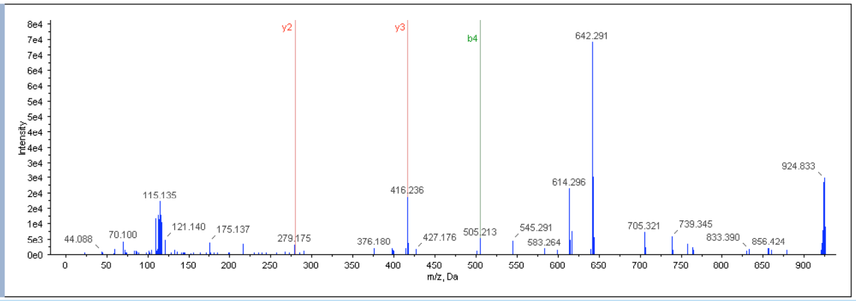
</td></tr>
</table>

37	1714.771		Fragmentation Evidence for Peptide

ASDESQPC[CAM]GVQYFVK

Residue	b	y
A	72.0444	1714.7741
S	159.0764	1643.7370
D	274.1034	1556.7050
E	403.1460	1441.6780
S	490.1780	1312.6354
Q	618.2366	1225.6034
P	715.2893	1097.5448
C[CAM]	875.3200	1000.4921
G	932.3414	840.4614
V	1031.4099	783.4400
Q	1159.4684	684.3715
Y	1322.5318	556.3130
F	1469.6002	393.2496
V	1568.6686	246.1812
K	1696.7635	147.1128

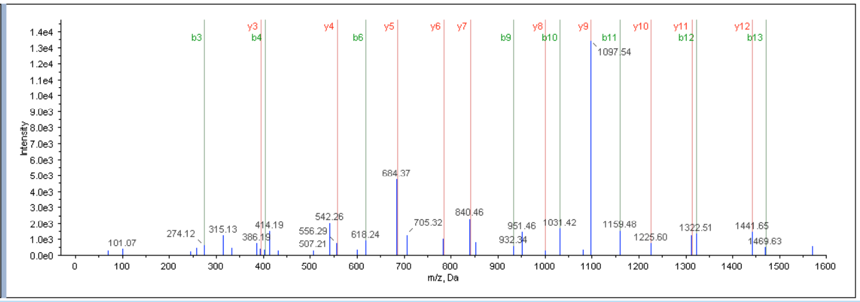

38	1316.67		Fragmentation Evidence for Peptide

LQLWDTAGQER

Residue	b	y
L	114.0913	1316.6593
Q	242.1499	1203.5753
L	355.2340	1075.5167
W	541.3133	962.4326
D	656.3402	776.3533
T	757.3879	661.3264
A	828.4250	560.2787
G	885.4465	489.2416
Q	1013.5051	432.2201
E	1142.5477	304.1615
R	1298.6488	175.1190

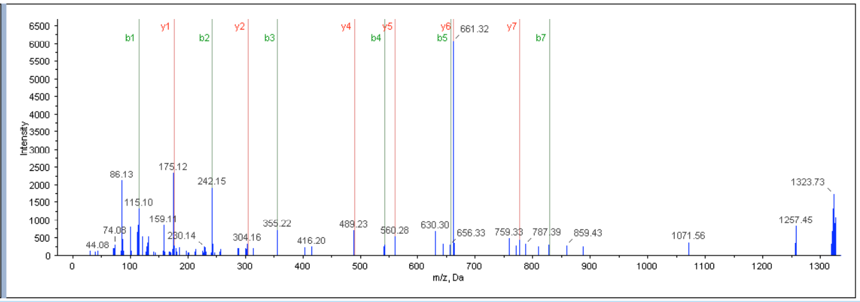

39	993.505		Fragmentation Evidence for Peptide

DAFDEIKR

Residue	b	y
D	116.0342	993.5000
A	187.0713	878.4730
F	334.1397	807.4359
D	449.1667	660.3675
E	578.2093	545.3406
I	691.2933	416.2980
K	819.3883	303.2139
R	975.4894	175.1190

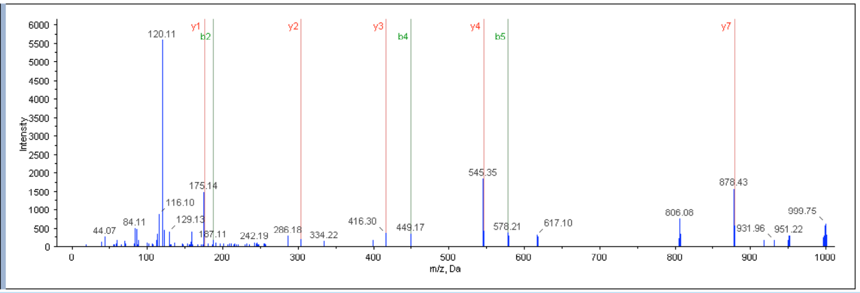

40	1649.851		

Fragmentation Evidence for Peptide

VIIFGVPGAFTPGC[CAM]SK

Residue	b	y
V	100.0757	1649.8720
I	**213.1598**	1550.8036
I	**326.2438**	1437.7195
F	473.3122	1324.6354
G	**530.3337**	**1177.5670**
V	**629.4021**	**1120.5456**
P	726.4549	**1021.4771**
G	783.4763	924.4244
A	854.5135	**867.4029**
F	1001.5819	**796.3658**
T	1102.6295	**649.2974**
P	1199.6823	**548.2497**
G	1256.7038	451.1969
C[CAM]	1416.7344	394.1755
S	1503.7664	234.1448
K	1631.8614	147.1128

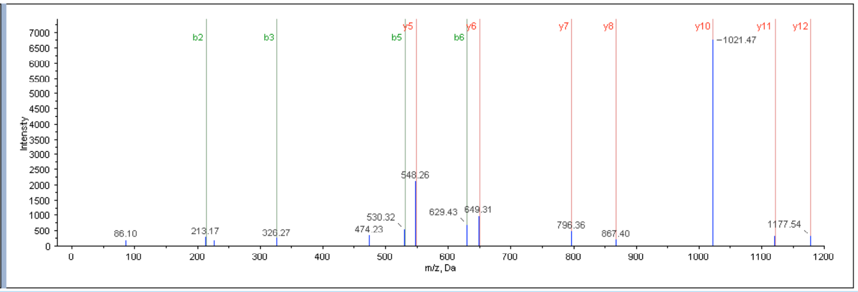

41	1497.726		

Fragmentation Evidence for Peptide

KMPEYDVIEFAR

Residue	b	y
K	**129.1022**	1497.7406
M	**260.1427**	1369.6457
P	357.1955	**1238.6052**
E	486.2381	1141.5524
Y	649.3014	**1012.5098**
D	**764.3284**	**849.4465**
V	863.3968	**734.4196**
I	976.4808	**635.3511**
E	1105.5234	**522.2671**
F	1252.5918	**393.2245**
A	1323.6290	**246.1561**
R	1479.7301	**175.1190**

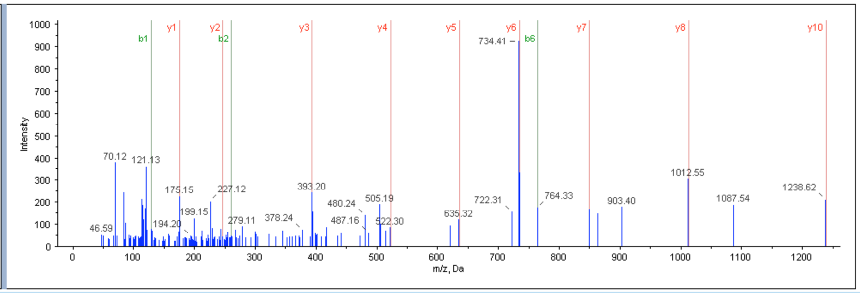

42	2127.085		

Fragmentation Evidence for Peptide

SSQQQEQPQQSQSQNVPAK

Residue	b	y
S	88.0393	2127.0061
S	175.0713	2039.9741
Q	303.1299	1952.9421
Q	431.1885	1824.8835
Q	559.2471	1696.8249
E	688.2897	**1568.7663**
Q	**816.3482**	1439.7237
P	913.4010	**1311.6652**
Q	**1041.4596**	1214.6124
Q	**1169.5182**	1086.5538
S	1256.5502	**958.4952**
Q	1384.6088	**871.4632**
S	1471.6408	**743.4046**
Q	1599.6994	**656.3726**
N	1713.7423	528.3140
V	**1812.8107**	414.2711
P	1909.8635	**315.2027**
A	1980.9006	218.1499
K	2108.9955	147.1128

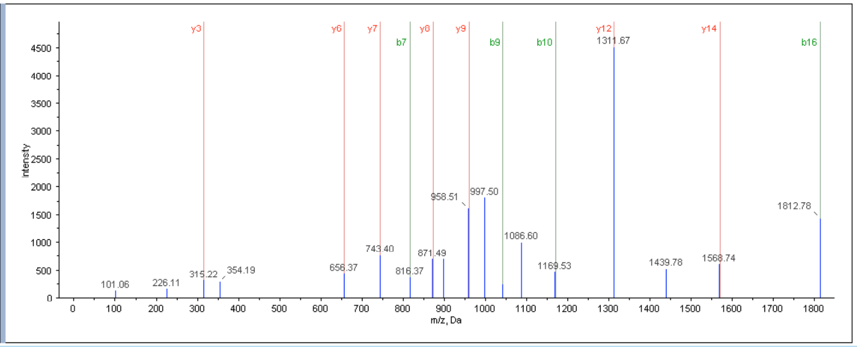

43	2122.015		Fragmentation Evidence for Peptide

Fragmentation Evidence for Peptide

KADVLATEPEDMYIAYHR

Residue	b	y
K	**129.1022**	2122.0274
A	200.1394	1993.9324
D	**315.1663**	**1922.8953**
V	414.2347	1807.8683
L	527.3188	1708.7999
A	**598.3559**	1595.7159
T	699.4036	1524.6788
E	828.4462	1423.6311
P	925.4989	**1294.5885**
E	1054.5415	1197.5357
D	1169.5685	1068.4931
M	1300.6089	953.4662
Y	1463.6723	822.4257
I	1576.7563	659.3624
A	1647.7935	546.2783
Y	1810.8568	475.2412
H	**1947.9157**	**312.1779**
R	2104.0168	**175.1190**

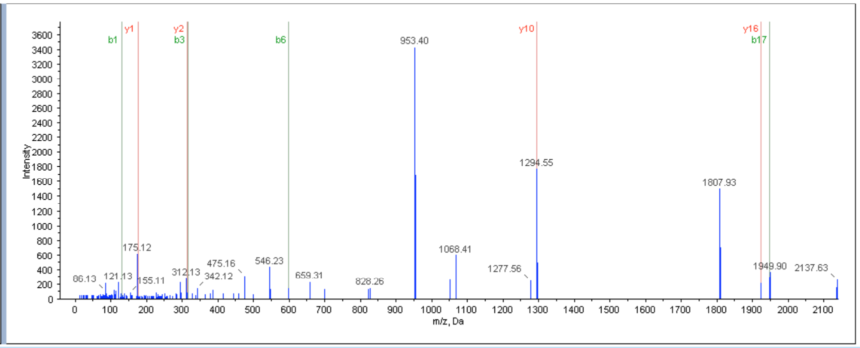

44	2175.976		Fragmentation Evidence for Peptide

Fragmentation Evidence for Peptide

ANHYVNGGDFGNREDQINR

Residue	b	y
A	72.0444	2175.9915
N	186.0873	2104.9544
H	**323.1462**	1990.9114
Y	486.2096	1853.8525
V	**585.2780**	1690.7892
N	**699.3209**	1591.7208
G	**756.3424**	**1477.6778**
G	813.3638	1420.6564
D	**928.3908**	**1363.6349**
F	1075.4592	**1248.6080**
G	1132.4806	1101.5396
N	1246.5236	1044.5181
R	1402.6247	930.4752
E	**1531.6673**	774.3741
D	**1646.6942**	645.3315
Q	1774.7528	**530.3045**
I	1887.8369	402.2459
N	**2001.8798**	289.1619
R	2157.9809	175.1190

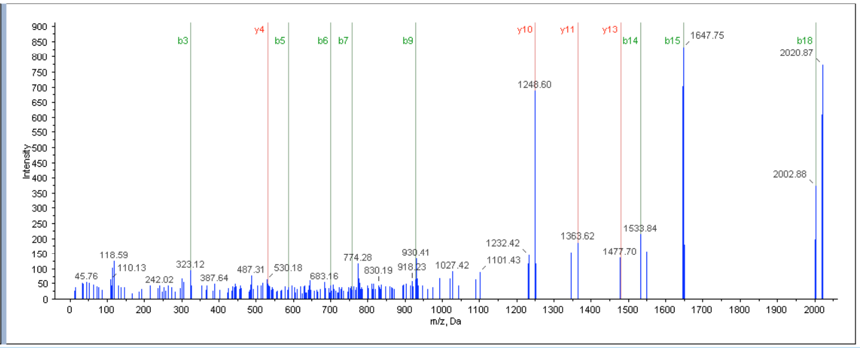

45	1698.86		Fragmentation Evidence for Peptide

Fragmentation Evidence for Peptide

ANHYVNGGDFGNREDQINR

Residue	b	y
A	72.0444	2175.9915
N	186.0873	2104.9544
H	**323.1462**	1990.9114
Y	486.2096	1853.8525
V	**585.2780**	1690.7892
N	**699.3209**	1591.7208
G	**756.3424**	**1477.6778**
G	813.3638	1420.6564
D	**928.3908**	**1363.6349**
F	1075.4592	**1248.6080**
G	1132.4806	1101.5396
N	1246.5236	1044.5181
R	1402.6247	930.4752
E	**1531.6673**	774.3741
D	**1646.6942**	645.3315
Q	1774.7528	**530.3045**
I	1887.8369	402.2459
N	**2001.8798**	289.1619
R	2157.9809	175.1190

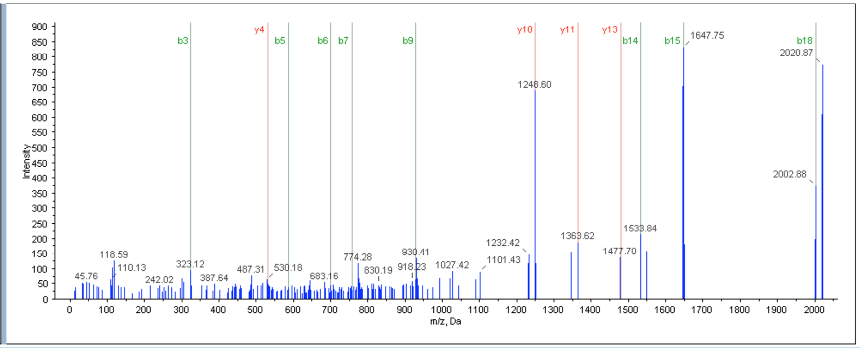

<table>
<tr><td>46</td><td>1095.464</td><td>
Fragmentation Evidence for Peptide
KFC[CAM]VGGNWK
<table>
<tr><th>Residue</th><th>b</th><th>y</th></tr>
<tr><td>K</td><td>129.1022</td><td>1095.5404</td></tr>
<tr><td>F</td><td>276.1707</td><td>967.4454</td></tr>
<tr><td>C[CAM]</td><td>436.2013</td><td>820.3770</td></tr>
<tr><td>V</td><td>535.2697</td><td>660.3464</td></tr>
<tr><td>G</td><td>592.2912</td><td>561.2780</td></tr>
<tr><td>G</td><td>649.3126</td><td>504.2565</td></tr>
<tr><td>N</td><td>763.3556</td><td>447.2350</td></tr>
<tr><td>W</td><td>949.4349</td><td>333.1921</td></tr>
<tr><td>K</td><td>1077.5298</td><td>147.1128</td></tr>
</table>
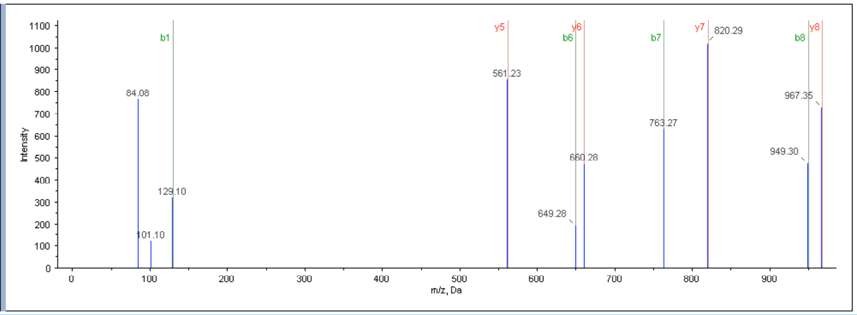

</td></tr>
<tr><td>47</td><td>2096.999</td><td>
Fragmentation Evidence for Peptide
ESHTDTPESSPKPTSPFPR
<table>
<tr><th>Residue</th><th>b</th><th>y</th></tr>
<tr><td>E</td><td>130.0499</td><td>2096.9883</td></tr>
<tr><td>S</td><td>217.0819</td><td>1967.9457</td></tr>
<tr><td>H</td><td>354.1408</td><td>1880.9137</td></tr>
<tr><td>T</td><td>455.1885</td><td>1743.8548</td></tr>
<tr><td>D</td><td>570.2154</td><td>1642.8071</td></tr>
<tr><td>T</td><td>671.2631</td><td>1527.7802</td></tr>
<tr><td>P</td><td>768.3159</td><td>1426.7325</td></tr>
<tr><td>E</td><td>897.3585</td><td>1329.6797</td></tr>
<tr><td>S</td><td>984.3905</td><td>1200.6371</td></tr>
<tr><td>S</td><td>1071.4225</td><td>1113.6051</td></tr>
<tr><td>P</td><td>1168.4753</td><td>1026.5731</td></tr>
<tr><td>K</td><td>1296.5703</td><td>929.5203</td></tr>
<tr><td>P</td><td>1393.6230</td><td>801.4254</td></tr>
<tr><td>T</td><td>1494.6707</td><td>704.3726</td></tr>
<tr><td>S</td><td>1581.7027</td><td>603.3249</td></tr>
<tr><td>P</td><td>1678.7555</td><td>516.2929</td></tr>
<tr><td>F</td><td>1825.8239</td><td>419.2401</td></tr>
<tr><td>P</td><td>1922.8767</td><td>272.1717</td></tr>
<tr><td>R</td><td>2078.9778</td><td>175.1190</td></tr>
</table>
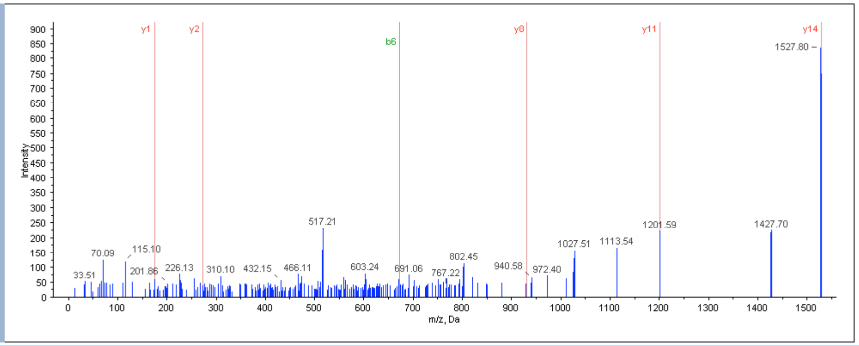

</td></tr>
<tr><td>48</td><td>2123.135</td><td>
Fragmentation Evidence for Peptide
IGNSPYNNKEFQTNKELK
<table>
<tr><th>Residue</th><th>b</th><th>y</th></tr>
<tr><td>I</td><td>114.0913</td><td>2124.0720</td></tr>
<tr><td>G</td><td>171.1128</td><td>2010.9879</td></tr>
<tr><td>N</td><td>285.1557</td><td>1953.9665</td></tr>
<tr><td>S</td><td>372.1878</td><td>1839.9236</td></tr>
<tr><td>P</td><td>469.2405</td><td>1752.8915</td></tr>
<tr><td>Y</td><td>632.3039</td><td>1655.8388</td></tr>
<tr><td>N</td><td>746.3468</td><td>1492.7754</td></tr>
<tr><td>N</td><td>860.3897</td><td>1378.7325</td></tr>
<tr><td>K</td><td>988.4847</td><td>1264.6896</td></tr>
<tr><td>E</td><td>1117.5273</td><td>1136.5946</td></tr>
<tr><td>F</td><td>1264.5957</td><td>1007.5520</td></tr>
<tr><td>Q</td><td>1392.6543</td><td>860.4836</td></tr>
<tr><td>T</td><td>1493.7019</td><td>732.4250</td></tr>
<tr><td>N</td><td>1607.7449</td><td>631.3774</td></tr>
<tr><td>K</td><td>1735.8398</td><td>517.3344</td></tr>
<tr><td>E</td><td>1864.8824</td><td>389.2395</td></tr>
<tr><td>L</td><td>1977.9665</td><td>260.1969</td></tr>
<tr><td>K</td><td>2106.0614</td><td>147.1128</td></tr>
</table>
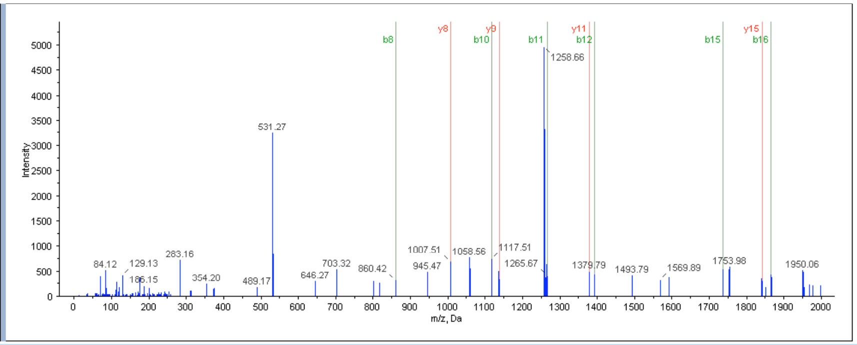

</td></tr>
</table>

49	1830.987		Fragmentation Evidence for Peptide (see below)

Fragmentation Evidence for Peptide

ITALDPTKIYGKPEER

Residue	b	y
I	114.0913	1830.9960
T	215.1390	1717.9119
A	286.1761	1616.8642
L	399.2602	1545.8271
D	514.2871	1432.7431
P	611.3399	1317.7161
T	712.3876	1220.6634
K	840.4825	1119.6157
I	953.5666	991.5207
Y	1116.6299	878.4367
G	1173.6514	715.3733
K	1301.7464	658.3519
P	1398.7991	530.2569
E	1527.8417	433.2041
E	1656.8843	304.1615
R	1812.9854	175.1190

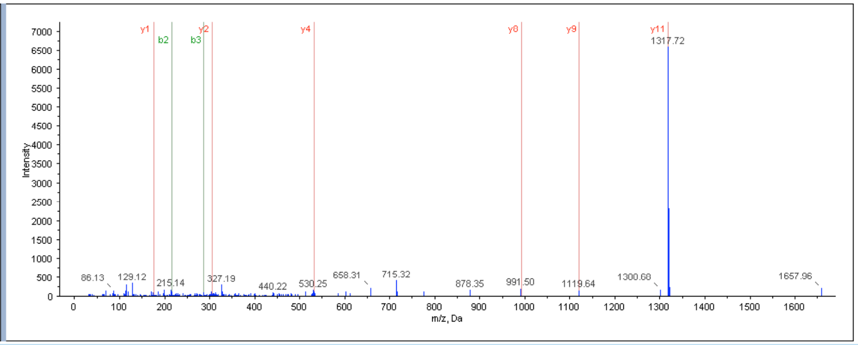

50	2215.101		Fragmentation Evidence for Peptide (see below)

Fragmentation Evidence for Peptide

MQNVC[CAM]RPSMAQTGPGKLPSR

Residue	b	y
M	132.0478	2215.0893
Q	260.1063	2084.0488
N	374.1493	1955.9902
V	473.2177	1841.9473
C[CAM]	633.2483	1742.8789
R	789.3494	1582.8482
P	886.4022	1426.7471
S	973.4342	1329.6943
M	1104.4747	1242.6623
A	1175.5118	1111.6218
Q	1303.5704	1040.5847
T	1404.6181	912.5261
G	1461.6395	811.4785
P	1558.6923	754.4570
G	1615.7138	657.4042
K	1743.8087	600.3828
L	1856.8928	472.2878
P	1953.9456	359.2037
S	2040.9776	262.1510
R	2197.0787	175.1190

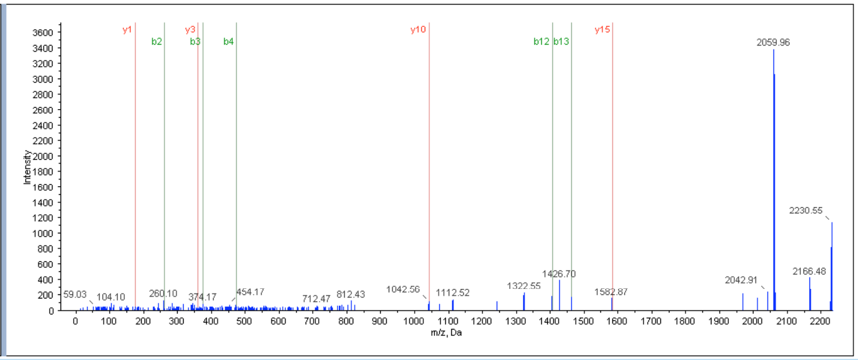

51	860.4683		Fragmentation Evidence for Peptide (see below)

Fragmentation Evidence for Peptide

DVVAMIGR

Residue	b	y
D	116.0342	860.4658
V	215.1026	745.4389
V	314.1710	646.3705
A	385.2082	547.3021
M	516.2486	476.2650
I	629.3327	345.2245
G	686.3542	232.1404
R	842.4553	175.1190

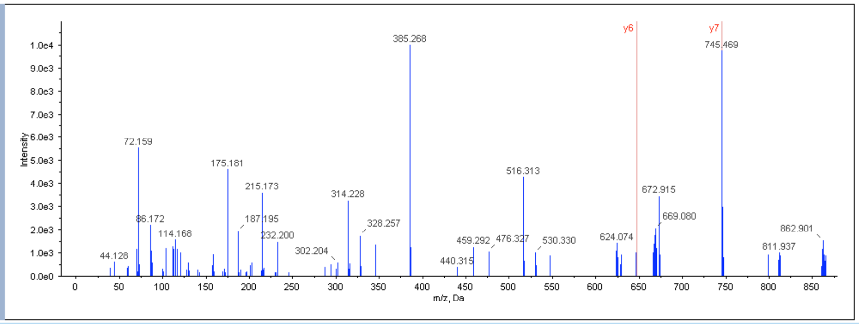

52	1143.606		

Fragmentation Evidence for Peptide

GFTGATPTVHR

Residue	b	y
G	58.0287	1143.5905
F	205.0972	1086.5691
T	306.1448	**939.5007**
G	**363.1663**	**838.4530**
A	**434.2034**	**781.4315**
T	**535.2511**	**710.3944**
P	632.3039	**609.3467**
T	733.3515	512.2940
V	**832.4199**	**411.2463**
H	**969.4789**	**312.1779**
R	1125.5800	**175.1190**

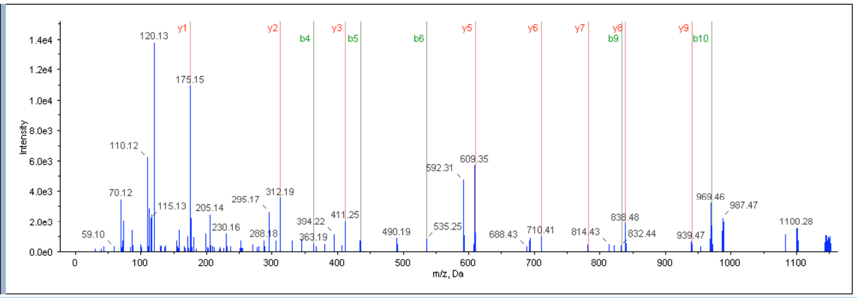

53	1464.684		

Fragmentation Evidence for Peptide

KIDSDLEGHPTPR

Residue	b	y
K	129.1022	1464.7441
I	242.1863	1336.6492
D	357.2132	1223.5651
S	444.2453	**1108.5382**
D	559.2722	1021.5061
L	672.3563	**906.4792**
E	801.3989	793.3951
G	858.4203	**664.3525**
H	**995.4793**	**607.3311**
P	1092.5320	**470.2722**
T	1193.5797	373.2194
P	1290.6325	**272.1717**
R	1446.7336	175.1190

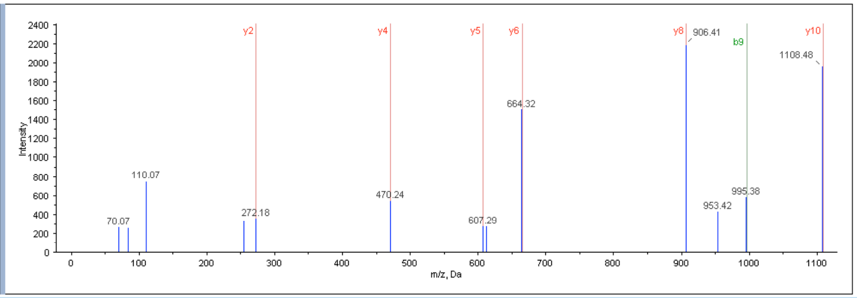

54	1713.894		

Fragmentation Evidence for Peptide

VAQPAYLPTEQDILR

Residue	b	y
V	100.0757	1713.9170
A	**171.1128**	1614.8486
Q	**299.1714**	1543.8115
P	396.2241	**1415.7529**
A	**467.2613**	1318.7001
Y	**630.3246**	1247.6630
L	**743.4087**	**1084.5997**
P	840.4614	**971.5156**
T	941.5091	874.4629
E	1070.5517	**773.4152**
Q	1198.6103	**644.3726**
D	1313.6372	**516.3140**
I	1426.7213	**401.2871**
L	1539.8053	**288.2030**
R	1695.9064	**175.1190**

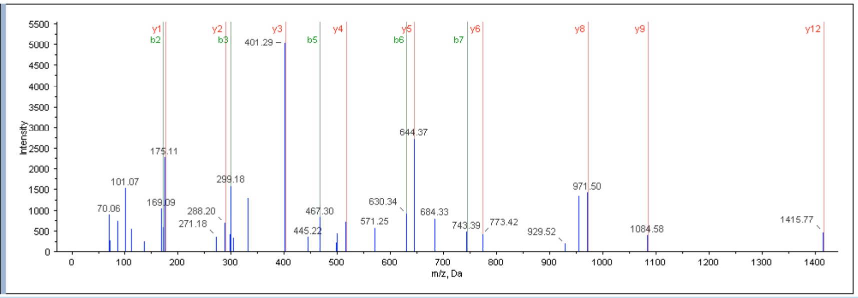

55	1733.859		Fragmentation Evidence for Peptide<br>SALYGRPAGSEGKAPSW

Residue	b	y
S	88.0393	1733.8606
A	**159.0764**	1646.8285
L	**272.1605**	1575.7914
Y	435.2238	1462.7074
G	492.2453	**1299.6440**
R	**648.3464**	**1242.6226**
P	745.3991	**1086.5214**
A	816.4363	989.4687
G	873.4577	918.4316
S	**960.4898**	861.4101
E	**1089.5323**	774.3781
G	**1146.5538**	645.3355
K	**1274.6488**	588.3140
A	1345.6859	460.2191
P	1442.7387	**389.1819**
S	**1529.7707**	**292.1292**
W	1715.8500	205.0972

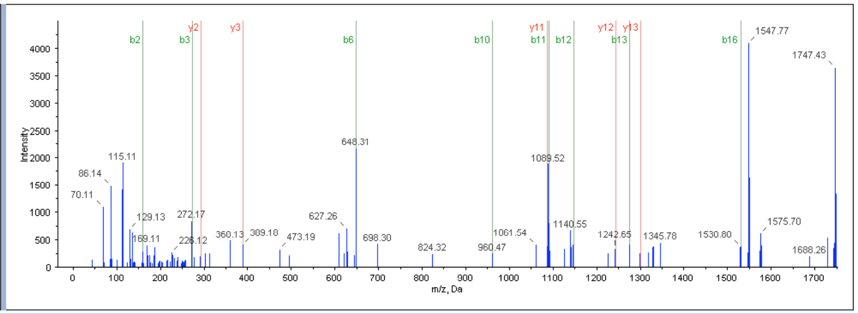

56	2685.247		Fragmentation Evidence for Peptide<br>AKDDYDRAVKEFEANGGSSAANGGGAK

Residue	b	y
A	72.0444	2685.2499
K	200.1394	2614.2128
D	315.1663	2486.1178
D	430.1932	**2371.0909**
Y	593.2566	2256.0640
D	708.2835	2093.0006
R	864.3846	**1977.9737**
A	935.4217	1821.8726
V	**1034.4901**	1750.8355
K	**1162.5851**	1651.7671
E	1291.6277	1523.6721
F	1438.6961	1394.6295
E	1567.7387	1247.5611
A	1638.7758	1118.5185
N	1752.8188	1047.4814
G	1809.8402	933.4384
G	1866.8617	876.4170
S	1953.8937	819.3955
S	2040.9257	732.3635
A	2111.9629	645.3315
A	2183.0000	574.2944
N	2297.0429	503.2572
G	2354.0644	**389.2143**
G	2411.0858	332.1928
G	2468.1073	275.1714
A	2539.1444	218.1499
K	2667.2394	147.1128

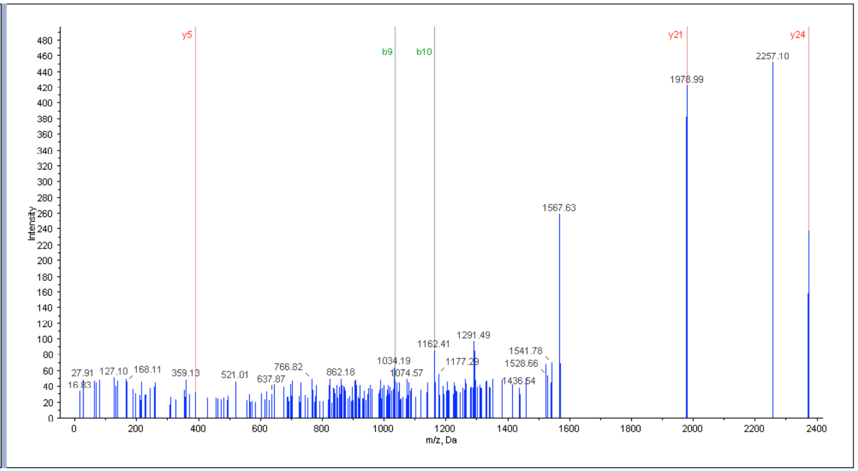

<table>
<tr>
<td>57</td>
<td>1732.955</td>
<td></td>
<td>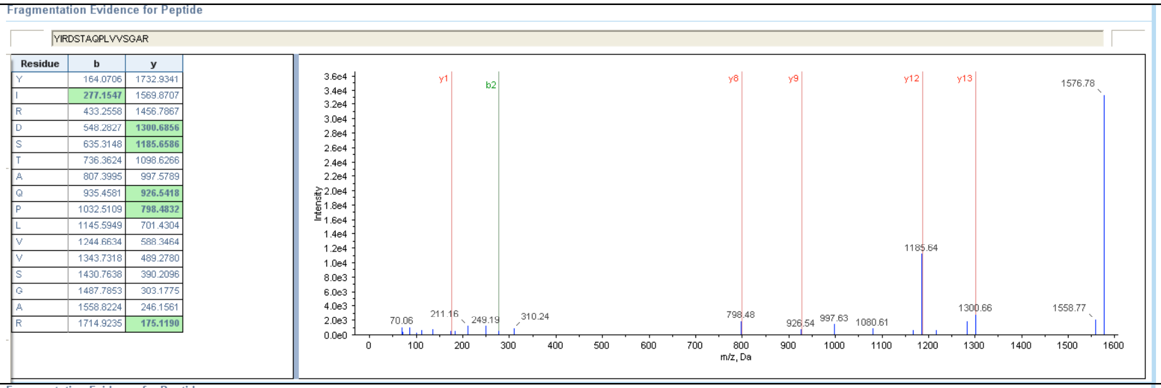
</td>
</tr>
<tr>
<td>58</td>
<td>1426.786</td>
<td></td>
<td>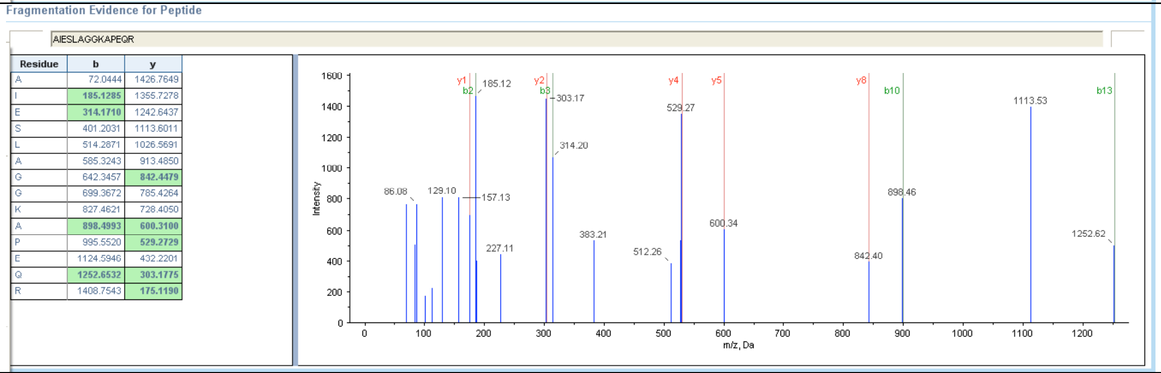
</td>
</tr>
<tr>
<td>59</td>
<td>1593.776</td>
<td></td>
<td>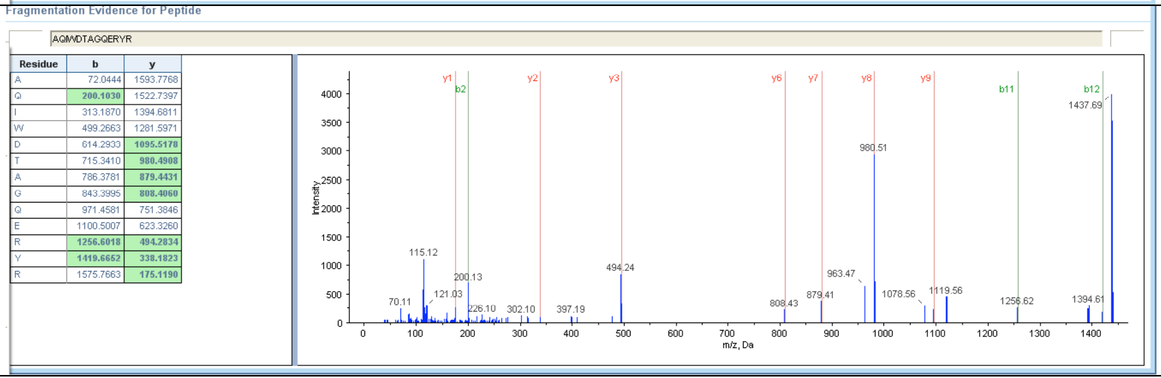
</td>
</tr>
</table>

60	2436.177		Fragmentation Evidence for Peptide

FAQYAIAGDYEGVKEFGSDLKK

Residue	b	y
F	148.0757	2436.2082
A	219.1128	2289.1398
Q	347.1714	2218.1026
Y	510.2347	2090.0441
A	581.2718	1926.9807
I	**694.3559**	1855.9436
A	765.3930	1742.8596
G	822.4145	**1671.8224**
D	937.4414	1614.8010
Y	1100.5047	**1499.7740**
E	1229.5473	**1336.7107**
G	1286.5688	1207.6681
V	1385.6372	1150.6467
K	1513.7322	1051.5782
E	**1642.7748**	923.4833
F	1789.8432	794.4407
G	1846.8646	647.3723
S	1933.8967	590.3508
D	2048.9236	503.3188
L	2162.0077	**388.2918**
K	2290.1026	275.2078
K	2418.1976	147.1128

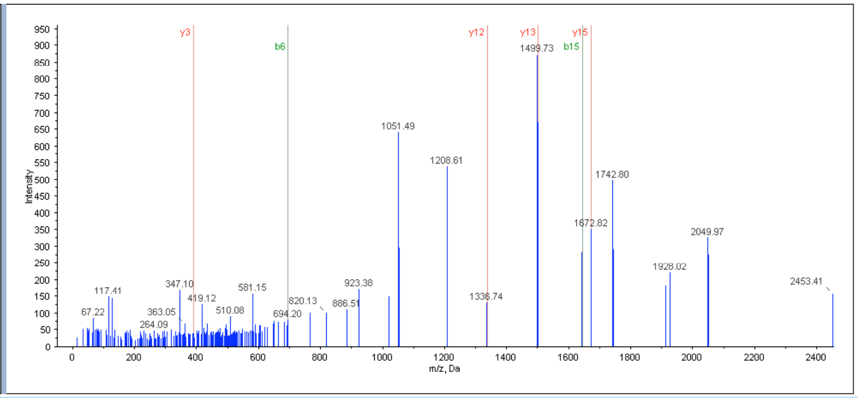

61	1577.802		Fragmentation Evidence for Peptide

AQQWAVPYVETSAK

Residue	b	y
A	72.0444	1577.7958
Q	**200.1030**	1506.7587
Q	**328.1615**	1378.7001
W	514.2409	1250.6416
A	**585.2780**	**1064.5623**
V	**684.3464**	**993.5251**
P	781.3991	**894.4567**
Y	944.4625	797.4040
V	**1043.5309**	**634.3406**
E	1172.5735	**535.2722**
T	1273.6212	406.2296
S	1360.6532	305.1819
A	1431.6903	218.1499
K	1559.7853	147.1128

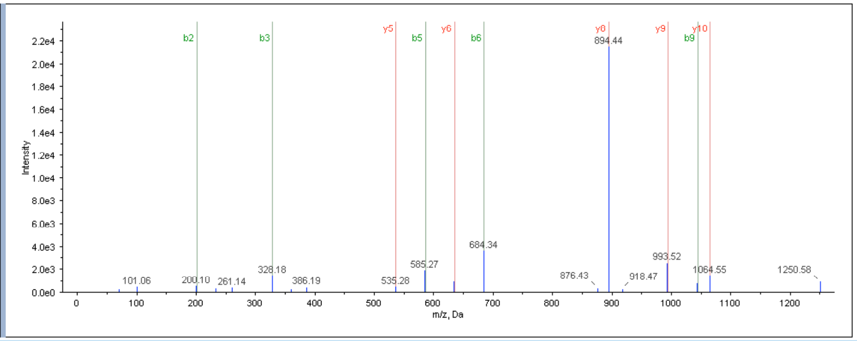

62	984.4777		Fragmentation Evidence for Peptide

KGYSPFGTK

Residue	b	y
K	**129.1022**	984.5149
G	186.1237	**856.4199**
Y	349.1870	**799.3985**
S	**436.2191**	**636.3352**
P	533.2718	**549.3031**
F	**680.3402**	452.2504
G	**737.3617**	305.1819
T	**838.4094**	248.1605
K	966.5043	147.1128

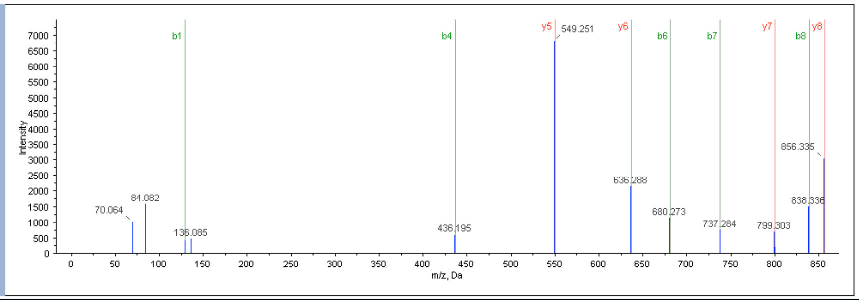

63	1741.764		

Fragmentation Evidence for Peptide

SFEDIGTYREQIKR

Residue	b	y
S	88.0393	1741.8868
F	235.1077	1654.8547
E	364.1503	1507.7863
D	479.1773	1378.7437
I	592.2613	1263.7168
G	649.2828	1150.6327
T	750.3305	1093.6113
Y	913.3938	992.5636
R	1069.4949	829.5003
E	1198.5375	673.3991
Q	1326.5961	544.3566
I	1439.6801	416.2980
K	1567.7751	303.2139
R	1723.8762	175.1190

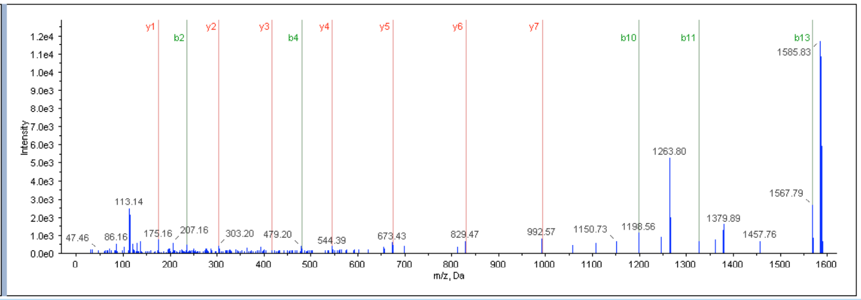

64	1538.761		

Fragmentation Evidence for Peptide

MSSVANIKPLSFNM

Residue	b	y
M	132.0478	1538.7705
S	219.0798	1407.7301
S	306.1118	1320.6980
V	405.1802	1233.6660
A	476.2173	1134.5976
N	590.2603	1063.5605
I	703.3443	949.5176
K	831.4393	836.4335
P	928.4921	708.3385
L	1041.5761	611.2858
S	1128.6082	498.2017
F	1275.6766	411.1697
N	1389.7195	264.1013
M	1520.7600	150.0583

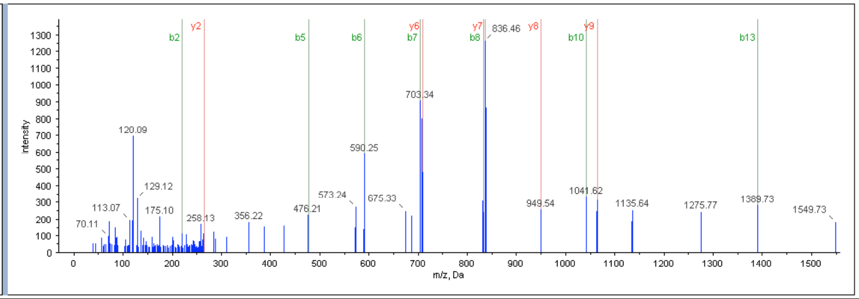

65	2213.16		

Fragmentation Evidence for Peptide

1Ac AQPQPPAYENKNIYATLPR

Residue	b	y
A	114.0550	2213.1349
Q	242.1135	2100.0873
P	339.1663	1972.0287
Q	467.2249	1874.9759
P	564.2776	1746.9173
P	661.3304	1649.8646
A	732.3675	1552.8118
Y	895.4308	1481.7747
E	1024.4734	1318.7114
N	1138.5164	1189.6688
K	1266.6113	1075.6259
N	1380.6543	947.5309
I	1493.7383	833.4880
Y	1656.8016	720.4039
A	1727.8388	557.3406
T	1828.8864	486.3035
L	1941.9705	385.2558
P	2039.0233	272.1717
R	2195.1244	175.1190

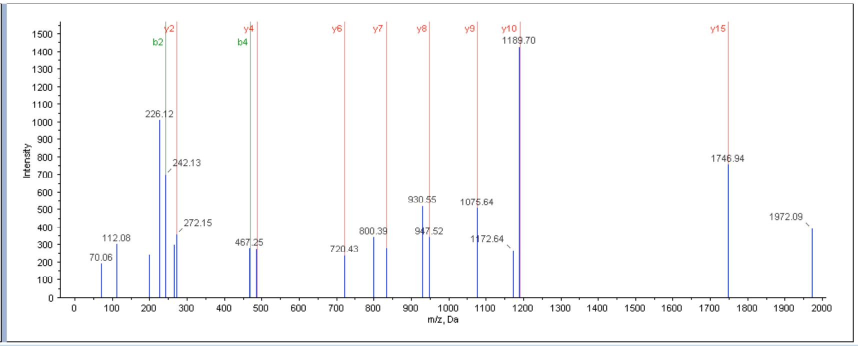

<table>
<tr>
<td>66</td>
<td>940.5369</td>
<td></td>
<td>Fragmentation Evidence for Peptide
LGPWIAQR
<table>
<tr><th>Residue</th><th>b</th><th>y</th></tr>
<tr><td>L</td><td>114.0913</td><td>940.5363</td></tr>
<tr><td>G</td><td>171.1128</td><td>827.4522</td></tr>
<tr><td>P</td><td>268.1656</td><td>770.4308</td></tr>
<tr><td>W</td><td>454.2449</td><td>673.3780</td></tr>
<tr><td>I</td><td>567.3289</td><td>487.2987</td></tr>
<tr><td>A</td><td>638.3661</td><td>374.2146</td></tr>
<tr><td>Q</td><td>766.4246</td><td>303.1775</td></tr>
<tr><td>R</td><td>922.5257</td><td>175.1190</td></tr>
</table>
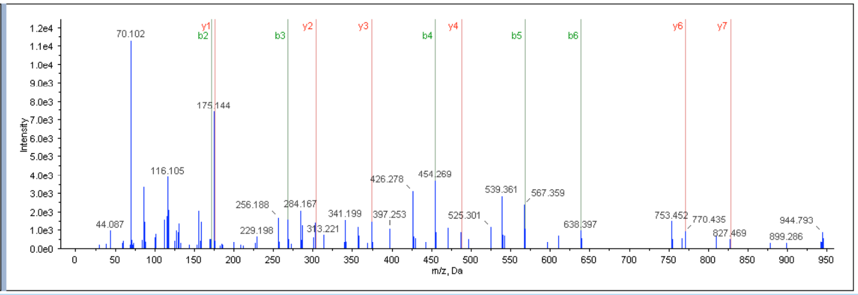

</td>
</tr>
<tr>
<td>67</td>
<td>943.4778</td>
<td></td>
<td>Fragmentation Evidence for Peptide
NFHEGLAR
<table>
<tr><th>Residue</th><th>b</th><th>y</th></tr>
<tr><td>N</td><td>115.0502</td><td>943.4744</td></tr>
<tr><td>F</td><td>262.1186</td><td>829.4315</td></tr>
<tr><td>H</td><td>399.1775</td><td>682.3631</td></tr>
<tr><td>E</td><td>528.2201</td><td>545.3042</td></tr>
<tr><td>G</td><td>585.2416</td><td>416.2616</td></tr>
<tr><td>L</td><td>698.3257</td><td>359.2401</td></tr>
<tr><td>A</td><td>769.3628</td><td>246.1561</td></tr>
<tr><td>R</td><td>925.4639</td><td>175.1190</td></tr>
</table>
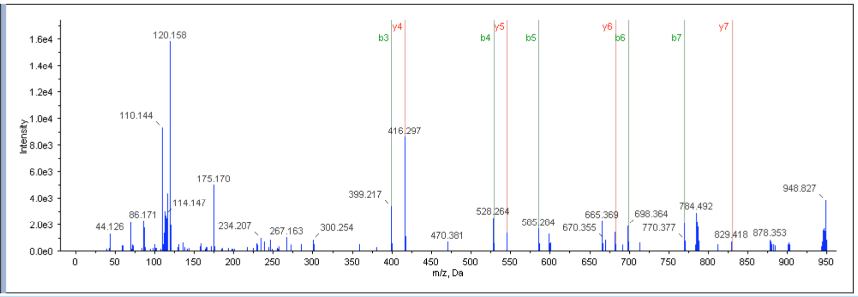

</td>
</tr>
<tr>
<td>68</td>
<td>1182.619</td>
<td></td>
<td>Fragmentation Evidence for Peptide
DSKLLHGQER
<table>
<tr><th>Residue</th><th>b</th><th>y</th></tr>
<tr><td>D</td><td>116.0342</td><td>1182.6226</td></tr>
<tr><td>S</td><td>203.0662</td><td>1067.5956</td></tr>
<tr><td>K</td><td>331.1612</td><td>980.5636</td></tr>
<tr><td>L</td><td>444.2453</td><td>852.4686</td></tr>
<tr><td>L</td><td>557.3293</td><td>739.3846</td></tr>
<tr><td>H</td><td>694.3883</td><td>626.3005</td></tr>
<tr><td>G</td><td>751.4097</td><td>489.2416</td></tr>
<tr><td>Q</td><td>879.4683</td><td>432.2201</td></tr>
<tr><td>E</td><td>1008.5109</td><td>304.1615</td></tr>
<tr><td>R</td><td>1164.6120</td><td>175.1190</td></tr>
</table>
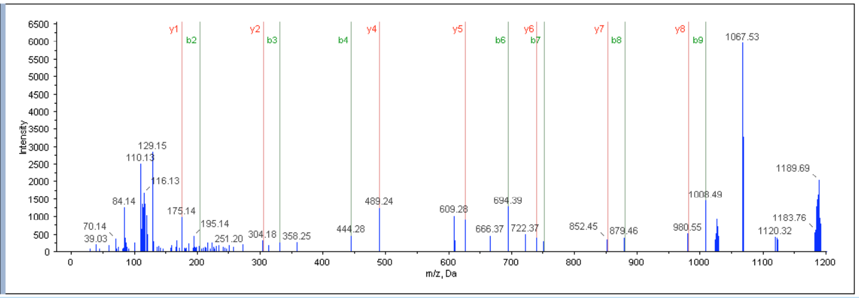

</td>
</tr>
</table>

69	1142.575		Fragmentation Evidence for Peptide: YTMLKDMIK (see below)
70	1460.751		Fragmentation Evidence for Peptide: FAEEVITAEPAQR (see below)
71	1327.765		Fragmentation Evidence for Peptide: DAAKQTGPIVISK (see below)

Fragmentation Evidence for Peptide

YTMLKDMIK

Residue	b	y
Y	164.0706	1142.5948
T	265.1183	979.5315
M	396.1588	878.4838
L	509.2428	747.4433
K	637.3378	634.3593
D	752.3647	506.2643
M	883.4052	391.2374
I	996.4893	260.1969
K	1124.5842	147.1128

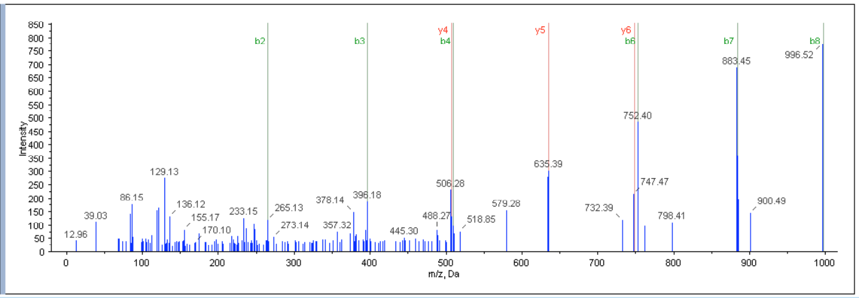

Fragmentation Evidence for Peptide

FAEEVITAEPAQR

Residue	b	y
F	148.0757	1460.7380
A	219.1128	1313.6696
E	348.1554	1242.6325
E	477.1980	1113.5899
V	576.2664	984.5473
I	689.3505	885.4789
T	790.3981	772.3948
A	861.4353	671.3471
E	990.4779	600.3100
P	1087.5306	471.2674
A	1158.5677	374.2146
Q	1286.6263	303.1775
R	1442.7274	175.1190

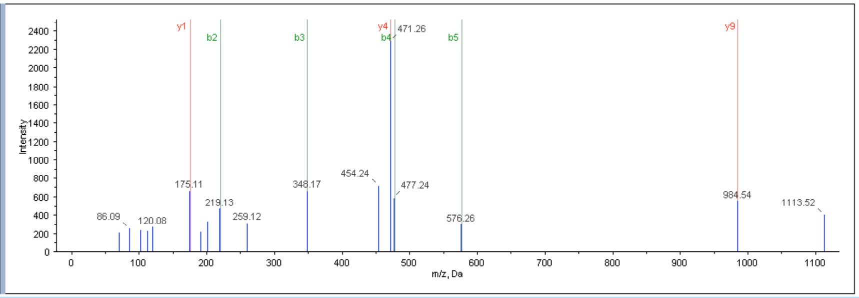

Fragmentation Evidence for Peptide

DAAKQTGPIVISK

Residue	b	y
D	116.0342	1327.7580
A	187.0713	1212.7310
A	258.1084	1141.6939
K	386.2034	1070.6568
Q	514.2620	942.5619
T	615.3097	814.5033
G	672.3311	713.4556
P	769.3839	656.4341
I	882.4680	559.3814
V	981.5364	446.2973
I	1094.6204	347.2289
S	1181.6525	234.1448
K	1309.7474	147.1128

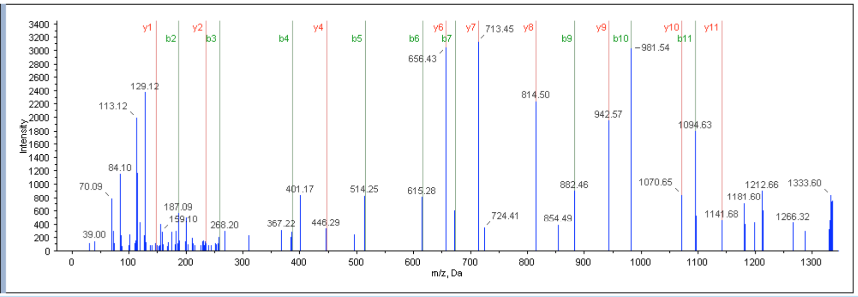

72	1497.734		

Fragmentation Evidence for Peptide

ASSGIEGNPTVFYR

Residue	b	y
A	72.0444	1497.7332
S	**159.0764**	1426.6961
S	**246.1084**	1339.6641
G	**303.1299**	1252.6321
I	416.2140	1195.6106
E	545.2566	1082.5265
G	602.2780	**953.4839**
N	**716.3210**	896.4625
P	813.3737	**782.4196**
T	**914.4214**	685.3668
V	1013.4898	**584.3191**
F	1160.5582	**485.2507**
Y	1323.6216	**338.1823**
R	1479.7227	**175.1190**

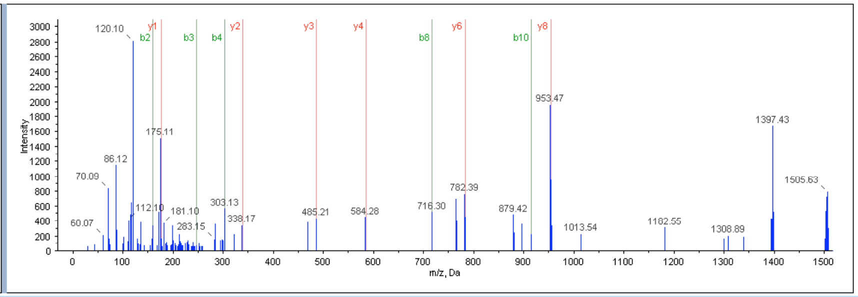

73	1460.7		

Fragmentation Evidence for Peptide

FYGPGGPYATFAGR

Residue	b	y
F	148.0757	1460.6957
Y	**311.1390**	**1313.6273**
G	**368.1605**	**1150.5640**
P	465.2132	**1093.5425**
G	522.2347	996.4898
G	**579.2562**	**939.4683**
P	676.3089	**882.4468**
Y	**839.3723**	785.3941
A	**910.4094**	**622.3307**
T	**1011.4571**	**551.2936**
F	1158.5255	**450.2459**
A	1229.5626	**303.1775**
G	1286.5841	**232.1404**
R	1442.6852	**175.1190**

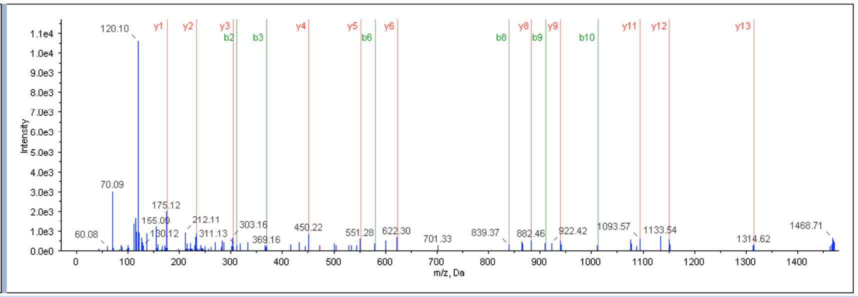

74	2935.26		

Fragmentation Evidence for Peptide

1Ac SKPVPMSPSFVDEDLHNLRKPKPFK

Residue	b	y
S	130.0499	2935.5499
K	258.1448	2806.5073
P	355.1976	2678.4123
V	454.2660	2581.3595
P	551.3188	2482.2911
M	682.3593	2385.2384
S	769.3913	**2254.1979**
P	866.4441	**2167.1658**
S	953.4761	2070.1131
F	1100.5445	1983.0811
V	1199.6129	1836.0126
D	1314.6399	**1736.9442**
E	1443.6824	**1621.9173**
D	1558.7094	**1492.8747**
L	1671.7935	**1377.8478**
H	1808.8524	**1264.7637**
N	1922.8953	**1127.7048**
L	2035.9794	**1013.6618**
R	**2192.0805**	**900.5778**
K	**2320.1754**	744.4767
P	2417.2282	616.3817
K	2545.3232	519.3289
P	2642.3759	391.2340
F	2789.4443	294.1812
K	2917.5393	147.1128

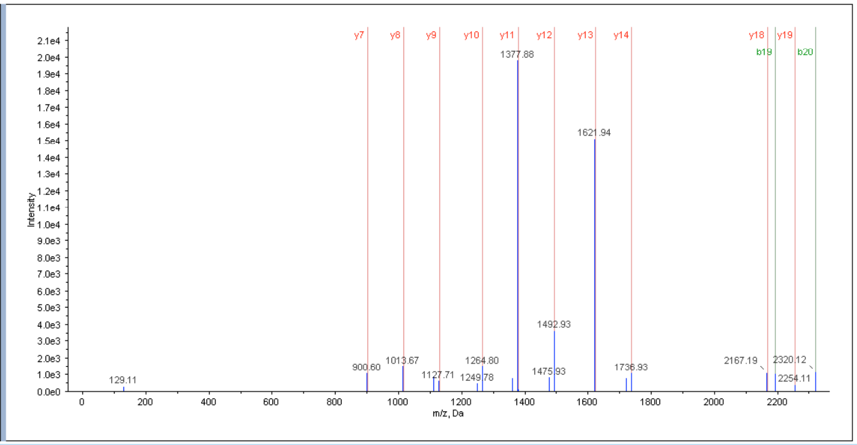

75	1887.949		Fragmentation Evidence for Peptide — VTHAVVTVPAYFNDAQR
76	1545.769		Fragmentation Evidence for Peptide — DARQEASVIGESGAR
77	1908.855		Fragmentation Evidence for Peptide — YHPFYITDSPEGGLGQK

Fragmentation Evidence for Peptide

VTHAVVTVPAYFNDAQR

Residue	b	y
V	100.0757	1887.9712
T	201.1234	1788.9028
H	338.1823	1687.8551
A	409.2194	1550.7962
V	508.2878	1479.7591
V	607.3562	1380.6906
T	708.4039	1281.6222
V	807.4723	1180.5745
P	904.5251	1081.5061
A	975.5622	984.4534
Y	1138.6255	913.4163
F	1285.6939	750.3529
N	1399.7369	603.2845
D	1514.7638	489.2416
A	1585.8009	374.2146
Q	1713.8595	303.1775
R	1869.9606	175.1190

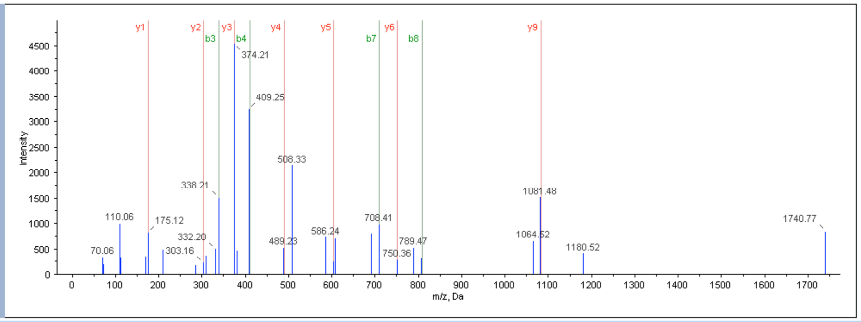

Fragmentation Evidence for Peptide

DARQEASVIGESGAR

Residue	b	y
D	116.0342	1545.7616
A	187.0713	1430.7346
R	343.1724	1359.6975
Q	471.2310	1203.5964
E	600.2736	1075.5378
A	671.3107	946.4952
S	758.3428	875.4581
V	857.4112	788.4261
I	970.4952	689.3577
G	1027.5167	576.2736
E	1156.5593	519.2522
S	1243.5913	390.2096
G	1300.6128	303.1775
A	1371.6499	246.1561
R	1527.7510	175.1190

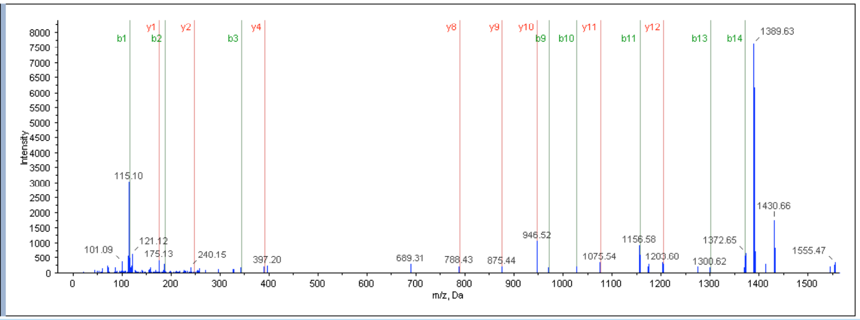

Fragmentation Evidence for Peptide

YHPFYITDSPEGGLGQK

Residue	b	y
Y	164.0706	1908.9127
H	301.1295	1745.8493
P	398.1823	1608.7904
F	545.2507	1511.7377
Y	708.3140	1364.6692
I	821.3981	1201.6059
T	922.4458	1088.5218
D	1037.4727	987.4742
S	1124.5047	872.4472
P	1221.5575	785.4152
E	1350.6001	688.3624
G	1407.6216	559.3198
G	1464.6430	502.2984
L	1577.7271	445.2769
G	1634.7485	332.1928
Q	1762.8071	275.1714
K	1890.9021	147.1128

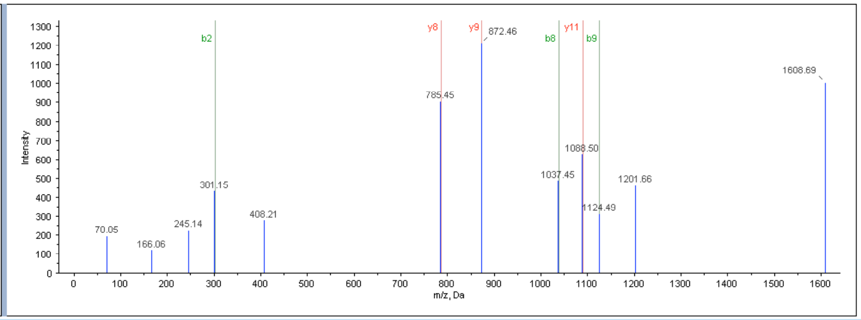

78	2131.004	

Fragmentation Evidence for Peptide

SAAVAEGGDAVAETAKGEEGSPK

Residue	b	y
S	88.0393	2131.0149
A	159.0764	2043.9829
A	230.1135	1972.9458
V	329.1819	1901.9087
A	400.2191	1802.8403
E	529.2617	1731.8032
G	586.2831	1602.7606
G	643.3046	1545.7391
D	758.3315	1488.7176
A	829.3686	1373.6907
V	928.4371	1302.6536
A	999.4742	1203.5852
E	1128.5168	1132.5481
T	1229.5644	1003.5055
A	1300.6016	902.4578
K	1428.6965	831.4207
G	1485.7180	703.3257
E	1614.7606	646.3042
E	1743.8032	517.2617
G	1800.8246	388.2191
S	1887.8567	331.1976
P	1984.9094	244.1656
K	2113.0044	147.1128

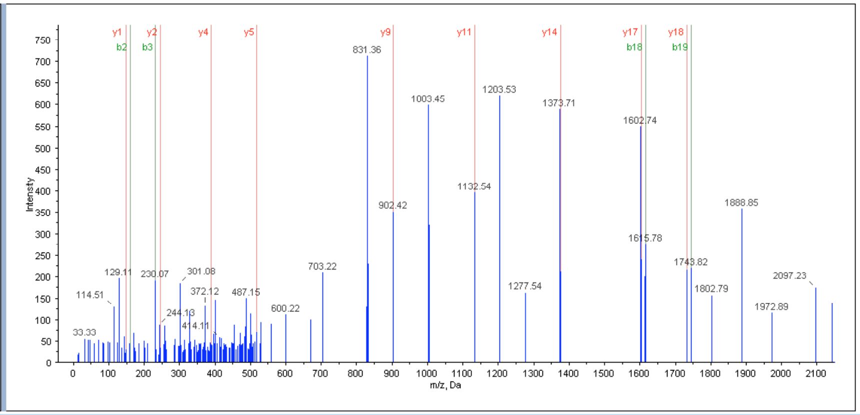

79	1220.679	

Fragmentation Evidence for Peptide

TPSASKEPLYK

Residue	b	y
T	102.0550	1220.6521
P	199.1077	1119.6045
S	286.1397	1022.5517
A	357.1769	935.5197
S	444.2089	864.4825
K	572.3039	777.4505
E	701.3464	649.3556
P	798.3992	520.3130
L	911.4833	423.2602
Y	1074.5466	310.1761
K	1202.6416	147.1128

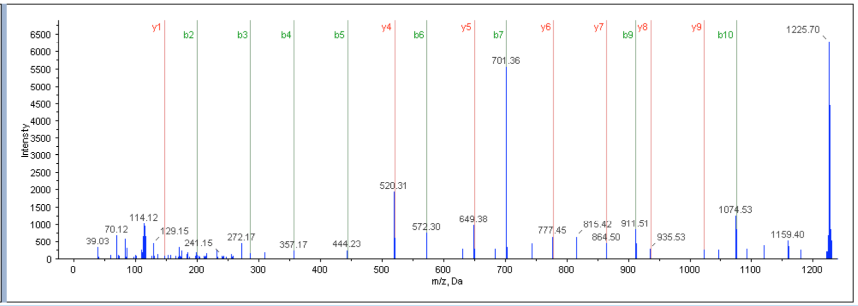

80	1901.002	

Fragmentation Evidence for Peptide

AAVDAGFVPNDLQIGQTGK

Residue	b	y
A	72.0444	1900.9763
A	143.0815	1829.9392
V	242.1499	1758.9021
D	357.1769	1659.8337
A	428.2140	1544.8067
G	485.2354	1473.7696
F	632.3039	1416.7482
V	731.3723	1269.6797
P	828.4250	1170.6113
N	942.4680	1073.5586
D	1057.4949	959.5156
L	1170.5790	844.4887
Q	1298.6375	731.4046
I	1411.7216	603.3461
G	1468.7431	490.2620
Q	1596.8016	433.2405
T	1697.8493	305.1819
G	1754.8708	204.1343
K	1882.9658	147.1128

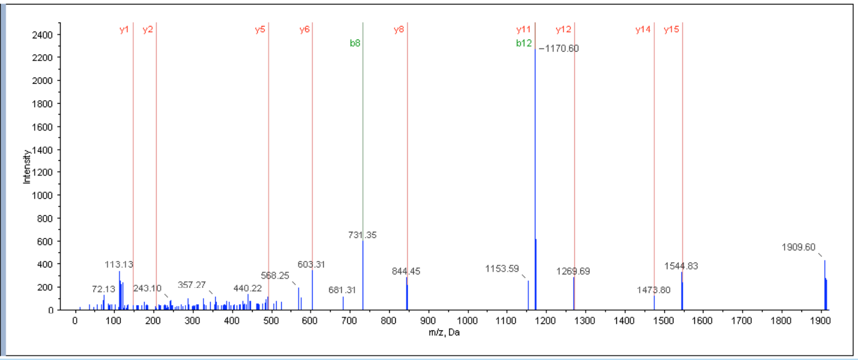

81	2176.132	

Fragmentation Evidence for Peptide

MGPSTDPTAVVDPQLRVHGAK

Residue	b	y
M	132.0478	2176.1179
G	**189.0692**	2045.0774
P	**286.1220**	1988.0560
S	373.1540	1891.0032
T	474.2017	1803.9712
D	589.2286	1702.9235
P	686.2814	**1587.8966**
T	787.3291	1490.8438
A	858.3662	**1389.7961**
V	957.4346	**1318.7590**
V	1056.5030	1219.6906
D	1171.5300	1120.6222
P	1268.5827	1005.5952
Q	1396.6413	908.5425
L	1509.7254	780.4839
R	1665.8265	667.3998
V	1764.8949	511.2987
H	1901.9538	412.2303
G	1958.9753	**275.1714**
A	2030.0124	218.1499
K	2158.1073	147.1128

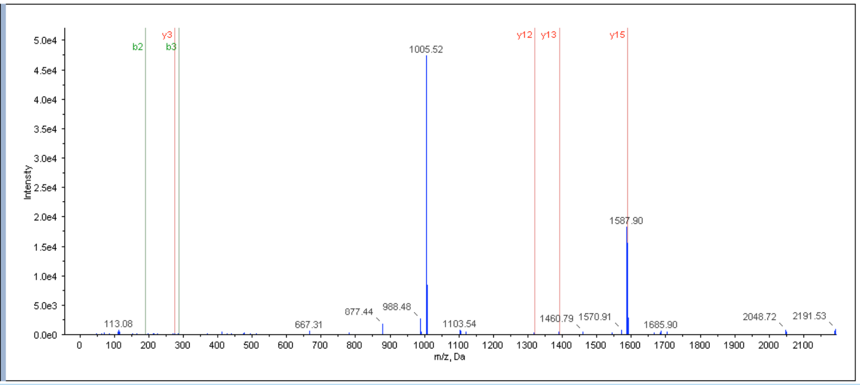

82	2126.868	

Fragmentation Evidence for Peptide

HSNYADEDDEDTRDMTGR

Residue	b	y
H	138.0662	2126.8316
S	**225.0982**	1989.7727
N	339.1411	1902.7406
Y	502.2045	1788.6977
A	573.2416	1625.6344
D	688.2685	1554.5973
E	817.3111	**1439.5703**
D	932.3381	1310.5277
D	1047.3650	1195.5008
E	1176.4076	1080.4738
D	1291.4345	951.4313
T	1392.4822	836.4043
R	1548.5833	735.3566
D	1663.6103	**579.2555**
M	1794.6508	464.2286
T	1895.6984	**333.1881**
G	**1952.7199**	232.1404
R	2108.8210	175.1190

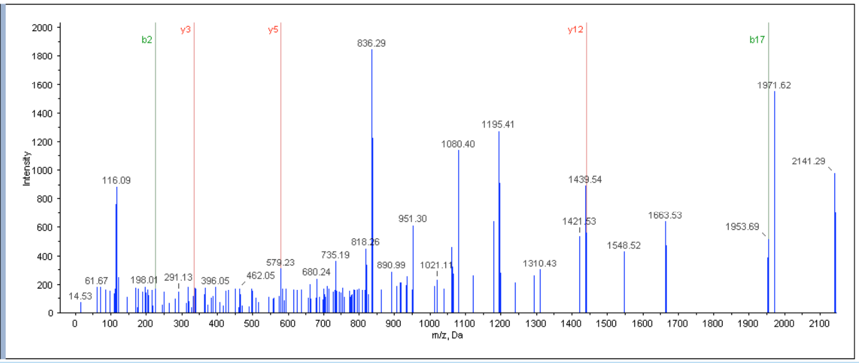

83	1035.568	

Fragmentation Evidence for Peptide

TFITQQGIK

Residue	b	y
T	102.0550	1035.5833
F	**249.1234**	934.5356
I	**362.2074**	**787.4672**
T	**463.2551**	**674.3832**
Q	**591.3137**	**573.3355**
Q	**719.3723**	**445.2769**
G	**776.3937**	**317.2183**
I	**889.4778**	260.1969
K	1017.5728	**147.1128**

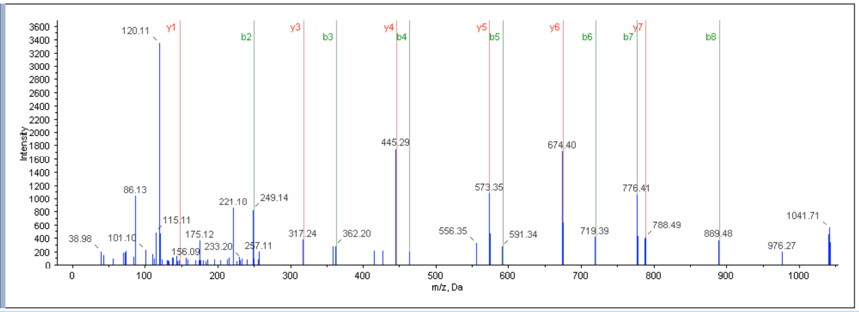

<table>
<tr>
<td>84</td>
<td>1726.831</td>
<td></td>
<td>

Fragmentation Evidence for Peptide

SGEYEEVRERPFTK

Residue	b	y
S	88.0393	1726.8395
G	145.0608	1639.8075
E	274.1034	1582.7860
Y	437.1667	1453.7434
E	566.2093	1290.6801
E	695.2519	1161.6375
V	794.3203	1032.5949
R	950.4214	933.5265
E	1079.4640	777.4254
R	1235.5651	648.3828
P	1332.6179	492.2817
F	1479.6863	395.2289
T	1580.7340	248.1605
K	1708.8289	147.1128

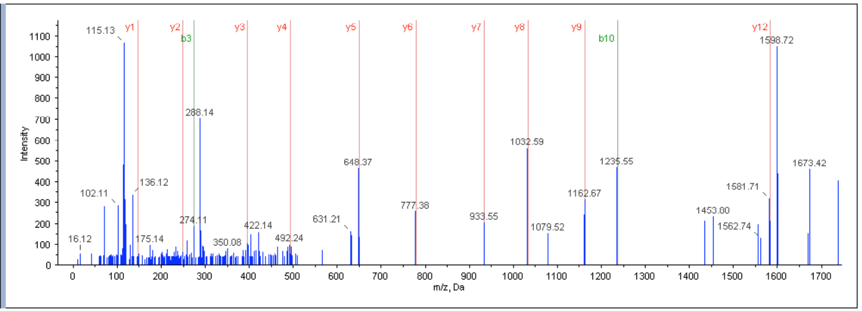

</td>
</tr>
<tr>
<td>85</td>
<td>1328.637</td>
<td></td>
<td>

Fragmentation Evidence for Peptide

RAPSYTFYAPR

Residue	b	y
R	157.1084	1328.6746
A	228.1455	1172.5735
P	325.1983	1101.5364
S	412.2303	1004.4836
Y	575.2936	917.4516
T	676.3413	754.3883
F	823.4097	653.3406
Y	986.4730	506.2722
A	1057.5102	343.2088
P	1154.5629	272.1717
R	1310.6640	175.1190

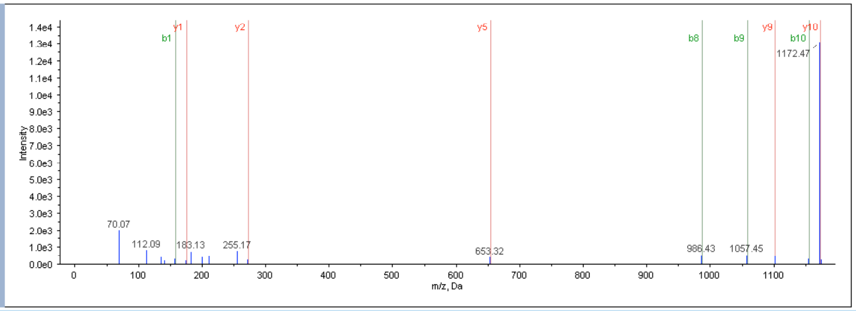

</td>
</tr>
<tr>
<td>86</td>
<td>1840.852</td>
<td></td>
<td>

Fragmentation Evidence for Peptide

ELDFNNDLENSQKFK

Residue	b	y
E	130.0499	1840.8712
L	243.1339	1711.8286
D	358.1609	1598.7445
F	505.2293	1483.7176
N	619.2722	1336.6492
N	733.3151	1222.6062
D	848.3421	1108.5633
L	961.4262	993.5364
E	1090.4687	880.4523
N	1204.5117	751.4097
S	1291.5437	637.3668
Q	1419.6023	550.3348
K	1547.6972	422.2762
F	1694.7657	294.1812
K	1822.8606	147.1128

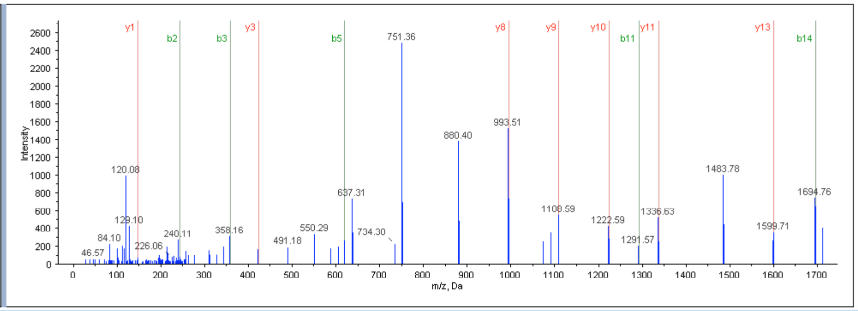

</td>
</tr>
</table>

87	2237.079		

Fragmentation Evidence for Peptide

KDESVAEDFHGTQIKDVYR

Residue	b	y
K	**129.1022**	2237.0833
D	**244.1292**	2108.9883
E	373.1718	1993.9614
S	460.2038	**1864.9188**
V	559.2722	1777.8868
A	630.3093	1678.8184
E	759.3519	1607.7812
D	874.3789	**1478.7387**
F	1021.4473	**1363.7117**
H	1158.5062	**1216.6433**
G	1215.5277	**1079.5844**
T	1316.5753	1022.5629
Q	1444.6339	921.5152
I	1557.7180	**793.4567**
K	1685.8129	680.3726
D	1800.8399	552.2776
V	1899.9083	437.2507
Y	**2062.9716**	338.1823
R	2219.0727	**175.1190**

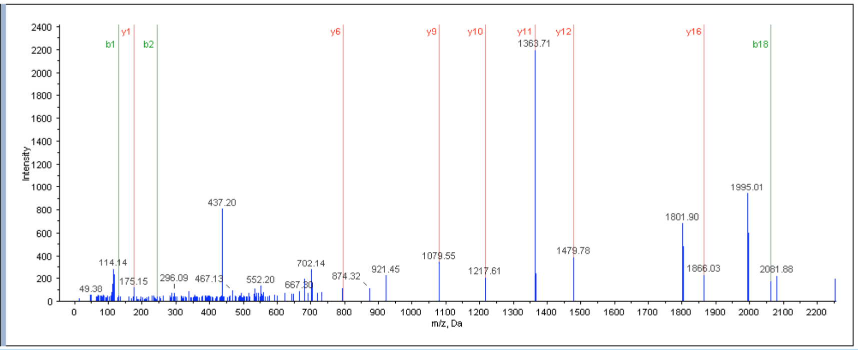

88	971.5056		

Fragmentation Evidence for Peptide

GQHFGNLAK

Residue	b	y
G	58.0287	971.5057
Q	186.0873	914.4843
H	**323.1462**	**786.4257**
F	**470.2146**	**649.3668**
G	**527.2361**	**502.2984**
N	**641.2790**	**445.2769**
L	**754.3631**	331.2340
A	**825.4002**	218.1499
K	953.4952	147.1128

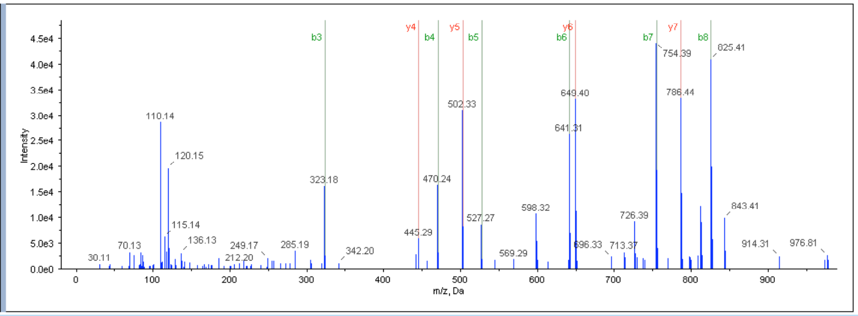

89	1275.562		

Fragmentation Evidence for Peptide

SC[CAM]YFGNIADTK

Residue	b	y
S	88.0393	1275.5674
C[CAM]	**248.0700**	1188.5354
Y	**411.1333**	1028.5047
F	**558.2017**	**865.4414**
G	**615.2232**	**718.3730**
N	**729.2661**	**661.3515**
I	**842.3502**	547.3086
A	913.3873	**434.2245**
D	**1028.4142**	**363.1874**
T	1129.4619	248.1605
K	1257.5568	**147.1128**

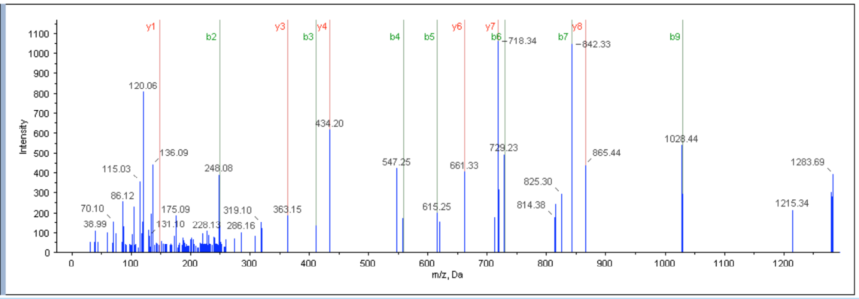

<table>
<tr><td>90</td><td>1482.706</td><td></td><td>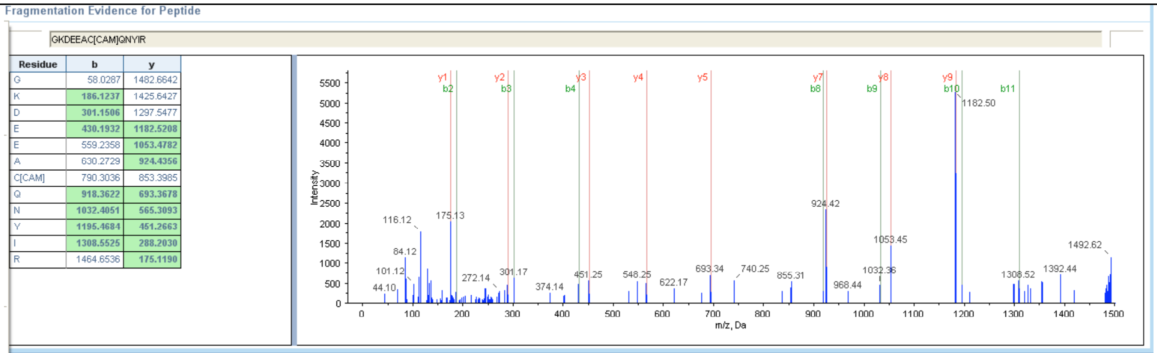
</td></tr>
<tr><td>91</td><td>1918.965</td><td></td><td>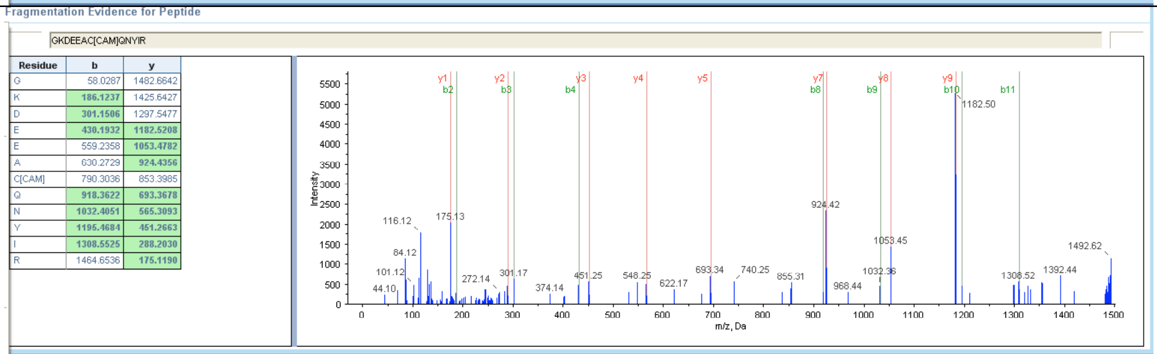
</td></tr>
<tr><td>92</td><td>1110.561</td><td></td><td>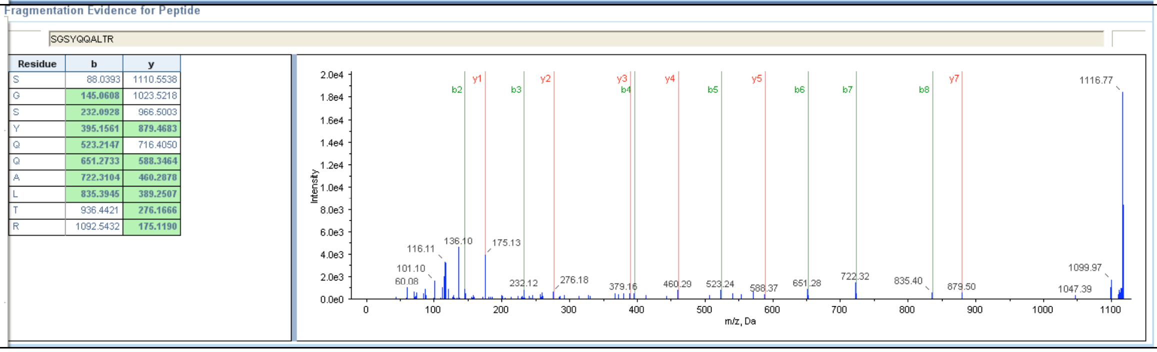
</td></tr>
</table>

93	1439.751		Fragmentation Evidence for Peptide

GVTQINFFEKEK

Residue	b	y
G	58.0287	1439.7529
V	**157.0972**	1382.7314
T	**258.1448**	**1283.6630**
Q	**386.2034**	1182.6154
I	**499.2875**	1054.5568
N	613.3304	**941.4727**
F	**760.3988**	**827.4298**
F	**907.4672**	**680.3614**
E	1036.5098	**533.2930**
K	1164.6048	**404.2504**
E	**1293.6474**	**276.1554**
K	1421.7423	**147.1128**

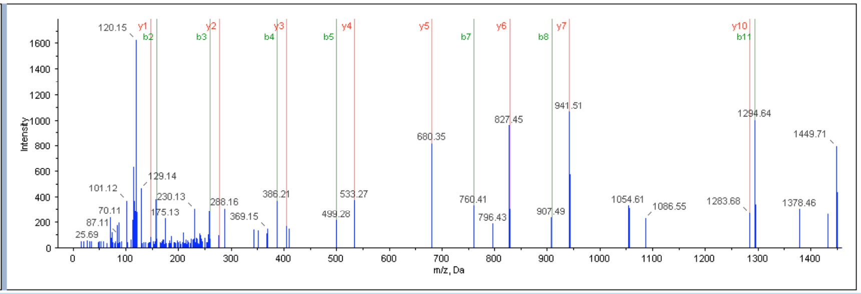

94	1716.844		Fragmentation Evidence for Peptide

SSPEIVPASFSNAPSAR

Residue	b	y
S	88.0393	1716.8551
S	**175.0713**	1629.8231
P	272.1241	1542.7911
E	**401.1667**	1445.7383
I	**514.2508**	**1316.6957**
V	613.3192	1203.6117
P	710.3719	**1104.5432**
A	781.4090	1007.4905
S	868.4411	**936.4534**
F	1015.5095	849.4213
S	1102.5415	702.3529
N	1216.5844	**615.3209**
A	1287.6216	**501.2780**
P	1384.6743	**430.2409**
S	1471.7064	333.1881
A	1542.7435	246.1561
R	1698.8446	**175.1190**

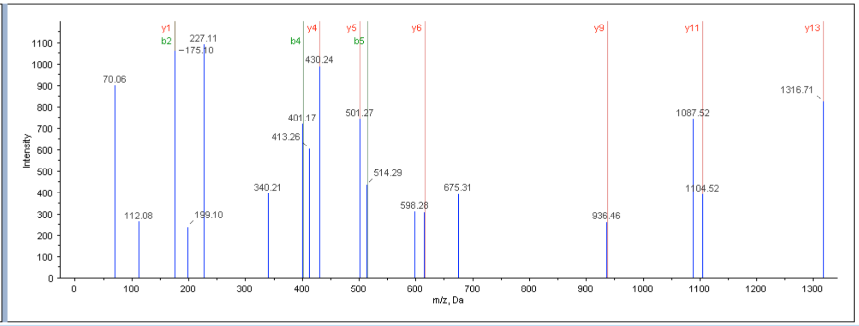

95	1724.94		Fragmentation Evidence for Peptide

AAQQLAEKESPIKLAK

Residue	b	y
A	72.0444	1724.9905
A	**143.0815**	1653.9534
Q	**271.1401**	1582.9163
Q	**399.1987**	1454.8577
L	**512.2827**	**1326.7991**
A	**583.3198**	**1213.7151**
E	712.3624	**1142.6780**
K	840.4574	**1013.6354**
E	**969.5000**	**885.5404**
S	**1056.5320**	**756.4978**
P	**1153.5848**	**669.4658**
I	**1266.6688**	572.4130
K	**1394.7638**	459.3289
L	**1507.8479**	331.2340
A	**1578.8850**	218.1499
K	1706.9799	147.1128

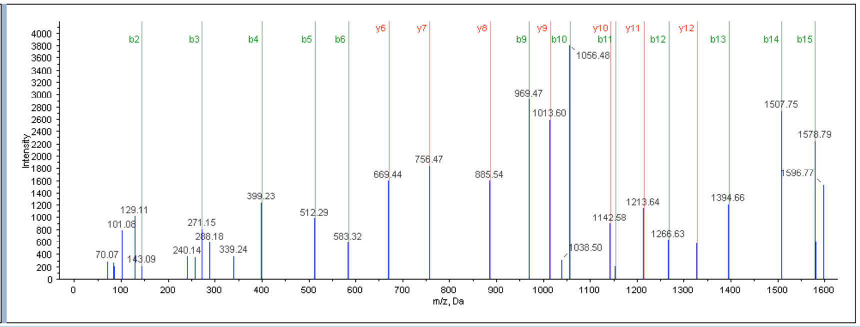

96	959.5087		

Fragmentation Evidence for Peptide

DAVTNVQGR

Residue	b	y
D	116.0342	959.4905
A	187.0713	**844.4635**
V	286.1397	773.4264
T	**387.1874**	**674.3580**
N	**501.2304**	**573.3103**
V	600.2988	459.2674
Q	728.3573	360.1990
G	785.3788	**232.1404**
R	941.4799	175.1190

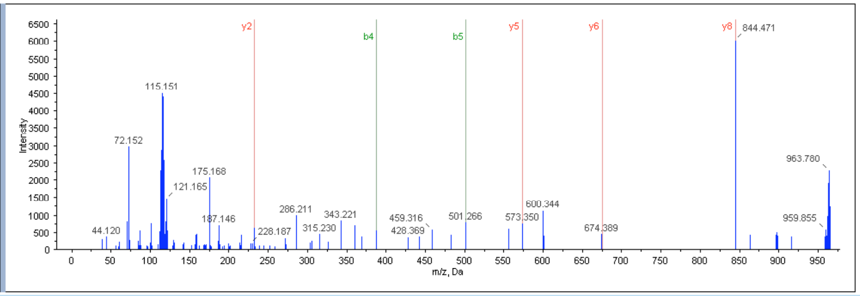

97	2122.914		

Fragmentation Evidence for Peptide

ISAEAAMQHPYFTDSSSSGH

Residue	b	y
I	**114.0913**	2122.9135
S	**201.1234**	2009.8294
A	**272.1605**	1922.7974
E	401.2031	1851.7603
A	472.2402	1722.7177
A	**543.2773**	1651.6805
M	**674.3178**	1580.6434
Q	802.3764	**1449.6029**
H	939.4353	**1321.5444**
P	1036.4880	**1184.4855**
Y	1199.5514	1087.4327
F	1346.6198	924.3694
T	1447.6675	777.3010
D	**1562.6944**	676.2533
S	1649.7264	**561.2263**
S	1736.7585	474.1943
S	1823.7905	387.1623
S	1910.8225	300.1302
G	1967.8440	**213.0982**
H	2104.9029	**156.0768**

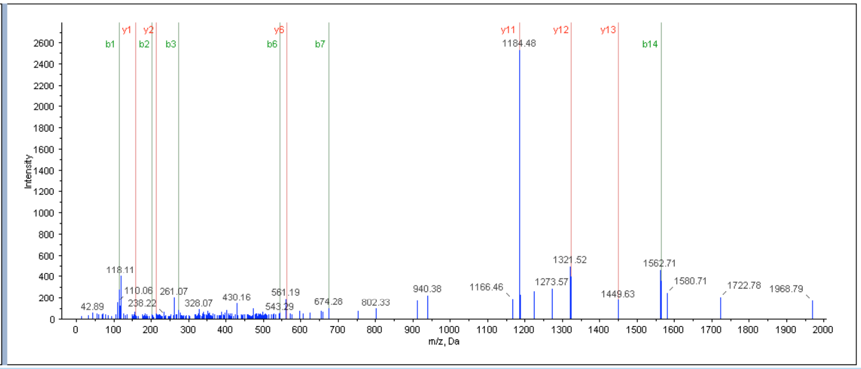

98	2448.198		

Fragmentation Evidence for Peptide

VKDTFQDFAQSPAIAGGAESSPPK

Residue	b	y
V	100.0757	2448.2041
K	228.1707	2349.1357
D	343.1976	2221.0408
T	444.2453	2106.0138
F	591.3137	2004.9661
Q	719.3723	1857.8977
D	834.3992	1729.8392
F	981.4676	**1614.8122**
A	1052.5047	**1467.7438**
Q	**1180.5633**	**1396.7067**
S	**1267.5953**	**1268.6481**
P	1364.6481	1181.6161
A	1435.6852	1084.5633
I	**1548.7693**	1013.5262
A	**1619.8064**	**900.4421**
G	1676.8279	829.4050
G	1733.8493	772.3836
A	**1804.8864**	715.3621
E	1933.9290	644.3250
S	2020.9611	515.2824
S	2107.9931	428.2504
P	2205.0459	341.2183
P	2302.0986	**244.1656**
K	2430.1936	**147.1128**

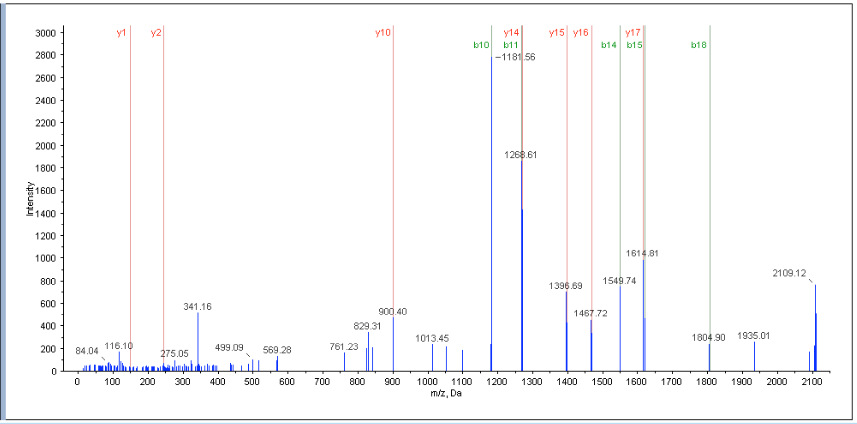

Khanna et al. Supplementary Table 3
(Part 2. Spectra #99-#198)

Spectra for single peptide identifications

Spectrum Number	Precursor m/z	Spectrum
99	2022.986	see below

Fragmentation Evidence for Peptide

SGTQMILPYRGDDSDVIR

Residue	b	y
S	**88.0393**	2022.9913
G	**145.0608**	1935.9593
T	246.1084	1878.9378
Q	374.1670	1777.8901
M	505.2075	1649.8316
I	618.2916	1518.7911
L	731.3756	1405.7070
P	828.4284	1292.6230
Y	991.4917	1195.5702
R	**1147.5928**	1032.5069
G	1204.6143	876.4058
D	**1319.6412**	819.3843
D	**1434.6682**	704.3573
S	1521.7002	589.3304
D	**1636.7272**	502.2984
V	1735.7956	387.2714
I	**1848.8796**	288.2030
R	2004.9808	**175.1190**

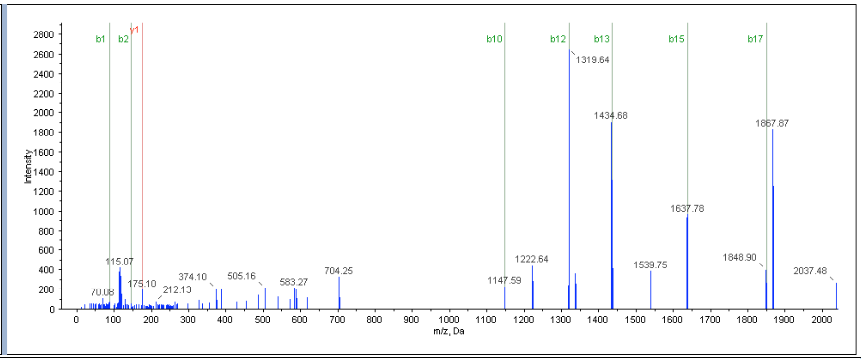

Spectrum Number	Precursor m/z	Spectrum
100	1589.873	see below

Fragmentation Evidence for Peptide

VELTPPTPADIPAIR

Residue	b	y
V	100.0757	1589.8897
E	**229.1183**	1490.8213
L	342.2023	**1361.7787**
T	443.2500	1248.6947
P	540.3028	**1147.6470**
P	637.3556	1050.5942
T	738.4032	953.5415
P	835.4560	852.4938
A	906.4931	755.4410
D	1021.5201	**684.4039**
I	1134.6041	**569.3770**
P	1231.6569	**456.2929**
A	1302.6940	359.2401
I	1415.7781	288.2030
R	1571.8792	**175.1190**

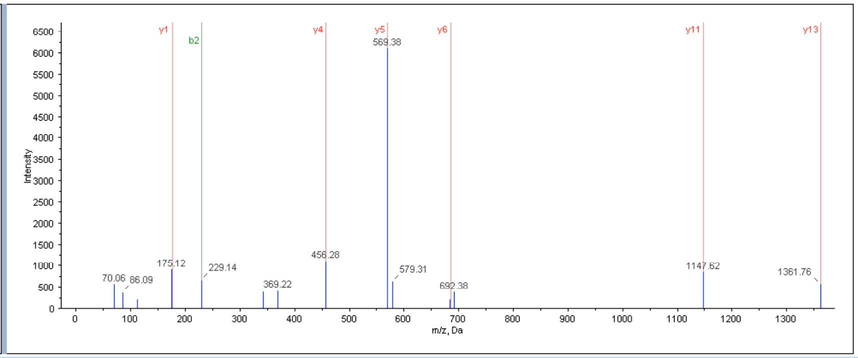

<table>
<tr>
<td>101</td>
<td>2159.945</td>
<td></td>
<td>
Fragmentation Evidence for Peptide
YGSNVDGISTC[CAM]DPEQYAKR
<table>
<tr><th>Residue</th><th>b</th><th>y</th></tr>
<tr><td>Y</td><td>164.0706</td><td>2159.9662</td></tr>
<tr><td>G</td><td>221.0921</td><td>1996.9029</td></tr>
<tr><td>S</td><td>308.1241</td><td>1939.8814</td></tr>
<tr><td>N</td><td>422.1670</td><td>1852.8494</td></tr>
<tr><td>V</td><td>521.2354</td><td>1738.8065</td></tr>
<tr><td>D</td><td>636.2624</td><td>1639.7381</td></tr>
<tr><td>G</td><td>693.2838</td><td>1524.7111</td></tr>
<tr><td>I</td><td>806.3679</td><td>1467.6897</td></tr>
<tr><td>S</td><td>893.3999</td><td>1354.6056</td></tr>
<tr><td>T</td><td>994.4476</td><td>1267.5736</td></tr>
<tr><td>C[CAM]</td><td>1154.4783</td><td>1166.5259</td></tr>
<tr><td>D</td><td>1269.5052</td><td>1006.4952</td></tr>
<tr><td>P</td><td>1366.5580</td><td>891.4683</td></tr>
<tr><td>E</td><td>1495.6006</td><td>794.4155</td></tr>
<tr><td>Q</td><td>1623.6591</td><td>665.3729</td></tr>
<tr><td>Y</td><td>1786.7225</td><td>537.3144</td></tr>
<tr><td>A</td><td>1857.7596</td><td>374.2510</td></tr>
<tr><td>K</td><td>1985.8545</td><td>303.2139</td></tr>
<tr><td>R</td><td>2141.9557</td><td>175.1190</td></tr>
</table>
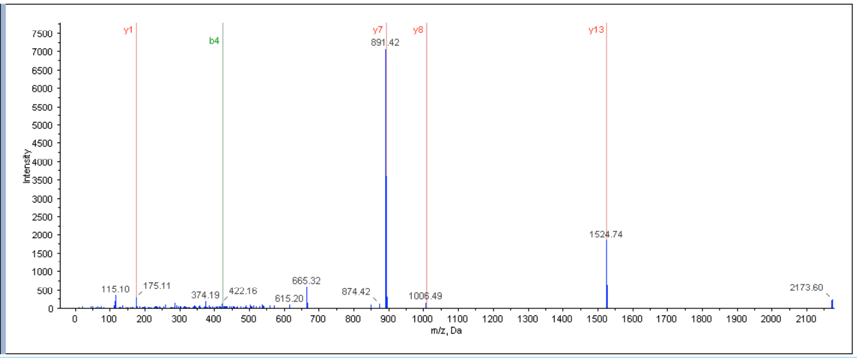

</td>
</tr>
<tr>
<td>102</td>
<td>2297.174</td>
<td></td>
<td>
Fragmentation Evidence for Peptide
VVPKEEVPPPTVPMDYSWAR
<table>
<tr><th>Residue</th><th>b</th><th>y</th></tr>
<tr><td>V</td><td>100.0757</td><td>2297.1635</td></tr>
<tr><td>V</td><td>199.1441</td><td>2198.0951</td></tr>
<tr><td>P</td><td>296.1969</td><td>2099.0266</td></tr>
<tr><td>K</td><td>424.2918</td><td>2001.9739</td></tr>
<tr><td>E</td><td>553.3344</td><td>1873.8789</td></tr>
<tr><td>E</td><td>682.3770</td><td>1744.8363</td></tr>
<tr><td>V</td><td>781.4454</td><td>1615.7937</td></tr>
<tr><td>P</td><td>878.4982</td><td>1516.7253</td></tr>
<tr><td>P</td><td>975.5510</td><td>1419.6725</td></tr>
<tr><td>P</td><td>1072.6037</td><td>1322.6198</td></tr>
<tr><td>T</td><td>1173.6514</td><td>1225.5670</td></tr>
<tr><td>V</td><td>1272.7198</td><td>1124.5193</td></tr>
<tr><td>P</td><td>1369.7726</td><td>1025.4509</td></tr>
<tr><td>M</td><td>1500.8131</td><td>928.3982</td></tr>
<tr><td>D</td><td>1615.8400</td><td>797.3577</td></tr>
<tr><td>Y</td><td>1778.9033</td><td>682.3307</td></tr>
<tr><td>S</td><td>1865.9354</td><td>519.2674</td></tr>
<tr><td>W</td><td>2052.0147</td><td>432.2354</td></tr>
<tr><td>A</td><td>2123.0518</td><td>246.1561</td></tr>
<tr><td>R</td><td>2279.1529</td><td>175.1190</td></tr>
</table>
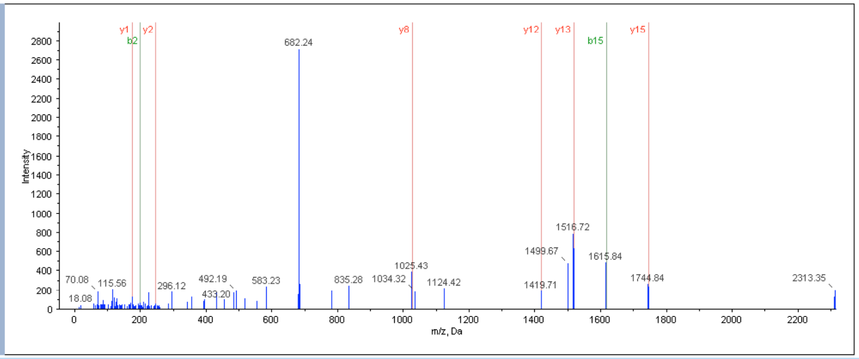

</td>
</tr>
<tr>
<td>103</td>
<td>1304.655</td>
<td></td>
<td>
Fragmentation Evidence for Peptide
GVYPEDALRER
<table>
<tr><th>Residue</th><th>b</th><th>y</th></tr>
<tr><td>G</td><td>58.0287</td><td>1304.6593</td></tr>
<tr><td>V</td><td>157.0972</td><td>1247.6379</td></tr>
<tr><td>Y</td><td>320.1605</td><td>1148.5695</td></tr>
<tr><td>P</td><td>417.2132</td><td>985.5061</td></tr>
<tr><td>E</td><td>546.2558</td><td>888.4534</td></tr>
<tr><td>D</td><td>661.2828</td><td>759.4108</td></tr>
<tr><td>A</td><td>732.3199</td><td>644.3838</td></tr>
<tr><td>L</td><td>845.4040</td><td>573.3467</td></tr>
<tr><td>R</td><td>1001.5051</td><td>460.2627</td></tr>
<tr><td>E</td><td>1130.5477</td><td>304.1615</td></tr>
<tr><td>R</td><td>1286.6488</td><td>175.1190</td></tr>
</table>
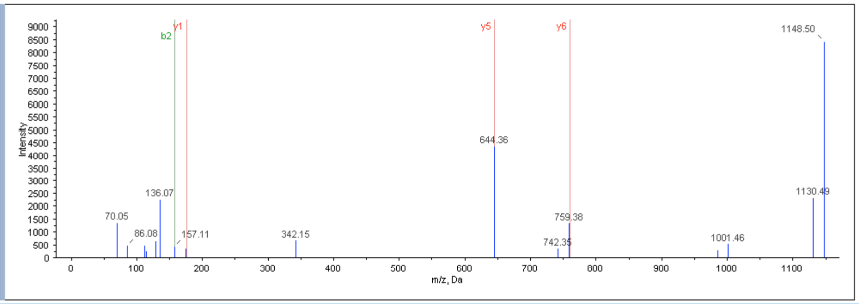

</td>
</tr>
</table>

104	1763.903		Fragmentation Evidence for Peptide

LIAEEADKKYEEVAR

Residue	b	y
L	114.0913	1763.9174
I	**227.1754**	1650.8333
A	**298.2125**	1537.7493
E	427.2551	1466.7122
E	556.2977	**1337.6696**
A	627.3348	**1208.6270**
D	742.3618	1137.5899
K	870.4567	**1022.5629**
K	**998.5517**	**894.4680**
Y	1161.6150	766.3730
E	1290.6576	603.3097
E	1419.7002	**474.2671**
V	1518.7686	**345.2245**
A	**1589.8057**	**246.1561**
R	1745.9068	**175.1190**

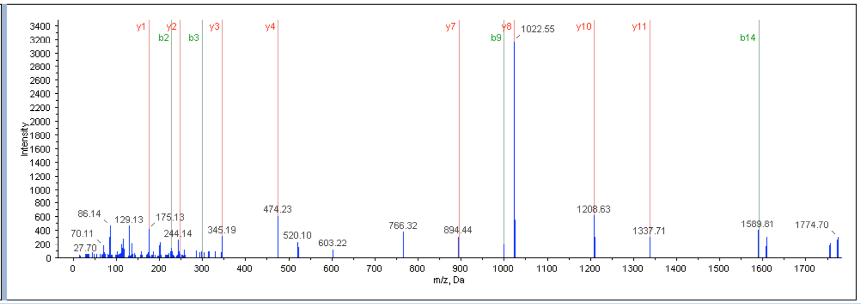

105	1676.827		Fragmentation Evidence for Peptide

KYNEIDNAPEEKAR

Residue	b	y
K	129.1022	1676.8238
Y	292.1656	1548.7289
N	406.2085	1385.6655
E	535.2511	1271.6226
I	648.3352	1142.5800
D	763.3621	**1029.4960**
N	877.4050	**914.4690**
A	948.4421	800.4261
P	1045.4949	729.3890
E	1174.5375	632.3362
E	1303.5801	**503.2936**
K	1431.6750	**374.2510**
A	1502.7122	**246.1561**
R	1658.8133	**175.1190**

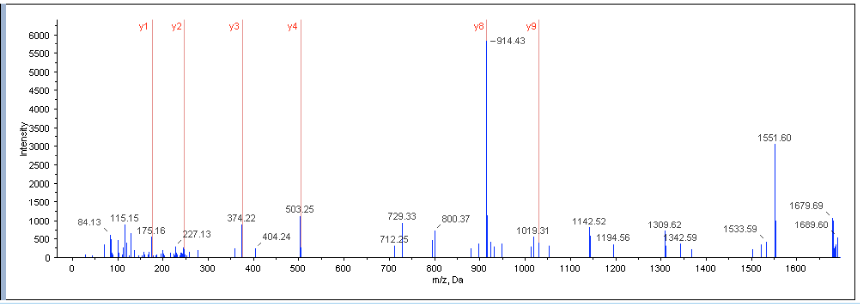

106	958.4904		Fragmentation Evidence for Peptide

FGAFEFLK

Residue	b	y
F	**148.0757**	958.5033
G	**205.0972**	811.4349
A	**276.1343**	754.4134
F	**423.2027**	**683.3763**
E	**552.2453**	**536.3079**
F	**699.3137**	**407.2653**
L	**812.3978**	**260.1969**
K	940.4927	**147.1128**

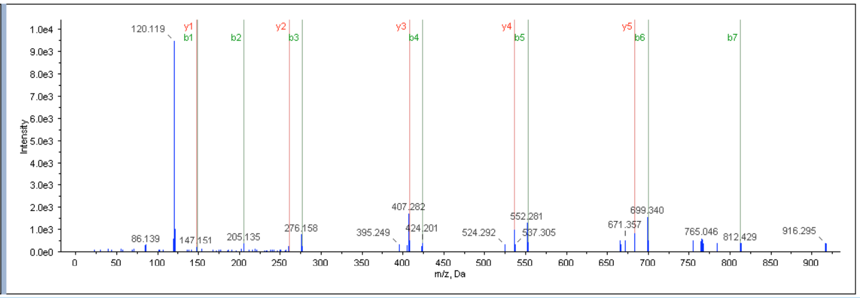

107	2349.187	Fragmentation Evidence for Peptide (see below)

Fragmentation Evidence for Peptide

SAYDVFAPGLTQGGATPATQLQR

Residue	b	y
S	88.0393	2349.1833
A	**159.0764**	2262.1513
Y	**322.1397**	2191.1142
D	437.1667	2028.0509
V	536.2351	1913.0239
F	683.3035	1813.9555
A	**754.3406**	1666.8871
P	851.3934	1595.8500
G	908.4149	1498.7972
L	1021.4989	1441.7758
T	1122.5466	1328.6917
Q	1250.6052	1227.6440
G	1307.6266	1099.5854
G	1364.6481	**1042.5640**
A	1435.6852	985.5425
T	1536.7329	914.5054
P	1633.7857	**813.4577**
A	1704.8228	716.4050
T	1805.8705	**645.3678**
Q	1933.9290	544.3202
L	2047.0131	416.2616
Q	2175.0717	**303.1775**
R	2331.1728	**175.1190**

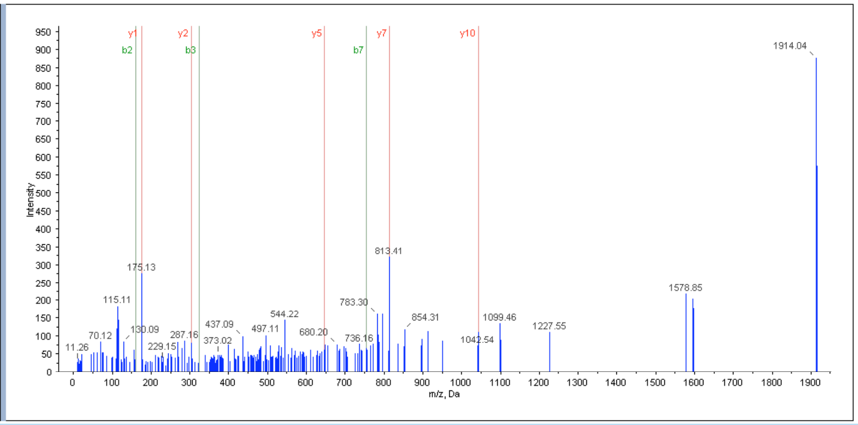

108	1604.75	Fragmentation Evidence for Peptide (see below)

Fragmentation Evidence for Peptide

AEEMQRREEEAAR

Residue	b	y
A	**72.0444**	1604.7445
E	**201.0870**	1533.7074
E	**330.1296**	1404.6648
M	461.1701	**1275.6222**
Q	589.2286	1144.5818
R	745.3297	**1016.5232**
R	**901.4309**	860.4221
E	1030.4735	**704.3210**
E	**1159.5160**	575.2784
E	**1288.5586**	446.2358
A	1359.5958	317.1932
A	1430.6329	**246.1561**
R	1586.7340	**175.1190**

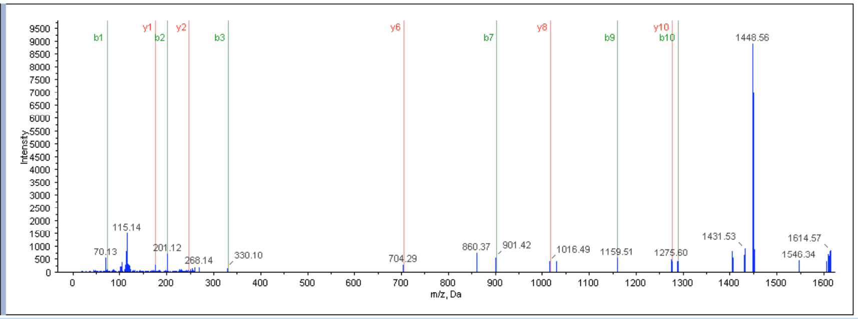

109	1895.028	Fragmentation Evidence for Peptide (see below)

Fragmentation Evidence for Peptide

AGDPVILEEAKIKEIAAK

Residue	b	y
A	72.0444	1895.0848
G	129.0659	1824.0477
D	244.0928	1767.0262
P	**341.1456**	**1651.9993**
V	**440.2140**	1554.9465
I	**553.2980**	1455.8781
L	**666.3821**	1342.7940
E	795.4247	1229.7100
E	924.4673	**1100.6674**
A	995.5044	**971.6248**
K	1123.5994	900.5877
I	**1236.6834**	**772.4927**
K	1364.7784	659.4087
E	**1493.8210**	531.3137
I	1606.9050	402.2711
A	**1677.9422**	289.1870
A	1748.9793	218.1499
K	1877.0742	**147.1128**

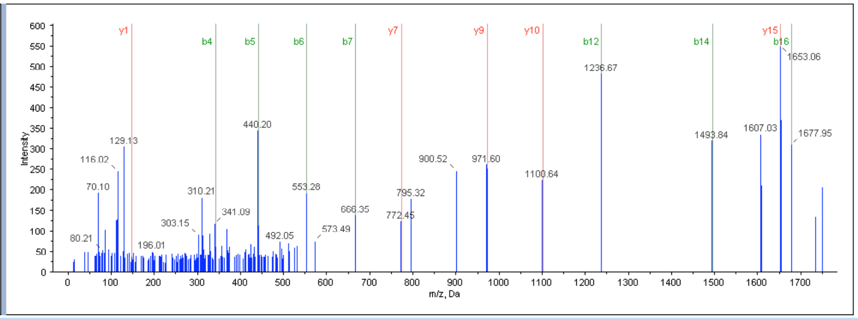

110	1627.828	

Fragmentation Evidence for Peptide

MSTGTGNVGLAQQHAR

Residue	b	y
M	132.0478	1627.7969
S	**219.0798**	1496.7564
T	320.1275	1409.7244
G	377.1489	1308.6767
T	478.1966	1251.6553
G	535.2181	**1150.6076**
N	649.2610	1093.5861
V	748.3294	**979.5432**
G	805.3509	880.4748
L	918.4349	823.4533
A	989.4721	710.3692
Q	1117.5306	639.3321
Q	1245.5892	511.2736
H	1382.6481	383.2150
A	1453.6852	**246.1561**
R	1609.7863	**175.1190**

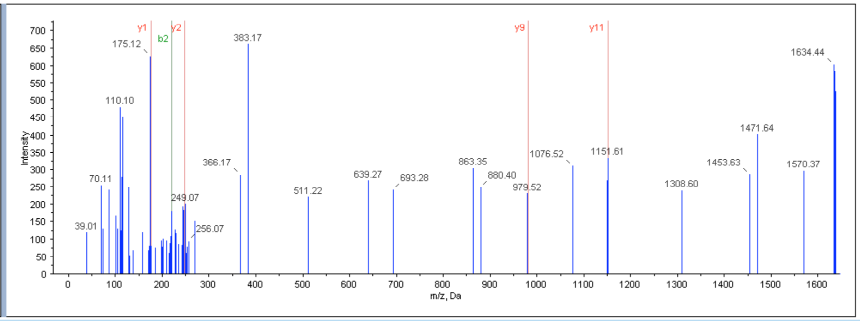

111	2007.9	

Fragmentation Evidence for Peptide

LAQSGDATC[CAM]NGLDSAEVGSR

Residue	b	y
L	114.0913	2007.9036
A	**185.1285**	1894.8196
Q	**313.1870**	1823.7824
S	**400.2191**	1695.7239
G	457.2405	1608.6918
D	572.2675	1551.6704
A	**643.3046**	**1436.6434**
T	744.3523	1365.6063
C[CAM]	904.3829	1264.5586
N	1018.4258	**1104.5280**
G	1075.4473	**990.4851**
L	1188.5314	933.4636
D	1303.5583	**820.3795**
S	1390.5903	**705.3526**
A	1461.6274	**618.3206**
E	1590.6700	547.2835
V	1689.7385	**418.2409**
G	1746.7599	**319.1724**
S	1833.7919	**262.1510**
R	1989.8931	**175.1190**

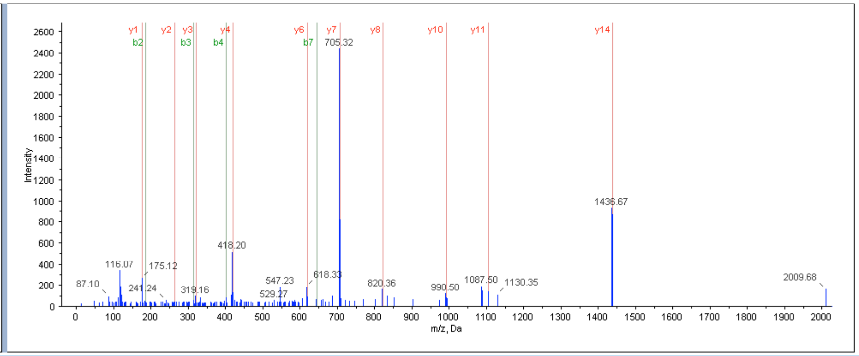

112	1562.843	

Fragmentation Evidence for Peptide

LLLQHDPNADIVSK

Residue	b	y
L	114.0913	1562.8537
L	**227.1754**	1449.7696
L	340.2595	1336.6856
Q	468.3180	1223.6015
H	605.3770	**1095.5429**
D	**720.4039**	**958.4840**
P	817.4567	**843.4571**
N	**931.4996**	746.4043
A	1002.5367	632.3614
D	**1117.5636**	561.3243
I	**1230.6477**	446.2973
V	**1329.7161**	333.2132
S	1416.7482	**234.1448**
K	1544.8431	147.1128

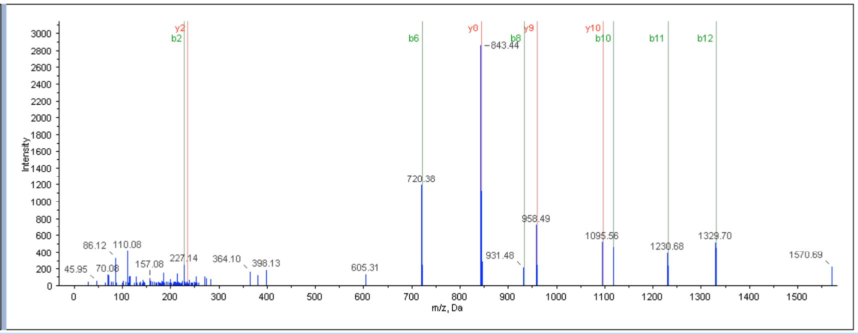

113	1116.584		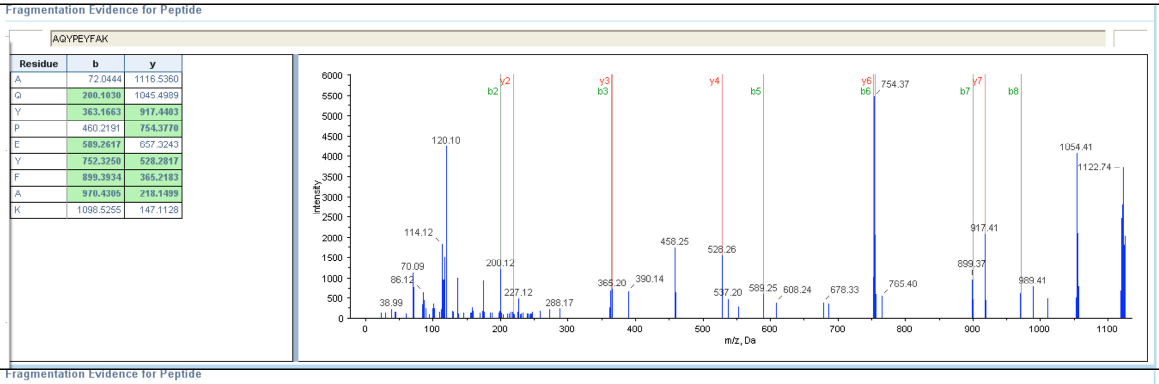

Fragmentation Evidence for Peptide

AQYPEYFAK

Residue	b	y
A	72.0444	1116.5360
Q	**200.1030**	1045.4989
Y	**363.1663**	**917.4403**
P	460.2191	**754.3770**
E	**589.2617**	657.3243
Y	**752.3250**	**528.2817**
F	**899.3934**	**365.2183**
A	**970.4305**	**218.1499**
K	1098.5255	147.1128

114	1687.774		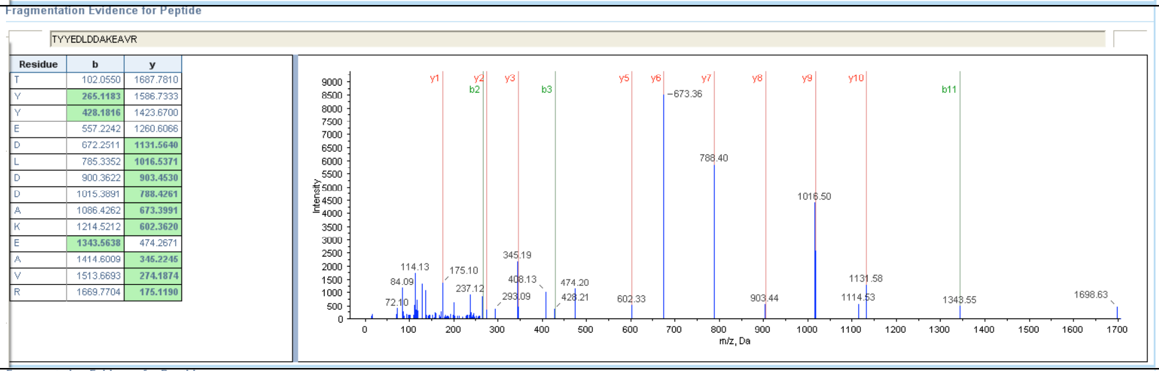

Fragmentation Evidence for Peptide

TYYEDLDDAKEAVR

Residue	b	y
T	102.0550	1687.7810
Y	**265.1183**	1586.7333
Y	**428.1816**	1423.6700
E	557.2242	1260.6066
D	672.2511	**1131.5640**
L	785.3352	**1016.5371**
D	900.3622	**903.4530**
D	1015.3891	**788.4261**
A	1086.4262	**673.3991**
K	1214.5212	**602.3620**
E	**1343.5638**	474.2671
A	1414.6009	**345.2245**
V	1513.6693	**274.1874**
R	1669.7704	**175.1190**

115	1181.608		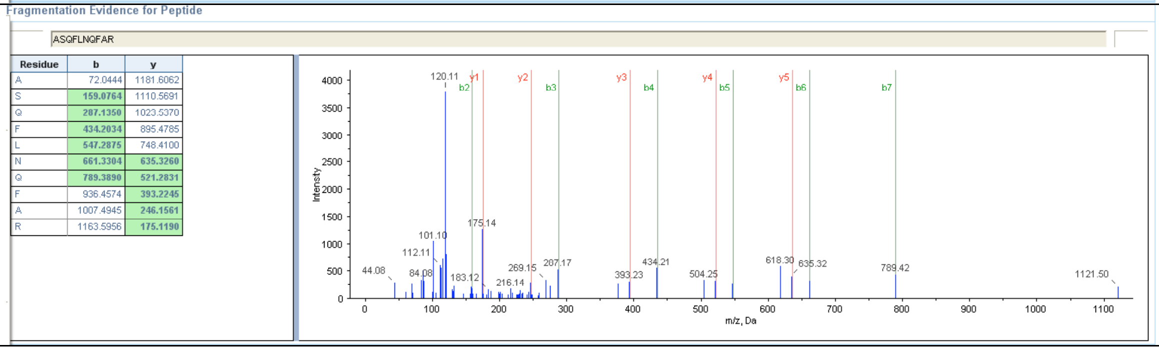

Fragmentation Evidence for Peptide

ASQFLNQFAR

Residue	b	y
A	72.0444	1181.6062
S	**159.0764**	1110.5691
Q	**287.1350**	1023.5370
F	**434.2034**	895.4785
L	**547.2875**	748.4100
N	**661.3304**	**635.3260**
Q	**789.3890**	**521.2831**
F	936.4574	**393.2245**
A	1007.4945	**246.1561**
R	1163.5956	**175.1190**

116	1452.769		Fragmentation Evidence for Peptide

SEAPPAQKFNVHK

Residue	b	y
S	88.0393	1452.7594
E	**217.0819**	1365.7274
A	**288.1190**	**1236.6848**
P	**385.1718**	**1165.6477**
P	**482.2245**	**1068.5949**
A	**553.2617**	**971.5421**
Q	**681.3202**	**900.5050**
K	809.4152	**772.4464**
F	**956.4836**	**644.3515**
N	**1070.5265**	**497.2831**
V	**1169.5949**	383.2401
H	1306.6539	**284.1717**
K	1434.7488	**147.1128**

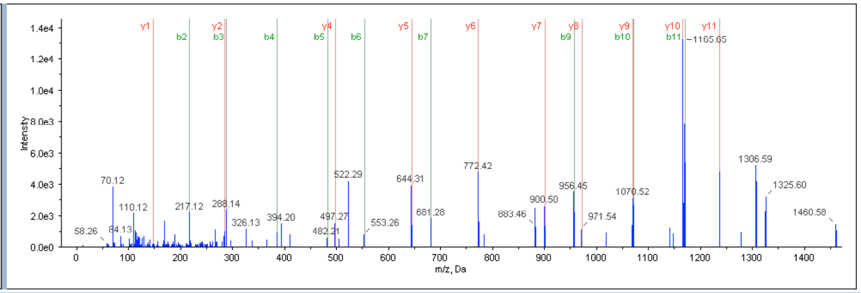

117	1468.744		Fragmentation Evidence for Peptide

GSFQFGKDGLADVK

Residue	b	y
G	58.0287	1468.7431
S	145.0608	1411.7216
F	292.1292	1324.6896
Q	420.1878	1177.6212
F	567.2562	1049.5626
G	624.2776	902.4942
K	752.3726	**845.4727**
D	**867.3995**	717.3777
G	924.4210	**602.3508**
L	1037.5051	545.3293
A	1108.5422	432.2453
D	**1223.5691**	361.2082
V	1322.6375	246.1812
K	1450.7325	147.1128

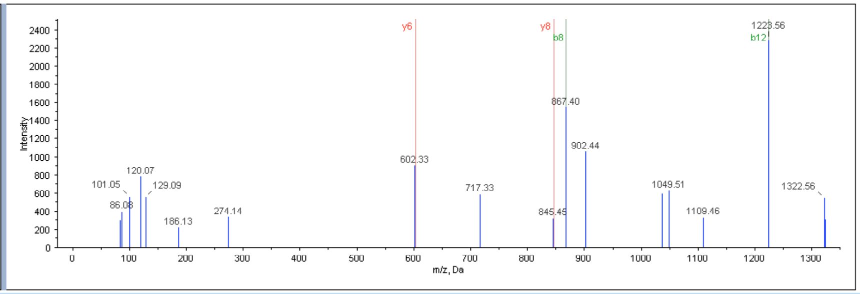

118	1130.494		Fragmentation Evidence for Peptide

HAEIASSFIR

Residue	b	y
H	138.0662	1130.5953
A	**209.1033**	993.5364
E	**338.1459**	922.4993
I	**451.2300**	**793.4567**
A	**522.2671**	680.3726
S	609.2991	609.3355
S	696.3311	**522.3035**
F	843.3995	435.2714
I	**956.4836**	288.2030
R	1112.5847	**175.1190**

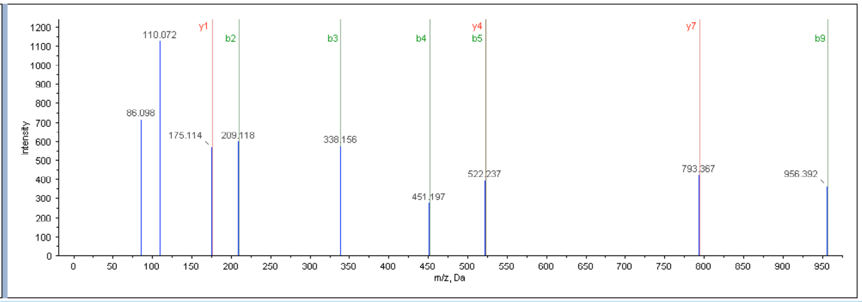

119	3102.404	

Fragmentation Evidence for Peptide

NAPAQKQSSYHGVHQAWNTNQDSKDYQ

Residue	b	y
N	115.0502	3102.4049
A	186.0873	2988.3619
P	283.1401	2917.3248
A	354.1772	2820.2721
Q	482.2358	2749.2349
K	610.3307	2621.1764
Q	738.3893	2493.0814
S	825.4213	2365.0228
S	912.4534	2277.9908
Y	1075.5167	2190.9588
H	1212.5756	**2027.8954**
G	1269.5971	1890.8365
V	**1368.6655**	1833.8151
H	**1505.7244**	1734.7467
Q	1633.7830	1597.6877
A	**1704.8201**	1469.6292
W	1890.8994	1398.5920
N	2004.9423	**1212.5127**
T	2105.9900	1098.4698
N	2220.0329	997.4221
Q	2348.0915	883.3792
D	2463.1185	755.3206
S	2550.1505	640.2937
K	2678.2454	553.2617
D	**2793.2724**	425.1667
Y	2956.3357	310.1397
Q	3084.3943	147.0764

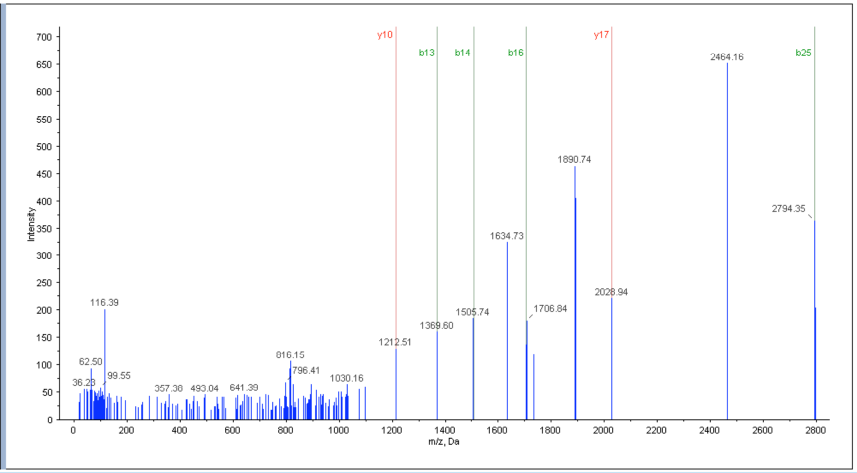

120	2158.935	

Fragmentation Evidence for Peptide

ISHPIYPPVETFGGGKETET

Residue	b	y
I	114.0913	2159.0655
S	201.1234	2045.9815
H	**338.1823**	**1958.9494**
P	435.2350	**1821.8905**
I	**548.3191**	1724.8378
Y	**711.3824**	**1611.7537**
P	808.4352	**1448.6904**
P	905.4880	1351.6376
V	**1004.5564**	1254.5848
E	**1133.5990**	1155.5164
T	1234.6467	1026.4738
F	1381.7151	925.4262
G	1438.7365	778.3577
G	1495.7580	721.3363
G	1552.7795	664.3148
K	1680.8744	607.2933
E	1809.9170	479.1984
T	1910.9647	350.1558
E	2040.0073	249.1081
T	2141.0550	120.0655

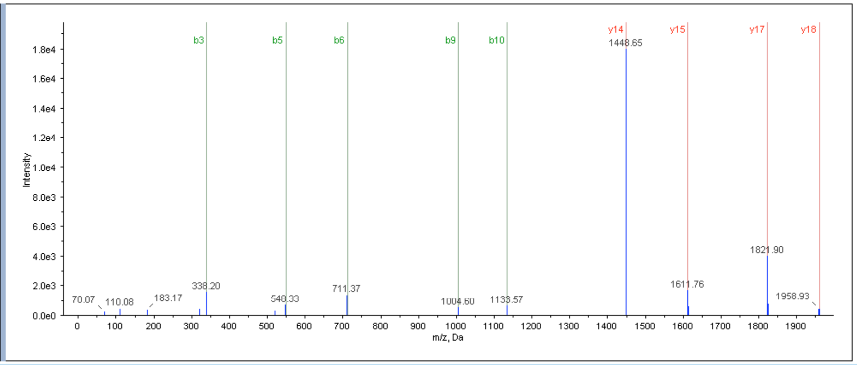

121	2251.964		Fragmentation Evidence for Peptide

FRDEDEDEDQFEEAPRPK

Residue	b	y
F	148.0757	2251.9738
R	304.1768	2104.9054
D	419.2037	1948.8043
E	548.2463	1833.7773
D	**663.2733**	**1704.7347**
E	792.3159	**1589.7078**
D	907.3428	1460.6652
E	1036.3854	**1345.6383**
D	1151.4124	1216.5957
Q	1279.4709	1101.5687
F	1426.5393	**973.5102**
E	1555.5819	826.4417
E	1684.6245	**697.3991**
A	1755.6616	568.3566
P	1852.7144	497.3194
R	2008.8155	400.2667
P	2105.8683	**244.1656**
K	2233.9632	147.1128

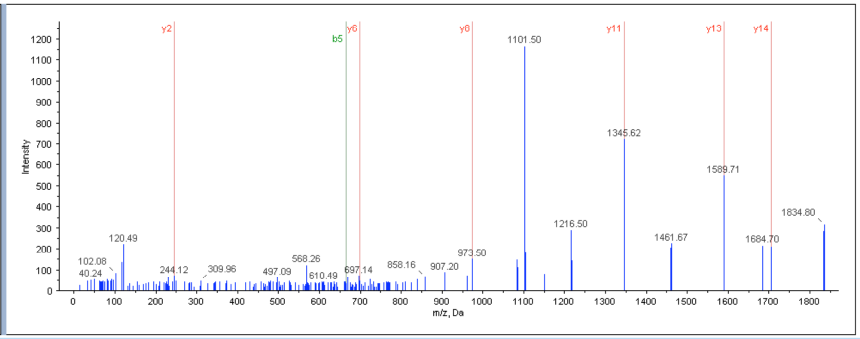

122	1414.762		Fragmentation Evidence for Peptide

VVHSYVGQEPSGR

Residue	b	y
V	100.0757	**1414.7074**
V	**199.1441**	1315.6389
H	**336.2030**	1216.5705
S	**423.2350**	**1079.5116**
Y	**586.2984**	**992.4796**
V	**685.3668**	**829.4163**
G	**742.3883**	**730.3478**
Q	**870.4468**	**673.3264**
E	**999.4894**	**545.2678**
P	1096.5422	**416.2252**
S	1183.5742	319.1724
G	**1240.5957**	**232.1404**
R	1396.6968	**175.1190**

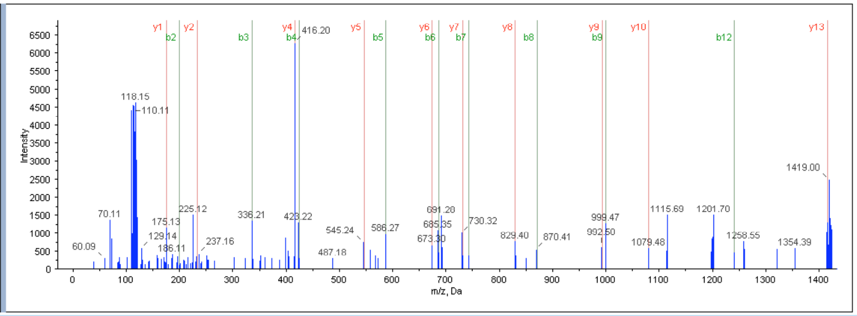

123	1306.579		Fragmentation Evidence for Peptide

GYGYGNQDYNR

Residue	b	y
G	58.0287	1306.5447
Y	**221.0921**	1249.5232
G	**278.1135**	**1086.4599**
Y	441.1769	1029.4384
G	498.1983	**866.3751**
N	**612.2413**	**809.3537**
Q	**740.2998**	**695.3107**
D	**855.3268**	**567.2522**
Y	1018.3901	**452.2252**
N	1132.4330	**289.1619**
R	1288.5341	**175.1190**

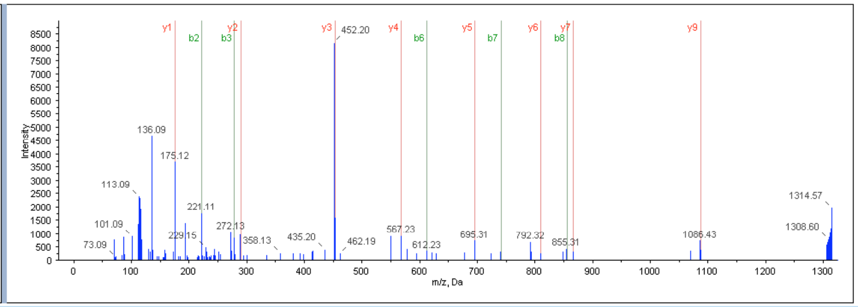

124	1385.776		

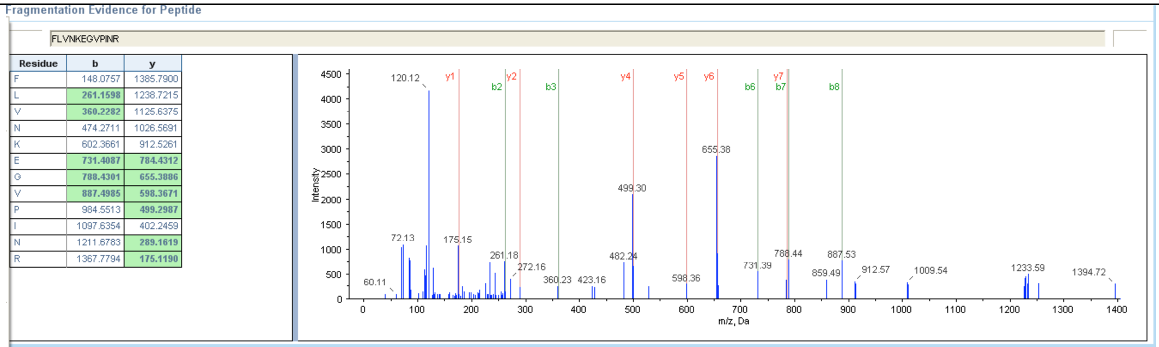

Fragmentation Evidence for Peptide

FLVNKEGVPINR

Residue	b	y
F	148.0757	1385.7900
L	**261.1598**	1238.7215
V	**360.2282**	1125.6375
N	474.2711	1026.5691
K	602.3661	912.5261
E	**731.4087**	**784.4312**
G	**788.4301**	**655.3886**
V	**887.4985**	**598.3671**
P	984.5513	**499.2987**
I	1097.6354	402.2459
N	1211.6783	**289.1619**
R	1367.7794	**175.1190**

125	2457.091		

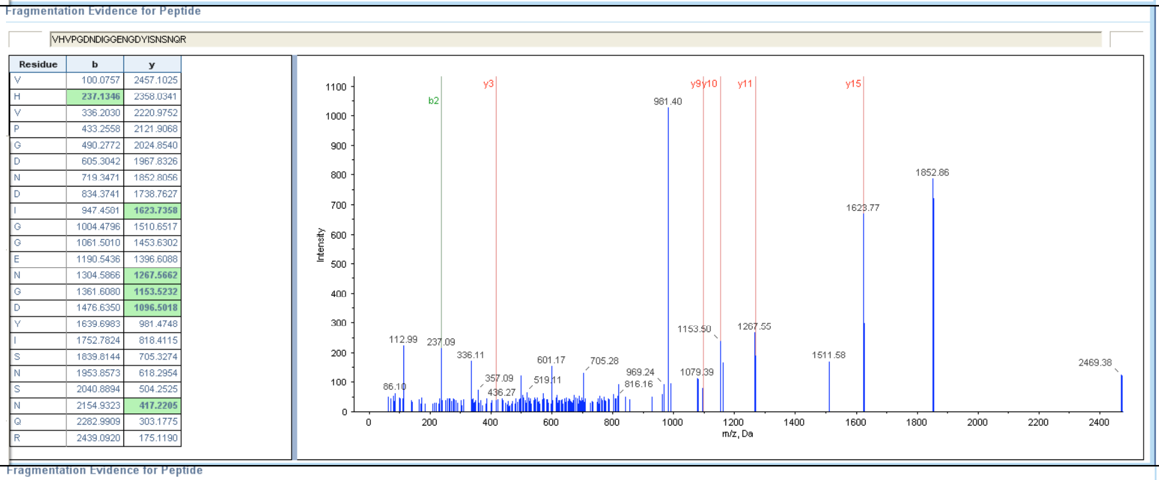

Fragmentation Evidence for Peptide

VHVPGDNDIGGENGDYISNSNQR

Residue	b	y
V	100.0757	2457.1025
H	**237.1346**	2358.0341
V	336.2030	2220.9752
P	433.2558	2121.9068
G	490.2772	2024.8540
D	605.3042	1967.8326
N	719.3471	1852.8056
D	834.3741	1738.7627
I	947.4581	**1623.7358**
G	1004.4796	1510.6517
G	1061.5010	1453.6302
E	1190.5436	1396.6088
N	1304.5866	**1267.5662**
G	1361.6080	**1153.5232**
D	1476.6350	**1096.5018**
Y	1639.6983	981.4748
I	1752.7824	818.4115
S	1839.8144	705.3274
N	1953.8573	618.2954
S	2040.8894	504.2525
N	2154.9323	**417.2205**
Q	2282.9909	303.1775
R	2439.0920	175.1190

126	1535.798		

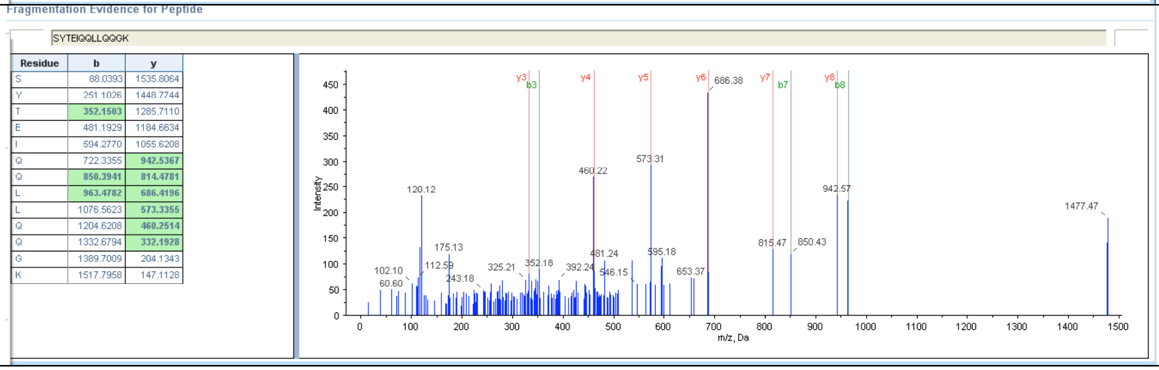

Fragmentation Evidence for Peptide

SYTEIQQLLQQGK

Residue	b	y
S	88.0393	1535.8064
Y	251.1026	1448.7744
T	**352.1503**	1285.7110
E	481.1929	1184.6634
I	594.2770	1055.6208
Q	722.3355	**942.5367**
Q	**850.3941**	**814.4781**
L	**963.4782**	**686.4196**
L	1076.5623	**573.3355**
Q	1204.6208	**460.2514**
Q	1332.6794	**332.1928**
G	1389.7009	204.1343
K	1517.7958	147.1128

127	1813.918	

Fragmentation Evidence for Peptide

GIYGYGFEKPSAIQQR

Residue	b	y
G	58.0287	1813.9232
I	**171.1128**	1756.9017
Y	334.1761	1643.8176
G	391.1976	1480.7543
Y	554.2609	1423.7328
G	611.2824	**1260.6695**
F	758.3508	**1203.6480**
E	887.3934	1056.5796
K	1015.4884	**927.5370**
P	1112.5411	**799.4421**
S	1199.5732	702.3893
A	1270.6103	**615.3573**
I	1383.6943	**544.3202**
Q	1511.7529	**431.2361**
Q	1639.8115	303.1775
R	1795.9126	**175.1190**

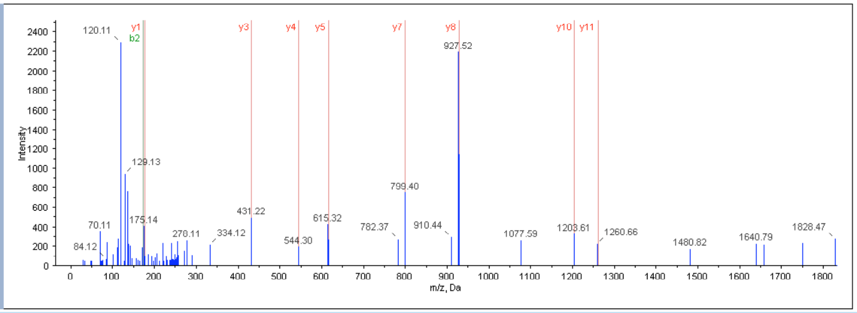

128	2213.087	

Fragmentation Evidence for Peptide

IIKDFMIQGGDFTKGDGTGGR

Residue	b	y
I	**114.0913**	2213.1019
I	227.1754	2100.0179
K	355.2704	1986.9338
D	470.2973	1858.8388
F	617.3657	**1743.8119**
M	748.4062	1596.7435
I	861.4903	1465.7030
Q	989.5489	1352.6189
G	1046.5703	**1224.5604**
G	1103.5918	1167.5389
D	1218.6187	1110.5174
F	1365.6871	995.4905
T	1466.7348	848.4221
K	1594.8298	747.3744
G	1651.8512	619.2794
D	**1766.8782**	562.2580
G	1823.8996	447.2310
T	1924.9473	**390.2096**
G	1981.9688	**289.1619**
G	2038.9903	**232.1404**
R	2195.0914	**175.1190**

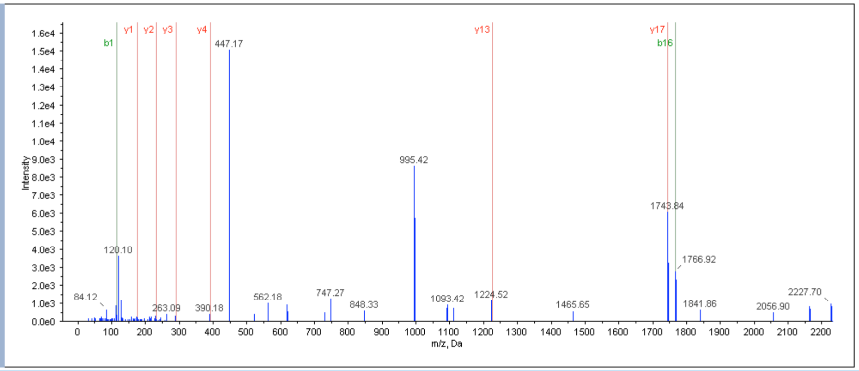

129	1409.802	

Fragmentation Evidence for Peptide

VKVPSGATPIGDLR

Residue	b	y
V	100.0757	1409.8111
K	**228.1707**	1310.7427
V	**327.2391**	**1182.6477**
P	424.2918	**1083.5793**
S	511.3239	986.5265
G	568.3453	**899.4945**
A	639.3824	842.4730
T	**740.4301**	**771.4359**
P	837.4829	**670.3883**
I	950.5669	573.3355
G	1007.5884	460.2514
D	1122.6154	**403.2300**
L	1235.6994	**288.2030**
R	1391.8005	**175.1190**

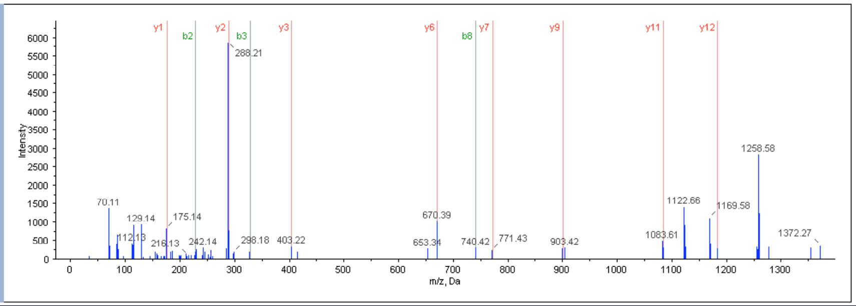

130	1934.886		Fragmentation Evidence for Peptide

TMVADPTKSKEYFETTS

Residue	b	y
T	102.0550	1934.9052
M	**233.0954**	1833.8575
V	**332.1639**	1702.8170
A	403.2010	1603.7486
D	518.2279	1532.7115
P	615.2807	1417.6846
T	716.3284	1320.6318
K	**844.4233**	1219.5841
S	**931.4553**	**1091.4891**
K	**1059.5503**	**1004.4571**
E	**1188.5929**	876.3622
Y	**1351.6562**	747.3196
F	1498.7246	584.2562
E	1627.7672	437.1878
T	**1728.8149**	308.1452
T	1829.8626	207.0975
S	1916.8946	106.0499

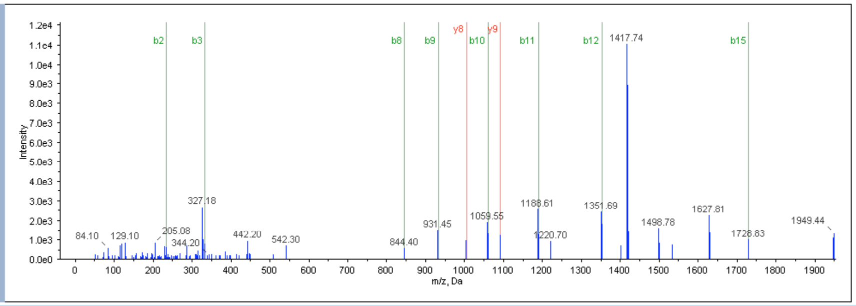

131	2184.095		Fragmentation Evidence for Peptide

ESIVEPNQQKQNWYHQR

Residue	b	y
E	130.0499	2184.0581
S	**217.0819**	2055.0155
I	**330.1660**	1967.9835
V	**429.2344**	1854.8994
E	558.2770	1755.8310
P	655.3297	1626.7884
N	769.3727	1529.7356
Q	897.4312	1415.6927
Q	1025.4898	1287.6341
K	1153.5848	**1159.5756**
Q	1281.6434	**1031.4806**
N	1395.6863	**903.4220**
W	1581.7656	789.3791
Y	1744.8289	603.2998
H	1881.8878	**440.2364**
Q	2009.9464	303.1775
R	2166.0475	**175.1190**

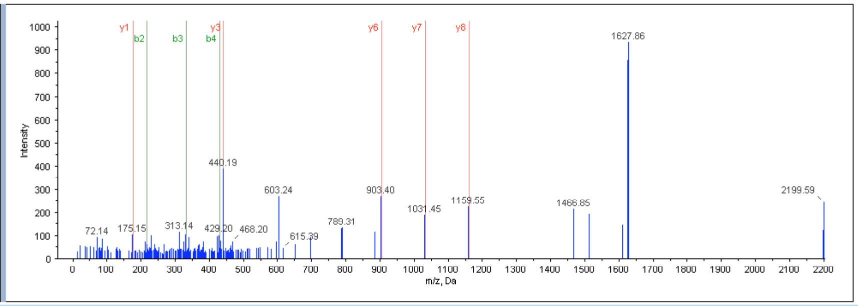

132	1943.854		Fragmentation Evidence for Peptide

TFEADGHDFC[CAM]LAQSFAK

Residue	b	y
T	**102.0550**	1943.8592
F	249.1234	1842.8116
E	378.1660	1695.7431
A	449.2031	1566.7006
D	564.2300	1495.6634
G	621.2515	**1380.6365**
H	758.3104	1323.6150
D	873.3373	**1186.5561**
F	**1020.4058**	**1071.5292**
C[CAM]	**1180.4364**	**924.4608**
L	**1293.5205**	764.4301
A	**1364.5576**	651.3461
Q	**1492.6162**	580.3089
S	1579.6482	452.2504
F	1726.7166	**365.2183**
A	1797.7537	218.1499
K	1925.8487	147.1128

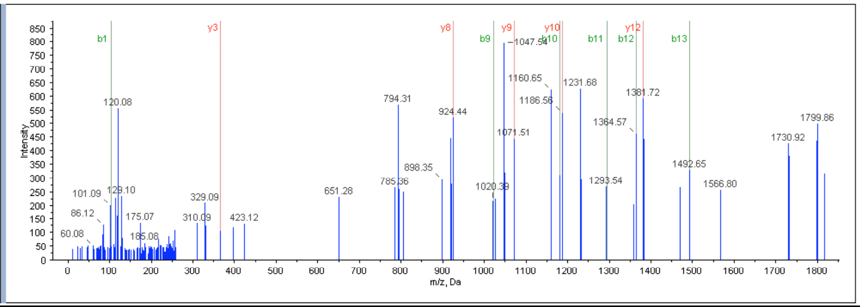

133	1785.812		

Fragmentation Evidence for Peptide

TFEADGHDFC[CAM]LAQSFAK

Residue	b	y
T	**102.0550**	1943.8592
F	249.1234	1842.8116
E	378.1660	1695.7431
A	449.2031	1566.7006
D	564.2300	1495.6634
G	621.2515	**1380.6365**
H	758.3104	1323.6150
D	873.3373	**1186.5561**
F	**1020.4058**	**1071.5292**
C[CAM]	**1180.4364**	**924.4608**
L	**1293.5205**	764.4301
A	**1364.5576**	651.3461
Q	**1492.6162**	580.3089
S	1579.6482	452.2504
F	1726.7166	**365.2183**
A	1797.7537	218.1499
K	1925.8487	147.1128

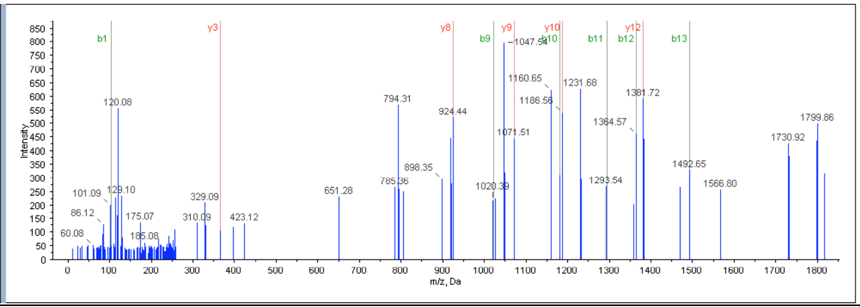

134	2123.932		

Fragmentation Evidence for Peptide

LDNDTANESQGNVQTGSAFR

Residue	b	y
L	114.0913	2123.9588
D	**229.1183**	2010.8748
N	343.1612	**1895.8478**
D	458.1882	1781.8049
T	559.2358	**1666.7780**
A	630.2729	1565.7303
N	744.3159	1494.6932
E	873.3585	1380.6502
S	960.3905	1251.6076
Q	1088.4491	1164.5756
G	1145.4705	1036.5170
N	1259.5135	979.4956
V	**1358.5819**	865.4526
Q	1486.6405	766.3842
T	1587.6881	638.3257
G	1644.7096	**537.2780**
S	1731.7416	480.2565
A	1802.7787	**393.2245**
F	1949.8472	**322.1874**
R	2105.9483	**175.1190**

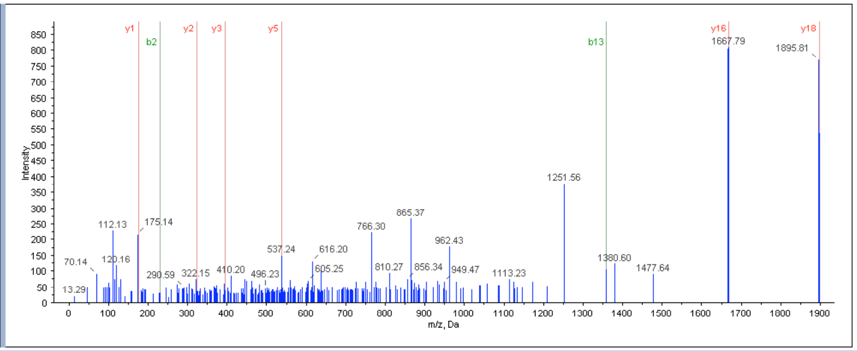

135	1577.766		

Fragmentation Evidence for Peptide

LVQHPNSYFMDVK

Residue	b	y
L	114.0913	1577.7781
V	**213.1598**	1464.6940
Q	**341.2183**	1365.6256
H	**478.2772**	1237.5670
P	575.3300	**1100.5081**
N	**689.3729**	1003.4553
S	776.4050	889.4124
Y	**939.4683**	802.3804
F	**1086.5367**	639.3171
M	1217.5772	**492.2486**
D	**1332.6041**	361.2082
V	**1431.6725**	**246.1812**
K	1559.7675	**147.1128**

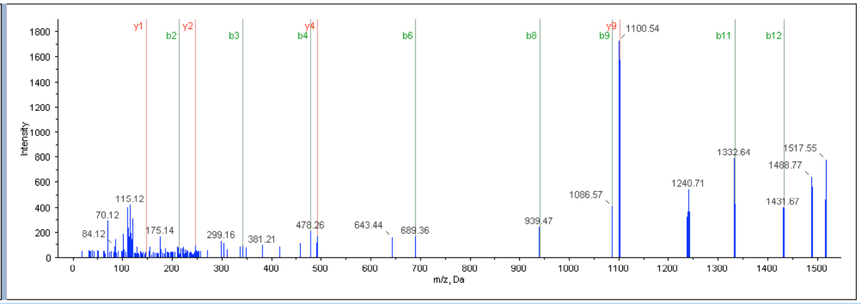

136	1205.632		Fragmentation Evidence for Peptide FVVEKAEQQK 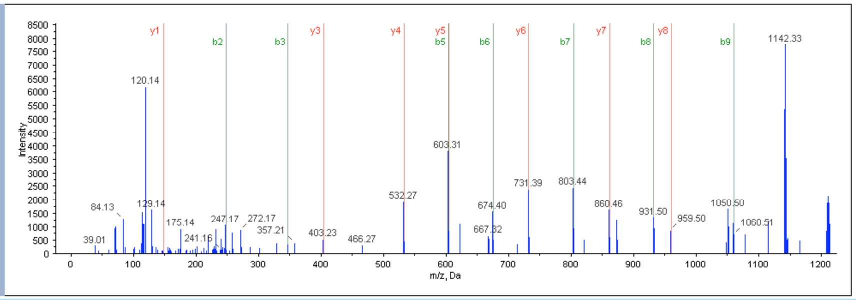
137	1505.741		Fragmentation Evidence for Peptide C[CAM]LVDNGKC[CAM]TPEGR 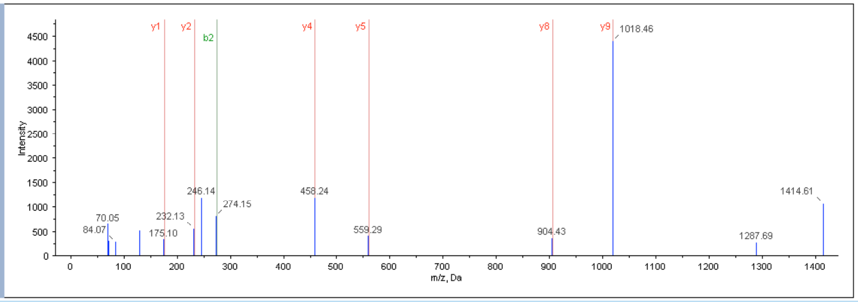
138	1896.966		Fragmentation Evidence for Peptide IVQHEEEANNEKNTEI 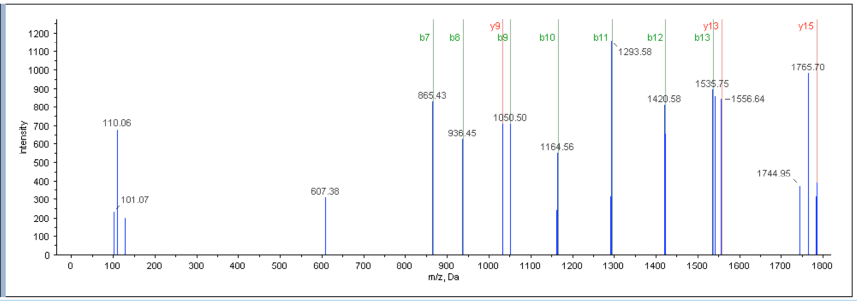

FVVEKAEQQK

Residue	b	y
F	148.0757	1205.6525
V	**247.1441**	1058.5841
V	**346.2125**	**959.5156**
E	475.2551	**860.4472**
K	**603.3501**	**731.4046**
A	**674.3872**	**603.3097**
E	**803.4298**	**532.2726**
Q	**931.4884**	**403.2300**
Q	**1059.5469**	275.1714
K	1187.6419	**147.1128**

C[CAM]LVDNGKC[CAM]TPEGR

Residue	b	y
C[CAM]	161.0379	1505.6835
L	**274.1220**	1345.6529
V	373.1904	1232.5688
D	488.2173	1133.5004
N	602.2603	**1018.4735**
G	659.2817	**904.4305**
K	787.3767	847.4091
C[CAM]	947.4073	719.3141
T	1048.4550	**559.2835**
P	1145.5078	**458.2358**
E	1274.5504	361.1830
G	1331.5718	**232.1404**
R	1487.6730	**175.1190**

IVQHEEEANNEKNTEI

Residue	b	y
I	114.0913	1896.8934
V	213.1598	**1783.8093**
Q	341.2183	1684.7409
H	478.2772	**1556.6823**
E	607.3198	1419.6234
E	736.3624	1290.5808
E	**865.4050**	1161.5382
A	**936.4421**	**1032.4956**
N	**1050.4851**	961.4585
N	**1164.5280**	847.4156
E	**1293.5706**	733.3727
K	**1421.6655**	604.3301
N	**1535.7085**	476.2351
T	1636.7562	362.1922
E	1765.7987	261.1445
I	1878.8828	132.1019

139	1568.803	Fragmentation Evidence for Peptide: KTNNEQVTSFTTAK

Fragmentation Evidence for Peptide

KTNNEQVTSFTTAK

Residue	b	y
K	**129.1022**	1568.7915
T	**230.1499**	1440.6965
N	344.1928	**1339.6488**
N	458.2358	**1225.6059**
E	**587.2784**	**1111.5630**
Q	715.3369	**982.5204**
V	814.4054	854.4618
T	915.4530	**755.3934**
S	**1002.4851**	654.3457
F	**1149.5535**	567.3137
T	**1250.6012**	420.2453
T	**1351.6488**	319.1976
A	1422.6859	**218.1499**
K	1550.7809	**147.1128**

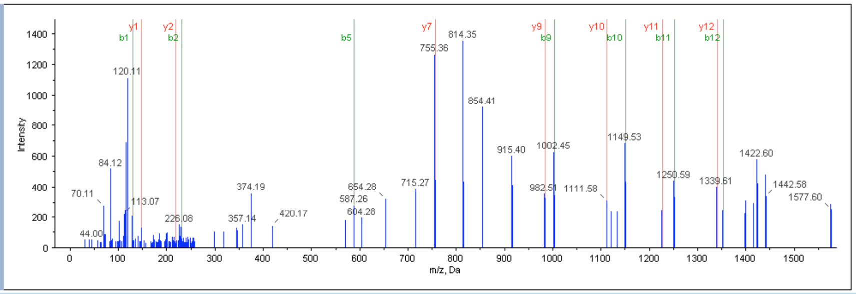

140	1641.732	Fragmentation Evidence for Peptide: SANGN[Dea]VAFENPTYTR

Fragmentation Evidence for Peptide

SANGN[Dea]VAFENPTYTR

Residue	b	y
S	88.0393	1641.7503
A	**159.0764**	1554.7183
N	273.1193	**1483.6812**
G	330.1408	**1369.6383**
N[Dea]	445.1678	1312.6168
V	**544.2362**	1197.5899
A	**615.2733**	1098.5214
F	762.3417	**1027.4843**
E	891.3843	880.4159
N	1005.4272	**751.3733**
P	1102.4800	**637.3304**
T	1203.5277	540.2776
Y	1366.5910	**439.2300**
T	1467.6387	**276.1666**
R	1623.7398	**175.1190**

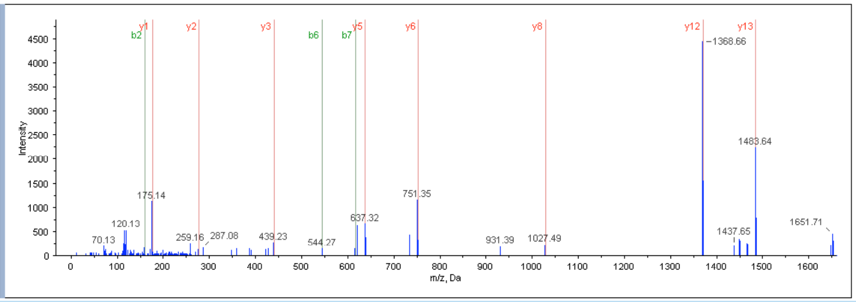

141	2488.081	Fragmentation Evidence for Peptide: NGDIASRIEEEEEEEDEAPAQK

Fragmentation Evidence for Peptide

NGDIASRIEEEEEEEDEAPAQK

Residue	b	y
N	115.0502	2488.0958
G	**172.0717**	2374.0528
D	**287.0986**	2317.0314
I	**400.1827**	**2202.0044**
A	471.2198	2088.9204
S	558.2518	2017.8833
R	714.3529	1930.8512
I	827.4370	1774.7501
E	956.4796	1661.6661
E	1085.5222	1532.6235
E	1214.5648	1403.5809
E	1343.6074	1274.5383
E	1472.6500	1145.4957
E	1601.6926	1016.4531
E	1730.7351	887.4105
D	1845.7621	758.3679
E	1974.8047	643.3410
A	2045.8418	514.2984
P	2142.8946	443.2613
A	2213.9317	346.2085
Q	2341.9902	**275.1714**
K	2470.0852	147.1128

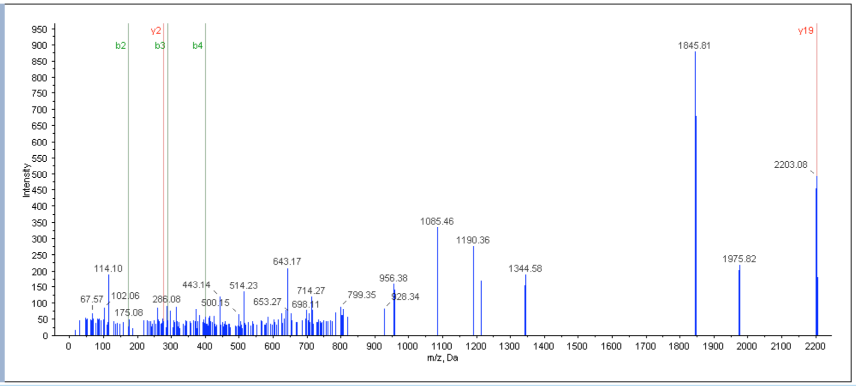

142	1618.792		Fragmentation Evidence for Peptide

Fragmentation Evidence for Peptide

EGEVPGVDYHFITR

Residue	b	y
E	**130.0499**	1618.7860
G	**187.0713**	**1489.7434**
E	**316.1139**	1432.7219
V	415.1823	**1303.6793**
P	512.2351	**1204.6109**
G	569.2566	1107.5582
V	668.3250	1050.5367
D	783.3519	951.4683
Y	946.4153	**836.4413**
H	1083.4742	**673.3780**
F	1230.5426	536.3191
I	1343.6266	**389.2507**
T	**1444.6743**	**276.1666**
R	1600.7754	**175.1190**

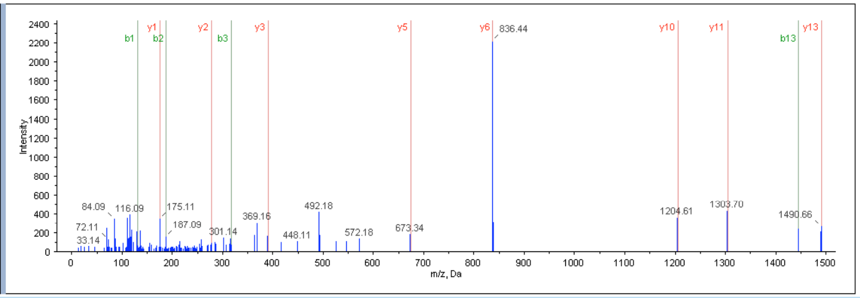

143	1763.848		Fragmentation Evidence for Peptide

Fragmentation Evidence for Peptide

ITQKHPEYTGPTLGPK

Residue	b	y
I	114.0913	1766.9436
T	**215.1390**	1653.8595
Q	343.1976	1552.8118
K	**471.2926**	1424.7532
H	**608.3515**	1296.6583
P	**705.4042**	**1159.5994**
E	834.4468	1062.5466
Y	**997.5102**	933.5040
T	**1098.5578**	770.4407
G	1155.5793	**669.3930**
P	1252.6321	612.3715
T	1353.6797	515.3188
L	1466.7638	**414.2711**
G	1523.7853	**301.1870**
P	1620.8380	**244.1656**
K	1748.9330	147.1128

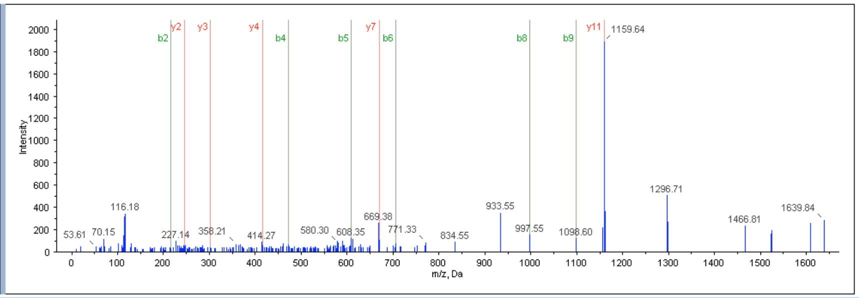

144	1573.742		Fragmentation Evidence for Peptide

Fragmentation Evidence for Peptide

FSEQEFEKNFLR

Residue	b	y
F	**148.0757**	1573.7645
S	**235.1077**	1426.6961
E	**364.1503**	1339.6641
Q	**492.2089**	**1210.6215**
E	**621.2515**	1082.5629
F	768.3199	**953.5203**
E	897.3625	806.4519
K	1025.4575	**677.4093**
N	**1139.5004**	**549.3144**
F	1286.5688	**435.2714**
L	1399.6529	**288.2030**
R	1555.7540	**175.1190**

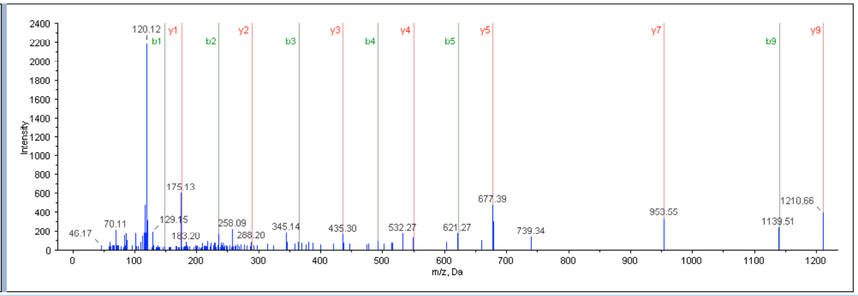

145	1496.718		Fragmentation Evidence for Peptide (see below)

Fragmentation Evidence for Peptide

SSGIIHFNYYAPK

Residue	b	y
S	88.0393	1496.7532
S	**175.0713**	1409.7212
G	232.0928	**1322.6892**
I	**345.1769**	1265.6677
I	**458.2609**	1152.5837
H	**595.3198**	**1039.4996**
F	**742.3883**	**902.4407**
N	**856.4312**	**755.3723**
Y	**1019.4945**	**641.3293**
Y	1182.5578	478.2660
A	1253.5949	**315.2027**
P	1350.6477	**244.1656**
K	1478.7427	**147.1128**

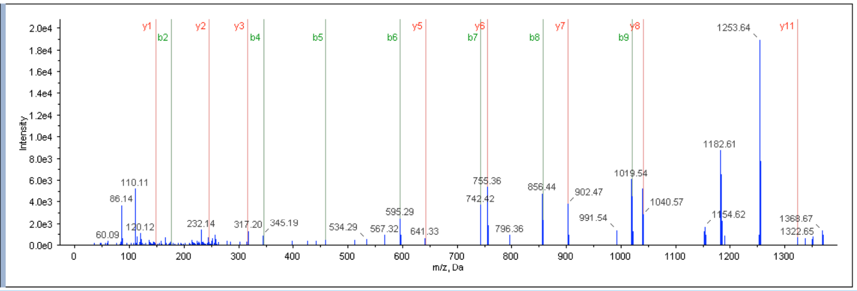

146	1669.918		Fragmentation Evidence for Peptide (see below)

Fragmentation Evidence for Peptide

LVPKGPFN[Dea]ITIDNIK

Residue	b	y
L	114.0913	1669.9523
V	**213.1598**	1556.8683
P	310.2125	**1457.7999**
K	438.3075	1360.7471
G	495.3289	1232.6521
P	592.3817	1175.6307
F	**739.4501**	1078.5779
N[Dea]	854.4771	931.5095
I	967.5611	816.4825
T	1068.6088	703.3985
I	**1181.6929**	**602.3508**
D	**1296.7198**	**489.2667**
N	**1410.7627**	**374.2398**
I	**1523.8468**	260.1969
K	1651.9418	147.1128

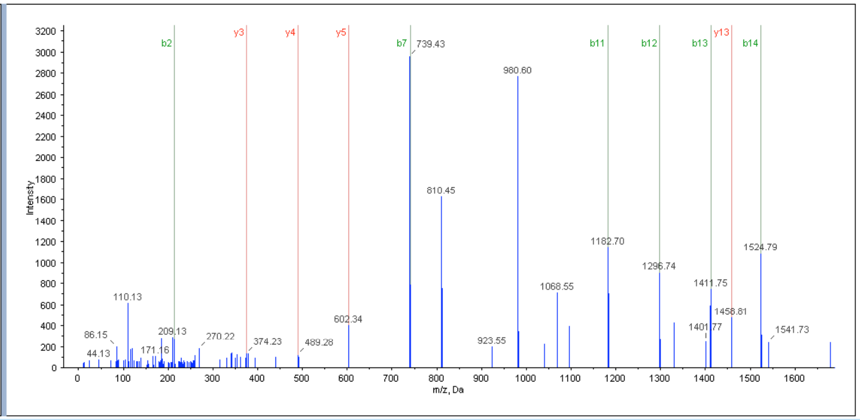

147	2214.931	

Fragmentation Evidence for Peptide

TGGEDFTQQFTENDFEGHR

Residue	b	y
T	**102.0550**	2214.9323
G	**159.0764**	2113.8846
G	216.0979	2056.8631
E	345.1405	1999.8417
D	**460.1674**	**1870.7991**
F	607.2358	**1755.7721**
T	708.2835	1608.7037
Q	836.3421	**1507.6560**
Q	964.4007	1379.5975
F	1111.4691	1251.5389
T	1212.5168	1104.4705
E	1341.5594	1003.4228
N	1455.6023	874.3802
D	1570.6292	760.3373
F	1717.6976	645.3103
E	1846.7402	498.2419
G	1903.7617	369.1993
H	**2040.8206**	312.1779
R	2196.9217	**175.1190**

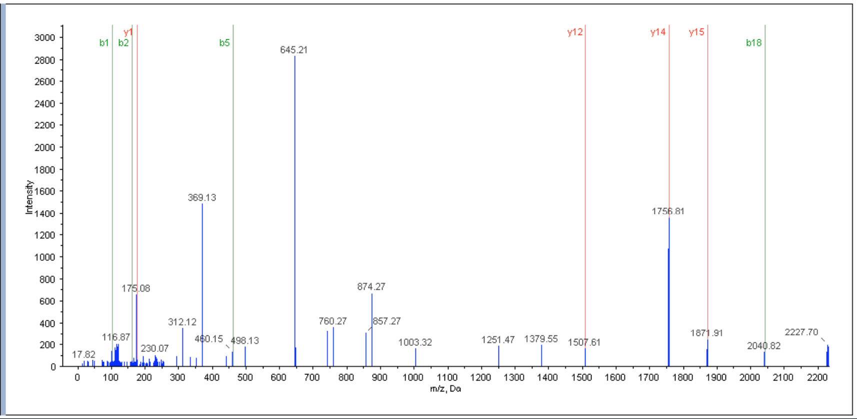

148	1389.684	

Fragmentation Evidence for Peptide

SQINFPIGGATER

Residue	b	y
S	88.0393	1389.7121
Q	**216.0979**	1302.6801
I	**329.1819**	**1174.6215**
N	**443.2249**	1061.5374
F	590.2933	**947.4945**
P	**687.3461**	**800.4261**
I	**800.4301**	703.3733
G	857.4516	**590.2893**
G	914.4730	533.2678
A	**985.5102**	476.2463
T	1086.5578	**405.2092**
E	1215.6004	**304.1615**
R	1371.7015	**175.1190**

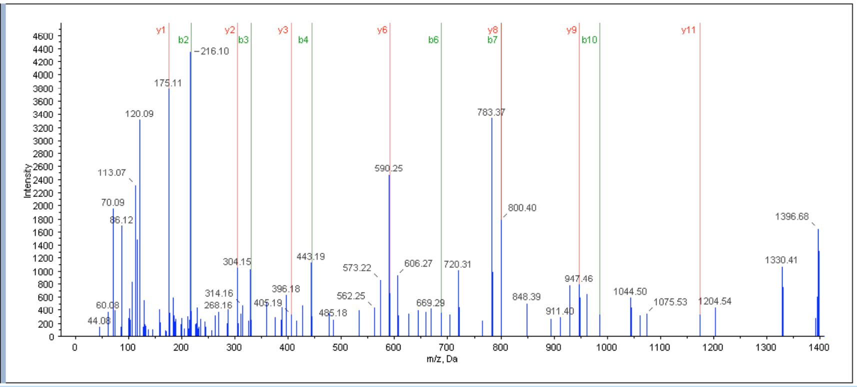

149	1454.649	Fragmentation Evidence for Peptide

Fragmentation Evidence for Peptide

SLEPEEGFSFGQK

Residue	b	y
S	88.0393	1454.6798
L	201.1234	1367.6478
E	330.1660	1254.5637
P	427.2187	**1125.5211**
E	556.2613	1028.4684
E	685.3039	**899.4258**
G	742.3254	**770.3832**
F	889.3938	**713.3617**
S	976.4258	**566.2933**
F	1123.4942	**479.2613**
G	1180.5157	332.1928
Q	1308.5743	275.1714
K	1436.6692	147.1128

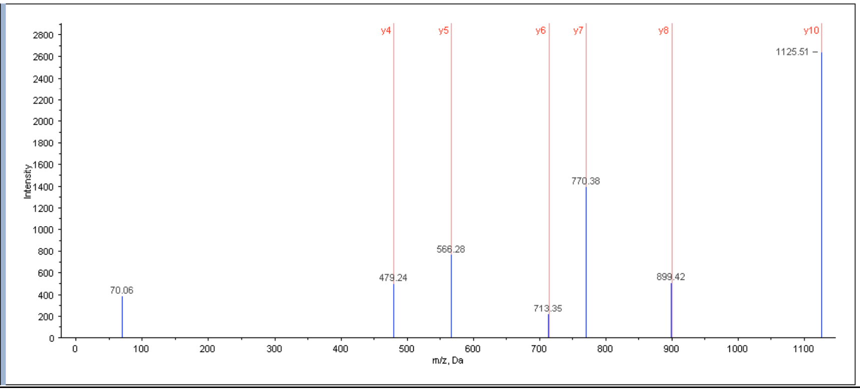

150	1523.763	Fragmentation Evidence for Peptide

Fragmentation Evidence for Peptide

FLDGEMKTIGKER

Residue	b	y
F	148.0757	1523.7886
L	**261.1598**	1376.7202
D	376.1867	1263.6362
G	433.2082	1148.6092
E	562.2508	1091.5878
M	693.2912	**962.5452**
K	821.3862	**831.5047**
T	**922.4339**	**703.4097**
I	1035.5179	**602.3620**
G	1092.5394	**489.2780**
K	1220.6344	432.2565
E	1349.6770	**304.1615**
R	1505.7781	**175.1190**

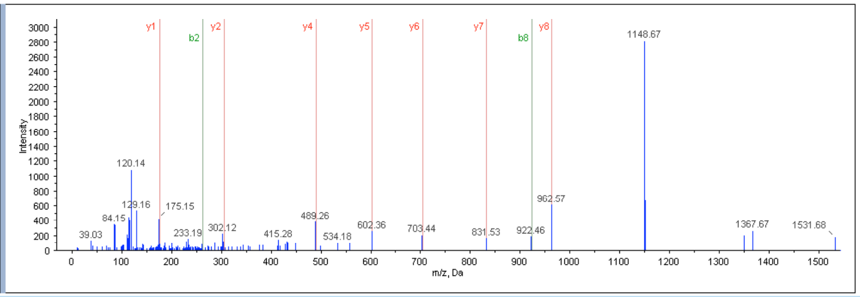

151	2706.405	

Fragmentation Evidence for Peptide

GAAPGPQQQQHLPGQPAGAAAPVPPAHR

Residue	b	y
G	58.0287	2706.3971
A	129.0659	2649.3757
A	200.1030	2578.3386
P	297.1557	2507.3014
G	354.1772	2410.2487
P	451.2300	2353.2272
Q	579.2885	2256.1745
Q	707.3471	2128.1159
Q	**835.4057**	2000.0573
Q	963.4643	1871.9987
H	1100.5232	**1743.9401**
L	1213.6072	1606.8812
P	**1310.6600**	**1493.7972**
G	1367.6815	1396.7444
Q	**1495.7401**	1339.7229
P	1592.7928	1211.6644
A	1663.8299	1114.6116
G	1720.8514	1043.5745
A	1791.8885	**986.5530**
A	1862.9256	**915.5159**
A	1933.9627	844.4788
P	2031.0155	773.4417
V	2130.0839	676.3889
P	2227.1367	577.3205
P	2324.1894	480.2677
A	2395.2266	**383.2150**
H	2532.2855	312.1779
R	2688.3866	**175.1190**

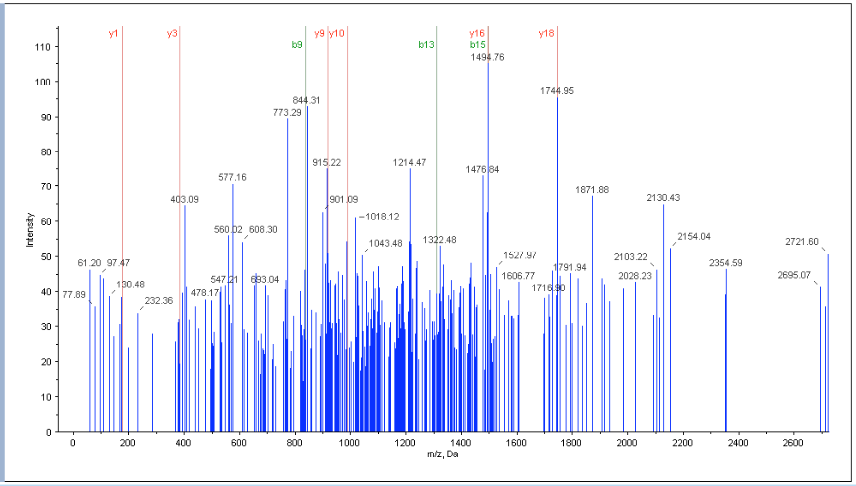

152	1007.573	

Fragmentation Evidence for Peptide

TLEAHALPR

Residue	b	y
T	102.0550	1007.5633
L	215.1390	906.5156
E	344.1816	793.4315
A	415.2187	**664.3889**
H	**552.2776**	**593.3518**
A	**623.3148**	**456.2929**
L	**736.3988**	**385.2558**
P	833.4516	**272.1717**
R	989.5527	**175.1190**

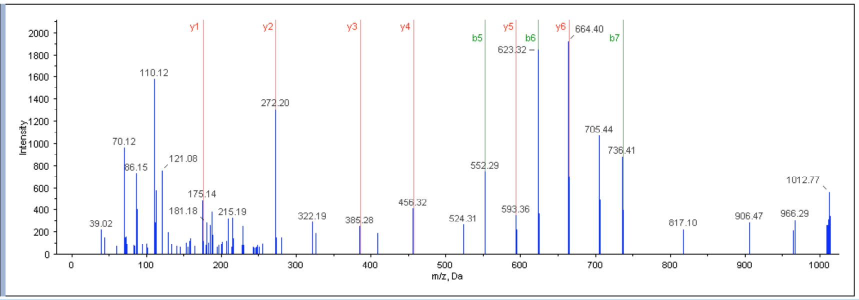

153	1205.583	

Fragmentation Evidence for Peptide

Q[Dea]TLGLDMELGK

Residue	b	y
Q[Dea]	130.0499	1205.6082
T	231.0975	1076.5656
L	344.1816	975.5179
G	401.2031	862.4339
L	514.2871	805.4124
D	**629.3141**	**692.3284**
M	**760.3546**	**577.3014**
E	**889.3972**	**446.2609**
L	**1002.4812**	317.2183
G	**1059.5027**	204.1343
K	1187.5977	**147.1128**

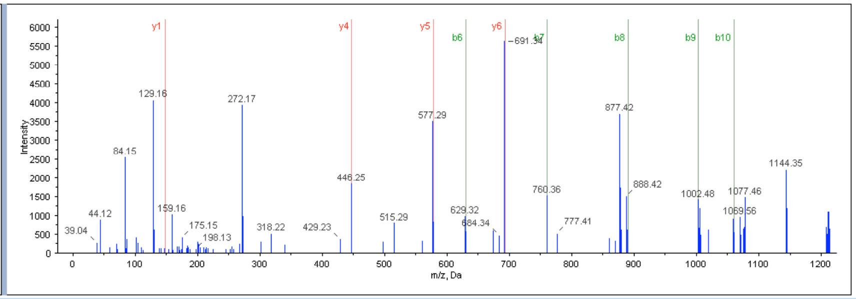

154	1312.653

Fragmentation Evidence for Peptide

SNFIEVYTPSR

Residue	b	y
S	88.0393	1312.6532
N	202.0822	1225.6212
F	349.1506	1111.5782
I	462.2347	964.5098
E	591.2773	851.4258
V	690.3457	722.3832
Y	853.4090	623.3148
T	954.4567	460.2514
P	1051.5095	359.2037
S	1138.5415	262.1510
R	1294.6426	175.1190

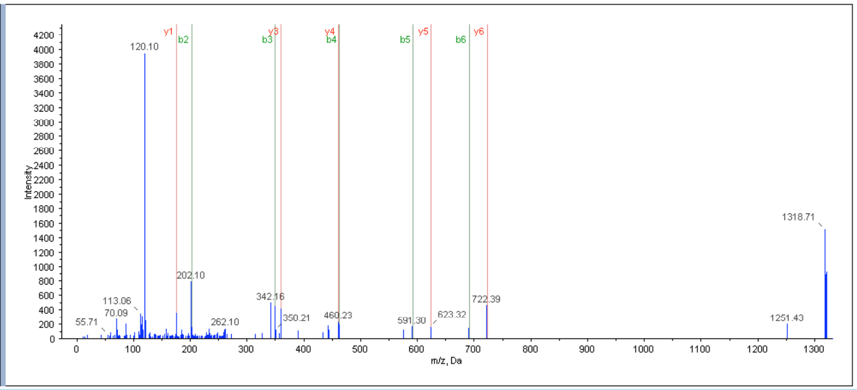

155	1577.838

Fragmentation Evidence for Peptide

LLKDHPNTYTFTK

Residue	b	y
L	114.0913	1577.8322
L	227.1754	1464.7482
K	355.2704	1351.6641
D	470.2973	1223.5691
H	607.3562	1108.5422
P	704.4090	971.4833
N	818.4519	874.4305
T	919.4996	760.3876
Y	1082.5629	659.3399
T	1183.6106	496.2766
F	1330.6790	395.2289
T	1431.7267	248.1605
K	1559.8217	147.1128

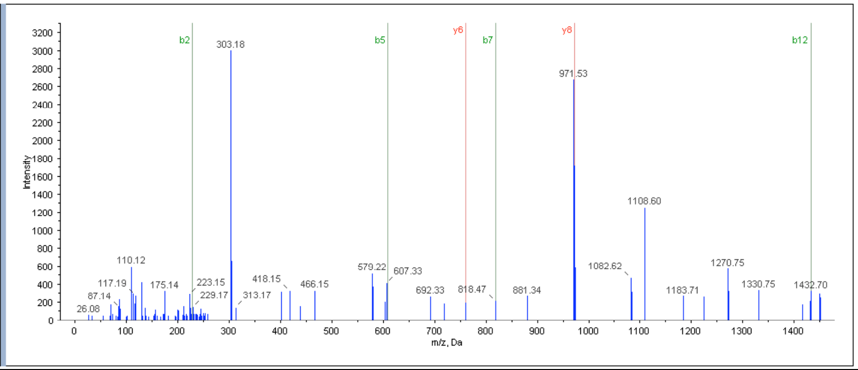

156	1442.723		Fragmentation Evidence for Peptide

NTATEFSYILQR

Residue	b	y
N	115.0502	1442.7274
T	**216.0979**	1328.6845
A	**287.1350**	1227.6368
T	**388.1827**	1156.5997
E	**517.2253**	1055.5520
F	**664.2937**	926.5094
S	**751.3257**	**779.4410**
Y	914.3890	**692.4090**
I	1027.4731	**529.3457**
L	1140.5572	**416.2616**
Q	1268.6157	**303.1775**
R	1424.7169	**175.1190**

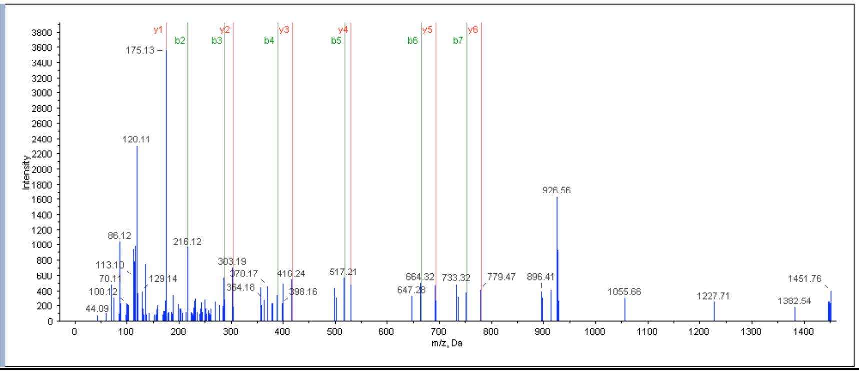

157	2169.132		Fragmentation Evidence for Peptide

TTRLEQGEKPPSPANFQLR

Residue	b	y
T	**102.0550**	2169.1411
T	203.1026	2068.0934
R	359.2037	1967.0457
L	**472.2878**	1810.9446
E	601.3304	1697.8606
Q	729.3890	**1568.8180**
G	786.4104	**1440.7594**
E	915.4530	1383.7379
K	1043.5480	**1254.6953**
P	1140.6008	**1126.6004**
P	1237.6535	1029.5476
S	1324.6856	932.4948
P	1421.7383	845.4628
A	1492.7754	748.4100
N	1606.8184	677.3729
F	1753.8868	563.3300
Q	1881.9454	416.2616
L	1995.0294	288.2030
R	2151.1305	**175.1190**

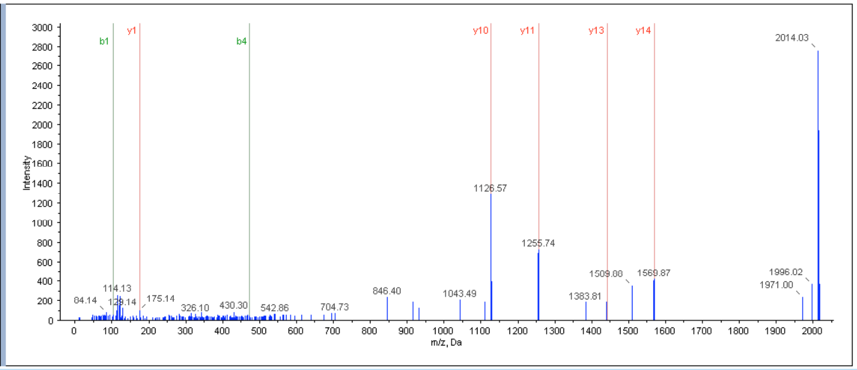

158	1627.887	Fragmentation Evidence for Peptide: NALKWQKPIDTDAK

Fragmentation Evidence for Peptide

NALKWQKPIDTDAK

Residue	b	y
N	115.0502	1627.8802
A	**186.0873**	1513.8373
L	**299.1714**	1442.8002
K	427.2663	**1329.7161**
W	**613.3457**	1201.6212
Q	741.4042	1015.5419
K	**869.4992**	**887.4833**
P	966.5520	**759.3883**
I	1079.6360	662.3355
D	1194.6630	549.2515
T	1295.7106	**434.2245**
D	**1410.7376**	**333.1769**
A	1481.7747	218.1499
K	1609.8697	147.1128

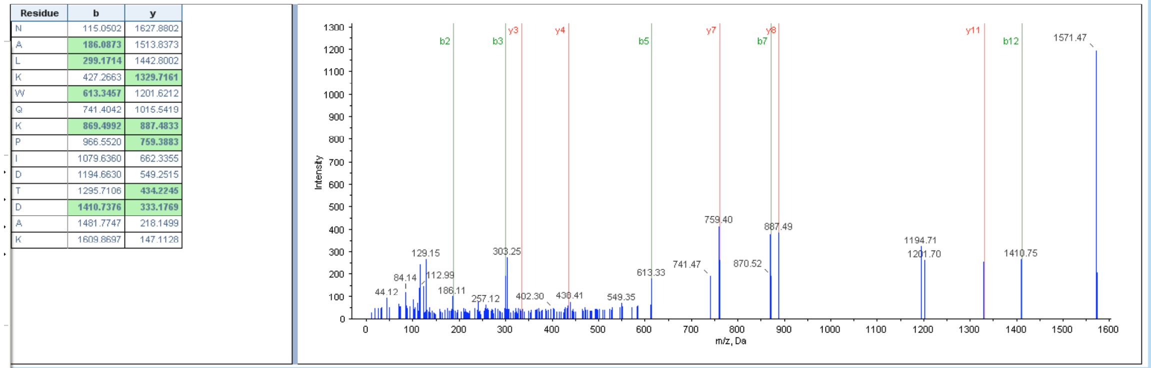

159	2247.311	Fragmentation Evidence for Peptide: AAAPAAAAPAPAAAAPAVAKPAPKPK

Fragmentation Evidence for Peptide

AAAPAAAAPAPAAAAPAVAKPAPKPK

Residue	b	y
A	72.0444	2247.2972
A	143.0815	2176.2601
A	214.1186	2105.2230
P	**311.1714**	2034.1859
A	382.2085	1937.1331
A	**453.2456**	1866.0960
A	**524.2827**	**1795.0589**
A	595.3198	**1724.0218**
P	692.3726	1652.9846
A	763.4097	**1555.9319**
P	**860.4625**	**1484.8948**
A	931.4996	1387.8420
A	1002.5367	1316.8049
A	1073.5738	**1245.7678**
A	1144.6109	1174.7307
P	1241.6637	1103.6935
A	1312.7008	1006.6408
V	1411.7692	935.6037
A	1482.8063	836.5352
K	1610.9013	765.4981
P	1707.9541	637.4032
A	1778.9912	540.3504
P	1876.0439	469.3133
K	2004.1389	**372.2605**
P	2101.1917	**244.1656**
K	2229.2866	**147.1128**

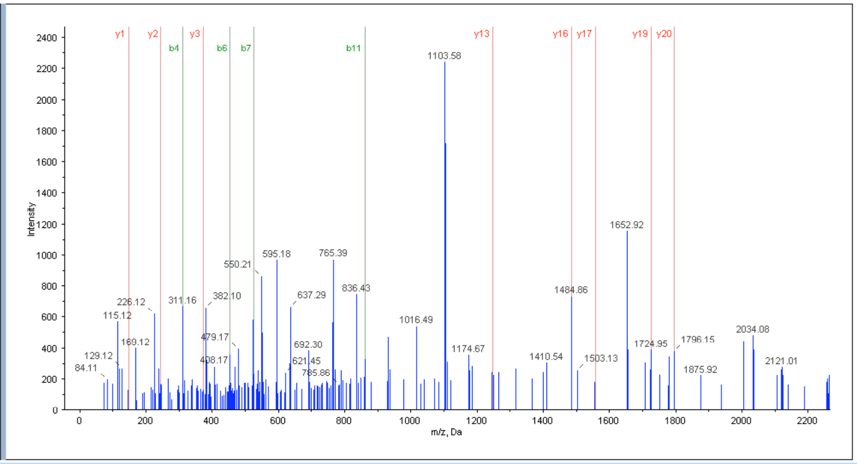

160	1226.574	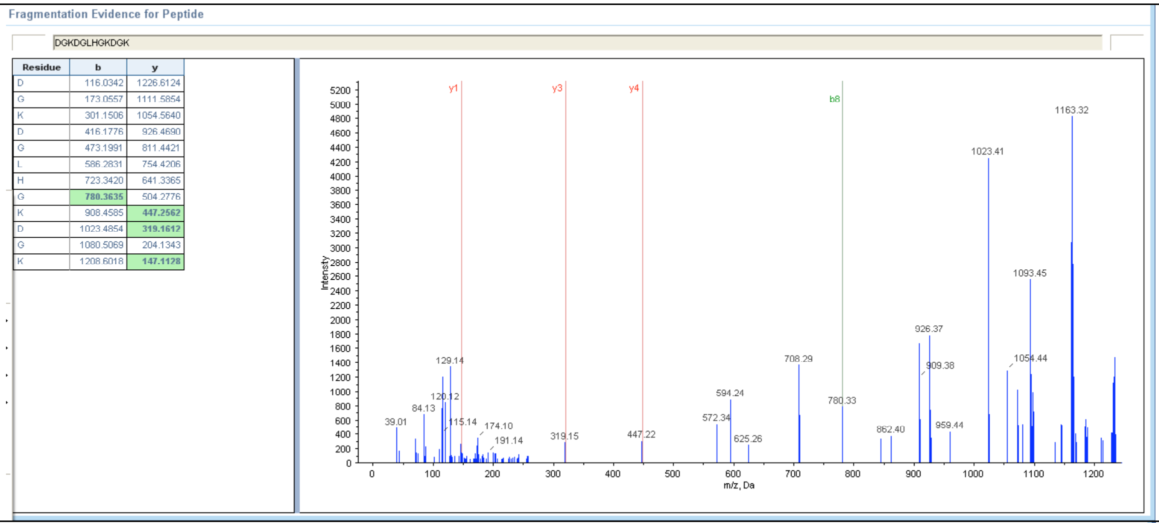

Residue	b	y
D	116.0342	1226.6124
G	173.0557	1111.5854
K	301.1506	1054.5640
D	416.1776	926.4690
G	473.1991	811.4421
L	586.2831	754.4206
H	723.3420	641.3365
G	**780.3635**	504.2776
K	908.4585	**447.2562**
D	1023.4854	**319.1612**
G	1080.5069	204.1343
K	1208.6018	**147.1128**

161	2242.103	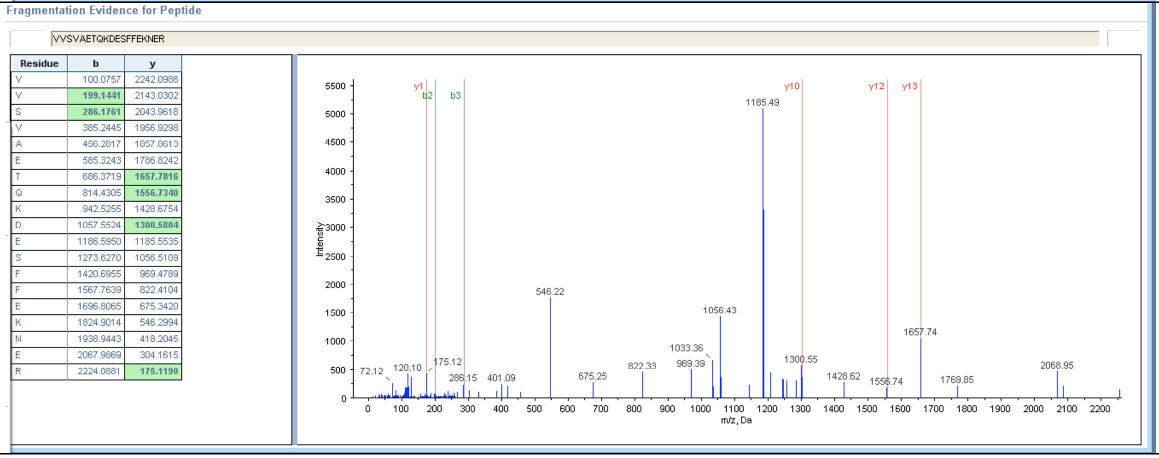

Residue	b	y
V	100.0757	2242.0986
V	**199.1441**	2143.0302
S	**286.1761**	2043.9618
V	385.2445	1956.9298
A	456.2817	1857.8613
E	585.3243	1786.8242
T	686.3719	**1657.7816**
Q	814.4305	**1556.7340**
K	942.5255	1428.6754
D	1057.5524	**1300.5804**
E	1186.5950	1185.5535
S	1273.6270	1056.5109
F	1420.6955	969.4789
F	1567.7639	822.4104
E	1696.8065	675.3420
K	1824.9014	546.2994
N	1938.9443	418.2045
E	2067.9869	304.1615
R	2224.0881	**175.1190**

162	1168.584	

Fragmentation Evidence for Peptide

YGIIDNFNGR

Residue	b	y
Y	164.0706	1168.5745
G	221.0921	1005.5112
I	334.1761	948.4898
I	447.2602	835.4057
D	562.2871	722.3216
N	676.3301	607.2947
F	823.3985	493.2518
N	937.4414	346.1833
G	994.4629	232.1404
R	1150.5640	175.1190

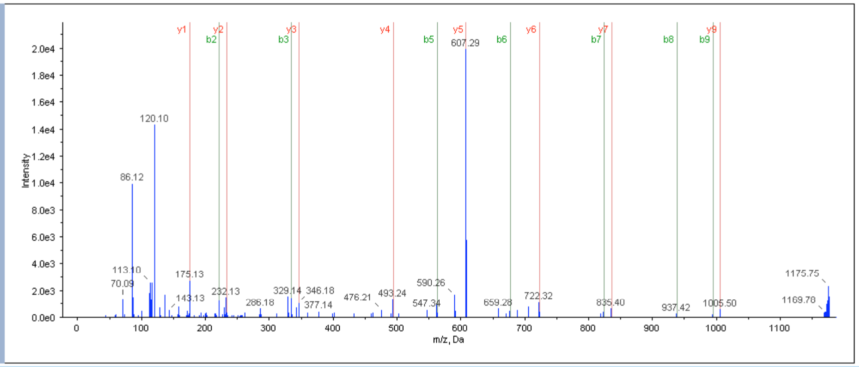

163	1359.682	

Fragmentation Evidence for Peptide

NPFQDKLVNER

Residue	b	y
N	115.0502	1359.7015
P	212.1030	1245.6586
F	359.1714	1148.6058
Q	487.2300	1001.5374
D	602.2569	873.4789
K	730.3519	758.4519
L	843.4359	630.3570
V	942.5043	517.2729
N	1056.5473	418.2045
E	1185.5899	304.1615
R	1341.6910	175.1190

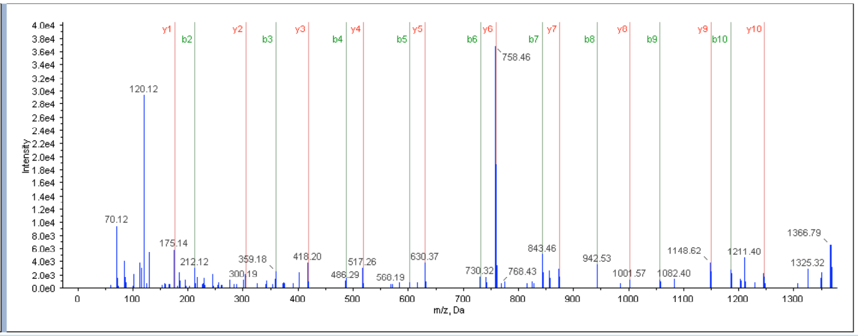

164	1712.828	

Fragmentation Evidence for Peptide

NAGGPASHTISGPNFSAK

Residue	b	y
N	115.0502	1712.8351
A	186.0873	1598.7921
G	243.1088	1527.7550
G	300.1302	1470.7336
P	397.1830	1413.7121
A	468.2201	1316.6593
S	555.2522	1245.6222
H	692.3111	1158.5902
T	793.3587	1021.5313
I	906.4428	920.4836
S	993.4748	807.3995
G	1050.4963	720.3675
P	1147.5491	663.3461
N	1261.5920	566.2933
F	1408.6604	452.2504
S	1495.6924	305.1819
A	1566.7295	218.1499
K	1694.8245	147.1128

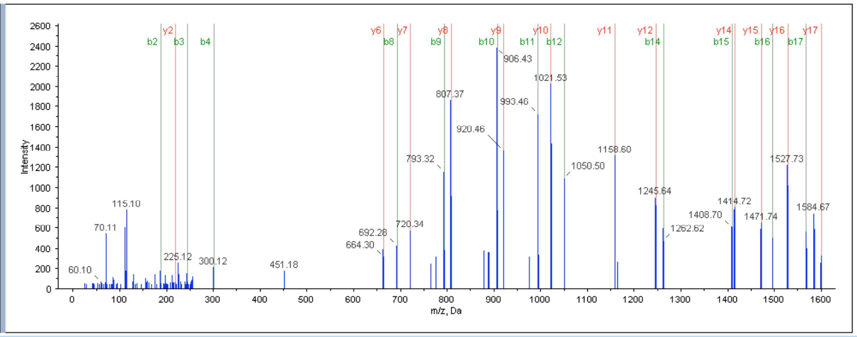

165	1666.766	
166	1135.724	
167	936.4918	

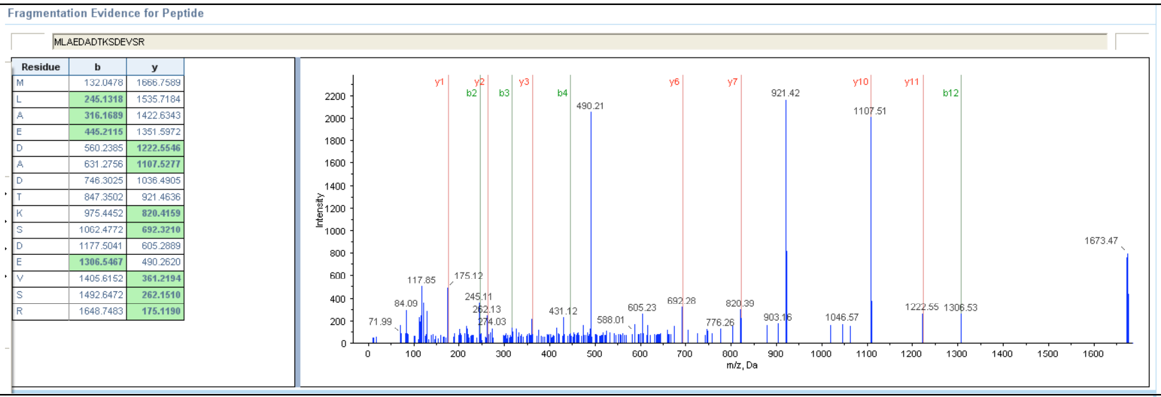

Residue	b	y
M	132.0478	1666.7589
L	**245.1318**	1535.7184
A	**316.1689**	1422.6343
E	**445.2115**	1351.5972
D	560.2385	**1222.5546**
A	631.2756	**1107.5277**
D	746.3025	1036.4905
T	847.3502	921.4636
K	975.4452	**820.4159**
S	1062.4772	**692.3210**
D	1177.5041	605.2889
E	**1306.5467**	490.2620
V	1405.6152	**361.2194**
S	1492.6472	**262.1510**
R	1648.7483	**175.1190**

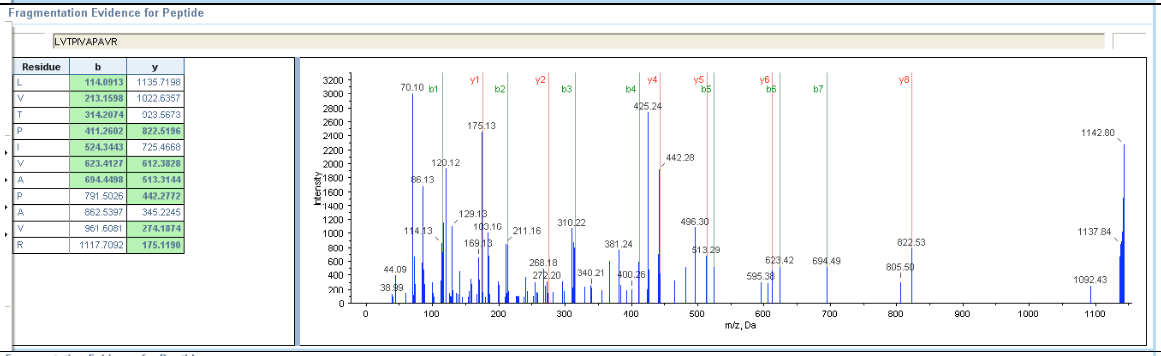

Residue	b	y
L	**114.0913**	1135.7198
V	**213.1598**	1022.6357
T	**314.2074**	923.5673
P	**411.2602**	**822.5196**
I	**524.3443**	725.4668
V	**623.4127**	**612.3828**
A	**694.4498**	**513.3144**
P	791.5026	**442.2772**
A	862.5397	345.2245
V	961.6081	**274.1874**
R	1117.7092	**175.1190**

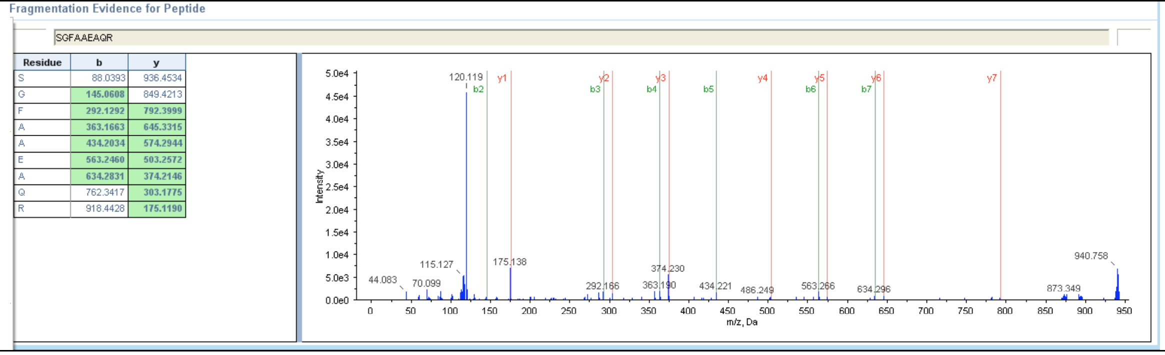

Residue	b	y
S	88.0393	936.4534
G	**145.0608**	849.4213
F	**292.1292**	**792.3999**
A	**363.1663**	**645.3315**
A	**434.2034**	**574.2944**
E	**563.2460**	**503.2572**
A	**634.2831**	**374.2146**
Q	762.3417	**303.1775**
R	918.4428	**175.1190**

168	1771.876		Fragmentation Evidence for Peptide (see below)

Fragmentation Evidence for Peptide

C[CAM]THYGAPLQTGALGLGR

Residue	b	y
C[CAM]	161.0379	1771.8908
T	**262.0856**	1611.8602
H	**399.1445**	1510.8125
Y	562.2078	1373.7536
G	619.2293	**1210.6902**
A	690.2664	1153.6688
P	787.3192	**1082.6317**
L	900.4032	985.5789
Q	1028.4618	872.4948
T	1129.5095	744.4363
G	1186.5310	**643.3886**
A	1257.5681	586.3671
L	1370.6521	515.3300
G	1427.6736	402.2459
L	1540.7577	**345.2245**
G	1597.7791	**232.1404**
R	1753.8802	**175.1190**

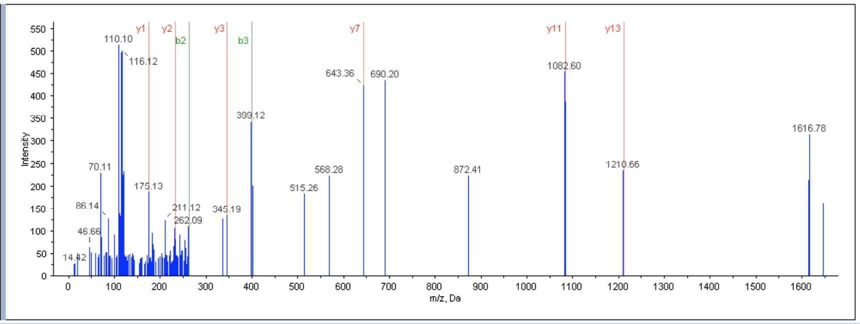

169	927.5477		Fragmentation Evidence for Peptide (see below)

Fragmentation Evidence for Peptide

DVSPLIQR

Residue	b	y
D	116.0342	927.5258
V	215.1026	**812.4989**
S	302.1347	713.4304
P	399.1874	626.3984
L	512.2715	529.3457
I	625.3556	416.2616
Q	753.4141	303.1775
R	909.5152	175.1190

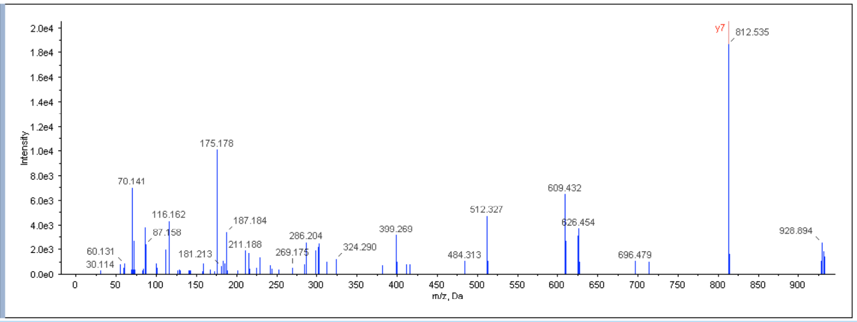

170	1117.621		Fragmentation Evidence for Peptide (see below)

Fragmentation Evidence for Peptide

QADAAPLLYR

Residue	b	y
Q	129.0659	1117.6000
A	**200.1030**	989.5415
D	**315.1299**	**918.5043**
A	**386.1670**	**803.4774**
A	**457.2041**	732.4403
P	554.2569	**661.4032**
L	**667.3410**	564.3504
L	780.4250	**451.2663**
Y	943.4884	338.1823
R	1099.5895	**175.1190**

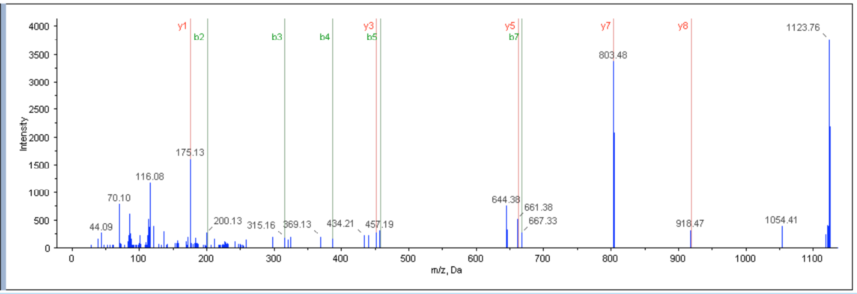

171	1222.611	Fragmentation Evidence for Peptide: WYDNVITVGR

WYDNVITVGR

Residue	b	y
W	187.0866	1222.6215
Y	350.1499	1036.5422
D	465.1769	873.4789
N	579.2198	758.4519
V	678.2882	644.4090
I	791.3723	545.3406
T	892.4199	432.2565
V	991.4884	331.2088
G	1048.5098	232.1404
R	1204.6109	175.1190

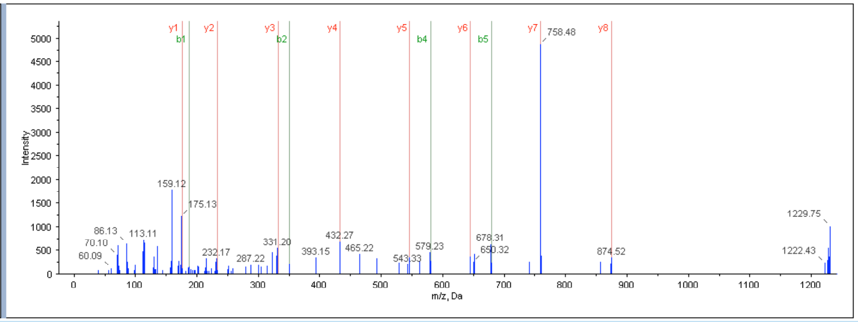

172	1230.572	Fragmentation Evidence for Peptide: YVADKEDVYE

YVADKEDVYE

Residue	b	y
Y	164.0706	1230.5525
V	263.1390	1067.4891
A	334.1761	968.4207
D	449.2031	897.3836
K	577.2980	782.3567
E	706.3406	654.2617
D	821.3676	525.2191
V	920.4360	410.1922
Y	1083.4993	311.1238
E	1212.5419	148.0604

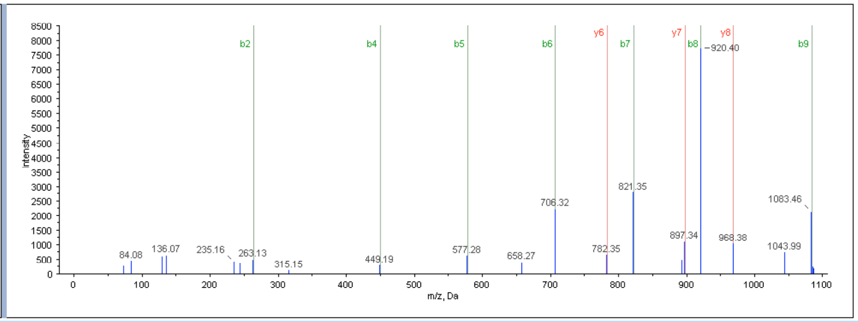

173	1106.568	Fragmentation Evidence for Peptide: QAMLFLQQK

QAMLFLQQK

Residue	b	y
Q	129.0659	1106.6027
A	200.1030	978.5441
M	331.1435	907.5070
L	444.2275	776.4665
F	591.2959	663.3824
L	704.3800	516.3140
Q	832.4386	403.2300
Q	960.4971	275.1714
K	1088.5921	147.1128

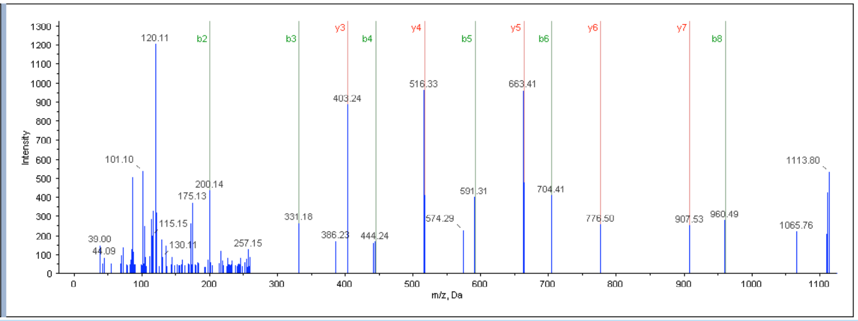

174	1484.715	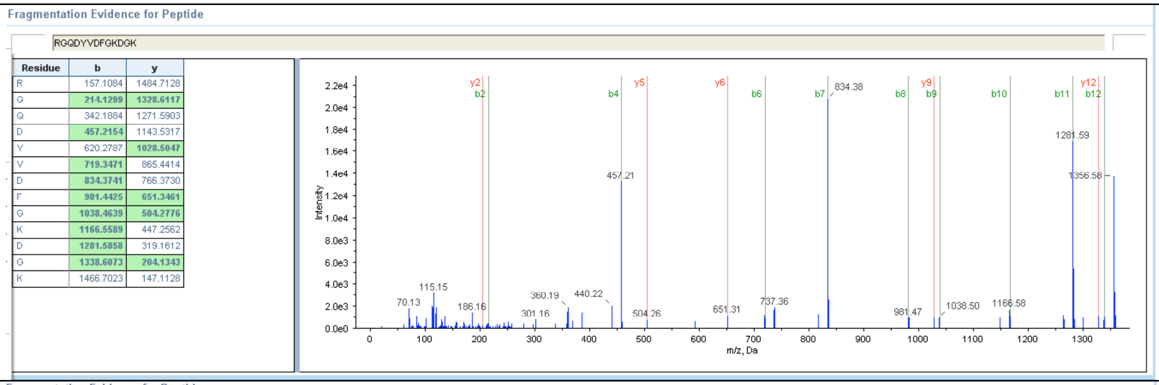
175	1519.846	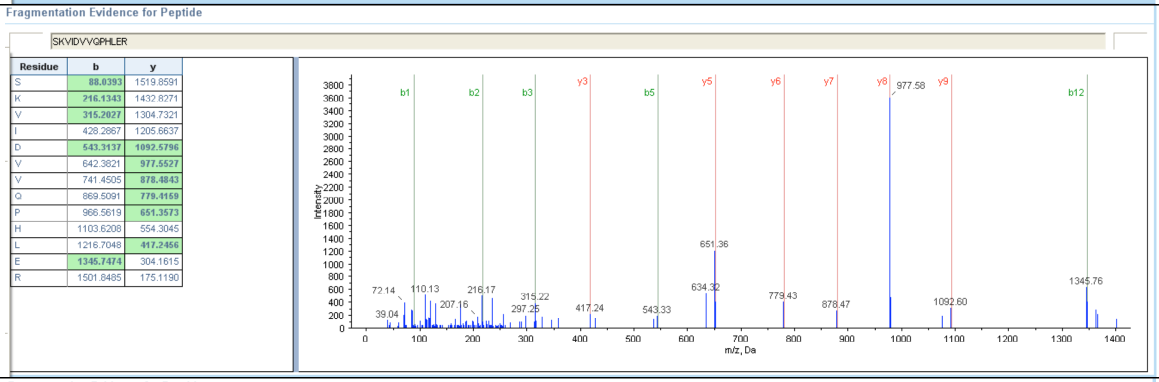
176	1740.779	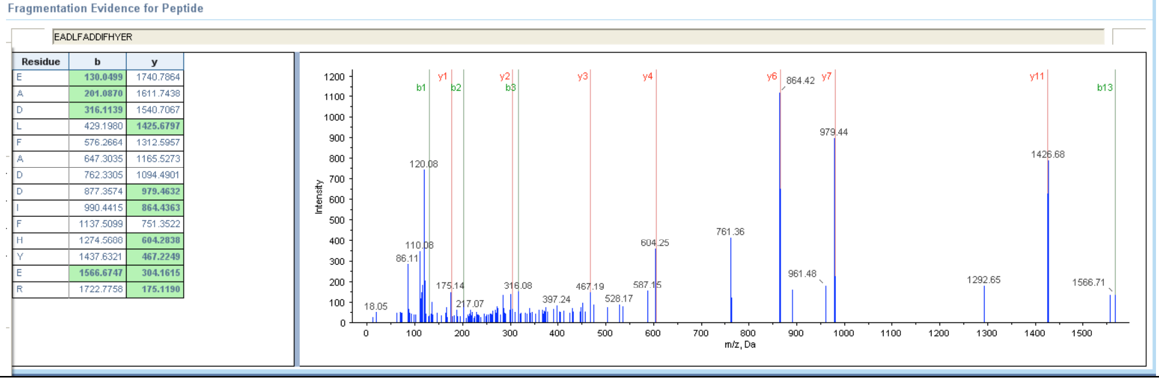

Residue	b	y
R	157.1084	1484.7128
G	**214.1299**	**1328.6117**
Q	342.1884	1271.5903
D	**457.2154**	1143.5317
Y	620.2787	**1028.5047**
V	**719.3471**	865.4414
D	**834.3741**	766.3730
F	**981.4425**	**651.3461**
G	**1038.4639**	**504.2776**
K	**1166.5589**	447.2562
D	**1281.5858**	319.1612
G	**1338.6073**	**204.1343**
K	1466.7023	147.1128

Residue	b	y
S	**88.0393**	1519.8591
K	**216.1343**	1432.8271
V	**315.2027**	1304.7321
I	428.2867	1205.6637
D	**543.3137**	**1092.5796**
V	642.3821	**977.5527**
V	741.4505	**878.4843**
Q	869.5091	**779.4159**
P	966.5619	**651.3573**
H	1103.6208	554.3045
L	1216.7048	**417.2456**
E	**1345.7474**	304.1615
R	1501.8485	175.1190

Residue	b	y
E	**130.0499**	1740.7864
A	**201.0870**	1611.7438
D	**316.1139**	1540.7067
L	429.1980	**1425.6797**
F	576.2664	1312.5957
A	647.3035	1165.5273
D	762.3305	1094.4901
D	877.3574	**979.4632**
I	990.4415	**864.4363**
F	1137.5099	751.3522
H	1274.5688	**604.2838**
Y	1437.6321	**467.2249**
E	**1566.6747**	**304.1615**
R	1722.7758	**175.1190**

177	1596.807	Fragmentation Evidence for Peptide

INHEPQYQIVGGNK

Residue	b	y
I	114.0913	1596.8129
N	228.1343	1483.7288
H	365.1932	1369.6859
E	494.2358	1232.6270
P	591.2885	1103.5844
Q	719.3471	1006.5316
Y	882.4104	878.4730
Q	1010.4690	715.4097
I	1123.5531	587.3511
V	1222.6215	474.2671
G	1279.6430	375.1987
G	1336.6644	318.1772
N	1450.7074	261.1557
K	1578.8023	147.1128

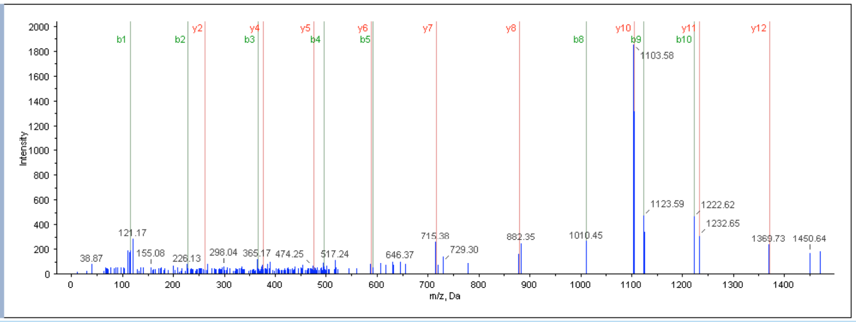

178	1081.564	Fragmentation Evidence for Peptide

SVEPHLLMR

Residue	b	y
S	88.0393	1081.5823
V	187.1077	994.5502
E	316.1503	895.4818
P	413.2031	766.4392
H	550.2620	669.3865
L	663.3461	532.3276
L	776.4301	419.2435
M	907.4706	306.1594
R	1063.5717	175.1190

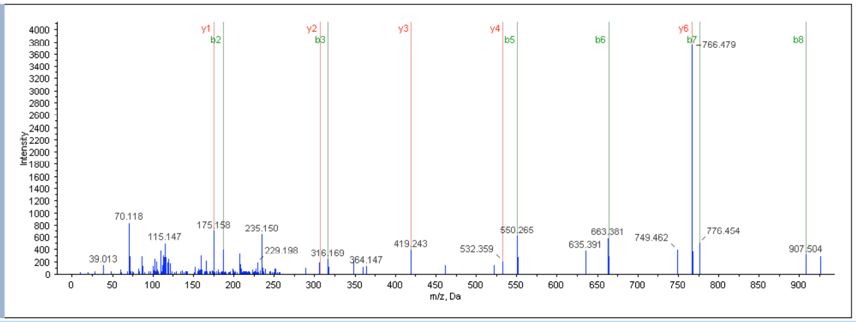

179	2426.041	Fragmentation Evidence for Peptide

NVVDGSGYVSGYQHKDTYDNAH

Residue	b	y
N	115.0502	2426.0644
V	214.1186	2312.0214
V	313.1870	2212.9530
D	428.2140	2113.8846
G	485.2354	1998.8577
S	572.2675	1941.8362
G	629.2889	1854.8042
Y	792.3523	1797.7827
V	891.4207	1634.7194
S	978.4527	1535.6510
G	1035.4742	1448.6189
Y	1198.5375	1391.5975
Q	1326.5961	1228.5341
H	1463.6550	1100.4756
K	1591.7499	963.4167
D	1706.7769	835.3217
T	1807.8246	720.2947
Y	1970.8879	619.2471
D	2085.9148	456.1837
N	2199.9578	341.1568
A	2270.9949	227.1139
H	2408.0538	156.0768

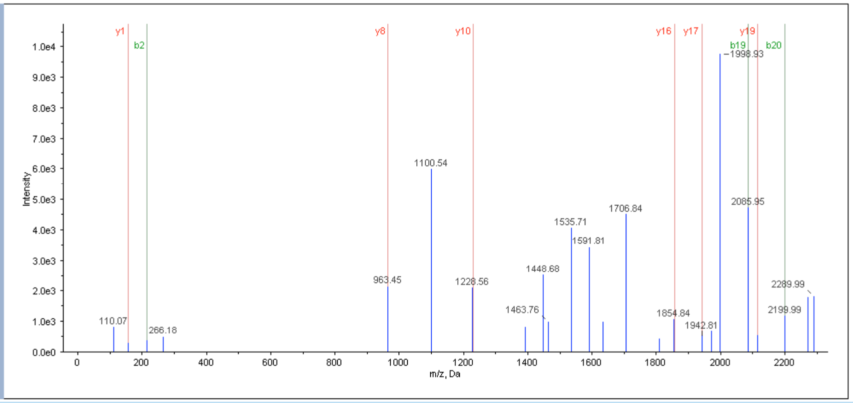

180	1397.686	

Fragmentation Evidence for Peptide

VSDHLDWIREK

Residue	b	y
V	**100.0757**	1397.7172
S	187.1077	1298.6488
D	**302.1347**	**1211.6167**
H	439.1936	1096.5898
L	552.2776	959.5309
D	**667.3046**	846.4468
W	**853.3839**	**731.4199**
I	966.4680	**545.3406**
R	1122.5691	**432.2565**
E	**1251.6117**	276.1554
K	1379.7066	**147.1128**

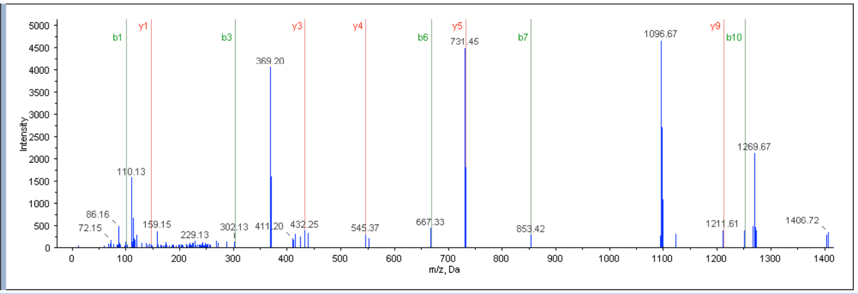

181	2480.319	

Fragmentation Evidence for Peptide

NAGGQALPPPAAGGVPQQQLPVGAVSR

Residue	b	y
N	115.0502	2480.3368
A	**186.0873**	2366.2939
G	243.1088	2295.2568
Q	**371.1674**	2238.2353
A	442.2045	**2110.1767**
L	555.2885	**2039.1396**
P	652.3413	**1926.0556**
P	749.3941	1829.0028
P	846.4468	1731.9500
A	917.4839	1634.8973
A	988.5211	1563.8602
G	1045.5425	1492.8231
G	1102.5640	1435.8016
V	1201.6324	1378.7801
P	1298.6852	**1279.7117**
Q	1426.7437	1182.6589
Q	1554.8023	1054.6004
Q	1682.8609	926.5418
L	1795.9450	798.4832
P	1892.9977	685.3991
V	1992.0661	588.3464
G	2049.0876	489.2780
A	2120.1247	432.2565
V	2219.1931	**361.2194**
S	2306.2252	262.1510
R	2462.3263	**175.1190**

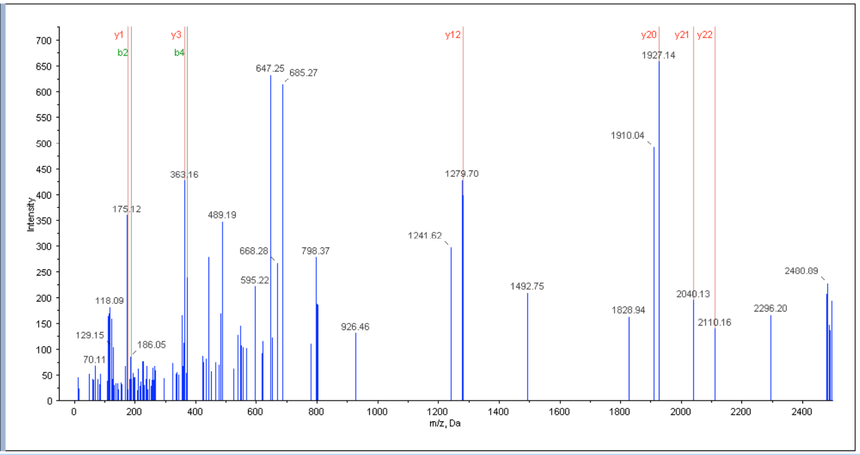

182	1466.877	Fragmentation Evidence for Peptide<br>LVTGWQKPIVIGR<br>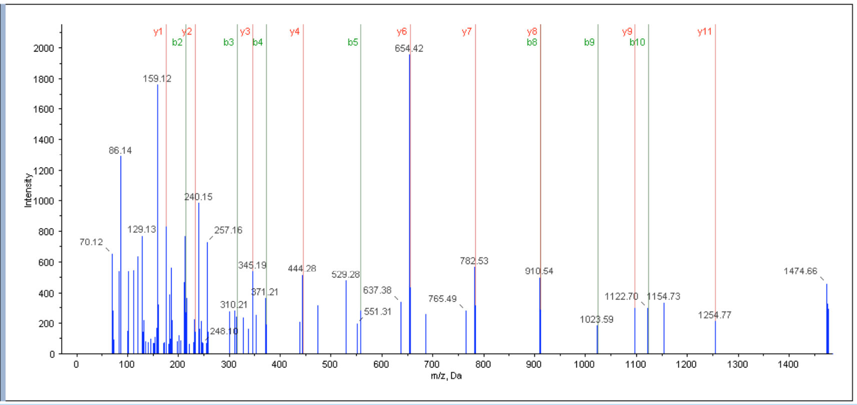
183	2203.077	Fragmentation Evidence for Peptide<br>KGEDEPLEDTAISGSDAANKR<br>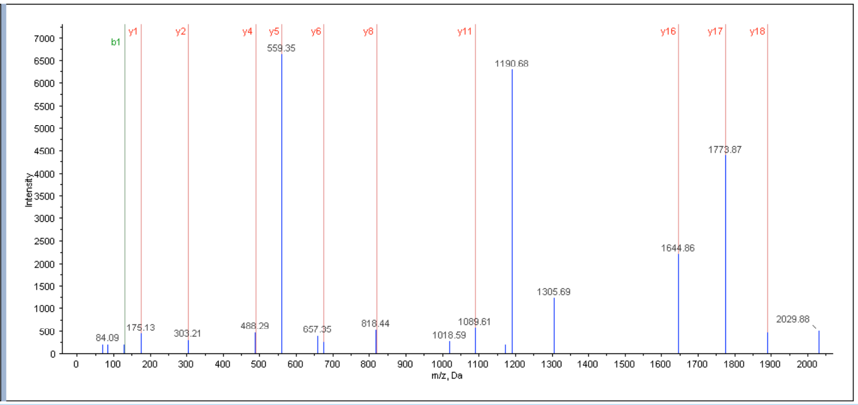

LVTGWQKPIVIGR

Residue	b	y
L	114.0913	1466.8842
V	**213.1598**	1353.8001
T	**314.2074**	**1254.7317**
G	**371.2289**	1153.6840
W	**557.3082**	**1096.6626**
Q	685.3668	**910.5833**
K	813.4618	**782.5247**
P	**910.5145**	**654.4297**
I	**1023.5986**	557.3770
V	**1122.6670**	**444.2929**
I	1235.7511	**345.2245**
G	1292.7725	**232.1404**
R	1448.8736	**175.1190**

KGEDEPLEDTAISGSDAANKR

Residue	b	y
K	**129.1022**	2203.0473
G	186.1237	2074.9523
E	315.1663	2017.9309
D	430.1932	**1888.8883**
E	559.2358	**1773.8613**
P	656.2886	**1644.8188**
L	769.3727	1547.7660
E	898.4153	1434.6819
D	1013.4422	1305.6393
T	1114.4899	1190.6124
A	1185.5270	**1089.5647**
I	1298.6111	1018.5276
S	1385.6431	905.4435
G	1442.6645	**818.4115**
S	1529.6966	761.3900
D	1644.7235	**674.3580**
A	1715.7606	**559.3311**
A	1786.7977	**488.2940**
N	1900.8407	417.2568
K	2028.9356	**303.2139**
R	2185.0367	**175.1190**

184	1785.913	

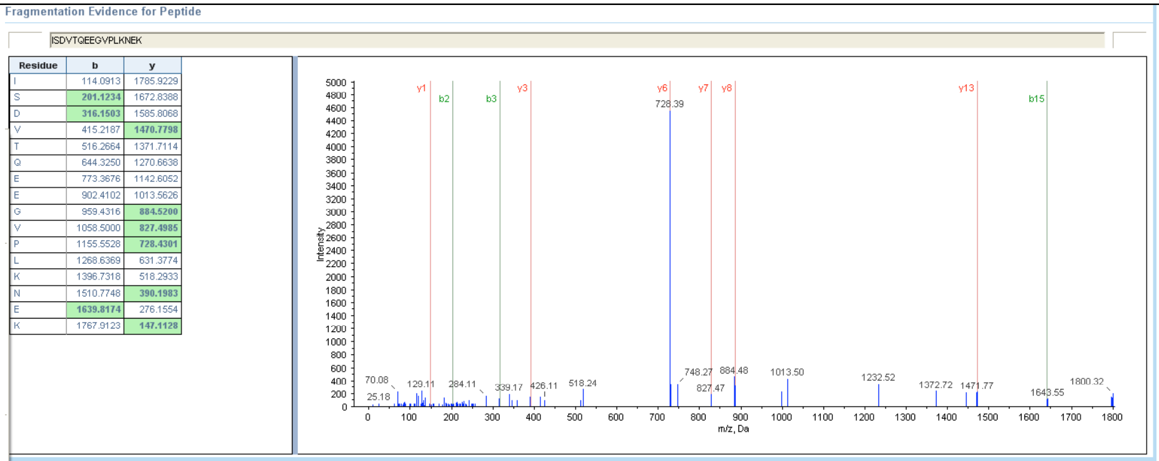

Fragmentation Evidence for Peptide

ISDVTQEEGVPLKNEK

Residue	b	y
I	114.0913	1785.9229
S	**201.1234**	1672.8388
D	**316.1503**	1585.8068
V	415.2187	**1470.7798**
T	516.2664	1371.7114
Q	644.3250	1270.6638
E	773.3676	1142.6052
E	902.4102	1013.5626
G	959.4316	**884.5200**
V	1058.5000	**827.4985**
P	1155.5528	**728.4301**
L	1268.6369	631.3774
K	1396.7318	518.2933
N	1510.7748	**390.1983**
E	**1639.8174**	276.1554
K	1767.9123	**147.1128**

185	1795.889	

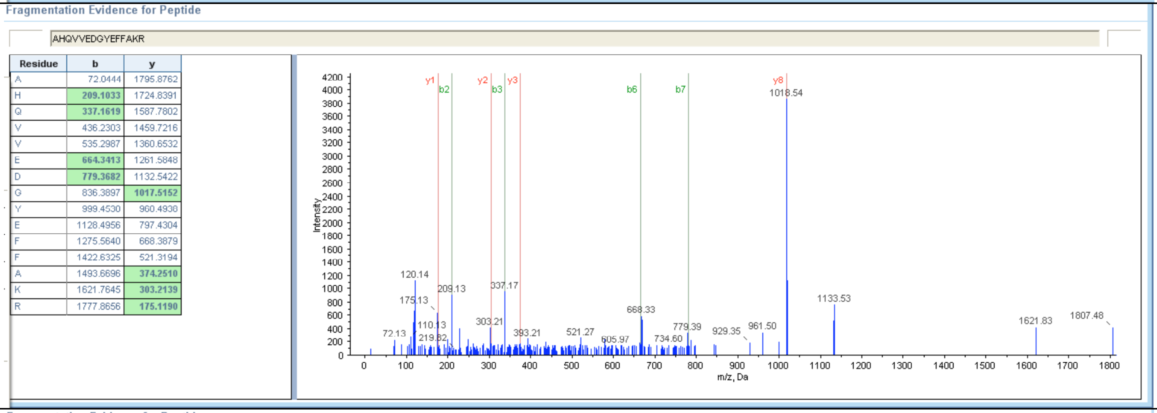

Fragmentation Evidence for Peptide

AHQVVEDGYEFFAKR

Residue	b	y
A	72.0444	1795.8762
H	**209.1033**	1724.8391
Q	**337.1619**	1587.7802
V	436.2303	1459.7216
V	535.2987	1360.6532
E	**664.3413**	1261.5848
D	**779.3682**	1132.5422
G	836.3897	**1017.5152**
Y	999.4530	960.4938
E	1128.4956	797.4304
F	1275.5640	668.3879
F	1422.6325	521.3194
A	1493.6696	**374.2510**
K	1621.7645	**303.2139**
R	1777.8656	**175.1190**

186	1002.576	

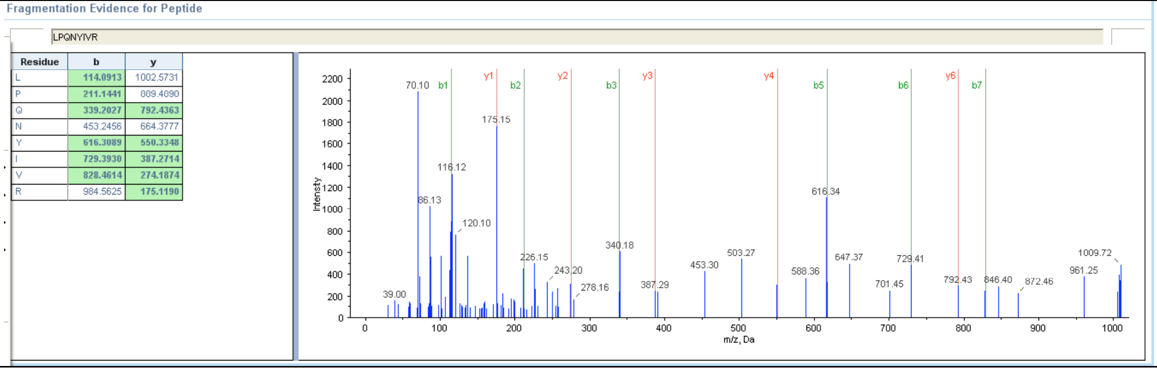

Fragmentation Evidence for Peptide

LPQNYIVR

Residue	b	y
L	**114.0913**	1002.5731
P	**211.1441**	889.4890
Q	**339.2027**	**792.4363**
N	453.2456	664.3777
Y	**616.3089**	**550.3348**
I	**729.3930**	**387.2714**
V	**828.4614**	**274.1874**
R	984.5625	**175.1190**

<table>
<tr>
<td>187</td>
<td>1203.618</td>
<td>
Fragmentation Evidence for Peptide
IGEAREELMR
<table>
<tr><th>Residue</th><th>b</th><th>y</th></tr>
<tr><td>I</td><td>114.0913</td><td>1203.6150</td></tr>
<tr><td>G</td><td>171.1128</td><td>1090.5310</td></tr>
<tr><td>E</td><td>300.1554</td><td>1033.5095</td></tr>
<tr><td>A</td><td>371.1925</td><td>904.4669</td></tr>
<tr><td>R</td><td>527.2936</td><td>833.4298</td></tr>
<tr><td>E</td><td>656.3362</td><td>677.3287</td></tr>
<tr><td>E</td><td>785.3788</td><td>548.2861</td></tr>
<tr><td>L</td><td>898.4629</td><td>419.2435</td></tr>
<tr><td>M</td><td>1029.5034</td><td>306.1594</td></tr>
<tr><td>R</td><td>1185.6045</td><td>175.1190</td></tr>
</table>
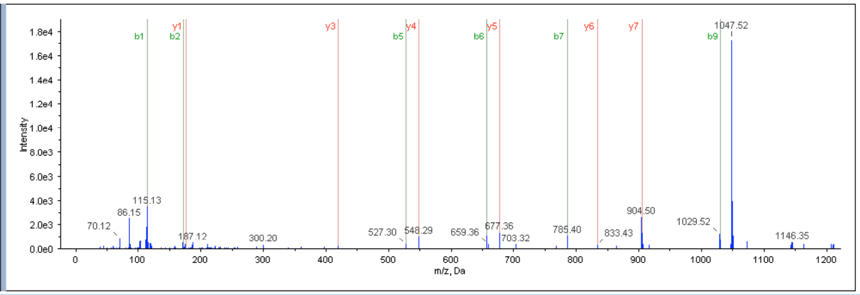
</td>
</tr>
<tr>
<td>188</td>
<td>1806.874</td>
<td>
Fragmentation Evidence for Peptide
STEVDKVC[CAM]LPFPGSDR
<table>
<tr><th>Residue</th><th>b</th><th>y</th></tr>
<tr><td>S</td><td>88.0393</td><td>1806.8691</td></tr>
<tr><td>T</td><td>189.0870</td><td>1719.8370</td></tr>
<tr><td>E</td><td>318.1296</td><td>1618.7894</td></tr>
<tr><td>V</td><td>417.1980</td><td>1489.7468</td></tr>
<tr><td>D</td><td>532.2249</td><td>1390.6784</td></tr>
<tr><td>K</td><td>660.3199</td><td>1275.6514</td></tr>
<tr><td>V</td><td>759.3883</td><td>1147.5565</td></tr>
<tr><td>C[CAM]</td><td>919.4190</td><td>1048.4880</td></tr>
<tr><td>L</td><td>1032.5030</td><td>888.4574</td></tr>
<tr><td>P</td><td>1129.5558</td><td>775.3733</td></tr>
<tr><td>F</td><td>1276.6242</td><td>678.3206</td></tr>
<tr><td>P</td><td>1373.6770</td><td>531.2522</td></tr>
<tr><td>G</td><td>1430.6984</td><td>434.1994</td></tr>
<tr><td>S</td><td>1517.7305</td><td>377.1779</td></tr>
<tr><td>D</td><td>1632.7574</td><td>290.1459</td></tr>
<tr><td>R</td><td>1788.8585</td><td>175.1190</td></tr>
</table>
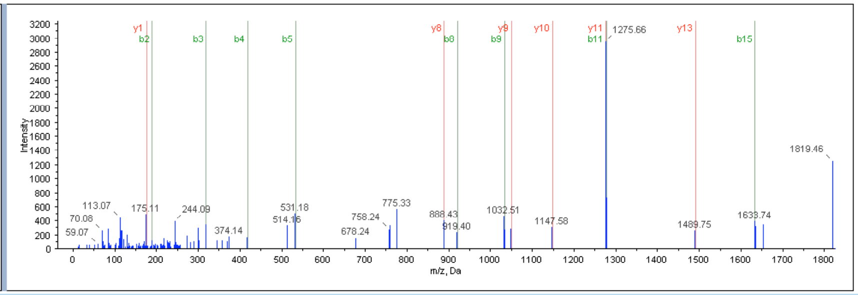
</td>
</tr>
<tr>
<td>189</td>
<td>2091.011</td>
<td>
Fragmentation Evidence for Peptide
SANGSN[Dea]AVTPTTSANTSPISGR
<table>
<tr><th>Residue</th><th>b</th><th>y</th></tr>
<tr><td>S</td><td>88.0393</td><td>2090.9949</td></tr>
<tr><td>A</td><td>159.0764</td><td>2003.9629</td></tr>
<tr><td>N</td><td>273.1193</td><td>1932.9257</td></tr>
<tr><td>G</td><td>330.1408</td><td>1818.8828</td></tr>
<tr><td>S</td><td>417.1728</td><td>1761.8613</td></tr>
<tr><td>N[Dea]</td><td>532.1998</td><td>1674.8293</td></tr>
<tr><td>A</td><td>603.2369</td><td>1559.8024</td></tr>
<tr><td>V</td><td>702.3053</td><td>1488.7653</td></tr>
<tr><td>T</td><td>803.3530</td><td>1389.6968</td></tr>
<tr><td>P</td><td>900.4058</td><td>1288.6492</td></tr>
<tr><td>T</td><td>1001.4534</td><td>1191.5964</td></tr>
<tr><td>T</td><td>1102.5011</td><td>1090.5487</td></tr>
<tr><td>S</td><td>1189.5331</td><td>989.5010</td></tr>
<tr><td>A</td><td>1260.5703</td><td>902.4690</td></tr>
<tr><td>N</td><td>1374.6132</td><td>831.4319</td></tr>
<tr><td>T</td><td>1475.6609</td><td>717.3890</td></tr>
<tr><td>S</td><td>1562.6929</td><td>616.3413</td></tr>
<tr><td>P</td><td>1659.7456</td><td>529.3093</td></tr>
<tr><td>I</td><td>1772.8297</td><td>432.2565</td></tr>
<tr><td>S</td><td>1859.8617</td><td>319.1724</td></tr>
<tr><td>G</td><td>1916.8832</td><td>232.1404</td></tr>
<tr><td>R</td><td>2072.9843</td><td>175.1190</td></tr>
</table>
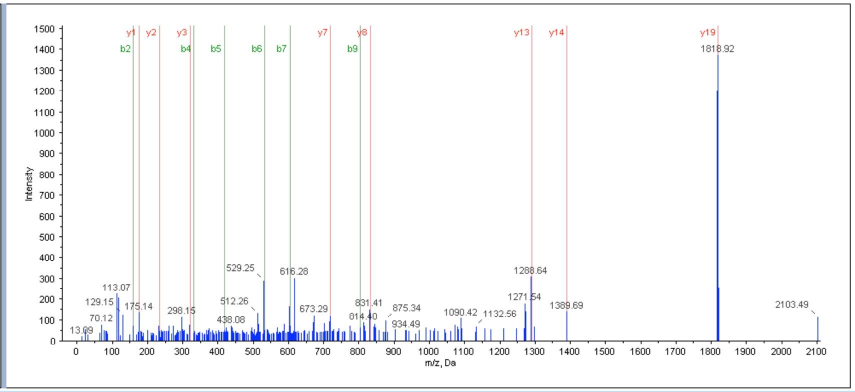
</td>
</tr>
</table>

190	1040.546	Fragmentation Evidence for Peptide<br>GIFLYDGQK

Residue	b	y
G	58.0287	1040.5411
I	171.1128	983.5197
F	318.1812	870.4356
L	431.2653	723.3672
Y	594.3286	610.2831
D	709.3556	447.2198
G	766.3770	332.1928
Q	894.4356	275.1714
K	1022.5306	147.1128

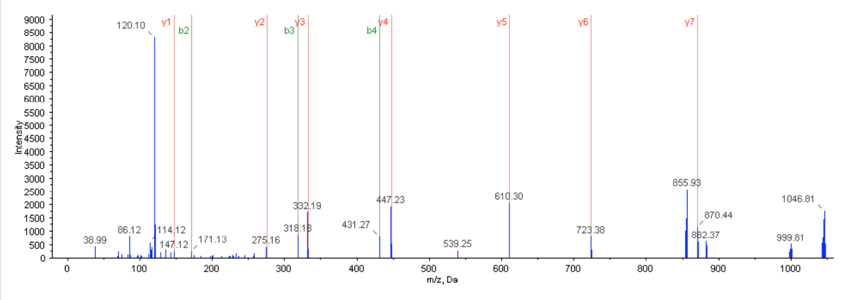

191	1631.808	Fragmentation Evidence for Peptide<br>TLNIRKDGPC[CAM]NETN

Residue	b	y
T	102.0550	1631.7806
L	215.1390	1530.7329
N	329.1819	1417.6489
I	442.2660	1303.6059
R	598.3671	1190.5219
K	726.4621	1034.4207
D	841.4890	906.3258
G	898.5105	791.2988
P	995.5633	734.2774
C[CAM]	1155.5939	637.2246
N	1269.6368	477.1940
E	1398.6794	363.1510
T	1499.7271	234.1084
N	1613.7700	133.0608

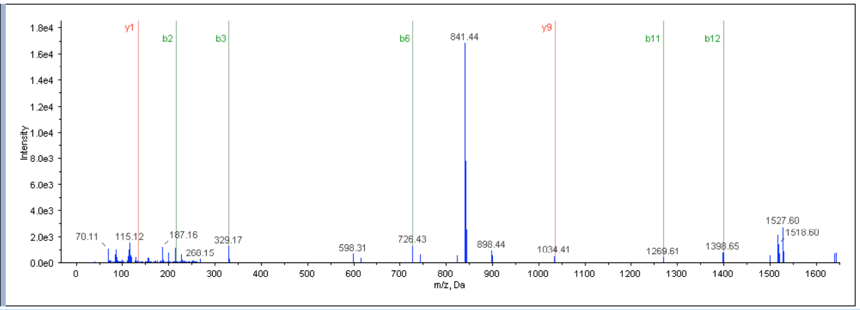

192	2161.059	Fragmentation Evidence for Peptide<br>AIELEPDNEVYKSNLEAAR

Residue	b	y
A	72.0444	2161.0772
I	185.1285	2090.0400
E	314.1710	1976.9560
L	427.2551	1847.9134
E	556.2977	1734.8293
P	653.3505	1605.7867
D	768.3774	1508.7340
N	882.4203	1393.7070
E	1011.4629	1279.6641
V	1110.5313	1150.6215
Y	1273.5947	1051.5531
K	1401.6896	888.4898
S	1488.7217	760.3948
N	1602.7646	673.3628
L	1715.8487	559.3198
E	1844.8913	446.2358
A	1915.9284	317.1932
A	1986.9655	246.1561
R	2143.0666	175.1190

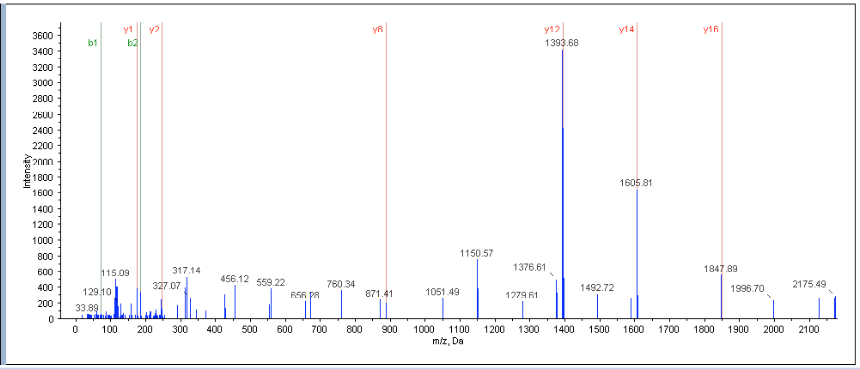

193	1402.746	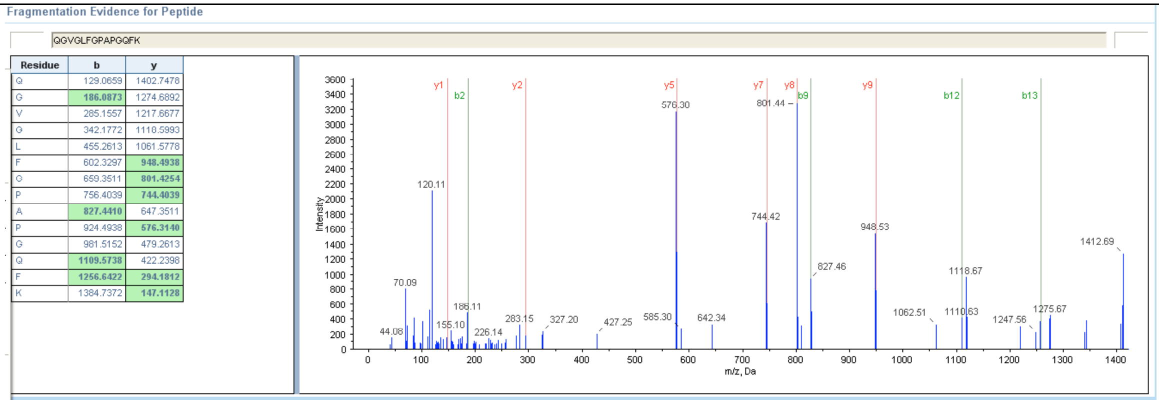

Residue	b	y
Q	129.0659	1402.7478
G	186.0873	1274.6892
V	285.1557	1217.6677
G	342.1772	1118.5993
L	455.2613	1061.5778
F	602.3297	948.4938
G	659.3511	801.4254
P	756.4039	744.4039
A	827.4410	647.3511
P	924.4938	576.3140
G	981.5152	479.2613
Q	1109.5738	422.2398
F	1256.6422	294.1812
K	1384.7372	147.1128

194	1459.63	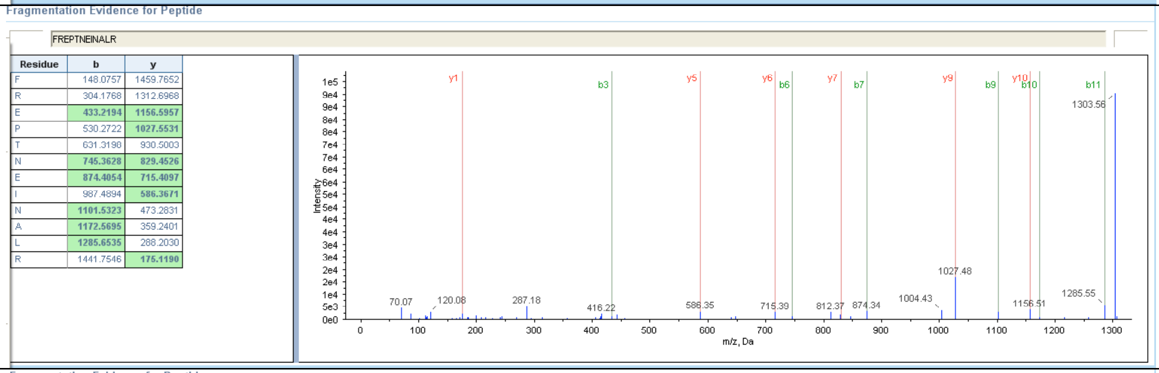

Residue	b	y
F	148.0757	1459.7652
R	304.1768	1312.6968
E	433.2194	1156.5957
P	530.2722	1027.5531
T	631.3198	930.5003
N	745.3628	829.4526
E	874.4054	715.4097
I	987.4894	586.3671
N	1101.5323	473.2831
A	1172.5695	359.2401
L	1285.6535	288.2030
R	1441.7546	175.1190

195	1406.783	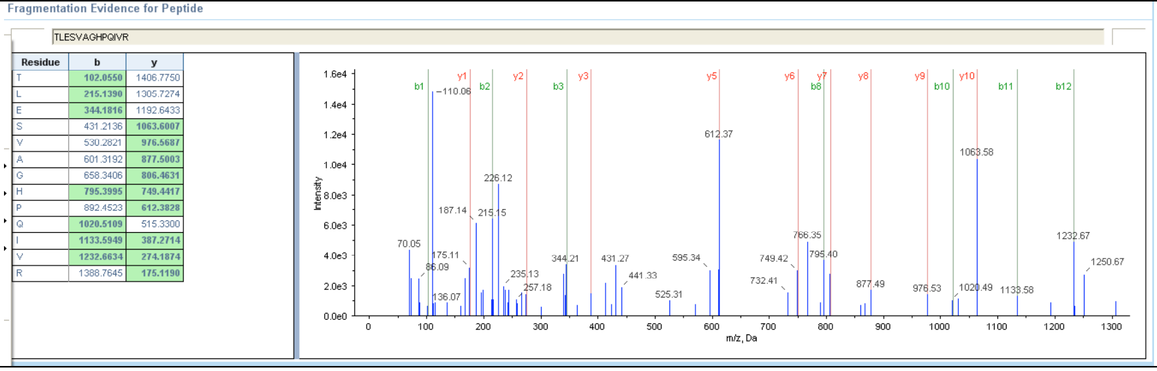

Residue	b	y
T	102.0550	1406.7750
L	215.1390	1305.7274
E	344.1816	1192.6433
S	431.2136	1063.6007
V	530.2821	976.5687
A	601.3192	877.5003
G	658.3406	806.4631
H	795.3995	749.4417
P	892.4523	612.3828
Q	1020.5109	515.3300
I	1133.5949	387.2714
V	1232.6634	274.1874
R	1388.7645	175.1190

196	1524.716		
197	2449.019		
198	912.5043		

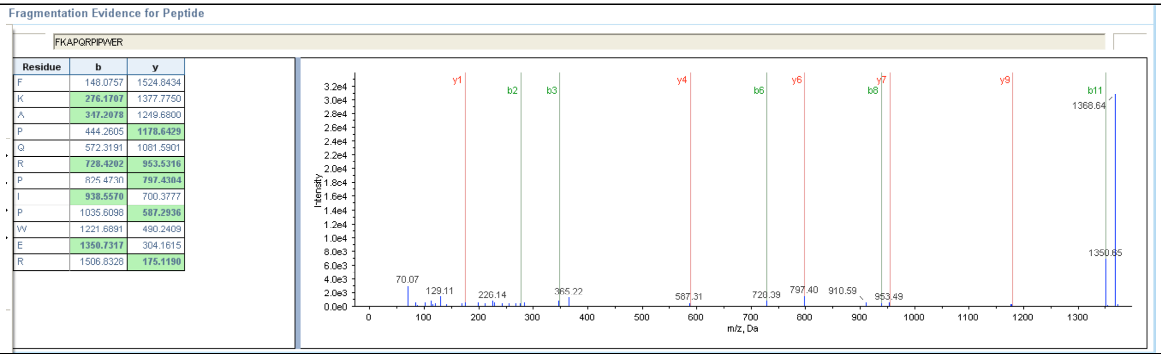

Fragmentation Evidence for Peptide

FKAPQRPIPWER

Residue	b	y
F	148.0757	1524.8434
K	**276.1707**	1377.7750
A	**347.2078**	1249.6800
P	444.2605	**1178.6429**
Q	572.3191	1081.5901
R	**728.4202**	**953.5316**
P	825.4730	**797.4304**
I	**938.5570**	700.3777
P	1035.6098	**587.2936**
W	1221.6891	490.2409
E	**1350.7317**	304.1615
R	1506.8328	**175.1190**

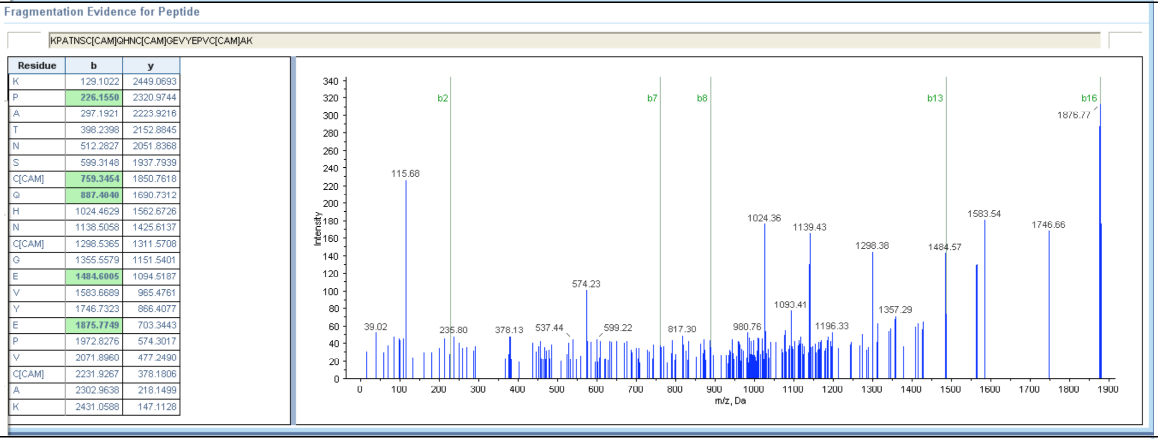

Fragmentation Evidence for Peptide

KPATNSC[CAM]QHNC[CAM]GEVYEPVC[CAM]AK

Residue	b	y
K	129.1022	2449.0693
P	**226.1550**	2320.9744
A	297.1921	2223.9216
T	398.2398	2152.8845
N	512.2827	2051.8368
S	599.3148	1937.7939
C[CAM]	**759.3454**	1850.7618
Q	**887.4040**	1690.7312
H	1024.4629	1562.6726
N	1138.5058	1425.6137
C[CAM]	1298.5365	1311.5708
G	1355.5579	1151.5401
E	**1484.6005**	1094.5187
V	1583.6689	965.4761
Y	1746.7323	866.4077
E	**1875.7749**	703.3443
P	1972.8276	574.3017
V	2071.8960	477.2490
C[CAM]	2231.9267	378.1806
A	2302.9638	218.1499
K	2431.0588	147.1128

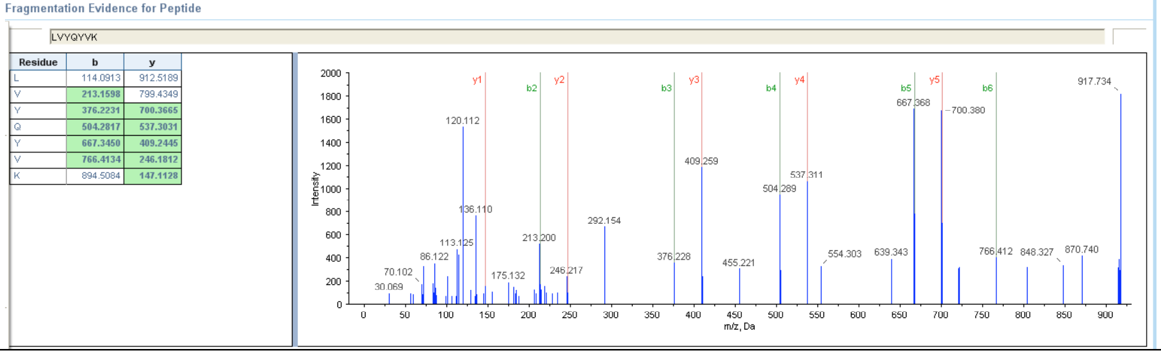

Fragmentation Evidence for Peptide

LVYQYVK

Residue	b	y
L	114.0913	912.5189
V	**213.1598**	799.4349
Y	**376.2231**	**700.3665**
Q	**504.2817**	**537.3031**
Y	**667.3450**	**409.2445**
V	**766.4134**	**246.1812**
K	894.5084	**147.1128**

Khanna et al. Supplementary Table 3
(Part 3. Spectra #199-#281)

<table>
<tr><th colspan="3">Spectra for single peptide identifications</th></tr>
<tr><th>Spectrum Number</th><th>Precursor m/z</th><th>Spectrum</th></tr>
<tr><td>199</td><td>2073.035</td><td>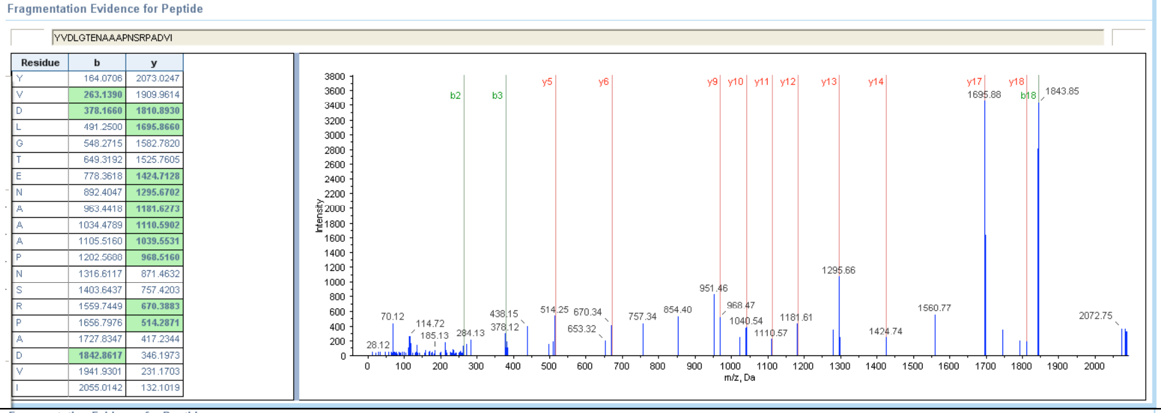
</td></tr>
<tr><td>200</td><td>994.5297</td><td>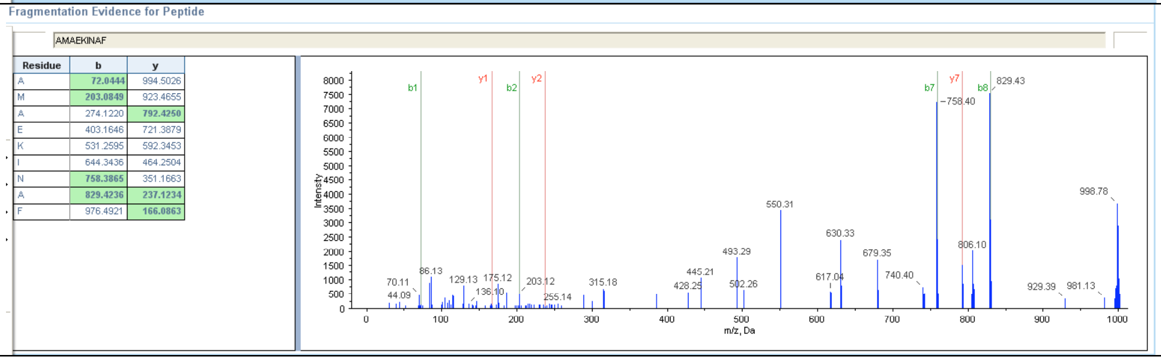
</td></tr>
</table>

201	1291.671		Fragmentation Evidence for Peptide: ALAELSKPTPKH (see below)
202	1546.872		Fragmentation Evidence for Peptide: VMATALKEASEKLR (see below)
203	1646.834		Fragmentation Evidence for Peptide: DIEKPHVGDSASVHR (see below)

201 — 1291.671

Fragmentation Evidence for Peptide

ALAELSKPTPKH

Residue	b	y
A	72.0444	1291.7369
L	**185.1285**	1220.6997
A	**256.1656**	1107.6157
E	**385.2082**	1036.5786
L	**498.2922**	**907.5360**
S	**585.3243**	**794.4519**
K	**713.4192**	**707.4199**
P	**810.4720**	**579.3249**
T	**911.5197**	**482.2722**
P	1008.5724	**381.2245**
K	**1136.6674**	284.1717
H	1273.7263	**156.0768**

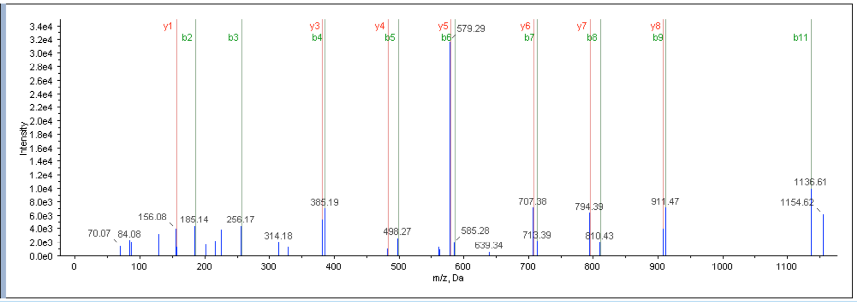

202 — 1546.872

Fragmentation Evidence for Peptide

VMATALKEASEKLR

Residue	b	y
V	100.0757	1546.8621
M	**231.1162**	1447.7937
A	**302.1533**	1316.7532
T	403.2010	1245.7161
A	**474.2381**	1144.6684
L	587.3221	1073.6313
K	715.4171	960.5473
E	**844.4597**	**832.4523**
A	**915.4968**	**703.4097**
S	1002.5288	**632.3726**
E	1131.5714	**545.3406**
K	1259.6664	**416.2980**
L	**1372.7505**	288.2030
R	1528.8516	**175.1190**

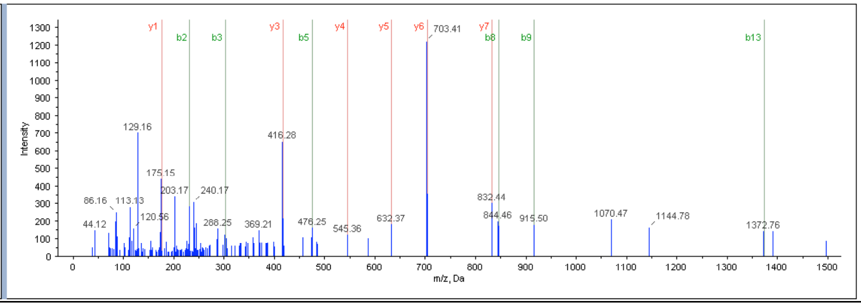

203 — 1646.834

Fragmentation Evidence for Peptide

DIEKPHVGDSASVHR

Residue	b	y
D	116.0342	1646.8245
I	**229.1183**	1531.7976
E	358.1609	1418.7135
K	486.2558	**1289.6709**
P	583.3086	**1161.5759**
H	720.3675	1064.5232
V	819.4359	**927.4643**
G	876.4574	**828.3959**
D	991.4843	**771.3744**
S	1078.5164	**656.3474**
A	1149.5535	569.3154
S	1236.5855	498.2783
V	1335.6539	**411.2463**
H	**1472.7128**	**312.1779**
R	1628.8139	**175.1190**

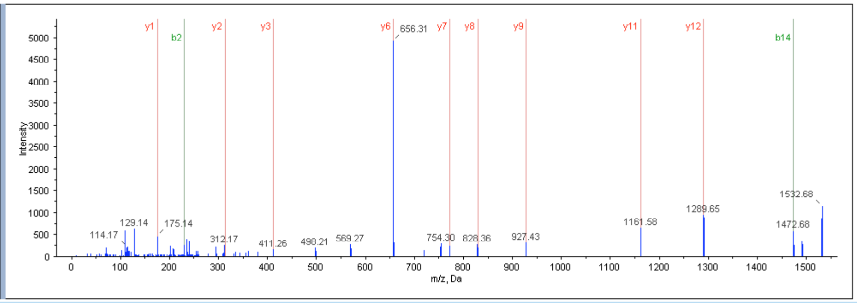

204	1444.805	Fragmentation Evidence for Peptide: LALADTGKKPFQR

Fragmentation Evidence for Peptide

LALADTGKKPFQR

Residue	b	y
L	114.0913	1444.8271
A	**185.1285**	1331.7430
L	**298.2125**	1260.7059
A	369.2496	1147.6218
D	**484.2766**	1076.5847
T	585.3243	**961.5578**
G	642.3457	860.5101
K	770.4407	803.4886
K	**898.5356**	**675.3937**
P	995.5884	**547.2987**
F	1142.6568	450.2459
Q	**1270.7154**	**303.1775**
R	1426.8165	175.1190

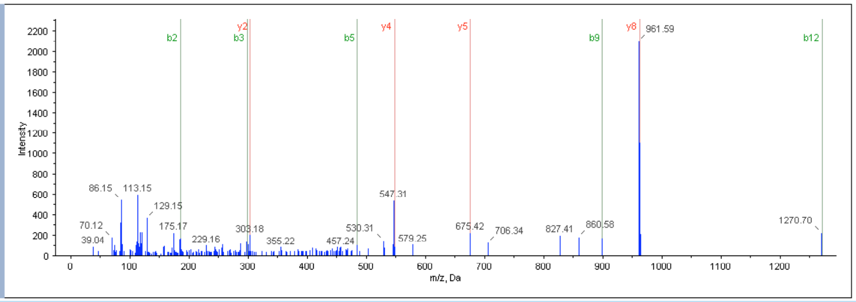

205	1976.989	Fragmentation Evidence for Peptide: YQRVNDLAGSGPGSVAGNSK

Fragmentation Evidence for Peptide

YQRVNDLAGSGPGSVAGNSK

Residue	b	y
Y	164.0706	1976.9784
Q	**292.1292**	1813.9151
R	448.2303	1685.8565
V	547.2987	1529.7554
N	661.3416	1430.6870
D	776.3686	1316.6441
L	**889.4526**	**1201.6171**
A	960.4898	1088.5331
G	1017.5112	1017.4960
S	1104.5432	**960.4745**
G	**1161.5647**	873.4425
P	1258.6175	**816.4210**
G	1315.6389	719.3682
S	1402.6710	662.3468
V	1501.7394	575.3148
A	1572.7765	476.2463
G	1629.7980	405.2092
N	1743.8409	348.1878
S	1830.8729	**234.1448**
K	1958.9679	147.1128

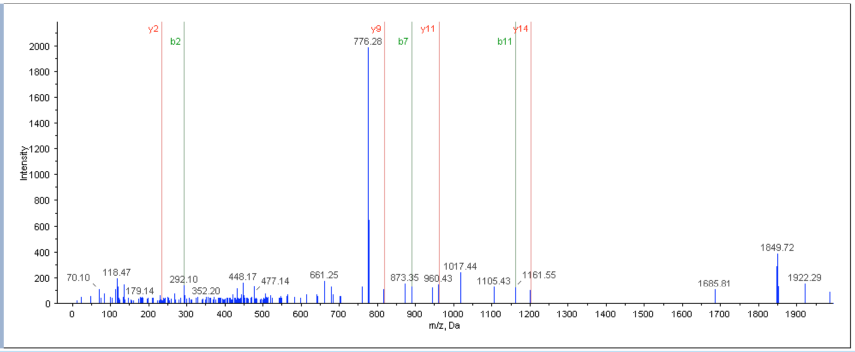

206	1116.572	Fragmentation Evidence for Peptide: TFINPYYAK

Fragmentation Evidence for Peptide

TFINPYYAK

Residue	b	y
T	**102.0550**	1116.5724
F	**249.1234**	1015.5247
I	362.2074	**868.4563**
N	**476.2504**	**755.3723**
P	**573.3031**	**641.3293**
Y	**736.3665**	544.2766
Y	**899.4298**	**381.2132**
A	**970.4669**	**218.1499**
K	1098.5619	**147.1128**

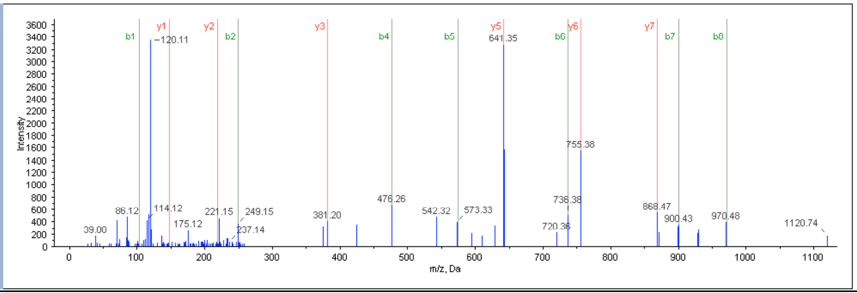

207	1244.7		Fragmentation Evidence for Peptide

Fragmentation Evidence for Peptide

GLKPVFEEAVR

Residue	b	y
G	58.0287	1244.6997
L	**171.1128**	1187.6783
K	**299.2078**	**1074.5942**
P	**396.2605**	**946.4993**
V	**495.3289**	849.4465
F	**642.3974**	**750.3781**
E	**771.4400**	**603.3097**
E	**900.4825**	**474.2671**
A	**971.5197**	**345.2245**
V	**1070.5881**	**274.1874**
R	1226.6892	**175.1190**

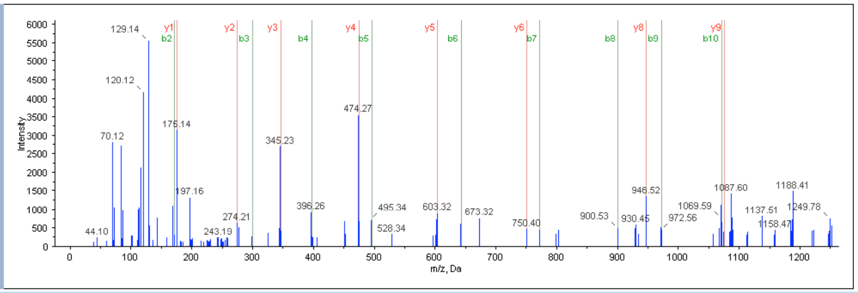

208	1206.611		Fragmentation Evidence for Peptide

Fragmentation Evidence for Peptide

NYTNVANALAR

Residue	b	y
N	115.0502	1206.6226
Y	**278.1135**	1092.5796
T	**379.1612**	929.5163
N	**493.2041**	828.4686
V	**592.2726**	**714.4257**
A	663.3097	**615.3573**
N	777.3526	**544.3202**
A	**848.3897**	**430.2772**
L	961.4738	**359.2401**
A	1032.5109	**246.1561**
R	1188.6120	**175.1190**

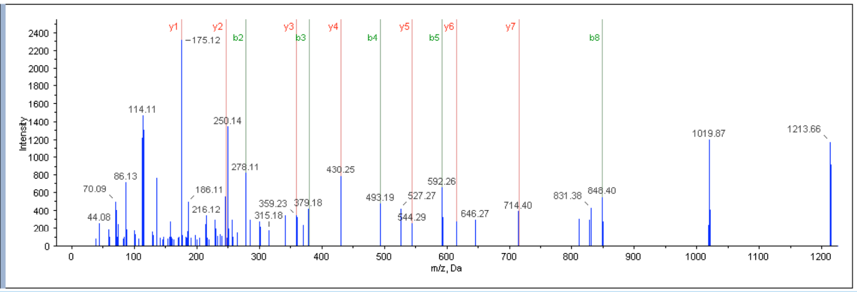

209	1377.729		Fragmentation Evidence for Peptide

Fragmentation Evidence for Peptide

AFGVQTISGTGALR

Residue	b	y
A	72.0444	1377.7485
F	**219.1128**	1306.7114
G	**276.1343**	1159.6430
V	**375.2027**	1102.6215
Q	503.2613	1003.5531
T	**604.3089**	875.4945
I	717.3930	774.4468
S	804.4250	**661.3628**
G	861.4465	574.3307
T	962.4942	517.3093
G	1019.5156	**416.2616**
A	1090.5527	359.2401
L	1203.6368	**288.2030**
R	1359.7379	**175.1190**

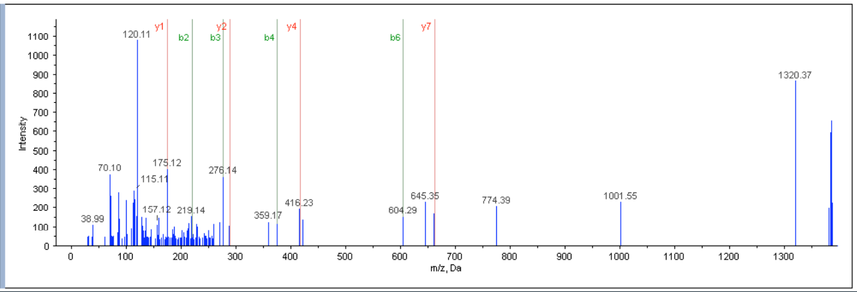

210	1360.645	Fragmentation Evidence for Peptide

Fragmentation Evidence for Peptide

SRAPGGAGTGGMWR

Residue	b	y
S	88.0393	1360.6539
R	**244.1404**	1273.6218
A	315.1775	**1117.5207**
P	412.2303	**1046.4836**
G	469.2518	949.4309
G	526.2732	892.4094
A	597.3103	835.3879
G	654.3318	**764.3508**
T	**755.3795**	707.3294
G	**812.4009**	**606.2817**
G	869.4224	549.2602
M	1000.4629	492.2387
W	**1186.5422**	361.1983
R	1342.6433	175.1190

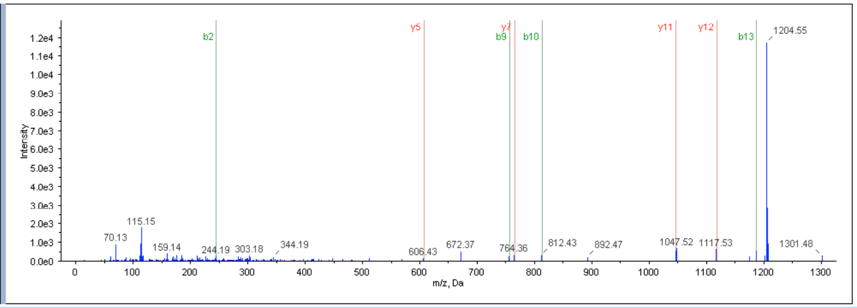

211	2248.015	Fragmentation Evidence for Peptide

SSSNVNSQVGEGAGSSVDADAPGR

Residue	b	y
S	88.0393	2248.0072
S	175.0713	2160.9752
S	262.1034	2073.9432
N	376.1463	1986.9112
V	475.2147	1872.8682
N	589.2576	**1773.7998**
S	**676.2897**	1659.7569
Q	804.3482	1572.7249
V	**903.4167**	**1444.6663**
G	960.4381	**1345.5979**
E	1089.4807	1288.5764
G	1146.5022	**1159.5338**
A	1217.5393	1102.5123
G	1274.5607	**1031.4752**
S	1361.5928	**974.4538**
S	1448.6248	887.4217
V	1547.6932	**800.3897**
D	1662.7202	701.3213
A	1733.7573	586.2944
D	1848.7842	515.2572
A	1919.8213	400.2303
P	2016.8741	**329.1932**
G	2073.8956	**232.1404**
R	2229.9967	**175.1190**

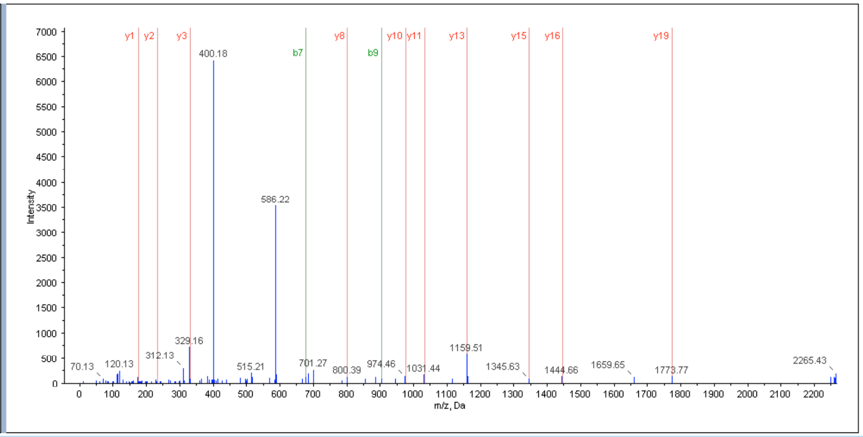

212	1735.886	Fragmentation Evidence for Peptide

VGTDYIQDVDKLQKN

Residue	b	y
V	100.0757	1735.8861
G	157.0972	1636.8177
T	258.1448	1579.7962
D	**373.1718**	1478.7485
Y	536.2351	**1363.7216**
I	649.3192	1200.6583
Q	777.3777	**1087.5742**
D	**892.4047**	**959.5156**
V	991.4731	**844.4887**
D	1106.5000	745.4203
K	1234.5950	**630.3933**
L	**1347.6791**	502.2984
Q	**1475.7377**	389.2143
K	**1603.8326**	**261.1557**
N	1717.8755	133.0608

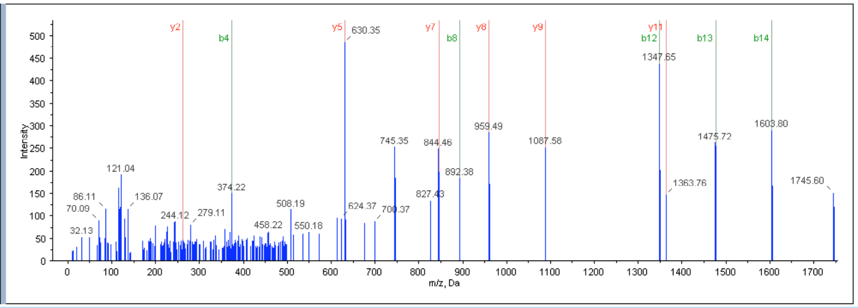

213	2291.126	

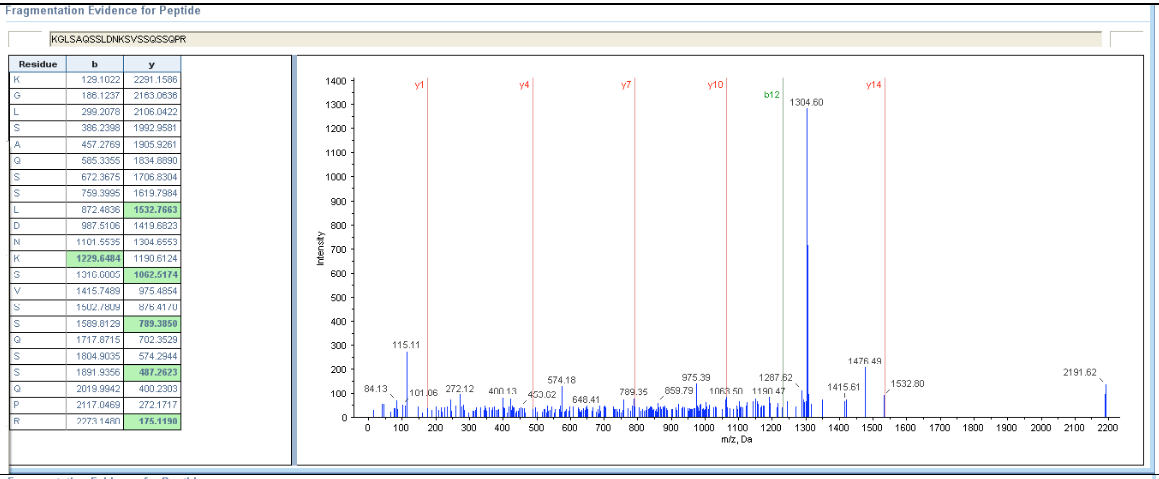

Residue	b	y
K	129.1022	2291.1586
G	186.1237	2163.0636
L	299.2078	2106.0422
S	386.2398	1992.9581
A	457.2769	1905.9261
Q	585.3355	1834.8890
S	672.3675	1706.8304
S	759.3995	1619.7984
L	872.4836	**1532.7663**
D	987.5106	1419.6823
N	1101.5535	1304.6553
K	**1229.6484**	1190.6124
S	1316.6805	**1062.5174**
V	1415.7489	975.4854
S	1502.7809	876.4170
S	1589.8129	**789.3850**
Q	1717.8715	702.3529
S	1804.9035	574.2944
S	1891.9356	**487.2623**
Q	2019.9942	400.2303
P	2117.0469	272.1717
R	2273.1480	**175.1190**

214	1732.81	

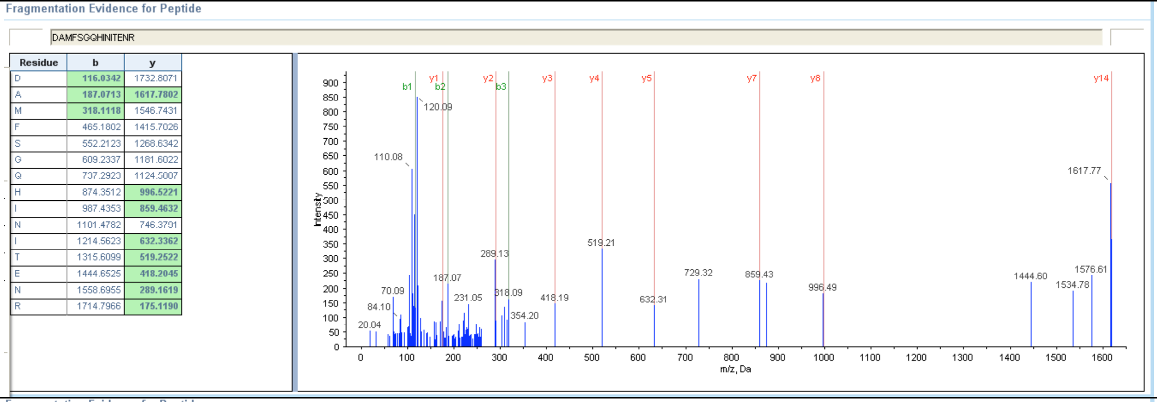

Residue	b	y
D	**116.0342**	1732.8071
A	**187.0713**	**1617.7802**
M	**318.1118**	1546.7431
F	465.1802	1415.7026
S	552.2123	1268.6342
G	609.2337	1181.6022
Q	737.2923	1124.5807
H	874.3512	**996.5221**
I	987.4353	**859.4632**
N	1101.4782	746.3791
I	1214.5623	**632.3362**
T	1315.6099	**519.2522**
E	1444.6525	**418.2045**
N	1558.6955	**289.1619**
R	1714.7966	**175.1190**

215	1075.576	

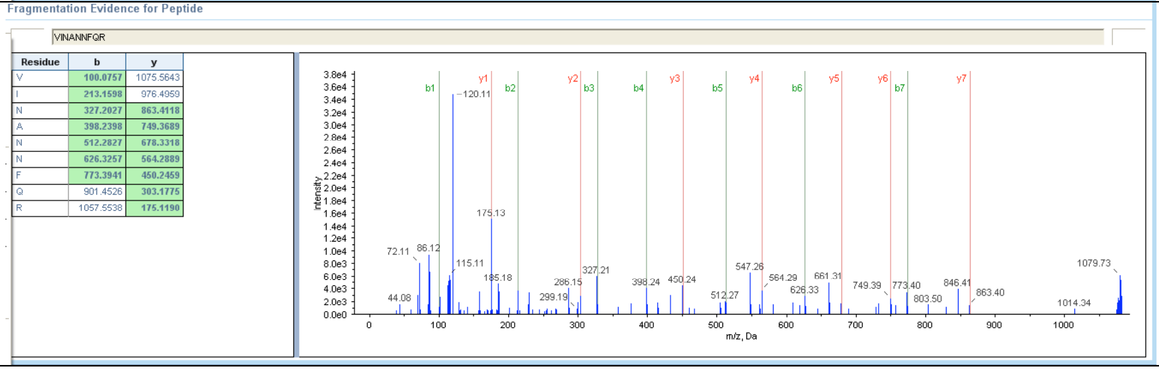

Residue	b	y
V	**100.0757**	1075.5643
I	**213.1598**	976.4959
N	**327.2027**	**863.4118**
A	**398.2398**	**749.3689**
N	**512.2827**	**678.3318**
N	**626.3257**	**564.2889**
F	**773.3941**	**450.2459**
Q	901.4526	**303.1775**
R	1057.5538	**175.1190**

216	964.5661	Fragmentation Evidence for Peptide GGVFDTIQK (see below)
217	2049.035	Fragmentation Evidence for Peptide ENNAILPTDSATYVGNTLR (see below)
218	1046.561	Fragmentation Evidence for Peptide GAIFPIEGSR (see below)

216 — 964.5661

Fragmentation Evidence for Peptide

GGVFDTIQK

Residue	b	y
G	58.0287	964.5098
G	115.0502	907.4884
V	214.1186	850.4669
F	361.1870	751.3985
D	476.2140	604.3301
T	577.2617	489.3031
I	690.3457	388.2554
Q	818.4043	275.1714
K	946.4993	147.1128

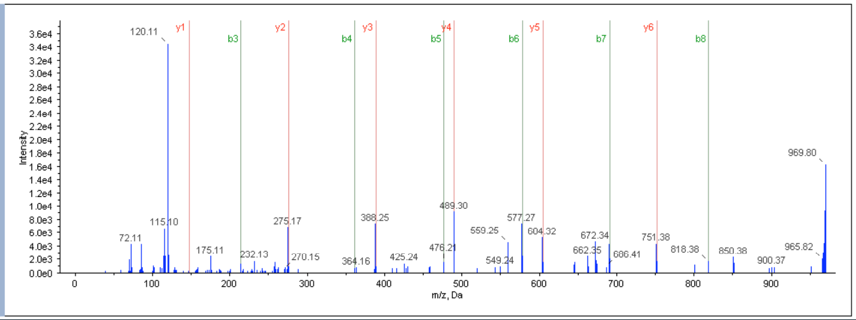

217 — 2049.035

Fragmentation Evidence for Peptide

ENNAILPTDSATYVGNTLR

Residue	b	y
E	130.0499	2049.0247
N	244.0928	1919.9821
N	358.1357	1805.9392
A	429.1728	1691.8963
I	542.2569	1620.8592
L	655.3410	1507.7751
P	752.3937	1394.6910
T	853.4414	1297.6383
D	968.4684	1196.5906
S	1055.5004	1081.5636
A	1126.5375	994.5316
T	1227.5852	923.4945
Y	1390.6485	822.4468
V	1489.7169	659.3835
G	1546.7384	560.3151
N	1660.7813	503.2936
T	1761.8290	389.2507
L	1874.9130	288.2030
R	2031.0142	175.1190

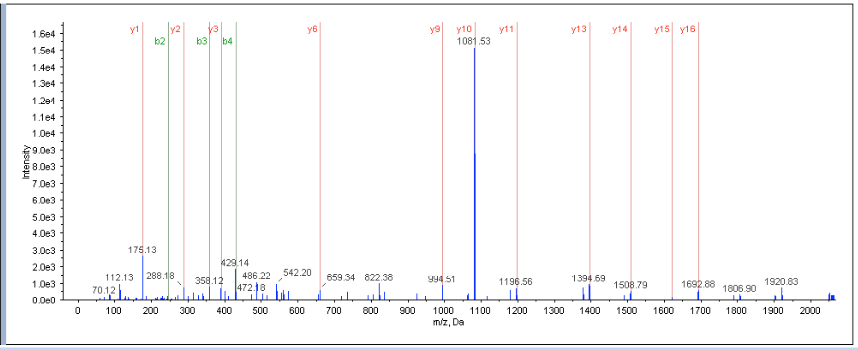

218 — 1046.561

Fragmentation Evidence for Peptide

GAIFPIEGSR

Residue	b	y
G	58.0287	1046.5629
A	129.0659	989.5415
I	242.1499	918.5043
F	389.2183	805.4203
P	486.2711	658.3519
I	599.3552	561.2991
E	728.3978	448.2150
G	785.4192	319.1724
S	872.4512	262.1510
R	1028.5524	175.1190

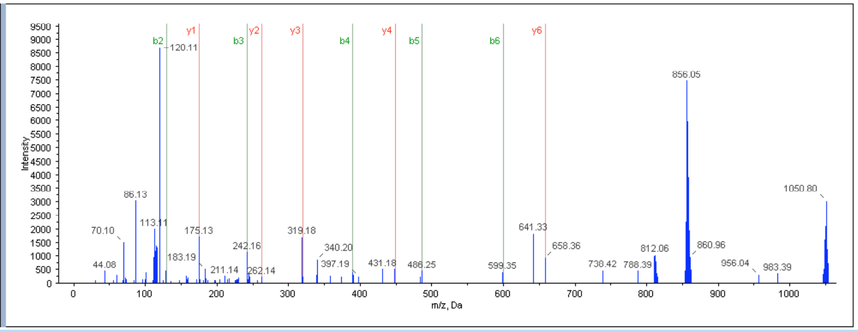

219	2078.067	

Fragmentation Evidence for Peptide

LMQEPVPGINAIPDENNAR

Residue	b	y
L	114.0913	2078.0335
M	**245.1318**	1964.9495
Q	**373.1904**	1833.9090
E	**502.2330**	1705.8504
P	599.2858	**1576.8078**
V	698.3542	1479.7550
P	795.4069	**1380.6866**
G	852.4284	1283.6339
I	**965.5125**	1226.6124
N	1079.5554	1113.5283
A	1150.5925	999.4854
I	1263.6766	928.4483
P	1360.7293	**815.3642**
D	1475.7563	718.3115
E	1604.7989	603.2845
N	1718.8418	474.2419
N	1832.8847	**360.1990**
A	1903.9218	**246.1561**
R	2060.0229	**175.1190**

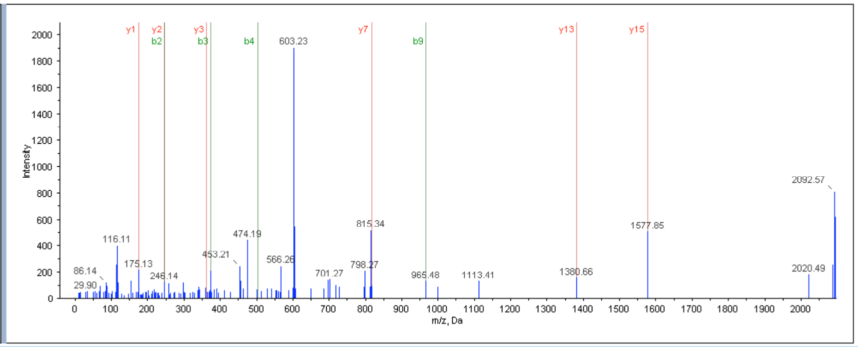

220	1884.942	

Fragmentation Evidence for Peptide

NLDPVIYKDVM[Oxi]SEFAK

Residue	b	y
N	115.0502	1884.9412
L	**228.1343**	1770.8982
D	**343.1612**	1657.8142
P	**440.2140**	1542.7872
V	**539.2824**	1445.7345
I	652.3665	**1346.6661**
Y	**815.4298**	**1233.5820**
K	943.5247	**1070.5187**
D	**1058.5517**	**942.4237**
V	**1157.6201**	827.3968
M[Oxi]	1304.6555	728.3284
S	1391.6875	581.2930
E	1520.7301	494.2609
F	1667.7985	365.2183
A	1738.8356	218.1499
K	1866.9306	147.1128

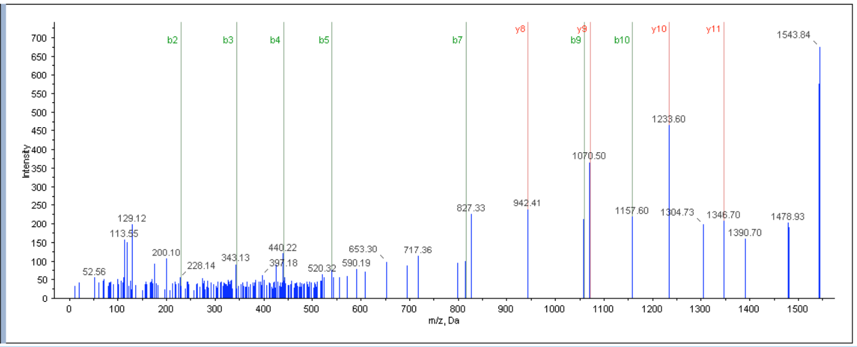

221	1811.954	

Fragmentation Evidence for Peptide

VGTKNLYLFDEAGKTR

Residue	b	y
V	100.0757	1811.9650
G	**157.0972**	1712.8966
T	258.1448	1655.8751
K	**386.2398**	1554.8275
N	**500.2827**	1426.7325
L	613.3668	1312.6896
Y	**776.4301**	1199.6055
L	889.5142	**1036.5422**
F	**1036.5826**	923.4581
D	1151.6095	**776.3897**
E	1280.6521	**661.3628**
A	1351.6892	**532.3202**
G	1408.7107	**461.2831**
K	1536.8057	**404.2616**
T	1637.8533	**276.1666**
R	1793.9545	**175.1190**

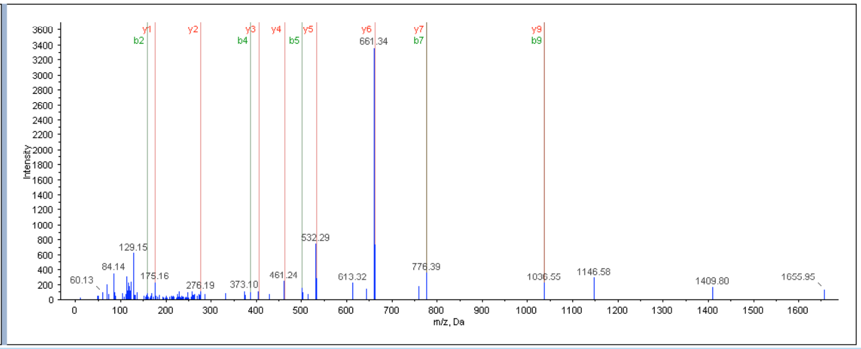

222	1312.659		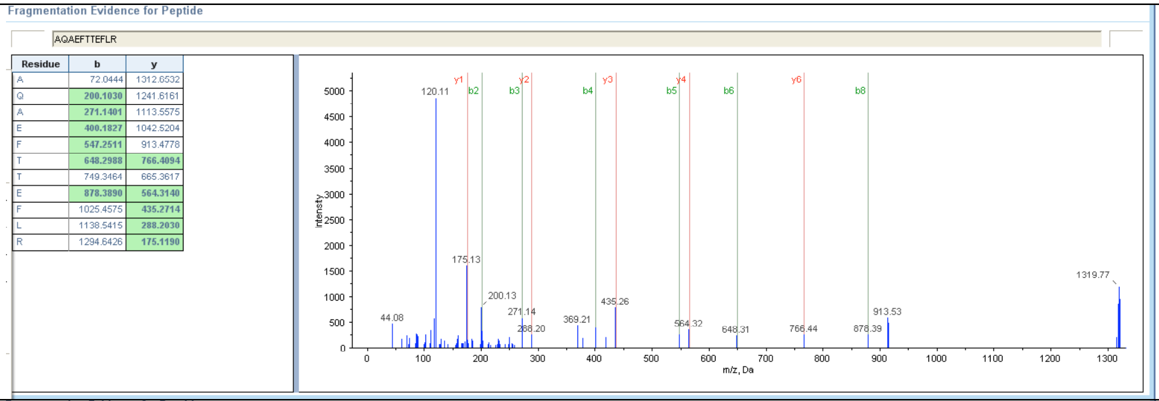
223	1418.683		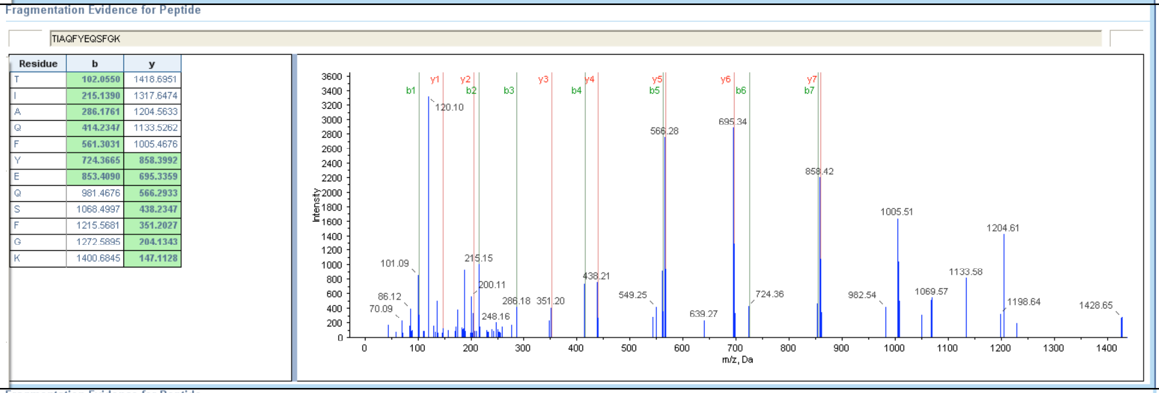
224	944.5371		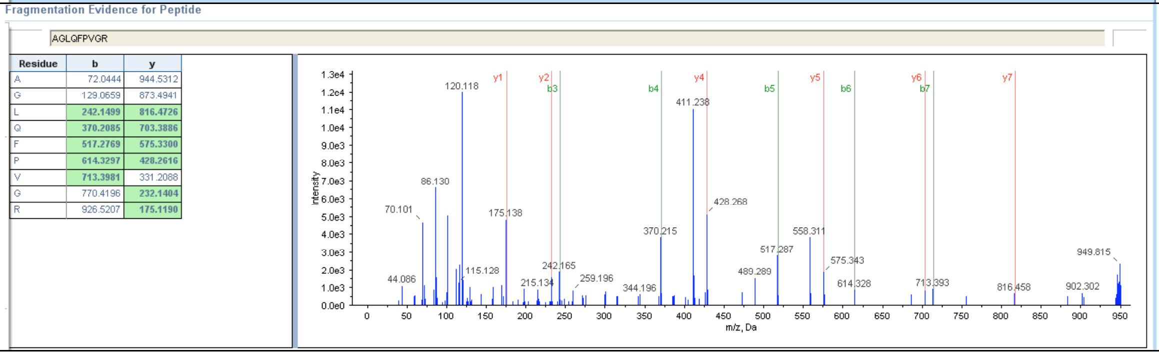

Fragmentation Evidence for Peptide

AQAEFTTEFLR

Residue	b	y
A	72.0444	1312.6532
Q	200.1030	1241.6161
A	271.1401	1113.5575
E	400.1827	1042.5204
F	547.2511	913.4778
T	648.2988	766.4094
T	749.3464	665.3617
E	878.3890	564.3140
F	1025.4575	435.2714
L	1138.5415	288.2030
R	1294.6426	175.1190

Fragmentation Evidence for Peptide

TIAQFYEQSFGK

Residue	b	y
T	102.0550	1418.6951
I	215.1390	1317.6474
A	286.1761	1204.5633
Q	414.2347	1133.5262
F	561.3031	1005.4676
Y	724.3665	858.3992
E	853.4090	695.3359
Q	981.4676	566.2933
S	1068.4997	438.2347
F	1215.5681	351.2027
G	1272.5895	204.1343
K	1400.6845	147.1128

Fragmentation Evidence for Peptide

AGLQFPVGR

Residue	b	y
A	72.0444	944.5312
G	129.0659	873.4941
L	242.1499	816.4726
Q	370.2085	703.3886
F	517.2769	575.3300
P	614.3297	428.2616
V	713.3981	331.2088
G	770.4196	232.1404
R	926.5207	175.1190

225	1834.84		

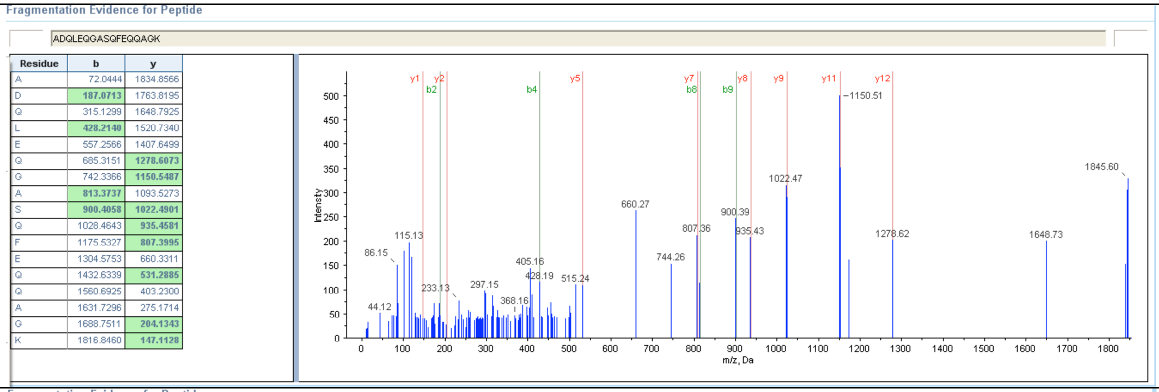

Fragmentation Evidence for Peptide

ADQLEQGASQFEQQAGK

Residue	b	y
A	72.0444	1834.8566
D	**187.0713**	1763.8195
Q	315.1299	1648.7925
L	**428.2140**	1520.7340
E	557.2566	1407.6499
Q	685.3151	**1278.6073**
G	742.3366	**1150.5487**
A	**813.3737**	1093.5273
S	**900.4058**	**1022.4901**
Q	1028.4643	**935.4581**
F	1175.5327	**807.3995**
E	1304.5753	660.3311
Q	1432.6339	**531.2885**
Q	1560.6925	403.2300
A	1631.7296	275.1714
G	1688.7511	**204.1343**
K	1816.8460	**147.1128**

226	2556.183		

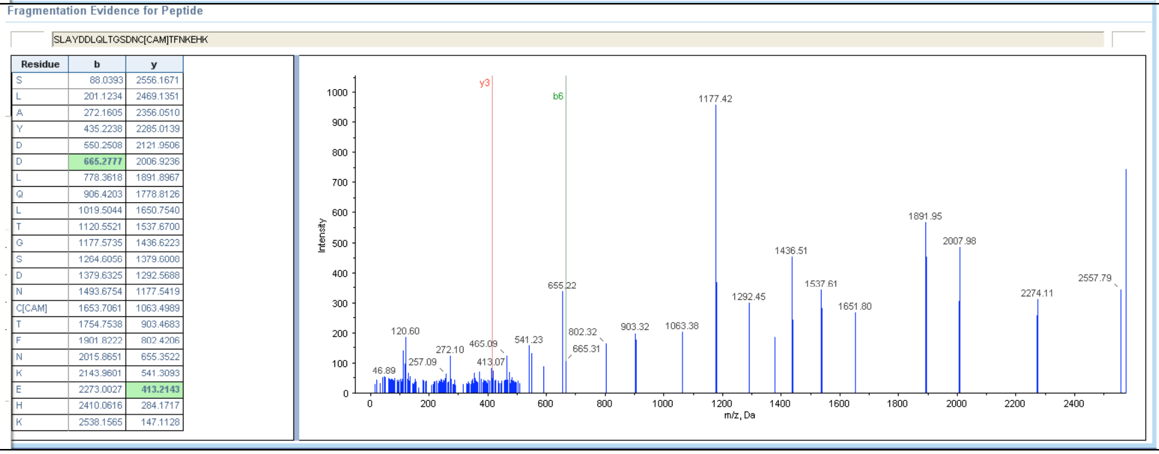

Fragmentation Evidence for Peptide

SLAYDDLQLTGSDNC[CAM]TFNKEHK

Residue	b	y
S	88.0393	2556.1671
L	201.1234	2469.1351
A	272.1605	2356.0510
Y	435.2238	2285.0139
D	550.2508	2121.9506
D	**665.2777**	2006.9236
L	778.3618	1891.8967
Q	906.4203	1778.8126
L	1019.5044	1650.7540
T	1120.5521	1537.6700
G	1177.5735	1436.6223
S	1264.6056	1379.6008
D	1379.6325	1292.5688
N	1493.6754	1177.5419
C[CAM]	1653.7061	1063.4989
T	1754.7538	903.4683
F	1901.8222	802.4206
N	2015.8651	655.3522
K	2143.9601	541.3093
E	2273.0027	**413.2143**
H	2410.0616	284.1717
K	2538.1565	147.1128

227	1832.812		Fragmentation Evidence for Peptide: NLATQFNC[CAM]AFMETSAK

Fragmentation Evidence for Peptide

NLATQFNC[CAM]AFMETSAK

Residue	b	y
N	115.0502	1832.8306
L	**228.1343**	1718.7877
A	**299.1714**	1605.7036
T	400.2191	**1534.6665**
Q	528.2776	1433.6188
F	**675.3461**	1305.5602
N	789.3890	**1158.4918**
C[CAM]	949.4196	**1044.4489**
A	1020.4567	**884.4182**
F	1167.5252	**813.3811**
M	1298.5656	**666.3127**
E	1427.6082	**535.2722**
T	1528.6559	**406.2296**
S	1615.6879	305.1819
A	1686.7251	218.1499
K	1814.8200	147.1128

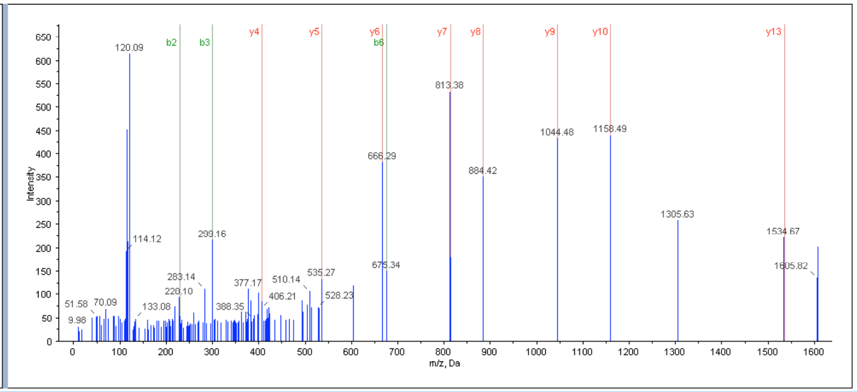

228	1232.649		Fragmentation Evidence for Peptide: TVEPFALNWR

Fragmentation Evidence for Peptide

TVEPFALNWR

Residue	b	y
T	**102.0550**	1232.6422
V	**201.1234**	**1131.5946**
E	**330.1660**	1032.5261
P	**427.2187**	903.4835
F	**574.2871**	806.4308
A	**645.3243**	**659.3624**
L	758.4083	**588.3253**
N	872.4512	**475.2412**
W	1058.5306	**361.1983**
R	1214.6317	**175.1190**

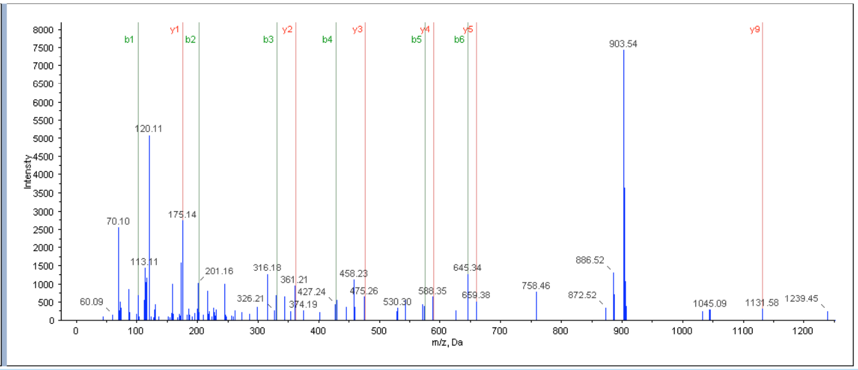

<table>
<tr>
<td>229</td>
<td>1681.873</td>
<td>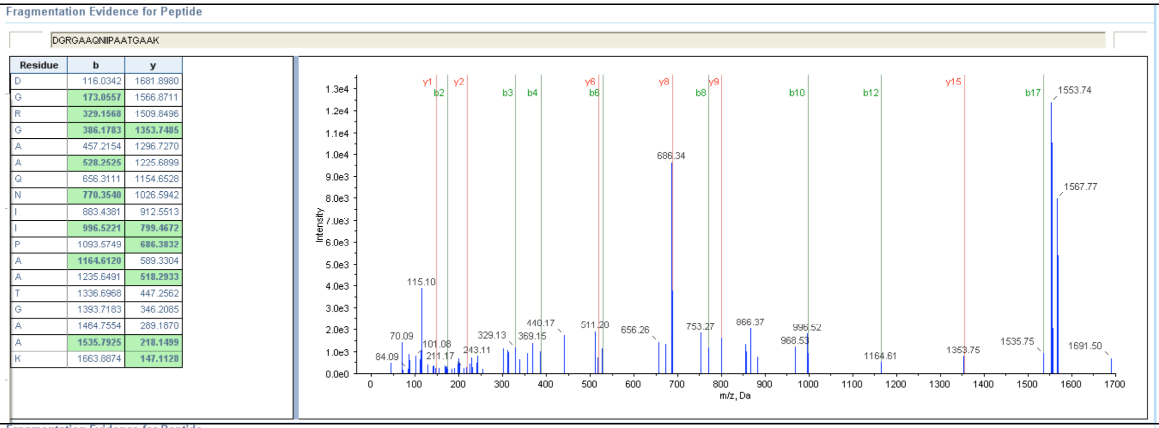
</td>
</tr>
<tr>
<td>230</td>
<td>1232.665</td>
<td>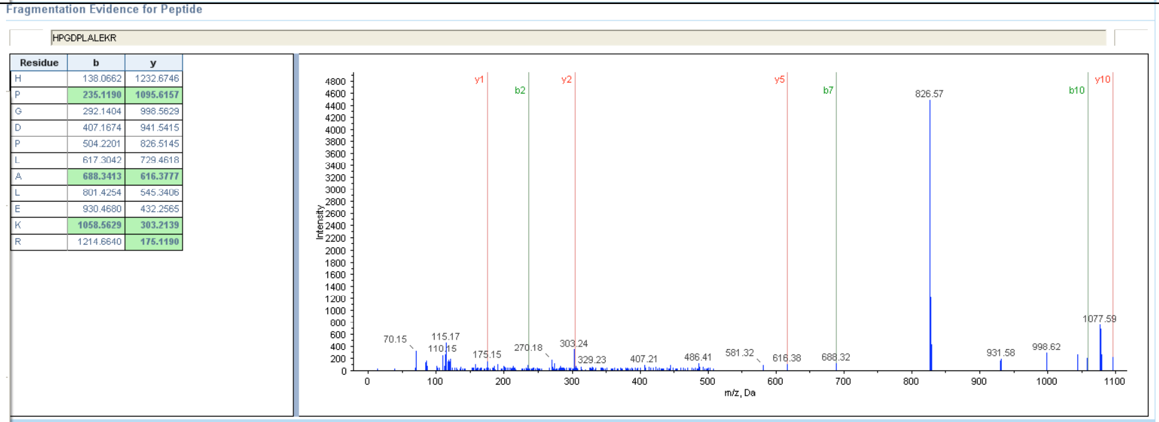
</td>
</tr>
<tr>
<td>231</td>
<td>1229.688</td>
<td>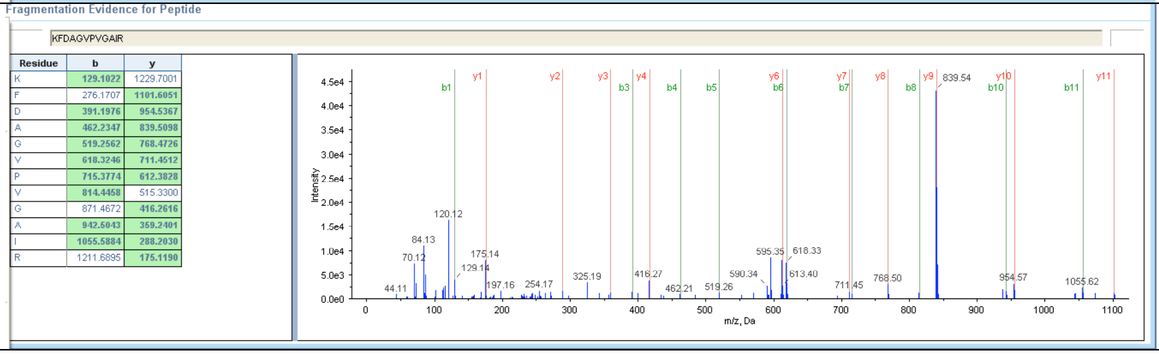
</td>
</tr>
</table>

232	1869.059	Fragmentation Evidence for Peptide

TVPLSVEKQDDGKLLVK

Residue	b	y
T	102.0550	1869.0692
V	**201.1234**	1768.0215
P	**298.1761**	1668.9531
L	411.2602	1571.9003
S	498.2922	1458.8162
V	597.3606	1371.7842
E	726.4032	**1272.7158**
K	854.4982	1143.6732
Q	**982.5568**	**1015.5782**
D	**1097.5837**	**887.5197**
D	**1212.6107**	772.4927
G	1269.6321	657.4658
K	**1397.7271**	600.4443
L	**1510.8111**	472.3493
L	1623.8952	359.2653
V	**1722.9636**	**246.1812**
K	1851.0586	**147.1128**

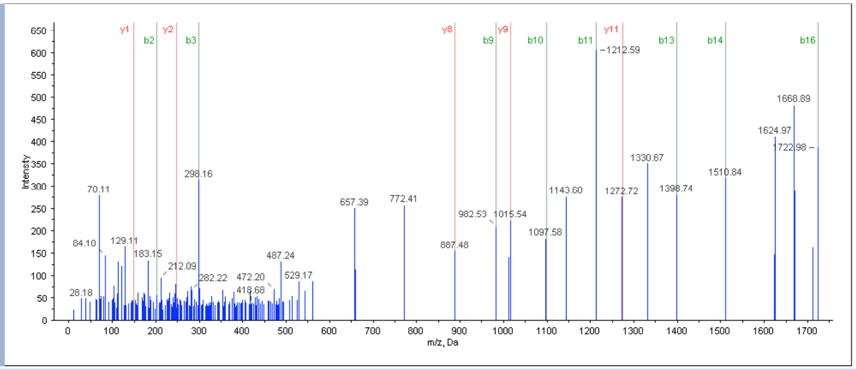

233	1715.784	Fragmentation Evidence for Peptide

GLDPDQHYEATVQSR

Residue	b	y
G	58.0287	1715.7984
L	**171.1128**	1658.7769
D	286.1397	1545.6928
P	**383.1925**	**1430.6659**
D	498.2195	1333.6131
Q	626.2780	**1218.5862**
H	763.3369	**1090.5276**
Y	**926.4003**	953.4687
E	1055.4429	**790.4054**
A	1126.4800	**661.3628**
T	1227.5277	**590.3257**
V	1326.5961	**489.2780**
Q	1454.6546	390.2096
S	1541.6867	**262.1510**
R	1697.7878	**175.1190**

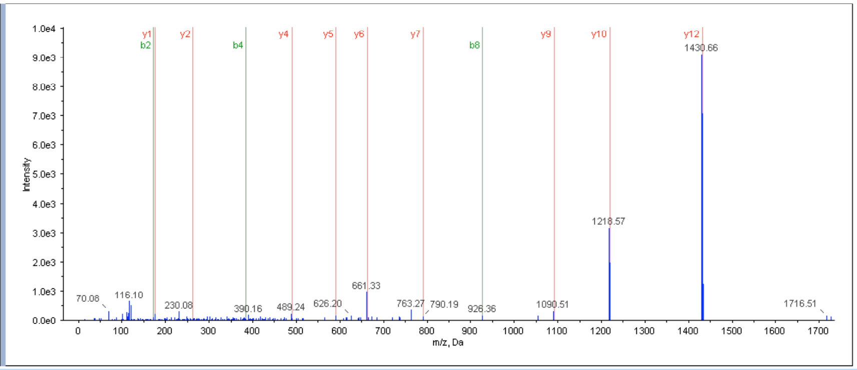

234	1233.641	Fragmentation Evidence for Peptide

1Ac TDSKYFTTTK

Residue	b	y
T	144.0655	1233.5998
D	259.0925	1090.5415
S	346.1245	975.5146
K	474.2195	888.4825
Y	**637.2828**	**760.3876**
F	**784.3512**	**597.3243**
T	885.3989	**450.2558**
T	986.4466	349.2082
T	1087.4942	248.1605
K	1215.5892	147.1128

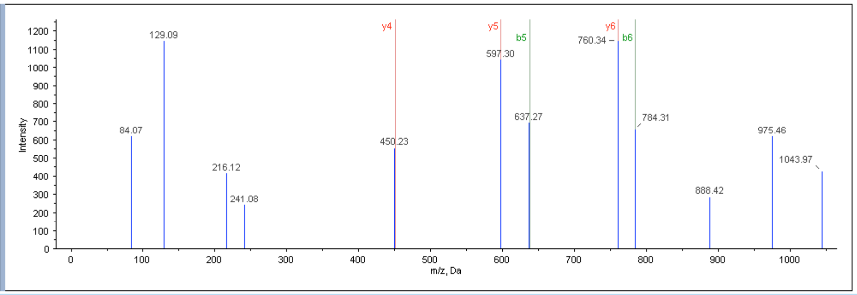

235	1239.646	

Fragmentation Evidence for Peptide

FKVDTEGSSNR

Residue	b	y
F	148.0757	1239.5964
K	**276.1707**	1092.5280
V	**375.2391**	964.4330
D	490.2660	**865.3646**
T	591.3137	**750.3377**
E	720.3563	**649.2900**
G	777.3777	**520.2474**
S	864.4098	463.2259
S	951.4418	**376.1939**
N	**1065.4847**	**289.1619**
R	1221.5858	**175.1190**

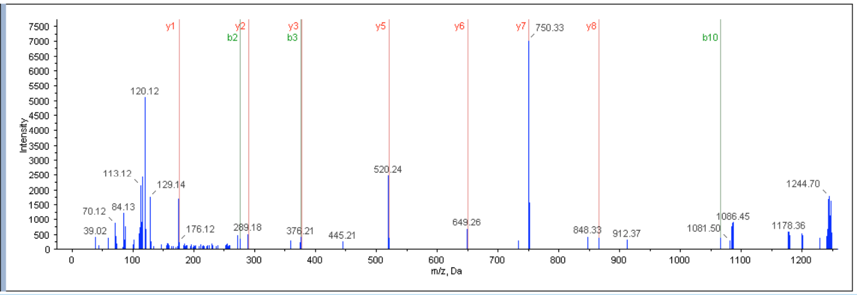

236	1681.826	

Fragmentation Evidence for Peptide

SQQGPLTVHC[CAM]SPGTGR

Residue	b	y
S	88.0393	1681.8075
Q	**216.0979**	1594.7754
Q	**344.1565**	1466.7169
G	**401.1779**	**1338.6583**
P	498.2307	1281.6368
L	611.3148	1184.5841
T	712.3624	1071.5000
V	811.4308	970.4523
H	948.4898	871.3839
C[CAM]	1108.5204	734.3250
S	**1195.5524**	574.2944
P	1292.6052	487.2623
G	1349.6267	390.2096
T	1450.6743	333.1881
G	1507.6958	**232.1404**
R	1663.7969	**175.1190**

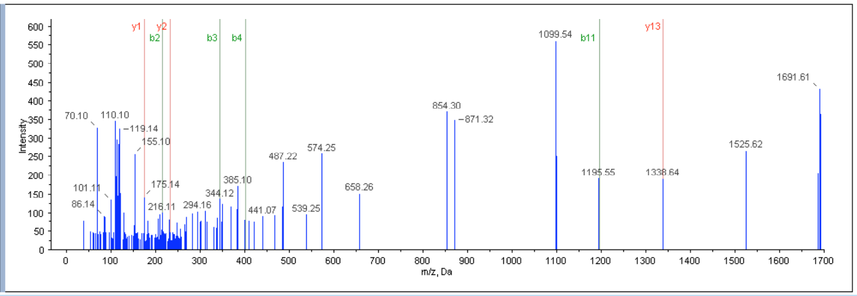

237	974.6174	

Fragmentation Evidence for Peptide

IYPISIIR

Residue	b	y
I	114.0913	974.6033
Y	**277.1547**	861.5193
P	**374.2074**	698.4559
I	**487.2915**	601.4032
S	574.3235	**488.3191**
I	68[illegible]076	**401.2871**
I	800.4917	**288.2030**
R	956.5928	**175.1190**

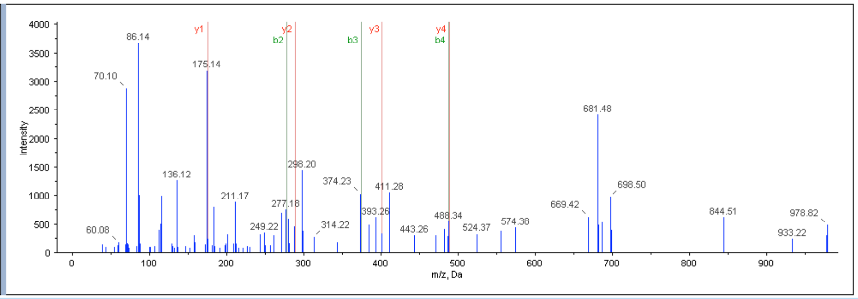

238	1857.868		Fragmentation Evidence for Peptide

VVYAPSDEWYDETKR

Residue	b	y
V	100.0757	1857.8654
V	**199.1441**	1758.7970
Y	**362.2074**	1659.7285
A	433.2445	1496.6652
P	530.2973	1425.6281
S	617.3293	1328.5753
D	732.3563	1241.5433
E	861.3989	**1126.5164**
W	1047.4782	**997.4738**
Y	1210.5415	811.3945
D	1325.5685	648.3311
E	1454.6111	**533.3042**
T	1555.6587	**404.2616**
K	1683.7537	**303.2139**
R	1839.8548	**175.1190**

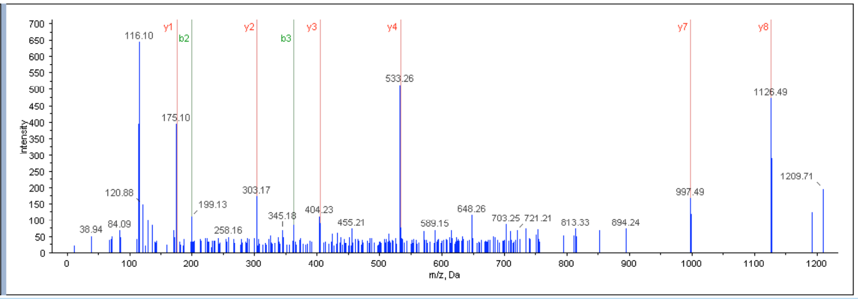

239	1772.817		Fragmentation Evidence for Peptide

GDEYTLPSAPLGFSYR

Residue	b	y
G	58.0287	1772.8490
D	173.0557	1715.8275
E	**302.0983**	**1600.8006**
Y	465.1616	**1471.7580**
T	566.2093	1308.6947
L	679.2933	**1207.6470**
P	776.3461	**1094.5629**
S	863.3781	997.5102
A	934.4153	910.4781
P	1031.4680	**839.4410**
L	1144.5521	742.3883
G	1201.5735	629.3042
F	1348.6420	572.2827
S	1435.6740	425.2143
Y	1598.7373	338.1823
R	1754.8384	**175.1190**

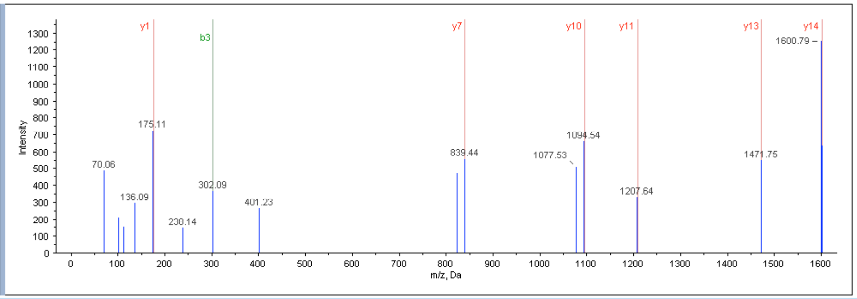

240	966.4969		Fragmentation Evidence for Peptide

QILC[CAM]YNR

Residue	b	y
Q	129.0659	966.4826
I	**242.1499**	838.4240
L	**355.2340**	**725.3399**
C[CAM]	**515.2646**	**612.2559**
Y	**678.3280**	**452.2252**
N	792.3709	**289.1619**
R	948.4720	**175.1190**

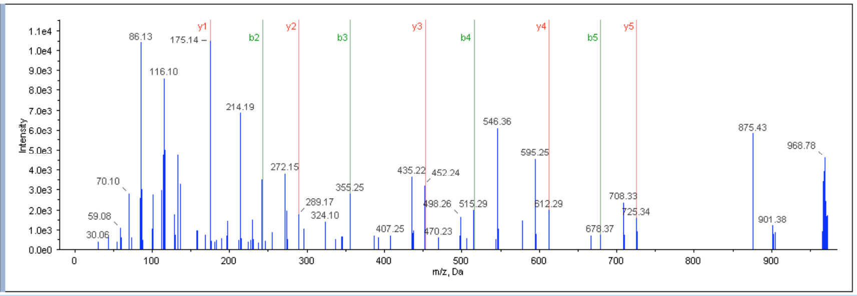

<table>
<tr>
<td>241</td>
<td>1739.84</td>
<td></td>
<td>
Fragmentation Evidence for Peptide
KGQEFFADPPRVDAY
<table>
<tr><th>Residue</th><th>b</th><th>y</th></tr>
<tr><td>K</td><td>129.1022</td><td>1739.8388</td></tr>
<tr><td>G</td><td>186.1237</td><td>1611.7438</td></tr>
<tr><td>Q</td><td>314.1823</td><td>1554.7223</td></tr>
<tr><td>E</td><td>443.2249</td><td>1426.6638</td></tr>
<tr><td>F</td><td>590.2933</td><td>1297.6212</td></tr>
<tr><td>F</td><td>737.3617</td><td>1150.5527</td></tr>
<tr><td>A</td><td>808.3988</td><td>1003.4843</td></tr>
<tr><td>D</td><td>923.4258</td><td>932.4472</td></tr>
<tr><td>P</td><td>1020.4785</td><td>817.4203</td></tr>
<tr><td>P</td><td>1117.5313</td><td>720.3675</td></tr>
<tr><td>R</td><td>1273.6324</td><td>623.3148</td></tr>
<tr><td>V</td><td>1372.7008</td><td>467.2136</td></tr>
<tr><td>D</td><td>1487.7278</td><td>368.1452</td></tr>
<tr><td>A</td><td>1558.7649</td><td>253.1183</td></tr>
<tr><td>Y</td><td>1721.8282</td><td>182.0812</td></tr>
</table>
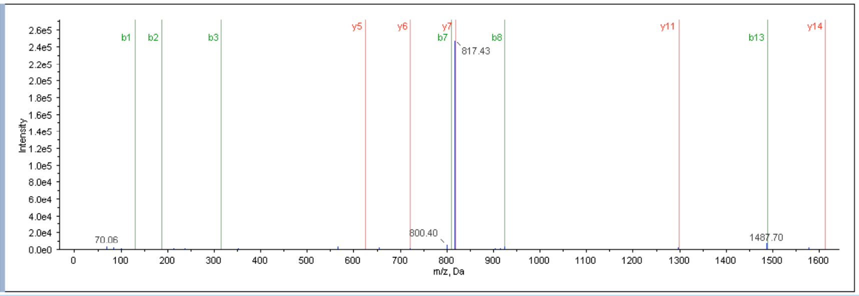

</td>
</tr>
<tr>
<td>242</td>
<td>1311.698</td>
<td></td>
<td>
Fragmentation Evidence for Peptide
YIETYLPKER
<table>
<tr><th>Residue</th><th>b</th><th>y</th></tr>
<tr><td>Y</td><td>164.0706</td><td>1311.6943</td></tr>
<tr><td>I</td><td>277.1547</td><td>1148.6310</td></tr>
<tr><td>E</td><td>406.1973</td><td>1035.5469</td></tr>
<tr><td>T</td><td>507.2449</td><td>906.5043</td></tr>
<tr><td>Y</td><td>670.3083</td><td>805.4567</td></tr>
<tr><td>L</td><td>783.3923</td><td>642.3933</td></tr>
<tr><td>P</td><td>880.4451</td><td>529.3093</td></tr>
<tr><td>K</td><td>1008.5401</td><td>432.2565</td></tr>
<tr><td>E</td><td>1137.5827</td><td>304.1615</td></tr>
<tr><td>R</td><td>1293.6838</td><td>175.1190</td></tr>
</table>
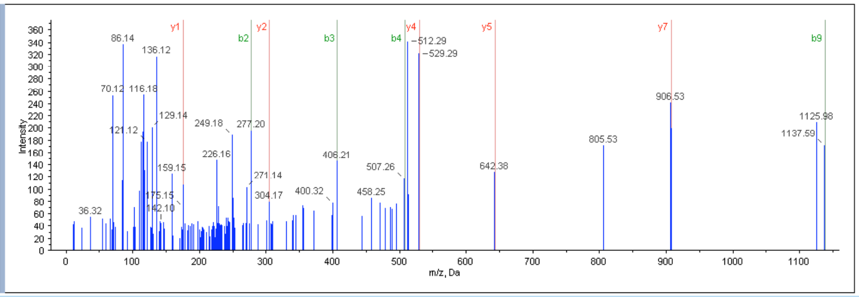

</td>
</tr>
<tr>
<td>243</td>
<td>1645.837</td>
<td></td>
<td>
Fragmentation Evidence for Peptide
AIEDQTKEELDKAR
<table>
<tr><th>Residue</th><th>b</th><th>y</th></tr>
<tr><td>A</td><td>72.0444</td><td>1645.8392</td></tr>
<tr><td>I</td><td>185.1285</td><td>1574.8020</td></tr>
<tr><td>E</td><td>314.1710</td><td>1461.7180</td></tr>
<tr><td>D</td><td>429.1980</td><td>1332.6754</td></tr>
<tr><td>Q</td><td>557.2566</td><td>1217.6484</td></tr>
<tr><td>T</td><td>658.3042</td><td>1089.5899</td></tr>
<tr><td>K</td><td>786.3992</td><td>988.5422</td></tr>
<tr><td>E</td><td>915.4418</td><td>860.4472</td></tr>
<tr><td>E</td><td>1044.4844</td><td>731.4046</td></tr>
<tr><td>L</td><td>1157.5685</td><td>602.3620</td></tr>
<tr><td>D</td><td>1272.5954</td><td>489.2780</td></tr>
<tr><td>K</td><td>1400.6904</td><td>374.2510</td></tr>
<tr><td>A</td><td>1471.7275</td><td>246.1561</td></tr>
<tr><td>R</td><td>1627.8286</td><td>175.1190</td></tr>
</table>
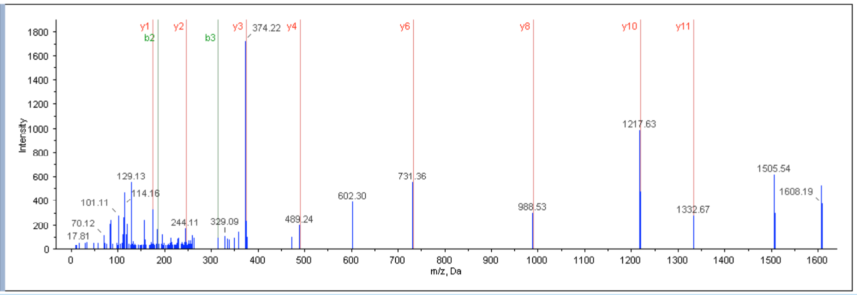

</td>
</tr>
</table>

244	1194.587	

Fragmentation Evidence for Peptide

R[Oxi]TKSGAMQDGK

Residue	b	y
R[Oxi]	**173.1033**	1194.5895
T	274.1510	**1022.4935**
K	402.2459	921.4458
S	489.2780	793.3509
G	546.2994	706.3189
A	**617.3365**	649.2974
M	**748.3770**	**578.2603**
Q	**876.4356**	**447.2198**
D	**991.4626**	319.1612
G	**1048.4840**	**204.1343**
K	1176.5790	**147.1128**

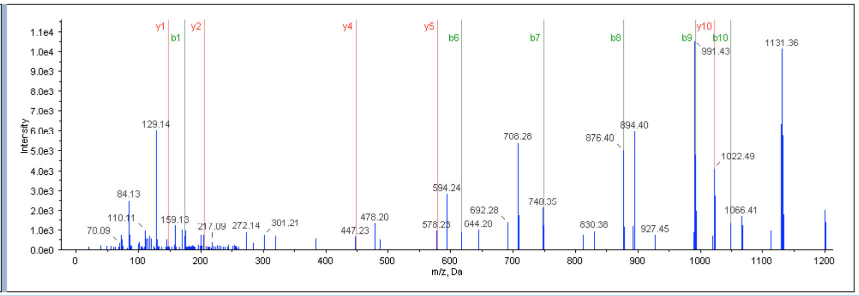

245	2200.036	

Fragmentation Evidence for Peptide

NVSPEDSDDYYC[CAM]EILPQR

Residue	b	y
N	**115.0502**	2199.9499
V	**214.1186**	2085.9070
S	**301.1506**	1986.8386
P	398.2034	1899.8065
E	527.2460	1802.7538
D	642.2729	1673.7112
S	729.3050	**1558.6842**
D	844.3319	1471.6522
D	959.3589	**1356.6253**
Y	1122.4222	**1241.5983**
Y	1285.4855	**1078.5350**
C[CAM]	1445.5162	915.4717
E	1574.5588	**755.4410**
I	1687.6428	**626.3984**
L	1800.7269	**513.3144**
P	1897.7797	**400.2303**
Q	2025.8382	**303.1775**
R	2181.9393	**175.1190**

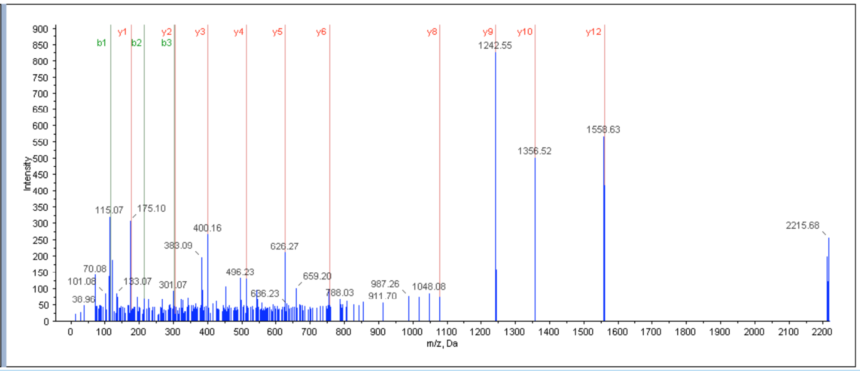

246	943.4237	

Fragmentation Evidence for Peptide

FGEFC[CAM]QR

Residue	b	y
F	148.0757	943.4091
G	**205.0972**	**796.3406**
E	**334.1397**	**739.3192**
F	**481.2082**	**610.2766**
C[CAM]	**641.2388**	**463.2082**
Q	769.2974	**303.1775**
R	925.3985	**175.1190**

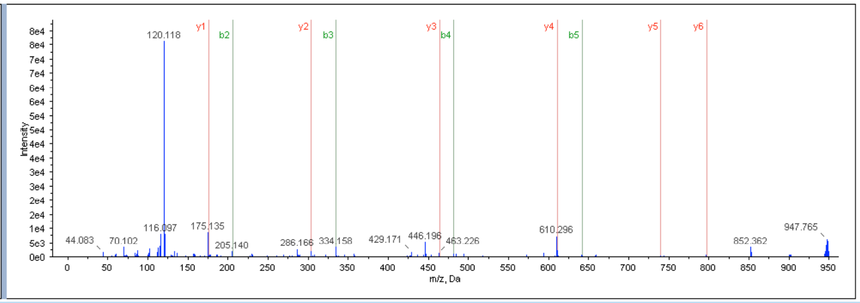

247	1437.722		Fragmentation Evidence for Peptide

NNEQRQDFVC[CAM]K

Residue	b	y
N	115.0502	1437.6539
N	229.0931	1323.6110
E	358.1357	1209.5681
Q	486.1943	1080.5255
R	642.2954	952.4669
Q	770.3540	796.3658
D	885.3809	668.3072
F	1032.4493	553.2803
V	1131.5178	406.2119
C[CAM]	1291.5484	307.1435
K	1419.6434	147.1128

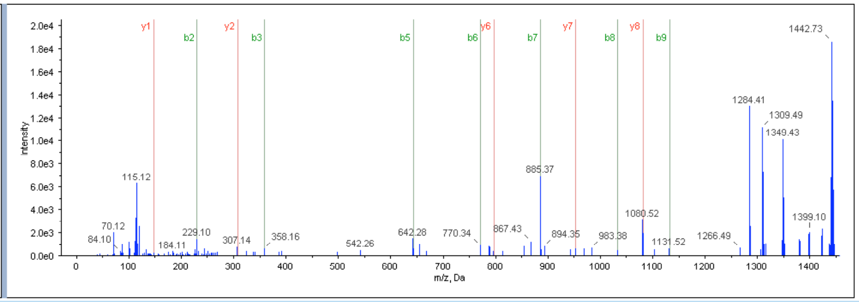

248	1033.504		Fragmentation Evidence for Peptide

FWAPPMER

Residue	b	y
F	148.0757	1033.4924
W	334.1550	886.4240
A	405.1921	700.3447
P	502.2449	629.3076
P	599.2976	532.2548
M	730.3381	435.2020
E	859.3807	304.1615
R	1015.4818	175.1190

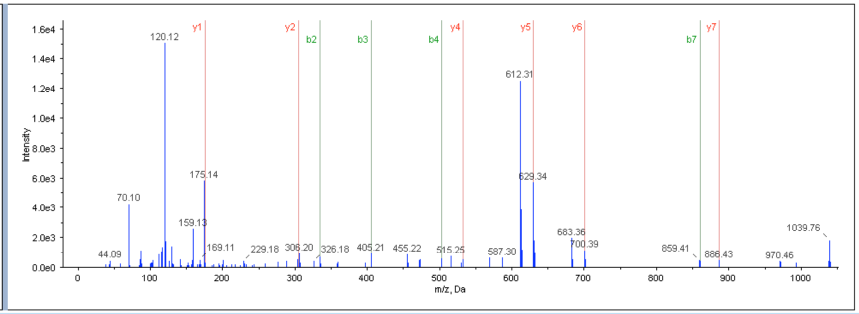

249	1125.579		Fragmentation Evidence for Peptide

VDLNHFQPR

Residue	b	y
V	100.0757	1125.5800
D	215.1026	1026.5116
L	328.1867	911.4846
N	442.2296	798.4005
H	579.2885	684.3576
F	726.3570	547.2987
Q	854.4155	400.2303
P	951.4683	272.1717
R	1107.5694	175.1190

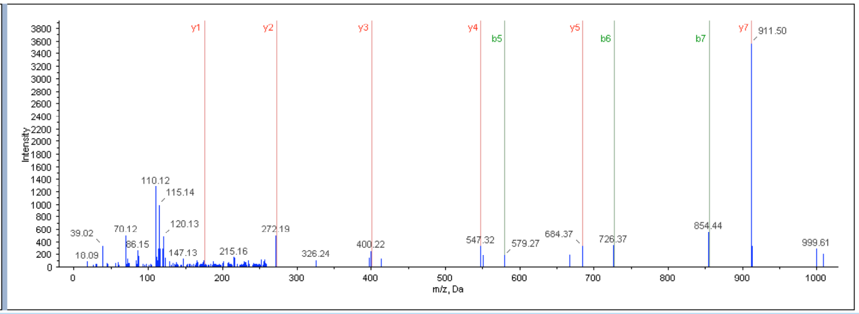

250

1186.563

Fragmentation Evidence for Peptide

PGQ QSSKNAVQ[Dea]GKR

Residue	b	y
Q	112.0393	1186.6175
S	199.0713	1075.5854
S	286.1034	988.5534
K	414.1983	901.5214
N	**528.2413**	773.4264
A	**599.2784**	659.3835
V	**698.3468**	588.3464
Q[Dea]	827.3894	**489.2780**
G	**884.4108**	360.2354
K	1012.5058	**303.2139**
R	1168.6069	175.1190

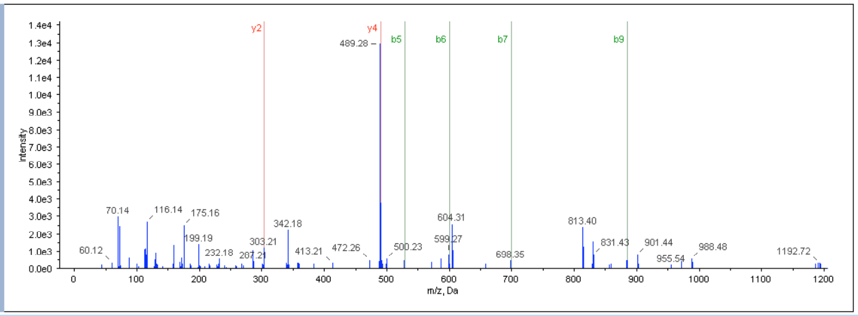

251

1612.737

Fragmentation Evidence for Peptide

SWTEYDLSHYGVR

Residue	b	y
S	88.0393	1612.7390
W	**274.1186**	1525.7070
T	375.1663	1339.6277
E	504.2089	1238.5800
Y	667.2722	**1109.5374**
D	782.2992	**946.4741**
L	895.3832	**831.4472**
S	982.4153	**718.3631**
H	1119.4742	**631.3311**
Y	1282.5375	**494.2722**
G	1339.5590	**331.2088**
V	**1438.6274**	274.1874
R	1594.7285	**175.1190**

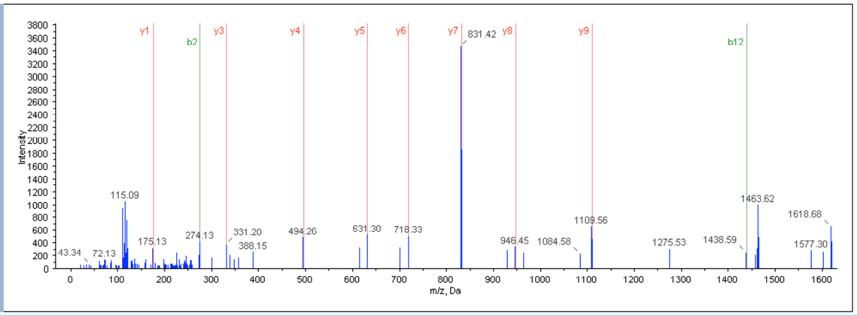

252

2369.971

Fragmentation Evidence for Peptide

VFSETQNNMEENDDHVGYGGK

Residue	b	y
V	100.0757	2369.9939
F	247.1441	2270.9255
S	**334.1761**	2123.8571
E	**463.2187**	2036.8250
T	564.2664	1907.7824
Q	692.3250	1806.7348
N	806.3679	1678.6762
N	920.4108	1564.6333
M	1051.4513	**1450.5903**
E	1180.4939	1319.5498
E	1309.5365	1190.5073
N	1423.5794	1061.4647
D	1538.6064	**947.4217**
D	1653.6333	832.3948
H	**1790.6922**	717.3678
V	1889.7606	580.3089
G	1946.7821	481.2405
Y	2109.8454	424.2191
G	2166.8669	**261.1557**
G	2223.8884	204.1343
K	2351.9833	147.1128

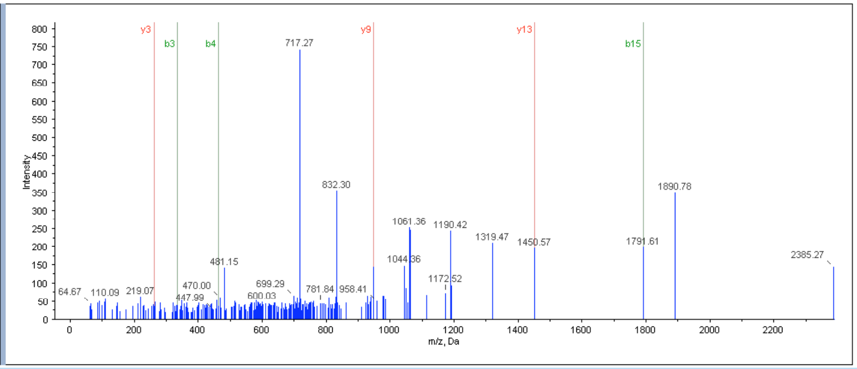

253	2201.034	

Fragmentation Evidence for Peptide

LQNSVQDLSSFDEYATEVR

Residue	b	y
L	114.0913	2201.0357
Q	**242.1499**	2087.9516
N	356.1928	1959.8930
S	443.2249	1845.8501
V	542.2933	1758.8181
Q	670.3519	1659.7497
D	785.3788	1531.6911
L	898.4629	**1416.6642**
S	985.4949	1303.5801
S	1072.5269	**1216.5481**
F	1219.5953	1129.5160
D	1334.6223	982.4476
E	1463.6649	867.4207
Y	1626.7282	**738.3781**
A	1697.7653	575.3148
T	1798.8130	**504.2776**
E	1927.8556	403.2300
V	2026.9240	**274.1874**
R	2183.0251	**175.1190**

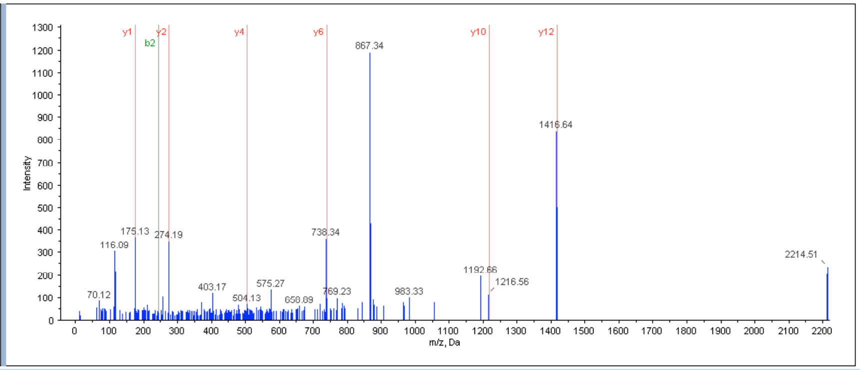

254	1910	

Fragmentation Evidence for Peptide

AAADATKPEQQLAAVQAAR

Residue	b	y
A	**72.0444**	1910.0090
A	**143.0815**	1838.9719
A	**214.1186**	1767.9348
D	**329.1456**	1696.8977
A	400.1827	**1581.8707**
T	501.2304	1510.8336
K	629.3253	1409.7859
P	726.3781	**1281.6910**
E	855.4207	1184.6382
Q	983.4793	**1055.5956**
Q	1111.5378	927.5370
L	1224.6219	**799.4785**
A	1295.6590	686.3944
A	1366.6961	615.3573
V	1465.7645	544.3202
Q	1593.8231	445.2518
A	1664.8602	**317.1932**
A	1735.8973	**246.1561**
R	1891.9984	**175.1190**

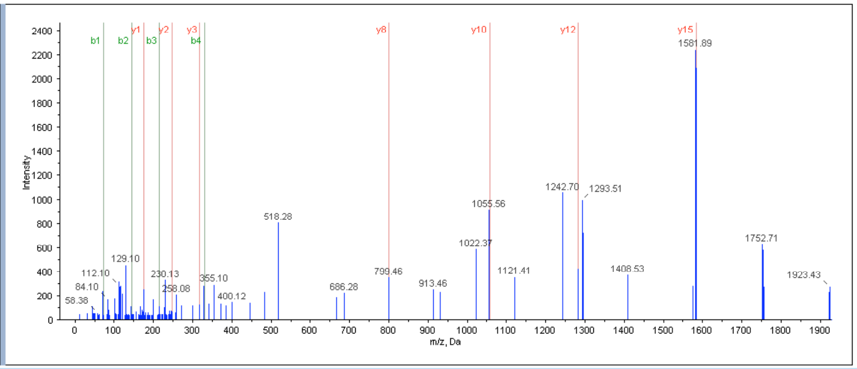

255	2103.852		

Fragmentation Evidence for Peptide

AMEAGMDGGDTTEMSSNPFR

Residue	b	y
A	72.0444	2103.8416
M	**203.0849**	2032.8045
E	**332.1275**	1901.7640
A	403.1646	1772.7214
G	**460.1860**	1701.6843
M	591.2265	1644.6628
D	706.2535	1513.6224
G	763.2749	**1398.5954**
G	**820.2964**	1341.5740
D	935.3233	1284.5525
T	1036.3710	**1169.5255**
T	1137.4187	1068.4779
E	1266.4613	967.4302
M	1397.5018	838.3876
S	1484.5338	707.3471
S	1571.5658	**620.3151**
N	1685.6088	**533.2831**
P	1782.6615	**419.2401**
F	1929.7299	322.1874
R	2085.8311	**175.1190**

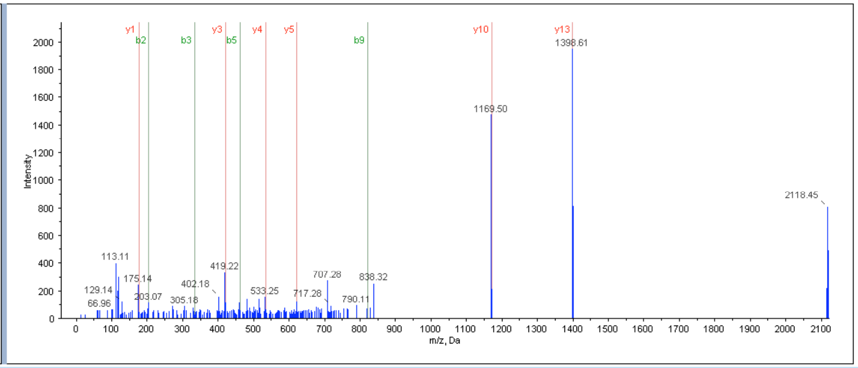

256	849.4479		

Fragmentation Evidence for Peptide

SPYEVVR

Residue	b	y
S	88.0393	849.4465
P	**185.0921**	762.4145
Y	**348.1554**	**665.3617**
E	**477.1980**	502.2984
V	**576.2664**	**373.2558**
V	**675.3348**	**274.1874**
R	831.4359	**175.1190**

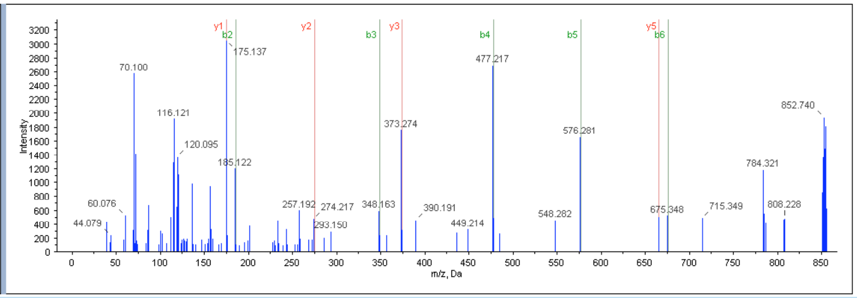

257	936.4551		

Fragmentation Evidence for Peptide

HSFSYAPK

Residue	b	y
H	138.0662	936.4574
S	225.0982	**799.3985**
F	372.1666	712.3665
S	**459.1987**	565.2980
Y	**622.2620**	**478.2660**
A	693.2991	315.2027
P	790.3519	244.1656
K	918.4468	147.1128

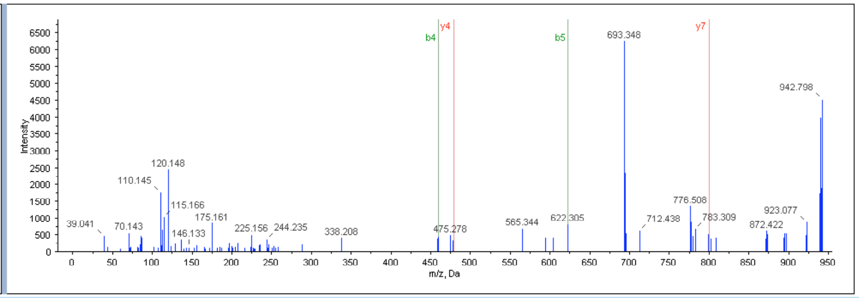

258	1663.824		

Fragmentation Evidence for Peptide

AAGARYEDIPVTNMR

Residue	b	y
A	72.0444	1663.8221
A	143.0815	1592.7850
G	**200.1030**	1521.7478
A	271.1401	1464.7264
R	427.2412	1393.6893
Y	590.3045	1237.5881
E	**719.3471**	1074.5248
D	**834.3741**	**945.4822**
I	**947.4581**	**830.4553**
P	1044.5109	**717.3712**
V	1143.5793	620.3185
T	1244.6270	521.2500
N	1358.6699	420.2024
M	1489.7104	306.1594
R	1645.8115	175.1190

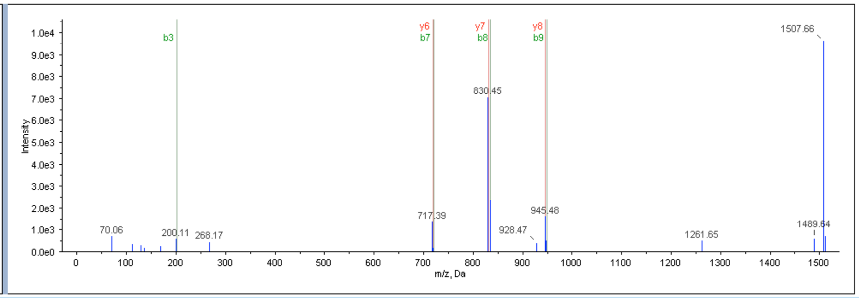

259	853.4534		

Fragmentation Evidence for Peptide

FQFGVAGK

Residue	b	y
F	148.0757	853.4567
Q	**276.1343**	**706.3883**
F	**423.2027**	578.3297
G	**480.2241**	**431.2613**
V	579.2926	**374.2398**
A	**650.3297**	275.1714
G	**707.3511**	**204.1343**
K	835.4461	**147.1128**

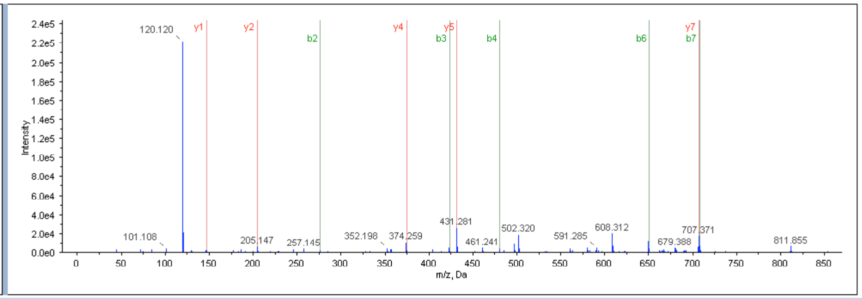

260	1134.637		

Fragmentation Evidence for Peptide

QVAQYIEKR

Residue	b	y
Q	129.0659	1134.6266
V	**228.1343**	1006.5680
A	**299.1714**	**907.4996**
Q	**427.2300**	836.4625
Y	**590.2933**	**708.4039**
I	703.3774	**545.3406**
E	832.4199	**432.2565**
K	960.5149	**303.2139**
R	1116.6160	**175.1190**

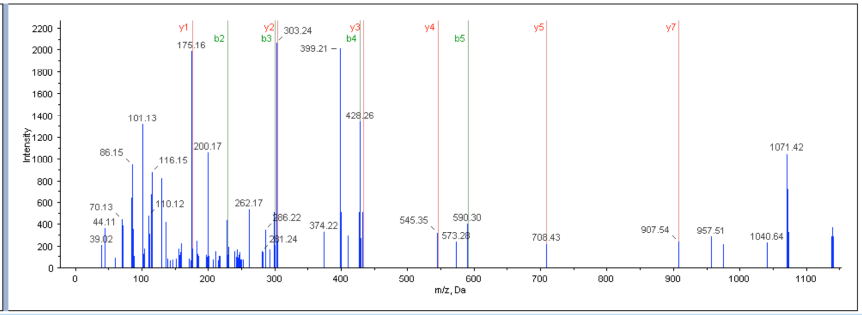

<table>
<tr>
<td>261</td>
<td>1507.728</td>
<td></td>
<td>
Fragmentation Evidence for Peptide

TANWVLGNHDQPR

<table>
<tr><th>Residue</th><th>b</th><th>y</th></tr>
<tr><td>T</td><td>102.0550</td><td>1507.7401</td></tr>
<tr><td>A</td><td>**173.0921**</td><td>1406.6924</td></tr>
<tr><td>N</td><td>**287.1350**</td><td>1335.6553</td></tr>
<tr><td>W</td><td>**473.2143**</td><td>1221.6123</td></tr>
<tr><td>V</td><td>572.2827</td><td>1035.5330</td></tr>
<tr><td>L</td><td>685.3668</td><td>936.4646</td></tr>
<tr><td>G</td><td>742.3883</td><td>823.3805</td></tr>
<tr><td>N</td><td>856.4312</td><td>766.3591</td></tr>
<tr><td>H</td><td>993.4901</td><td>**652.3161**</td></tr>
<tr><td>D</td><td>**1108.5170**</td><td>**515.2572**</td></tr>
<tr><td>Q</td><td>**1236.5756**</td><td>**400.2303**</td></tr>
<tr><td>P</td><td>1333.6284</td><td>**272.1717**</td></tr>
<tr><td>R</td><td>1489.7295</td><td>**175.1190**</td></tr>
</table>

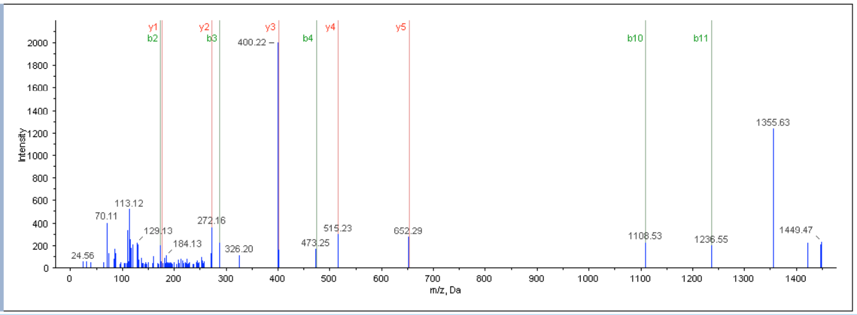

</td>
</tr>
<tr>
<td>262</td>
<td>1764.879</td>
<td></td>
<td>
Fragmentation Evidence for Peptide

NASTGNHIPIGVKEGDR

<table>
<tr><th>Residue</th><th>b</th><th>y</th></tr>
<tr><td>N</td><td>115.0502</td><td>1764.8987</td></tr>
<tr><td>A</td><td>**186.0873**</td><td>1650.8558</td></tr>
<tr><td>S</td><td>**273.1193**</td><td>1579.8187</td></tr>
<tr><td>T</td><td>374.1670</td><td>1492.7867</td></tr>
<tr><td>G</td><td>431.1885</td><td>1391.7390</td></tr>
<tr><td>N</td><td>545.2314</td><td>1334.7175</td></tr>
<tr><td>H</td><td>**682.2903**</td><td>1220.6746</td></tr>
<tr><td>I</td><td>**795.3744**</td><td>**1083.6157**</td></tr>
<tr><td>P</td><td>892.4272</td><td>**970.5316**</td></tr>
<tr><td>I</td><td>1005.5112</td><td>873.4789</td></tr>
<tr><td>G</td><td>1062.5327</td><td>**760.3948**</td></tr>
<tr><td>V</td><td>1161.6011</td><td>703.3733</td></tr>
<tr><td>K</td><td>1289.6961</td><td>**604.3049**</td></tr>
<tr><td>E</td><td>1418.7387</td><td>**476.2100**</td></tr>
<tr><td>G</td><td>1475.7601</td><td>**347.1674**</td></tr>
<tr><td>D</td><td>**1590.7871**</td><td>290.1459</td></tr>
<tr><td>R</td><td>1746.8882</td><td>**175.1190**</td></tr>
</table>

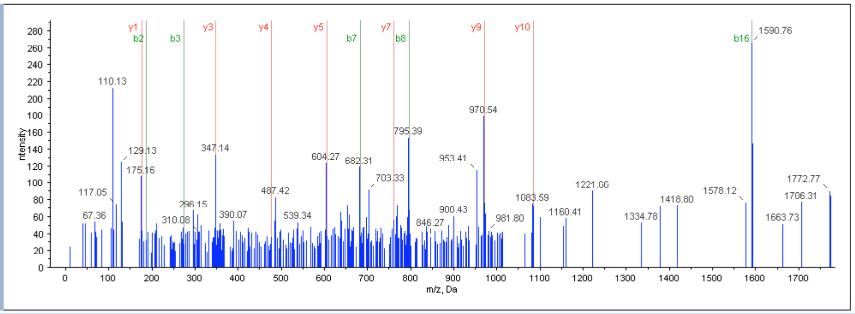

</td>
</tr>
<tr>
<td>263</td>
<td>1879.923</td>
<td></td>
<td>
Fragmentation Evidence for Peptide

MTQQQLLLFEEENTR

<table>
<tr><th>Residue</th><th>b</th><th>y</th></tr>
<tr><td>M</td><td>132.0478</td><td>1879.9218</td></tr>
<tr><td>T</td><td>233.0954</td><td>1748.8814</td></tr>
<tr><td>Q</td><td>361.1540</td><td>1647.8337</td></tr>
<tr><td>Q</td><td>489.2126</td><td>1519.7751</td></tr>
<tr><td>Q</td><td>617.2712</td><td>1391.7165</td></tr>
<tr><td>L</td><td>**730.3552**</td><td>1263.6579</td></tr>
<tr><td>L</td><td>**843.4393**</td><td>1150.5739</td></tr>
<tr><td>L</td><td>956.5234</td><td>1037.4898</td></tr>
<tr><td>F</td><td>1103.5918</td><td>924.4058</td></tr>
<tr><td>E</td><td>1232.6344</td><td>**777.3373**</td></tr>
<tr><td>E</td><td>1361.6770</td><td>648.2947</td></tr>
<tr><td>E</td><td>1490.7196</td><td>**519.2522**</td></tr>
<tr><td>N</td><td>1604.7625</td><td>**390.2096**</td></tr>
<tr><td>T</td><td>1705.8102</td><td>276.1666</td></tr>
<tr><td>R</td><td>1861.9113</td><td>**175.1190**</td></tr>
</table>

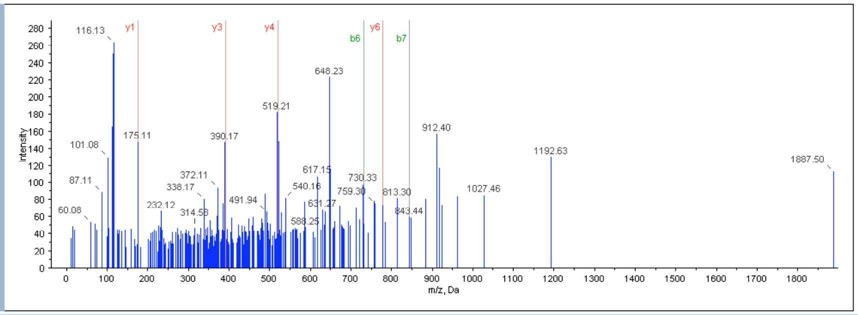

</td>
</tr>
</table>

264	1168.595		

Fragmentation Evidence for Peptide

GGPRPGGFGGGPR

Residue	b	y
G	58.0287	1168.5970
G	115.0502	1111.5756
P	212.1030	1054.5541
R	**368.2041**	957.5013
P	465.2568	**801.4002**
G	522.2783	704.3474
G	579.2998	**647.3260**
F	726.3682	590.3045
G	783.3896	443.2361
G	**840.4111**	386.2146
G	**897.4326**	329.1932
P	**994.4853**	**272.1717**
R	1150.5865	**175.1190**

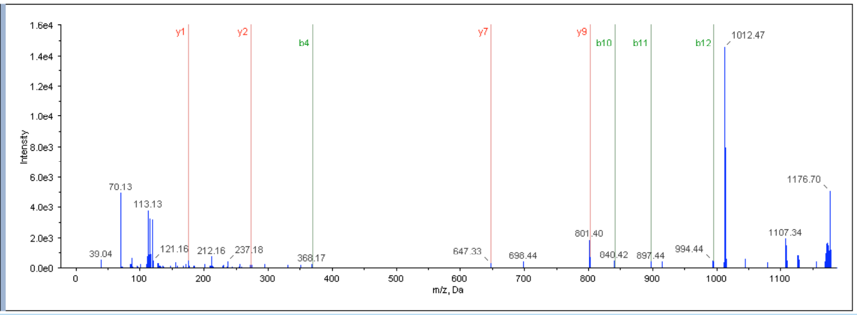

265	1475.681		

Fragmentation Evidence for Peptide

TSSWNFKDYAEK

Residue	b	y
T	**102.0550**	1475.6801
S	**189.0870**	1374.6325
S	276.1190	1287.6004
W	**462.1983**	1200.5684
N	576.2413	**1014.4891**
F	723.3097	**900.4462**
K	851.4046	**753.3777**
D	**966.4316**	**625.2828**
Y	1129.4949	510.2558
A	1200.5320	347.1925
E	1329.5746	**276.1554**
K	1457.6696	147.1128

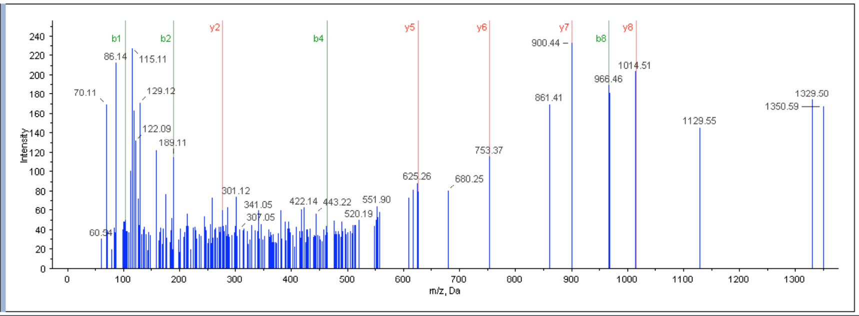

266	1595.683		

Fragmentation Evidence for Peptide

IGQPC[CAM]EDIYEESR

Residue	b	y
I	114.0913	1595.7006
G	171.1128	1482.6165
Q	**299.1714**	1425.5951
P	396.2241	1297.5365
C[CAM]	556.2548	1200.4837
E	685.2974	1040.4531
D	800.3243	**911.4105**
I	913.4084	**796.3836**
Y	1076.4717	683.2995
E	1205.5143	520.2362
E	1334.5569	**391.1936**
S	1421.5889	**262.1510**
R	1577.6900	**175.1190**

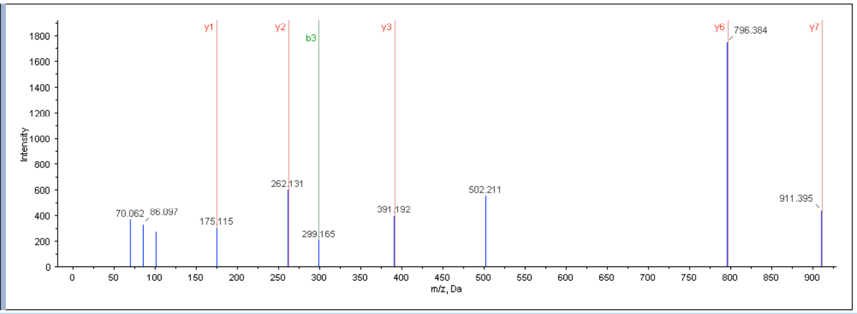

267	2890.353	Fragmentation Evidence for Peptide (see below)
268	1390.736	Fragmentation Evidence for Peptide (see below)

267 — Fragmentation Evidence for Peptide

PGQ QYW[Oxi]DN[Dea]PEDDEQKPALQTTVTTSPAR

Residue	b	y
Q	112.0393	2890.3014
Y	275.1026	2779.2693
W[Oxi]	477.1769	2616.2060
D	592.2038	2414.1318
N[Dea]	707.2307	**2299.1048**
P	804.2835	**2184.0779**
E	933.3261	2087.0251
D	1048.3530	**1957.9825**
D	1163.3800	**1842.9556**
E	1292.4226	1727.9286
Q	**1420.4812**	1598.8860
K	**1548.5761**	1470.8275
P	1645.6289	1342.7325
A	1716.6660	1245.6797
L	1829.7501	1174.6426
Q	1957.8086	1061.5586
T	2058.8563	933.5000
T	2159.9040	832.4523
V	2258.9724	731.4046
T	2360.0201	**632.3362**
T	2461.0678	531.2885
S	2548.0998	430.2409
P	2645.1526	343.2088
A	2716.1897	246.1561
R	2872.2908	175.1190

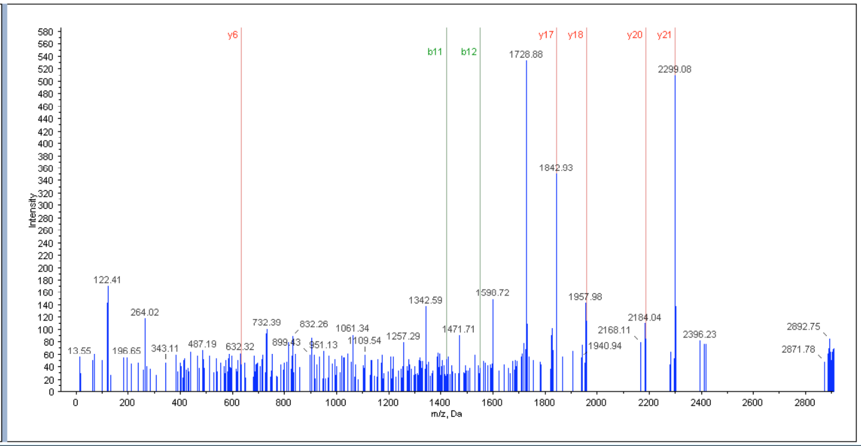

268 — Fragmentation Evidence for Peptide

TFIMVKPDGVQR

Residue	b	y
T	102.0550	1390.7511
F	**249.1234**	1289.7035
I	**362.2074**	1142.6350
M	**493.2479**	1029.5510
V	592.3163	**898.5105**
K	720.4113	**799.4421**
P	817.4641	**671.3471**
D	932.4910	574.2944
G	989.5125	**459.2674**
V	1088.5809	402.2459
Q	1216.6395	**303.1775**
R	1372.7406	**175.1190**

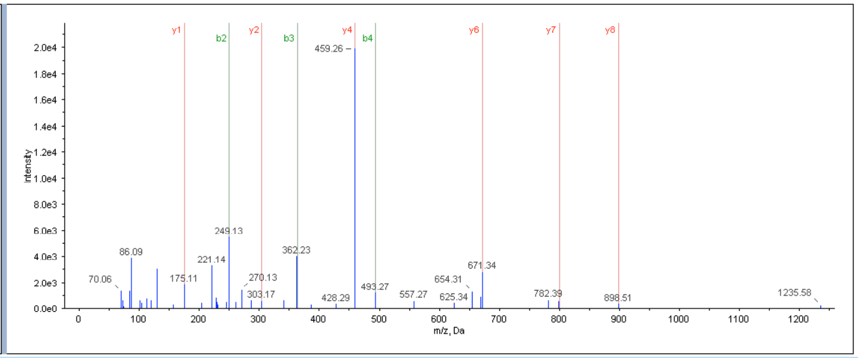

269	2179.916	

Fragmentation Evidence for Peptide

SSFDSHGESDHNLDVPHGSR

Residue	b	y
S	88.0393	2179.9388
S	175.0713	2092.9067
F	322.1397	2005.8747
D	437.1667	1858.8063
S	**524.1987**	**1743.7793**
H	661.2576	1656.7473
G	718.2791	1519.6884
E	847.3217	1462.6669
S	934.3537	1333.6244
D	1049.3807	1246.5923
H	1186.4396	1131.5654
N	1300.4825	**994.5065**
L	1413.5666	**880.4635**
D	**1528.5935**	767.3795
V	**1627.6619**	652.3525
P	1724.7147	**553.2841**
H	1861.7736	456.2314
G	1918.7951	**319.1724**
S	2005.8271	262.1510
R	2161.9282	175.1190

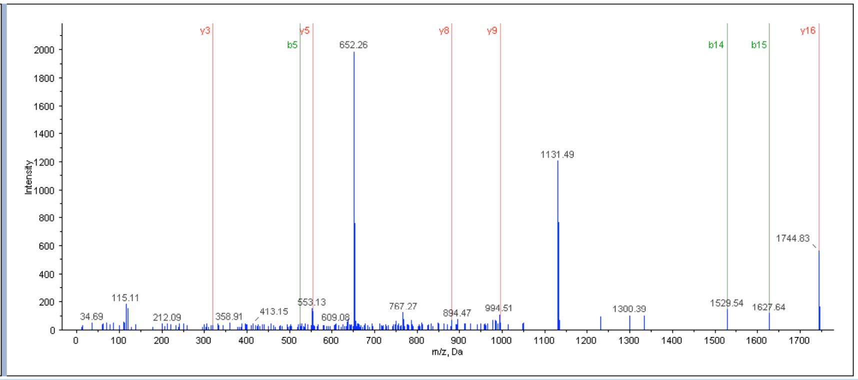

270	2007.065	

Fragmentation Evidence for Peptide

YGQKEQQLYPKELVQR

Residue	b	y
Y	164.0706	2007.0658
G	221.0921	1844.0025
Q	349.1506	1786.9810
K	477.2456	1658.9224
E	606.2882	1530.8275
Q	734.3468	1401.7849
Q	862.4054	1273.7263
L	975.4894	**1145.6677**
Y	1138.5527	1032.5837
P	1235.6055	869.5203
K	1363.7005	**772.4676**
E	1492.7431	644.3726
L	1605.8271	515.3300
V	1704.8955	402.2459
Q	1832.9541	303.1775
R	1989.0552	**175.1190**

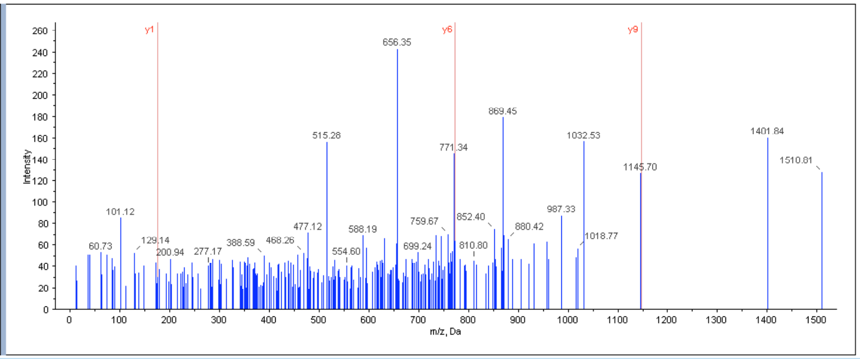

271	1268.533	

Fragmentation Evidence for Peptide

TTWNC[CAM]GEMNR

Residue	b	y
T	**102.0550**	1268.5147
T	**203.1026**	1167.4670
W	389.1819	1066.4193
N	503.2249	880.3400
C[CAM]	**663.2555**	**766.2971**
G	720.2770	**606.2664**
E	**849.3196**	**549.2450**
M	980.3601	**420.2024**
N	1094.4030	**289.1619**
R	1250.5041	**175.1190**

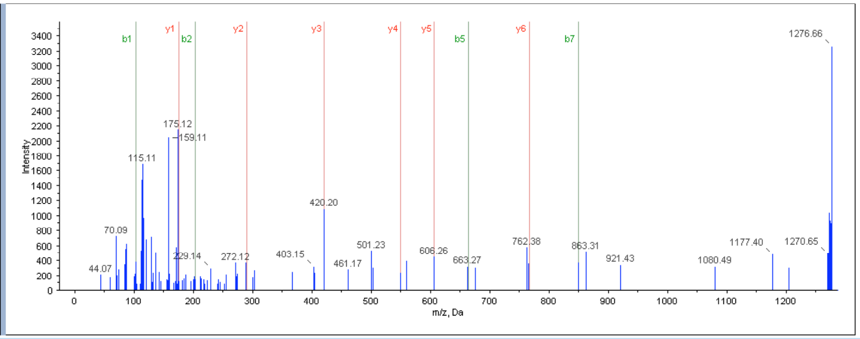

272	2006.04		

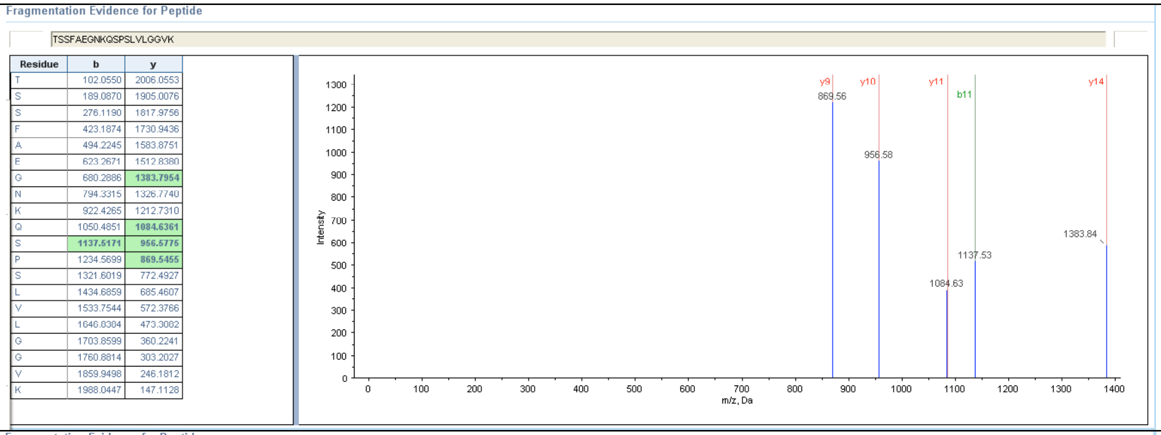

Fragmentation Evidence for Peptide

TSSFAEGNKQSPSLVLGGVK

Residue	b	y
T	102.0550	2006.0553
S	189.0870	1905.0076
S	276.1190	1817.9756
F	423.1874	1730.9436
A	494.2245	1583.8751
E	623.2671	1512.8380
G	680.2886	**1383.7954**
N	794.3315	1326.7740
K	922.4265	1212.7310
Q	1050.4851	**1084.6361**
S	**1137.5171**	**956.5775**
P	1234.5699	**869.5455**
S	1321.6019	772.4927
L	1434.6859	685.4607
V	1533.7544	572.3766
L	1646.8384	473.3082
G	1703.8599	360.2241
G	1760.8814	303.2027
V	1859.9498	246.1812
K	1988.0447	147.1128

273	2314.156		

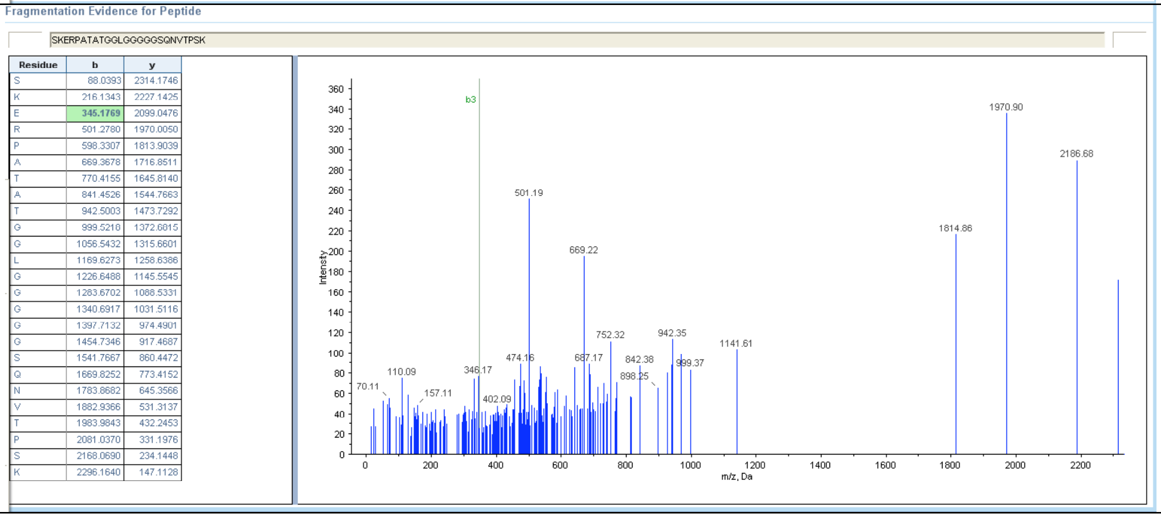

Fragmentation Evidence for Peptide

SKERPATATGGLGGGGGSQNVTPSK

Residue	b	y
S	88.0393	2314.1746
K	216.1343	2227.1425
E	**345.1769**	2099.0476
R	501.2780	1970.0050
P	598.3307	1813.9039
A	669.3678	1716.8511
T	770.4155	1645.8140
A	841.4526	1544.7663
T	942.5003	1473.7292
G	999.5218	1372.6815
G	1056.5432	1315.6601
L	1169.6273	1258.6386
G	1226.6488	1145.5545
G	1283.6702	1088.5331
G	1340.6917	1031.5116
G	1397.7132	974.4901
G	1454.7346	917.4687
S	1541.7667	860.4472
Q	1669.8252	773.4152
N	1783.8682	645.3566
V	1882.9366	531.3137
T	1983.9843	432.2453
P	2081.0370	331.1976
S	2168.0690	234.1448
K	2296.1640	147.1128

<table>
<tr>
<td>274</td>
<td>1286.675</td>
<td></td>
<td>

Fragmentation Evidence for Peptide

RVDIDQC[CAM]PQR

Residue	b	y
R	157.1084	1286.6270
V	256.1768	1130.5259
D	371.2037	1031.4575
I	484.2878	916.4305
D	599.3148	803.3465
Q	727.3733	688.3195
C[CAM]	887.4040	560.2609
P	984.4567	400.2303
Q	1112.5153	303.1775
R	1268.6164	175.1190

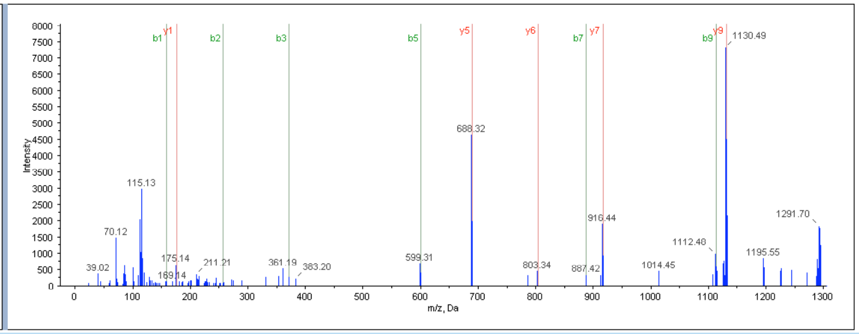

</td>
</tr>
<tr>
<td>275</td>
<td>1119.565</td>
<td></td>
<td>

Fragmentation Evidence for Peptide

NFQQIQEGR

Residue	b	y
N	115.0502	1119.5541
F	262.1186	1005.5112
Q	390.1772	858.4428
Q	518.2358	730.3842
I	631.3198	602.3257
Q	759.3784	489.2416
E	888.4210	361.1830
G	945.4425	232.1404
R	1101.5436	175.1190

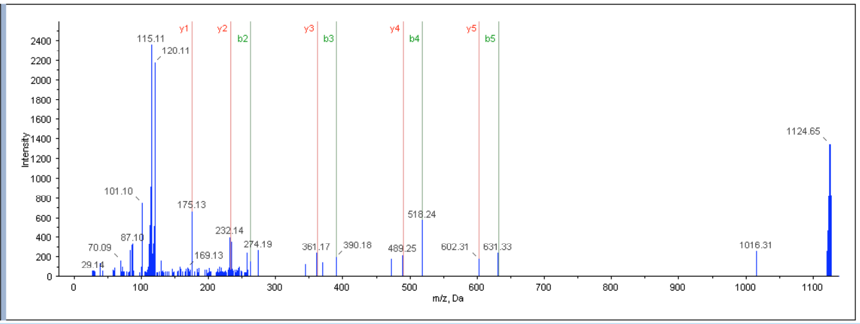

</td>
</tr>
<tr>
<td>276</td>
<td>867.4809</td>
<td></td>
<td>

Fragmentation Evidence for Peptide

AVFFVER

Residue	b	y
A	72.0444	867.4723
V	171.1128	796.4352
F	318.1812	697.3668
F	465.2496	550.2984
V	564.3180	403.2300
E	693.3606	304.1615
R	849.4618	175.1190

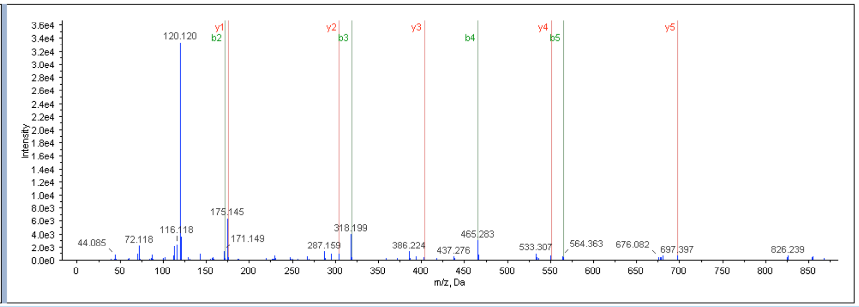

</td>
</tr>
</table>

<table>
<tr><td>277</td><td>1197.616</td><td></td><td>
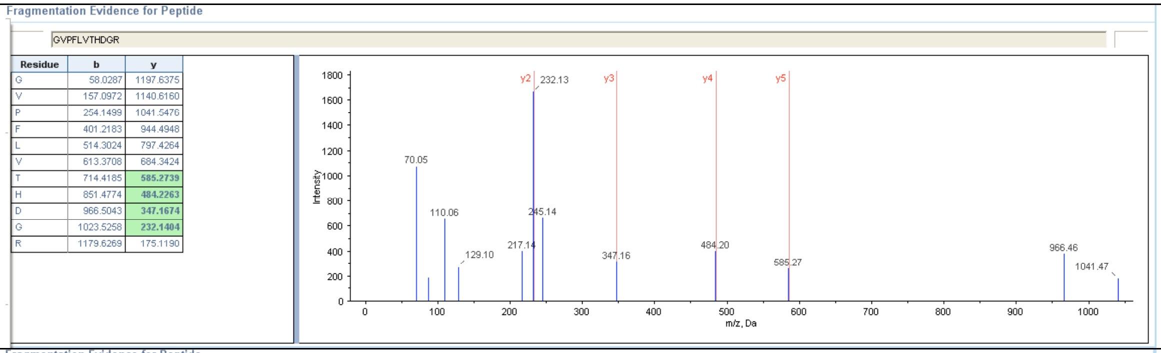

<table>
<tr><th>Residue</th><th>b</th><th>y</th></tr>
<tr><td>G</td><td>58.0287</td><td>1197.6375</td></tr>
<tr><td>V</td><td>157.0972</td><td>1140.6160</td></tr>
<tr><td>P</td><td>254.1499</td><td>1041.5476</td></tr>
<tr><td>F</td><td>401.2183</td><td>944.4948</td></tr>
<tr><td>L</td><td>514.3024</td><td>797.4264</td></tr>
<tr><td>V</td><td>613.3708</td><td>684.3424</td></tr>
<tr><td>T</td><td>714.4185</td><td>585.2739</td></tr>
<tr><td>H</td><td>851.4774</td><td>484.2263</td></tr>
<tr><td>D</td><td>966.5043</td><td>347.1674</td></tr>
<tr><td>G</td><td>1023.5258</td><td>232.1404</td></tr>
<tr><td>R</td><td>1179.6269</td><td>175.1190</td></tr>
</table>
</td></tr>
<tr><td>278</td><td>1316.675</td><td></td><td>
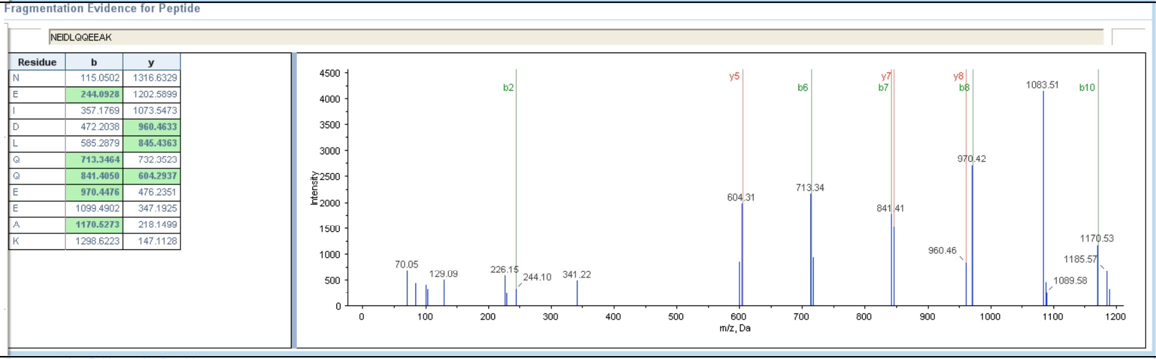

<table>
<tr><th>Residue</th><th>b</th><th>y</th></tr>
<tr><td>N</td><td>115.0502</td><td>1316.6329</td></tr>
<tr><td>E</td><td>244.0928</td><td>1202.5899</td></tr>
<tr><td>I</td><td>357.1769</td><td>1073.5473</td></tr>
<tr><td>D</td><td>472.2038</td><td>960.4633</td></tr>
<tr><td>L</td><td>585.2879</td><td>845.4363</td></tr>
<tr><td>Q</td><td>713.3464</td><td>732.3523</td></tr>
<tr><td>Q</td><td>841.4050</td><td>604.2937</td></tr>
<tr><td>E</td><td>970.4476</td><td>476.2351</td></tr>
<tr><td>E</td><td>1099.4902</td><td>347.1925</td></tr>
<tr><td>A</td><td>1170.5273</td><td>218.1499</td></tr>
<tr><td>K</td><td>1298.6223</td><td>147.1128</td></tr>
</table>
</td></tr>
<tr><td>279</td><td>1352.693</td><td></td><td>
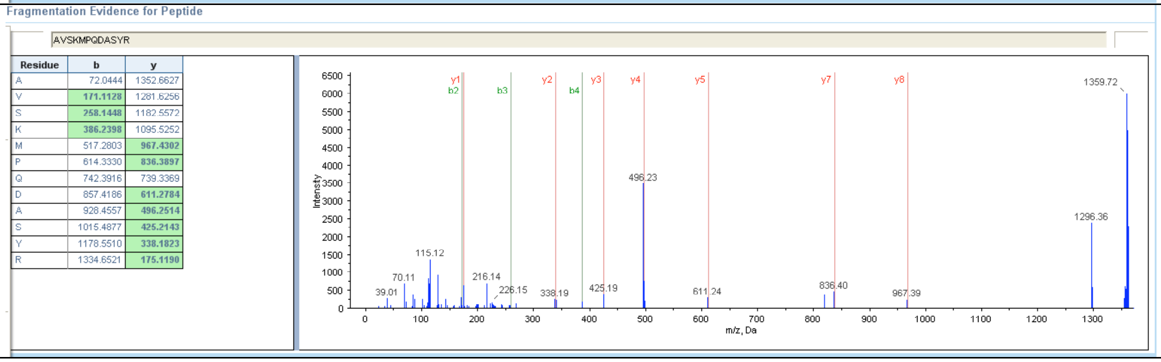

<table>
<tr><th>Residue</th><th>b</th><th>y</th></tr>
<tr><td>A</td><td>72.0444</td><td>1352.6627</td></tr>
<tr><td>V</td><td>171.1128</td><td>1281.6256</td></tr>
<tr><td>S</td><td>258.1448</td><td>1182.5572</td></tr>
<tr><td>K</td><td>386.2398</td><td>1095.5252</td></tr>
<tr><td>M</td><td>517.2803</td><td>967.4302</td></tr>
<tr><td>P</td><td>614.3330</td><td>836.3897</td></tr>
<tr><td>Q</td><td>742.3916</td><td>739.3369</td></tr>
<tr><td>D</td><td>857.4186</td><td>611.2784</td></tr>
<tr><td>A</td><td>928.4557</td><td>496.2514</td></tr>
<tr><td>S</td><td>1015.4877</td><td>425.2143</td></tr>
<tr><td>Y</td><td>1178.5510</td><td>338.1823</td></tr>
<tr><td>R</td><td>1334.6521</td><td>175.1190</td></tr>
</table>
</td></tr>
</table>

<table>
<tr>
<td>280</td>
<td>1333.613</td>
<td>

Fragmentation Evidence for Peptide

LSSN[Dea]Q[Dea]ATPSDGVR

Residue	b	y
L	114.0913	1333.6230
S	201.1234	1220.5390
S	288.1554	1133.5069
N[Dea]	403.1823	1046.4749
Q[Dea]	532.2249	931.4480
A	603.2620	**802.4054**
T	704.3097	731.3682
P	801.3625	630.3206
S	888.3945	533.2678
D	1003.4215	446.2358
G	1060.4429	**331.2088**
V	1159.5113	274.1874
R	1315.6124	**175.1190**

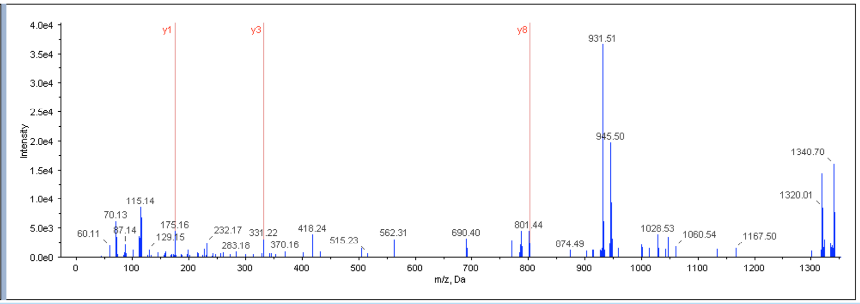

</td>
</tr>
<tr>
<td>281</td>
<td>1669.866</td>
<td>

Fragmentation Evidence for Peptide

VLM[Oxi]AC[CAM]VGIGYTN[Dea]INK

Residue	b	y
V	100.0757	1669.8288
L	213.1598	1570.7604
M[Oxi]	360.1952	**1457.6763**
A	431.2323	1310.6409
C[CAM]	591.2629	1239.6038
V	690.3313	1079.5732
G	747.3528	**980.5047**
I	860.4369	923.4833
G	917.4583	810.3992
Y	1080.5216	753.3777
T	**1181.5693**	590.3144
N[Dea]	1296.5963	489.2667
I	1409.6803	374.2398
N	**1523.7233**	261.1557
K	1651.8182	147.1128

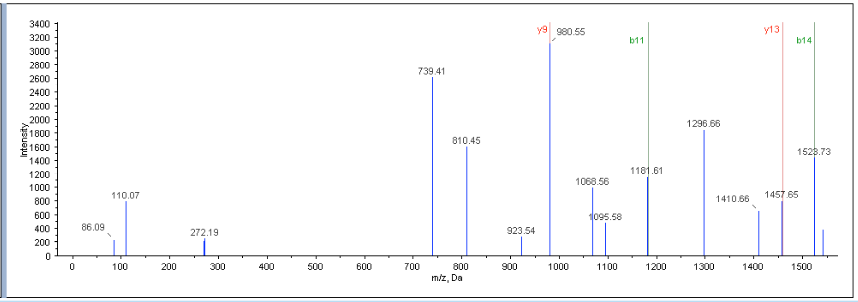

</td>
</tr>
</table>
