# Supplementary figures and images for: Towards a membrane proteome in Drosophila: a method for the isolation of plasma membrane
Source: BMC Genomics. 2010 May 12;11:302. doi: 10.1186/1471-2164-11-302 (PMC2876126; doi:10.1186/1471-2164-11-302)

# Hydropathy analysis of predicted integral membrane proteins

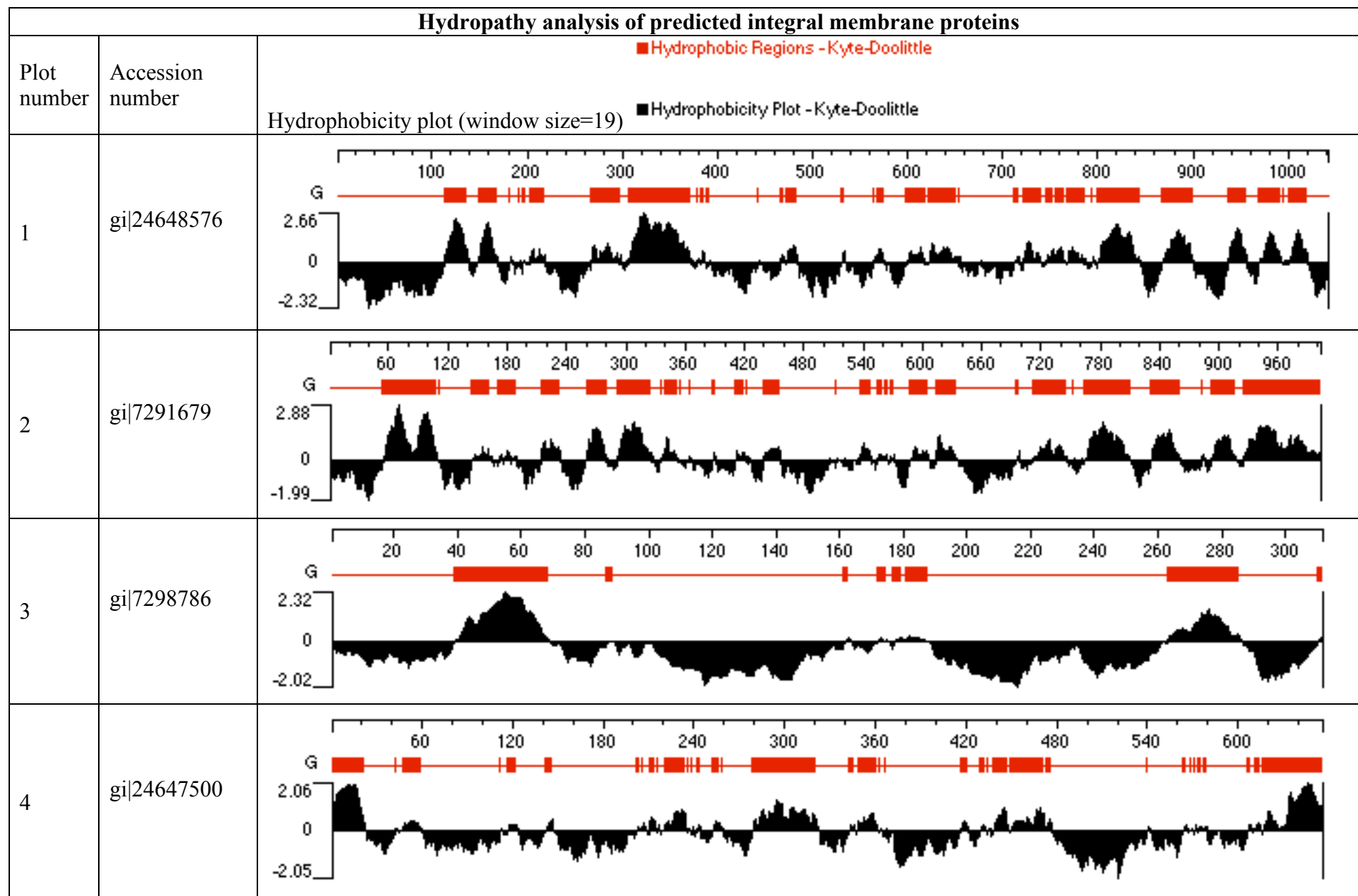

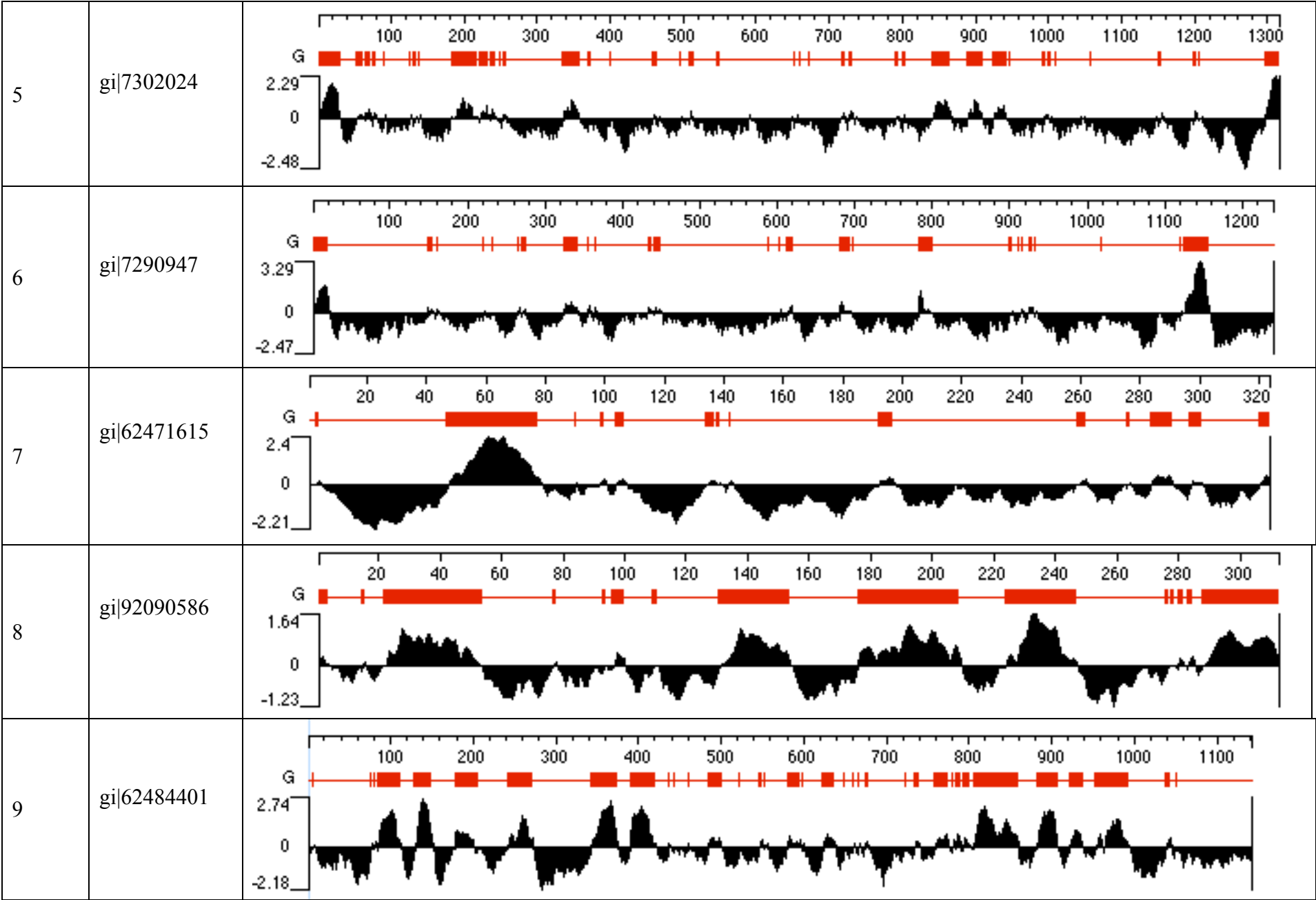

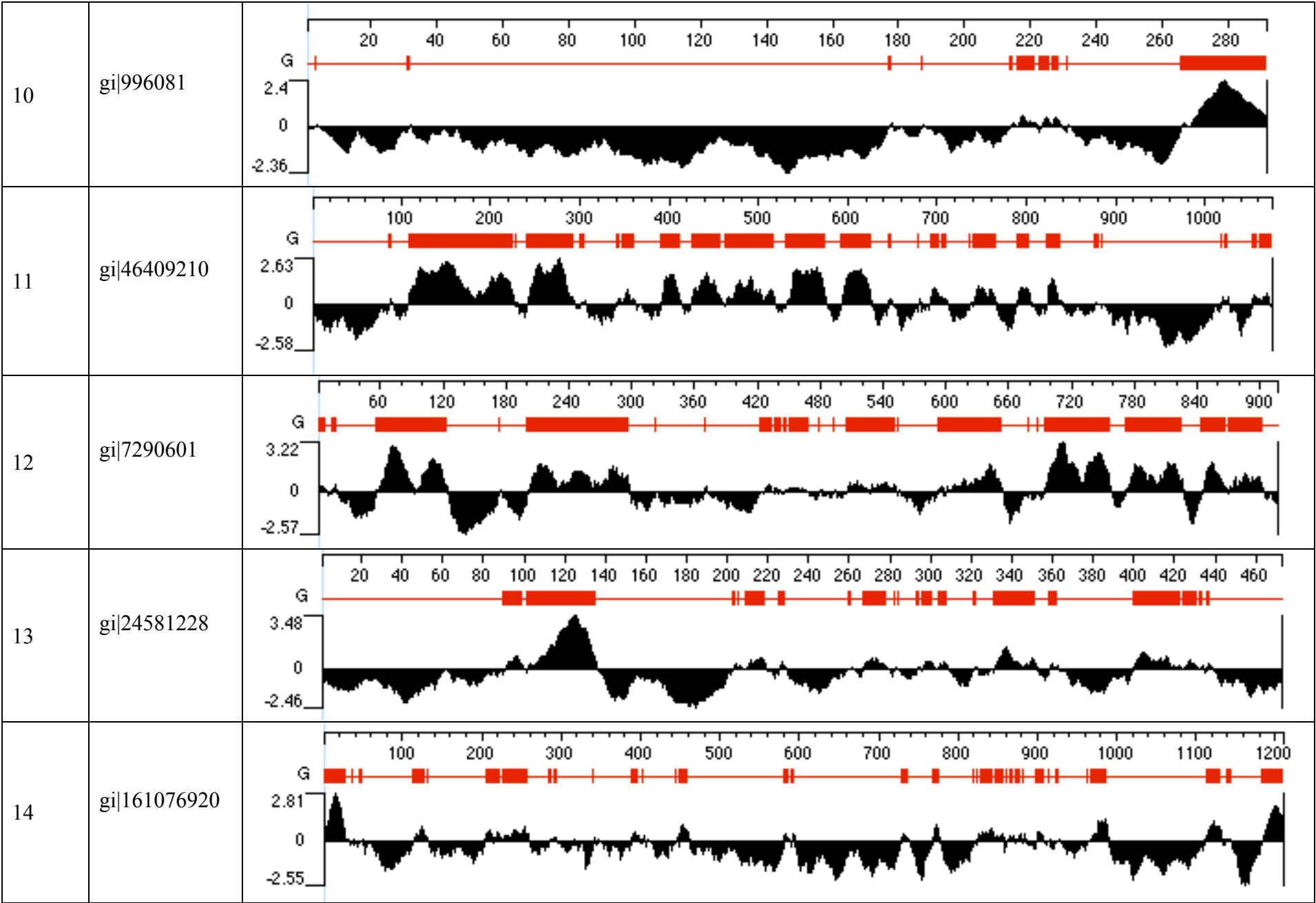

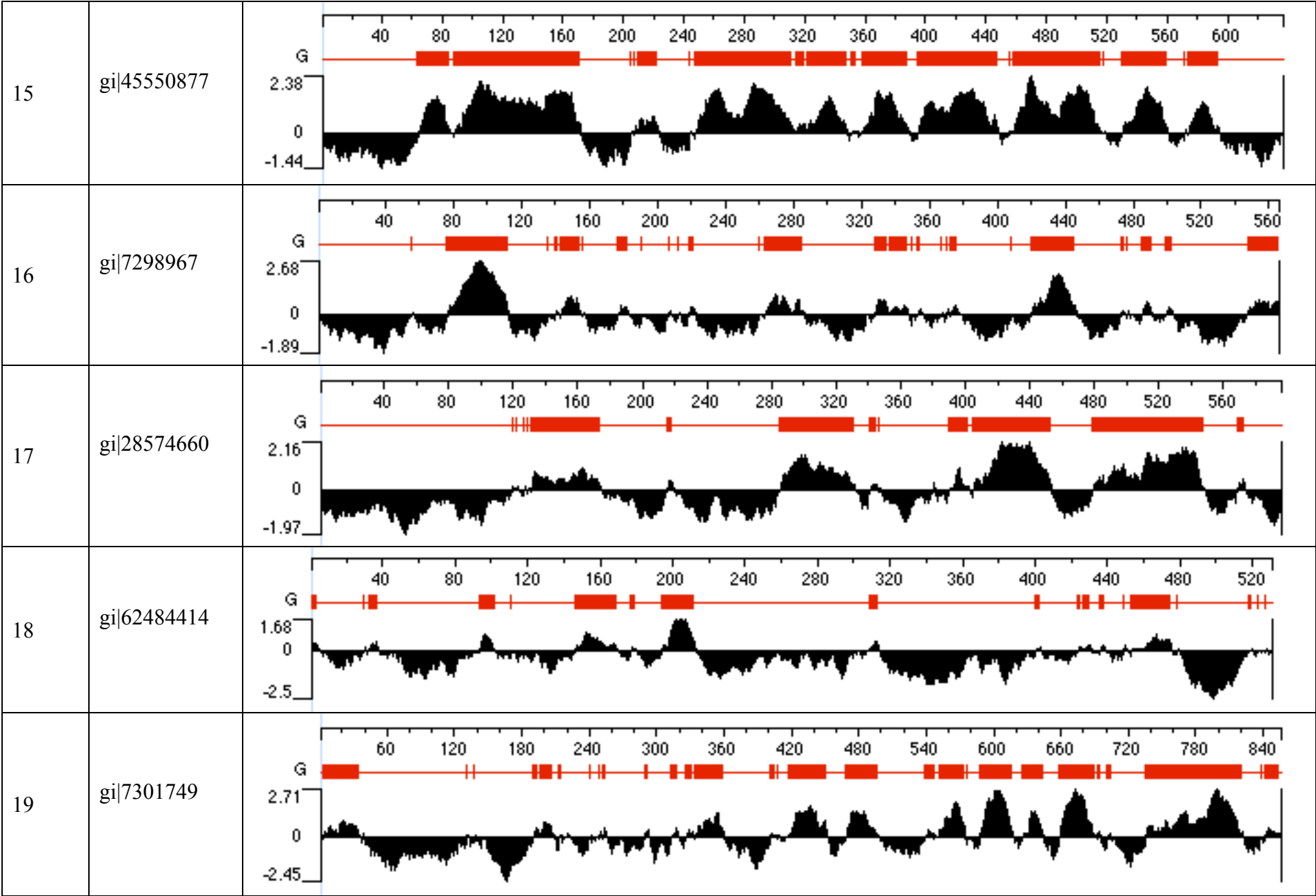

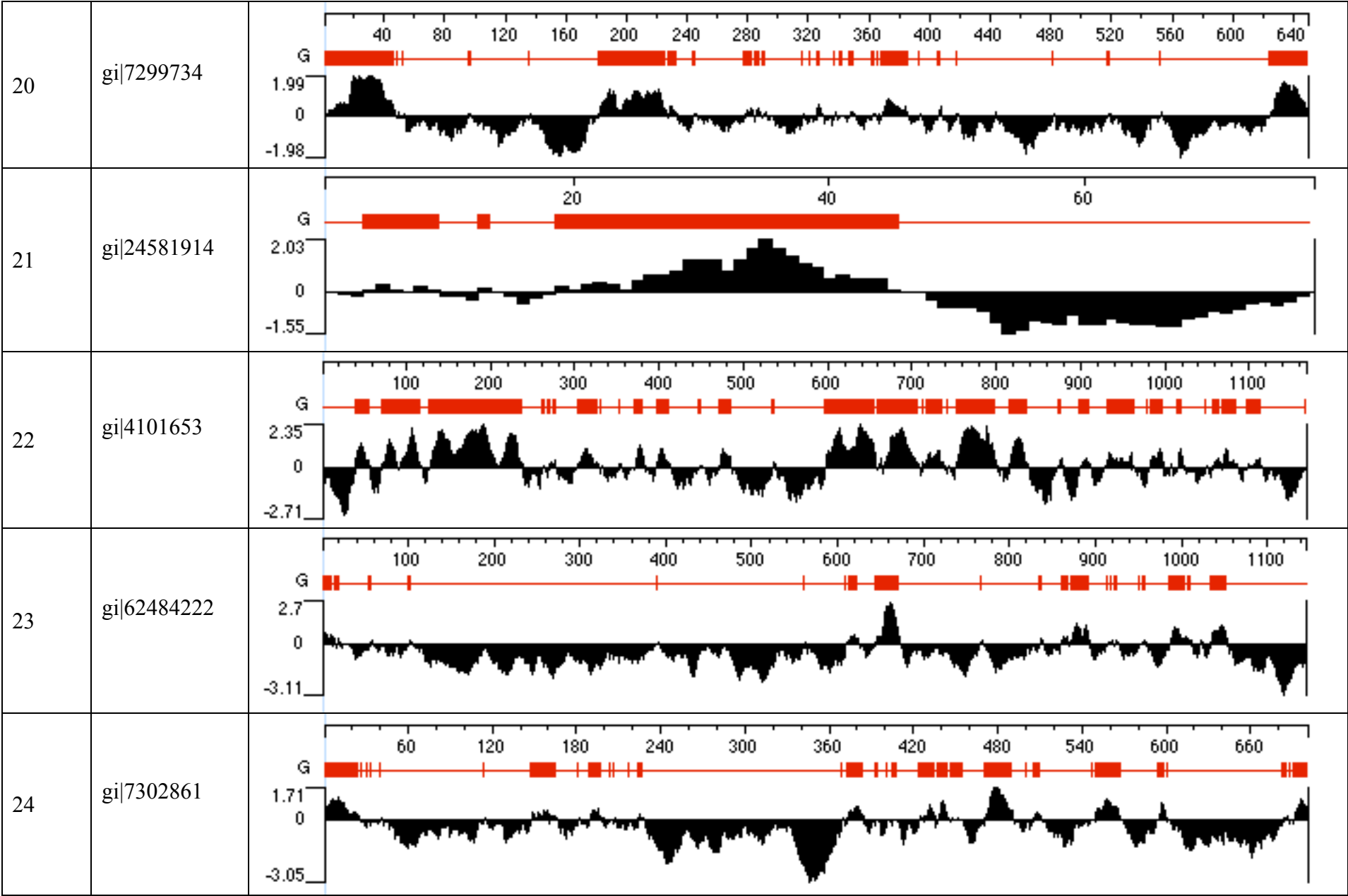

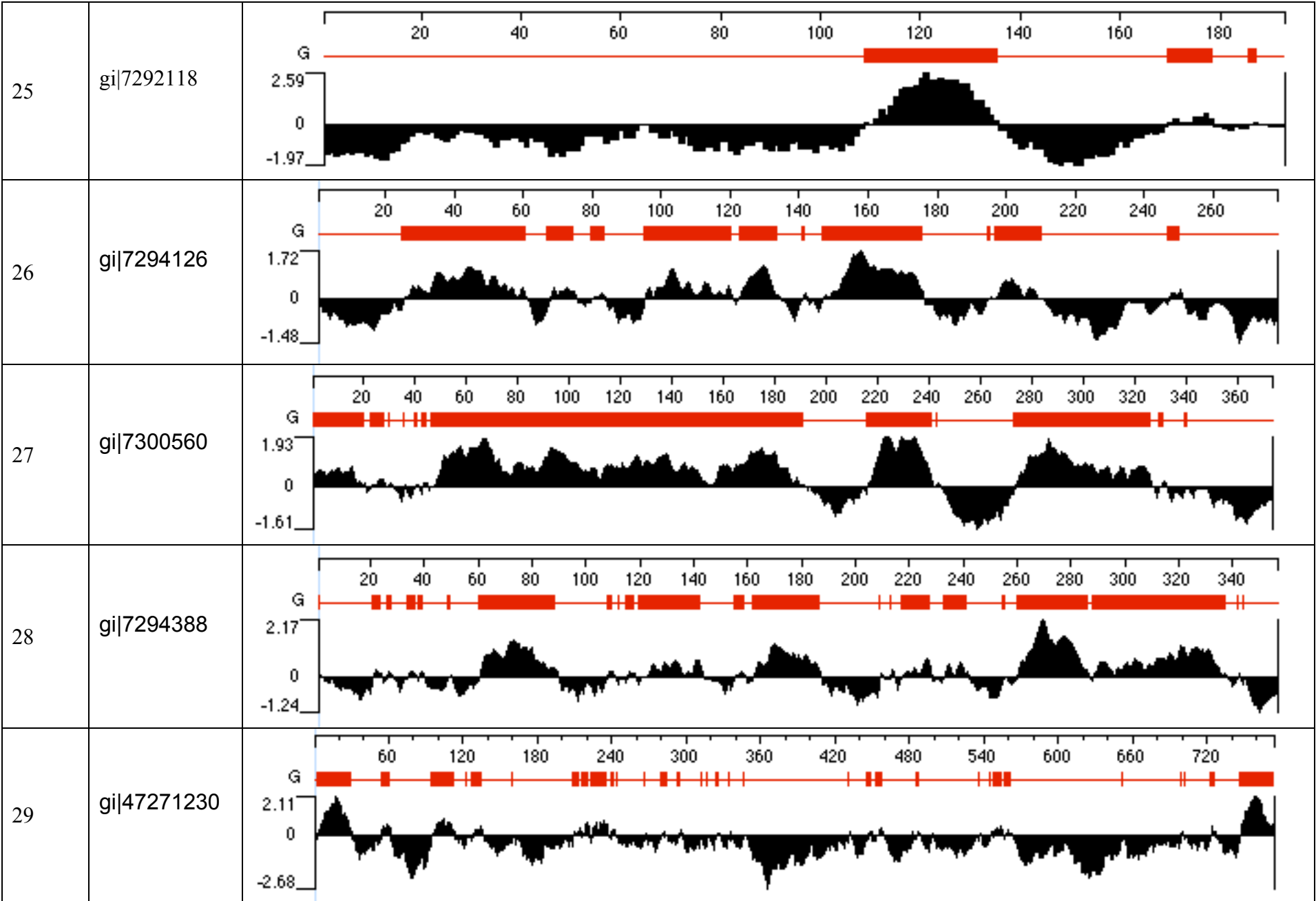

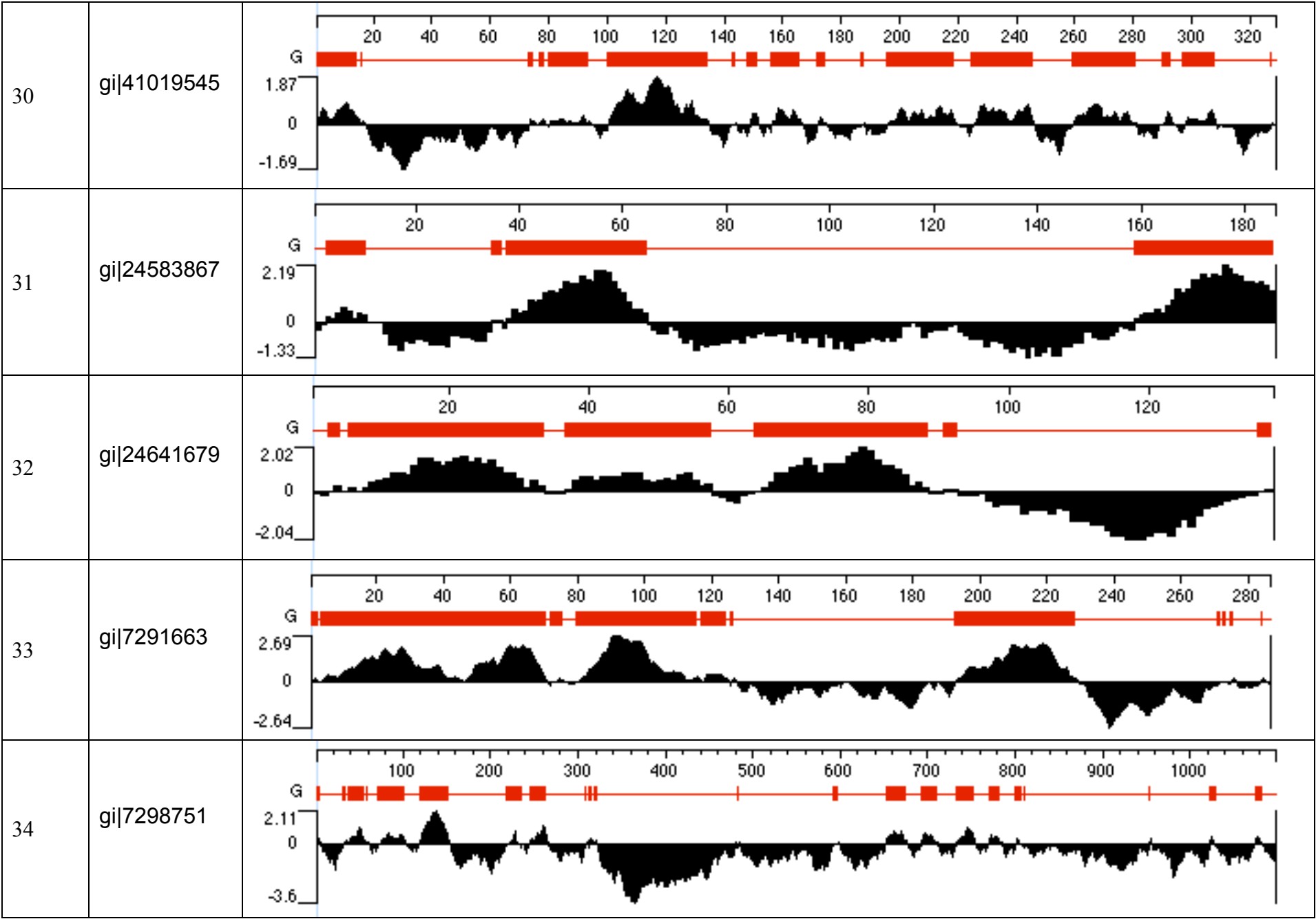

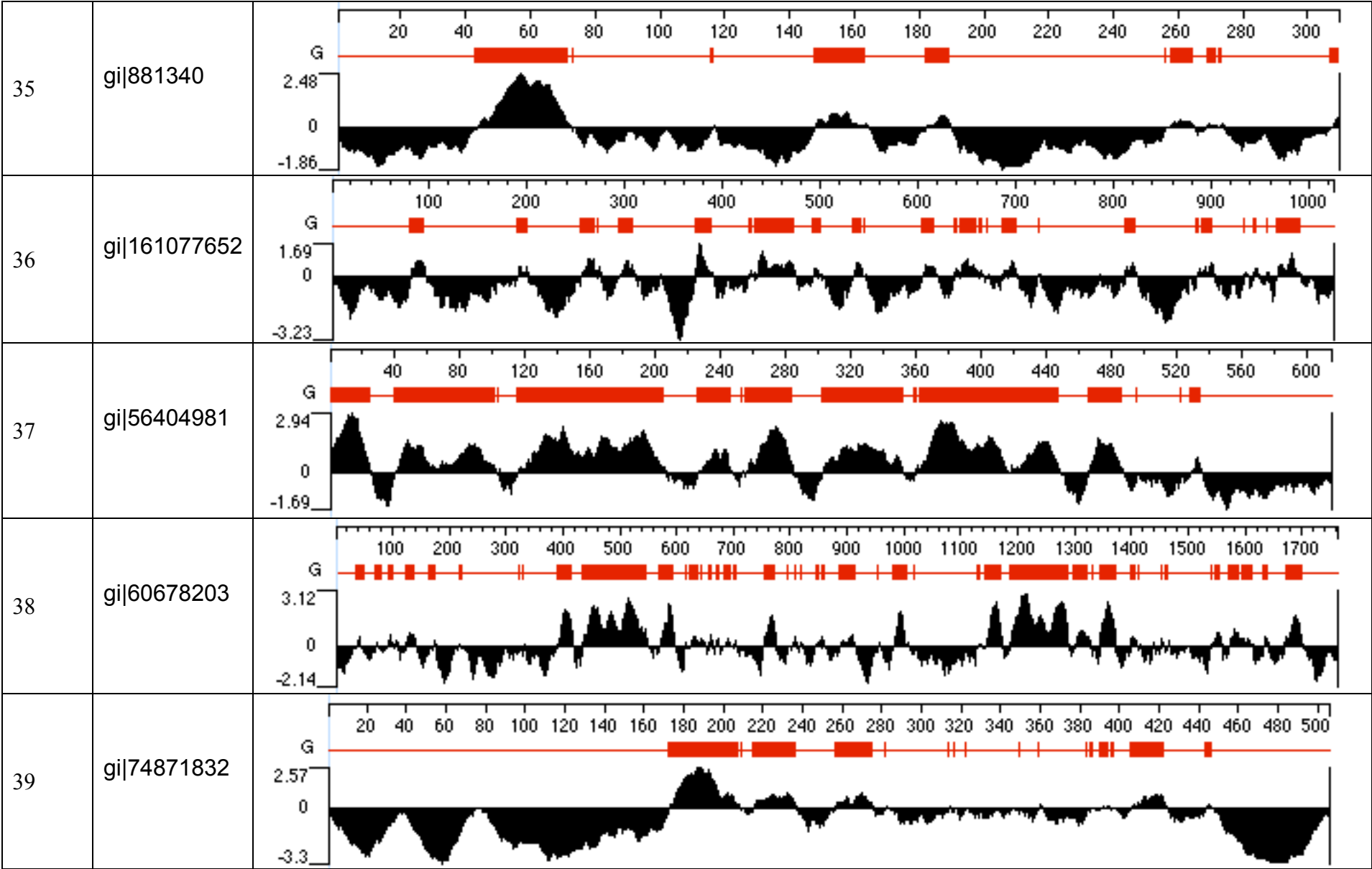

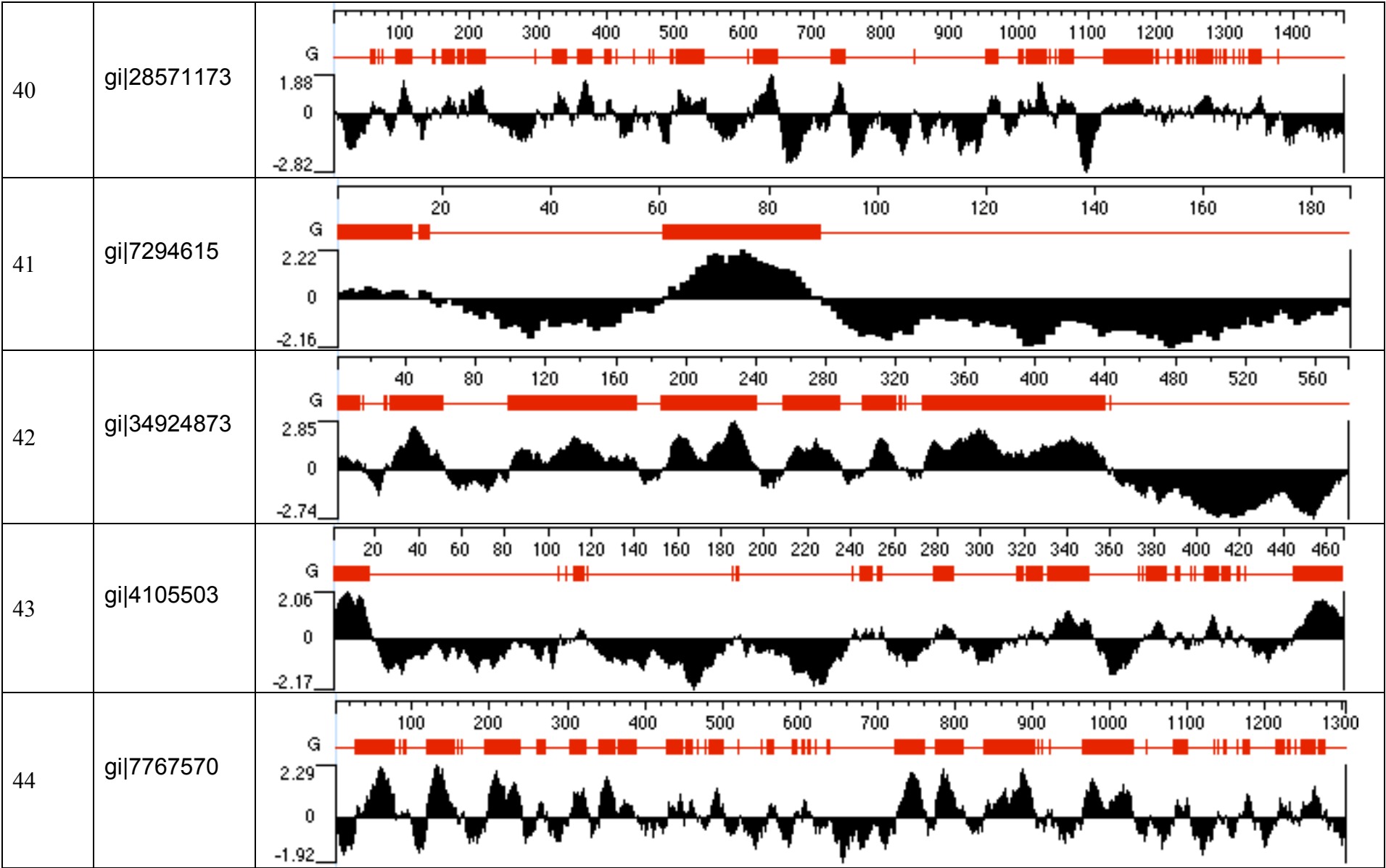

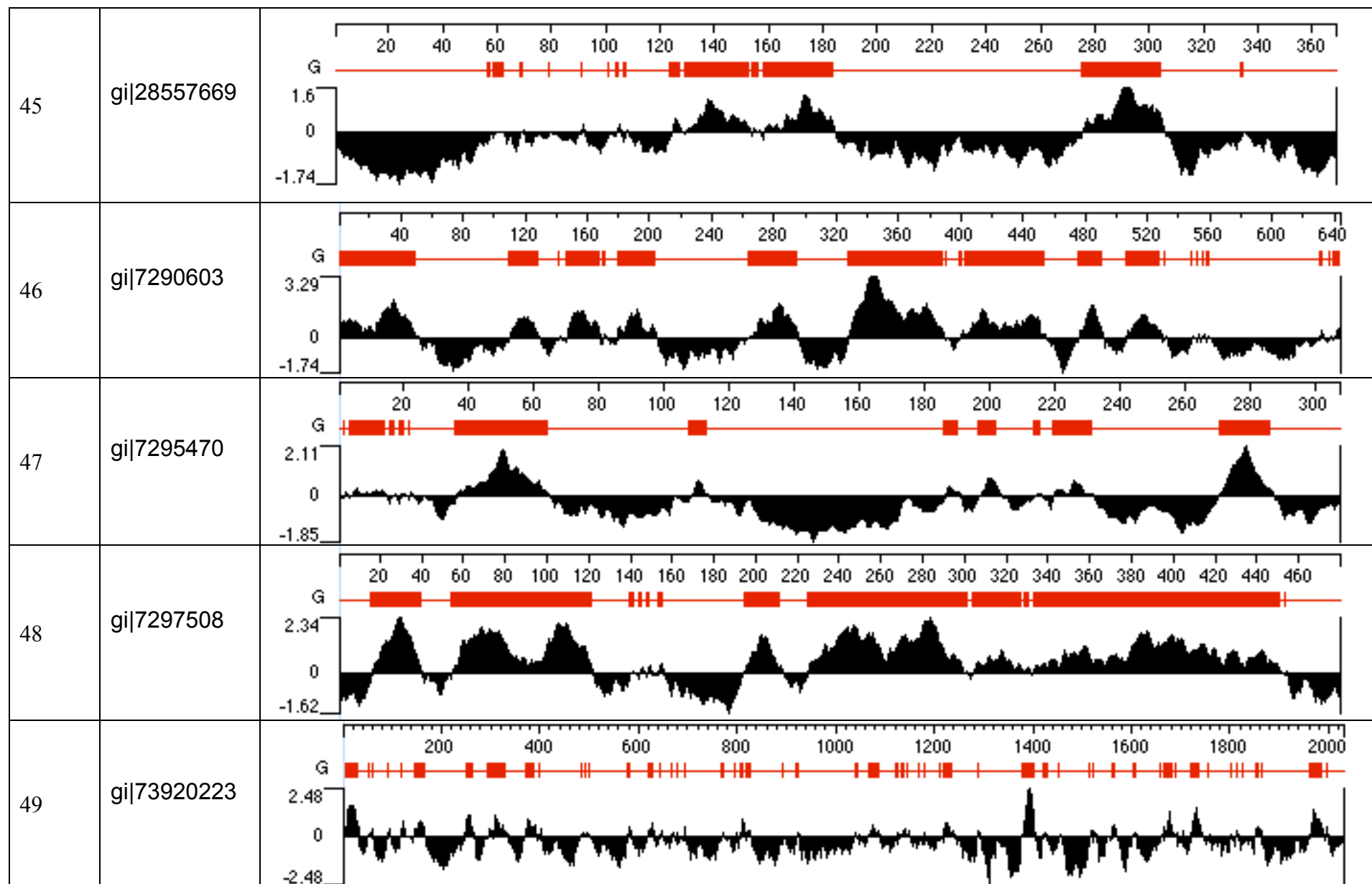

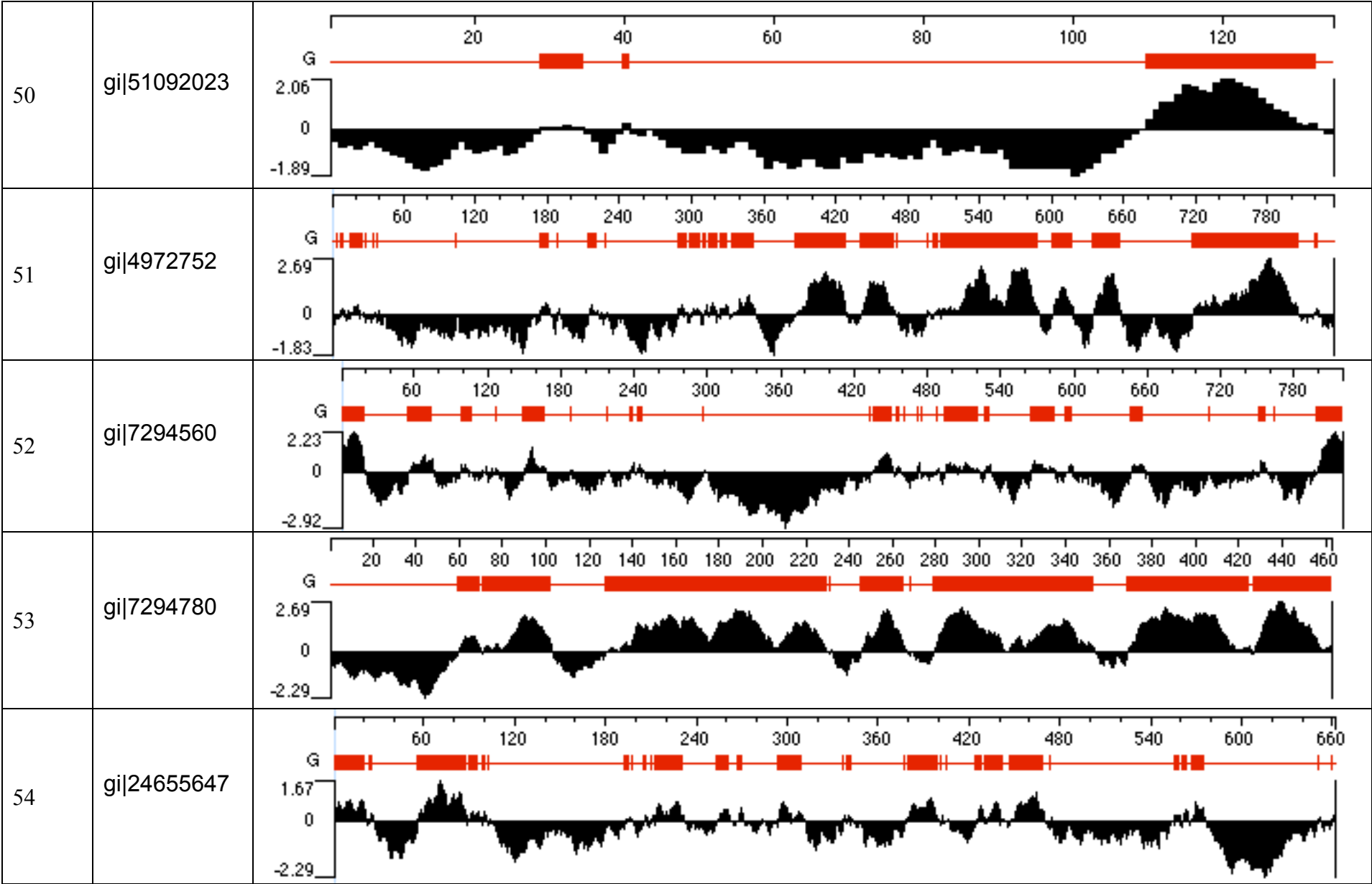

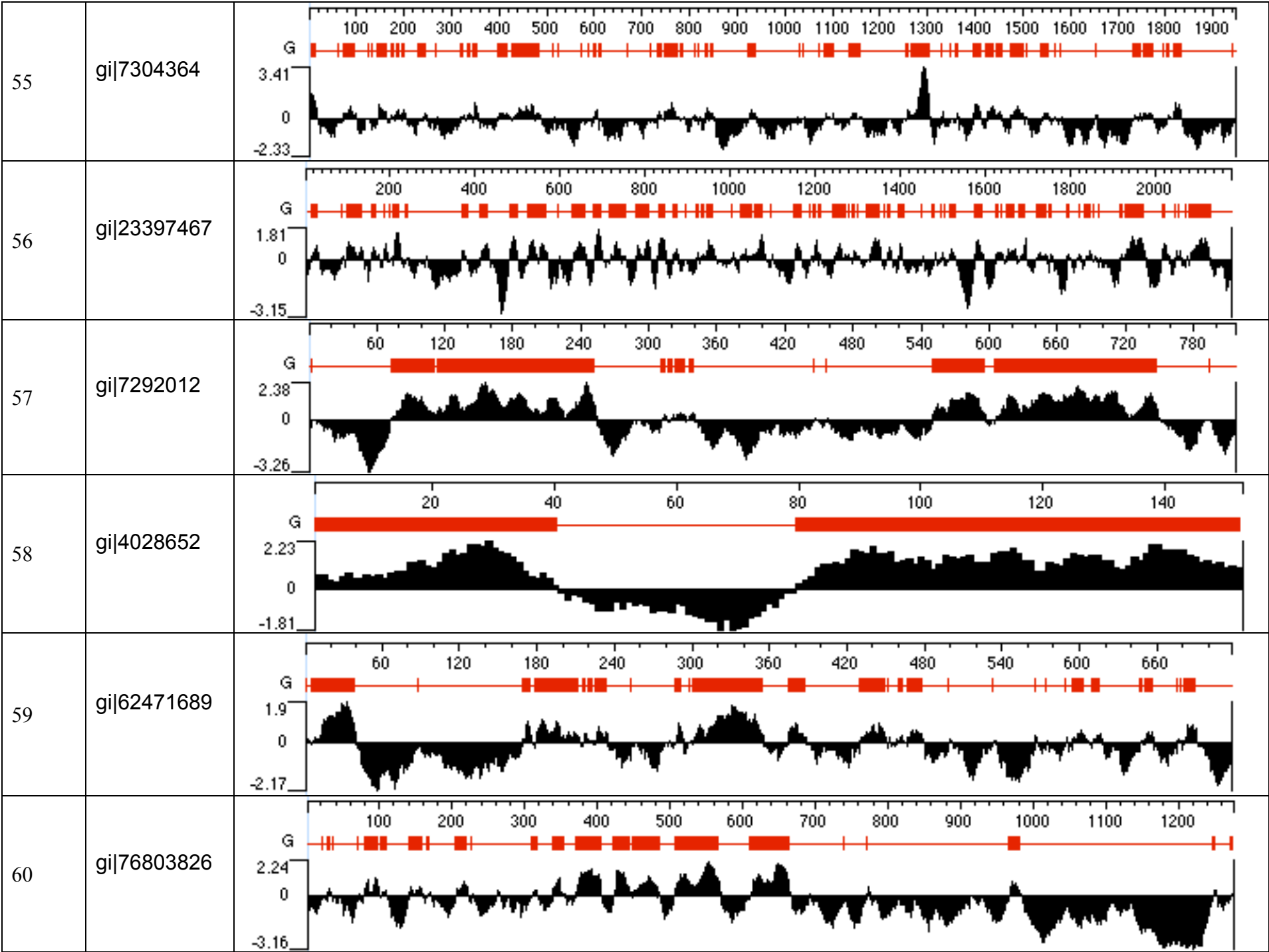

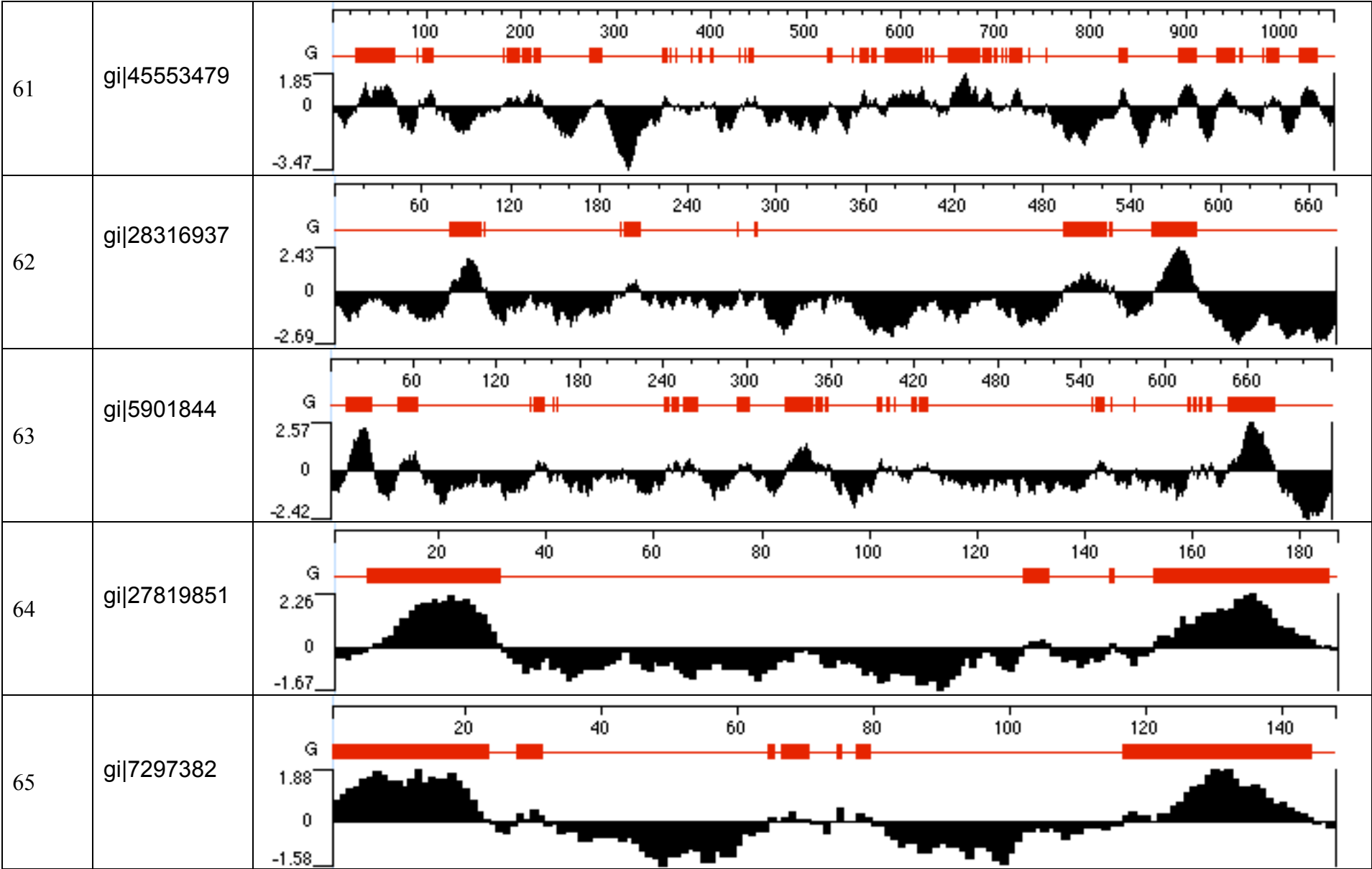

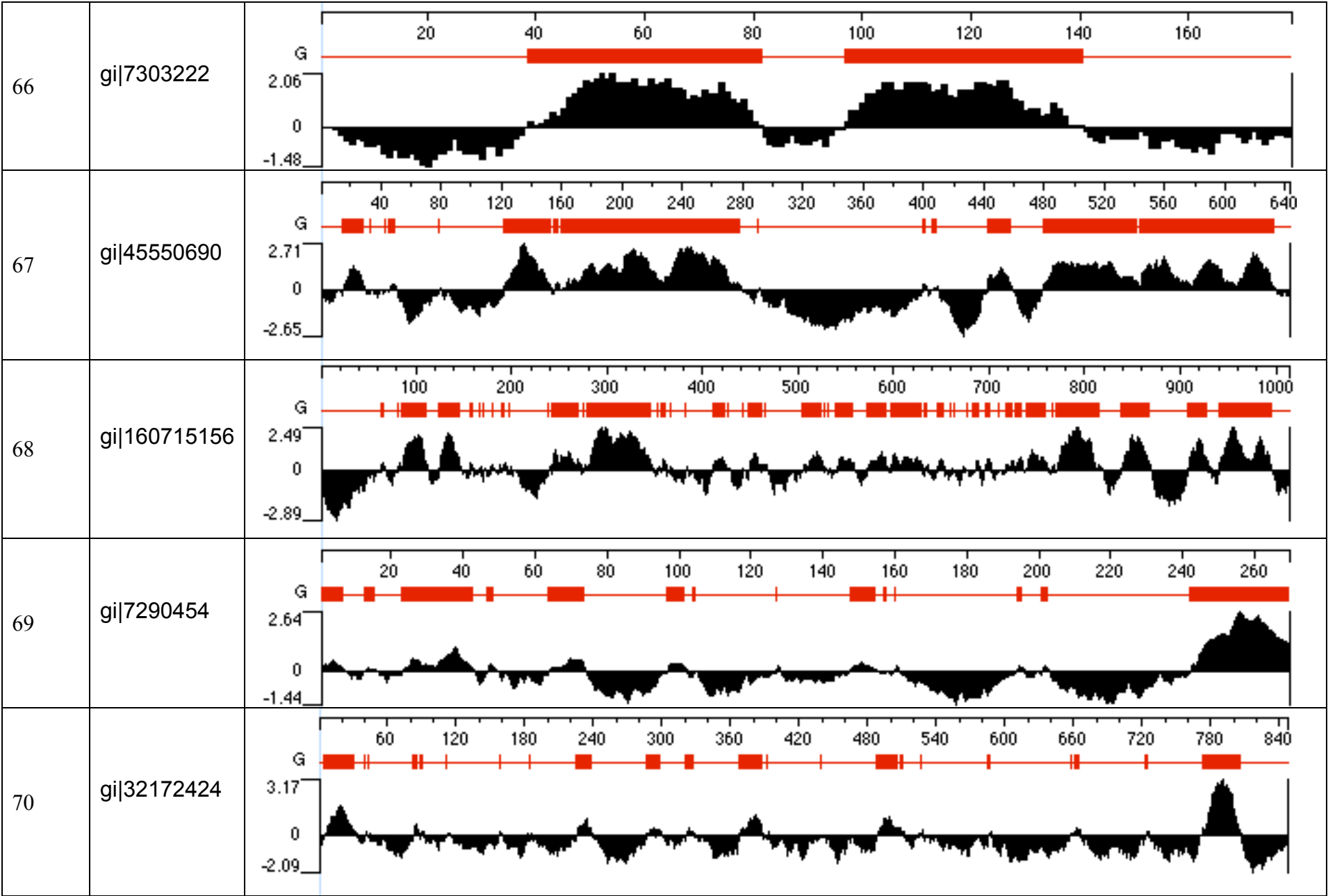

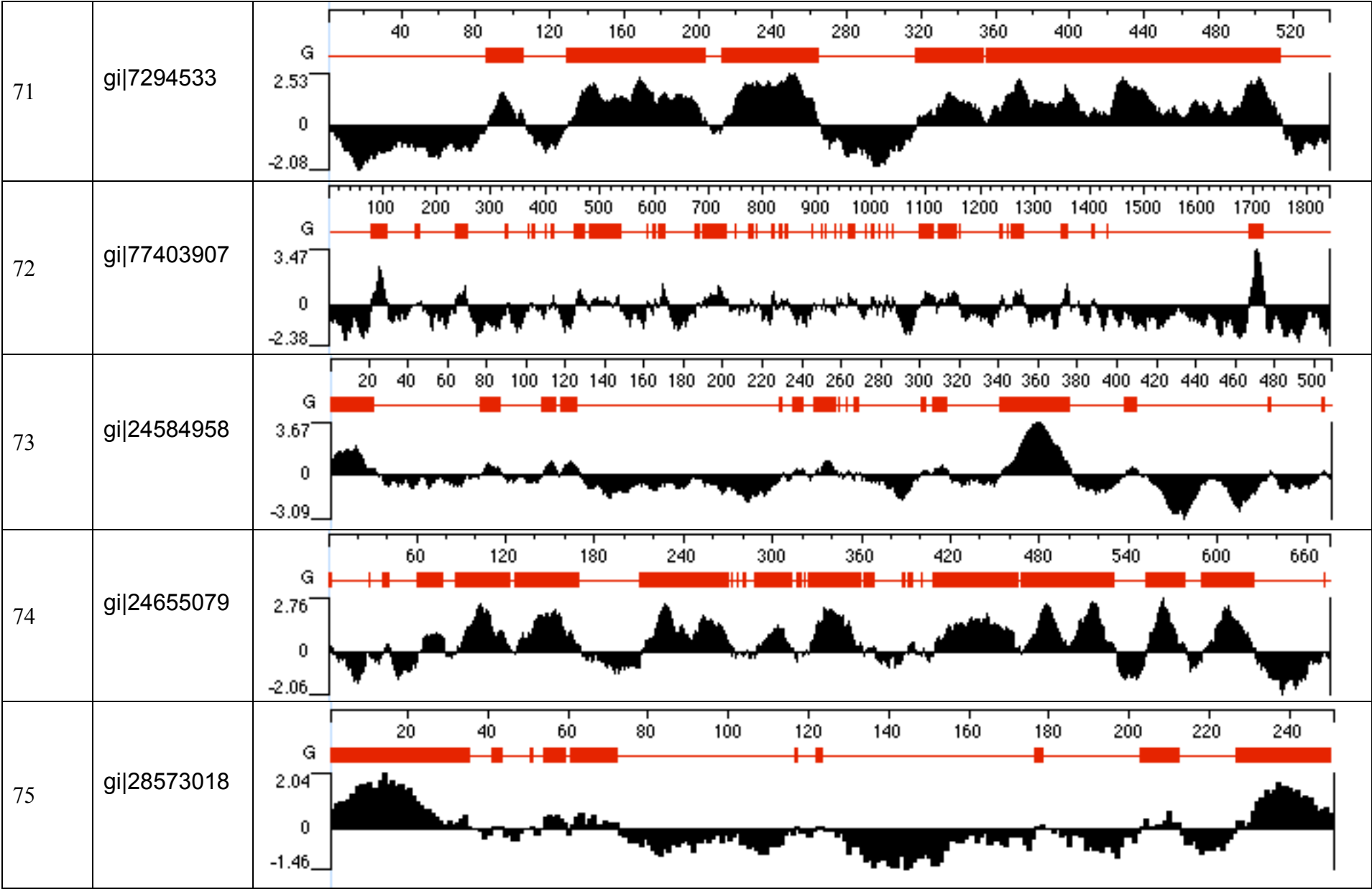

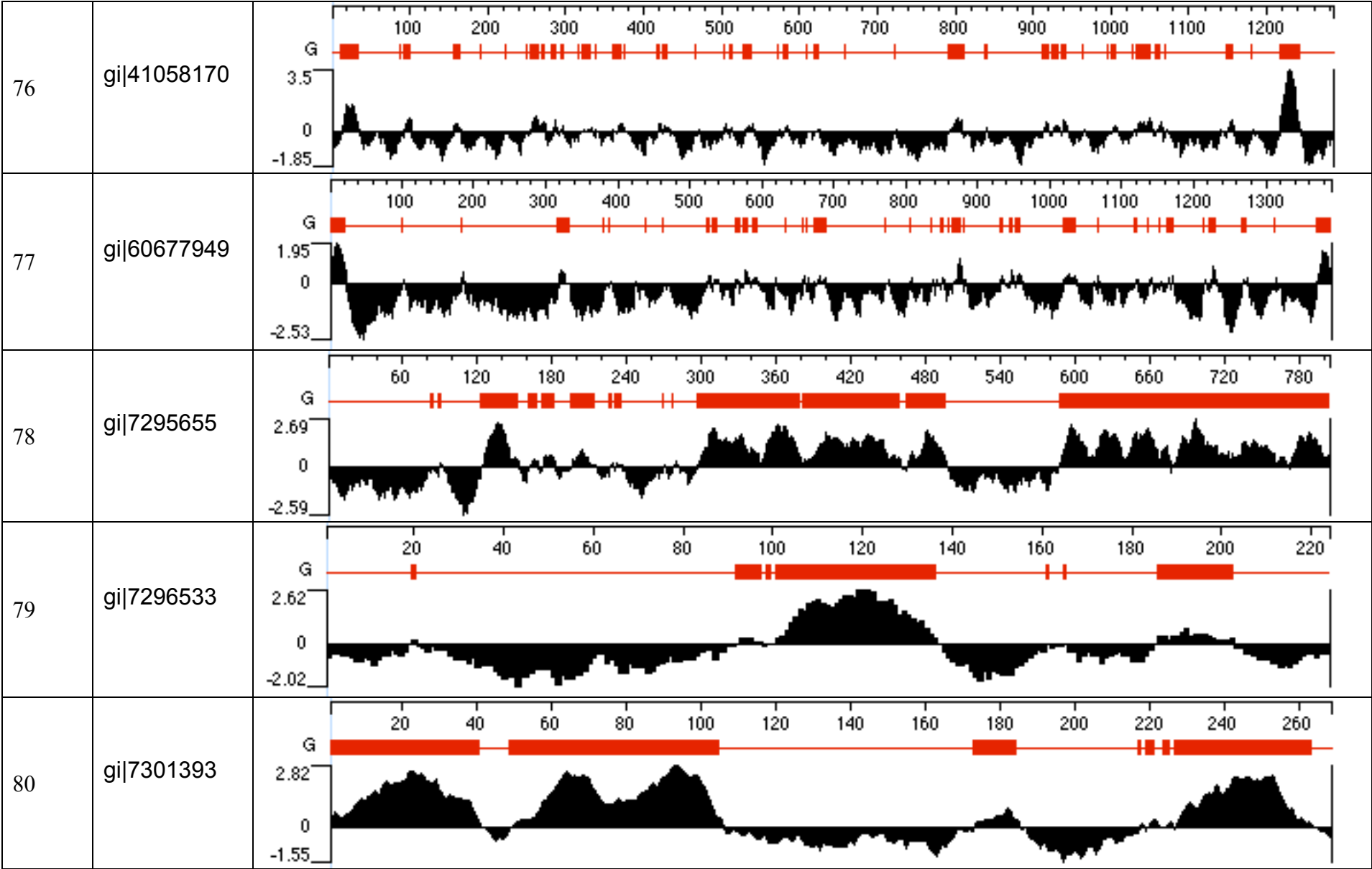

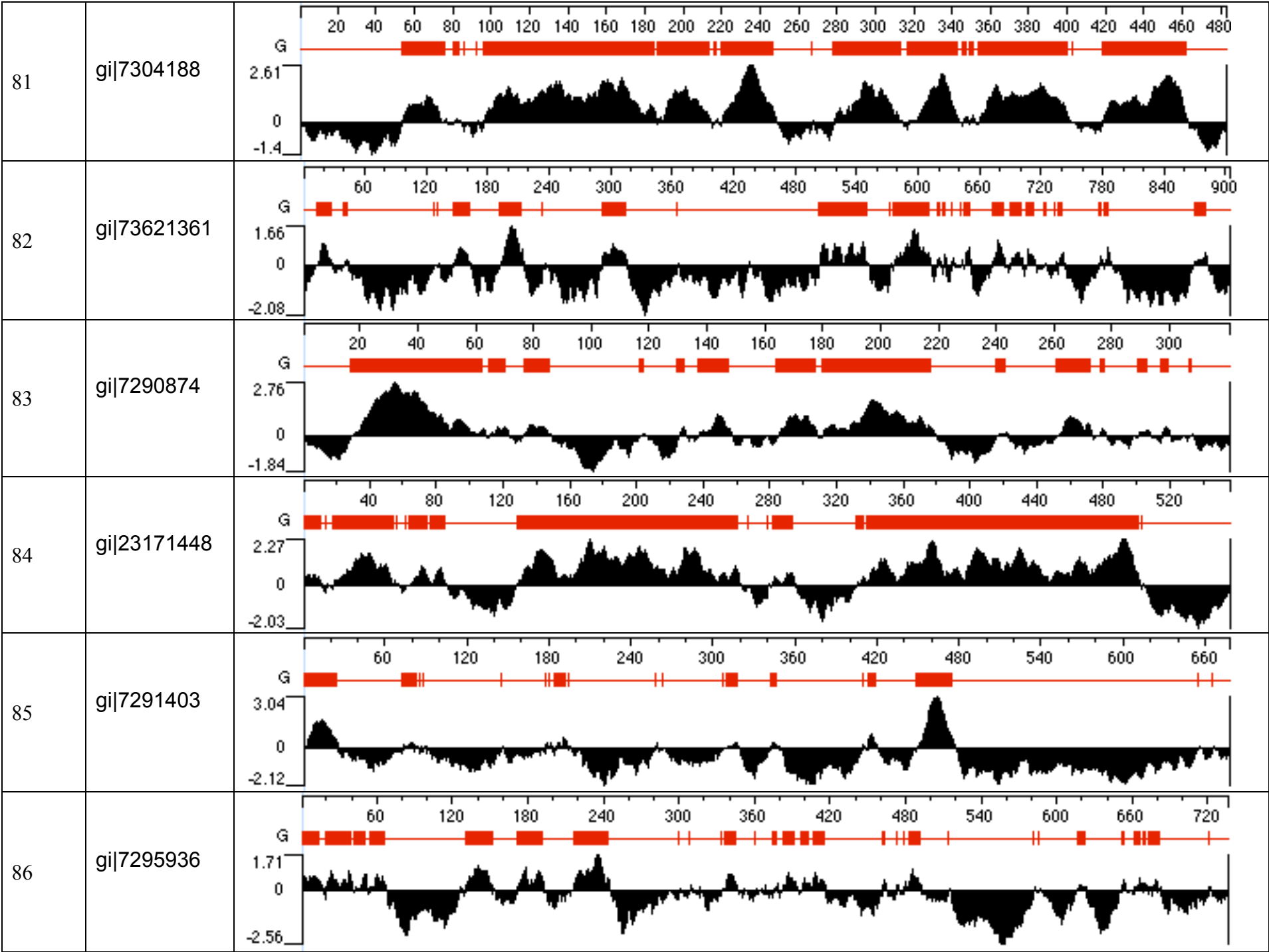

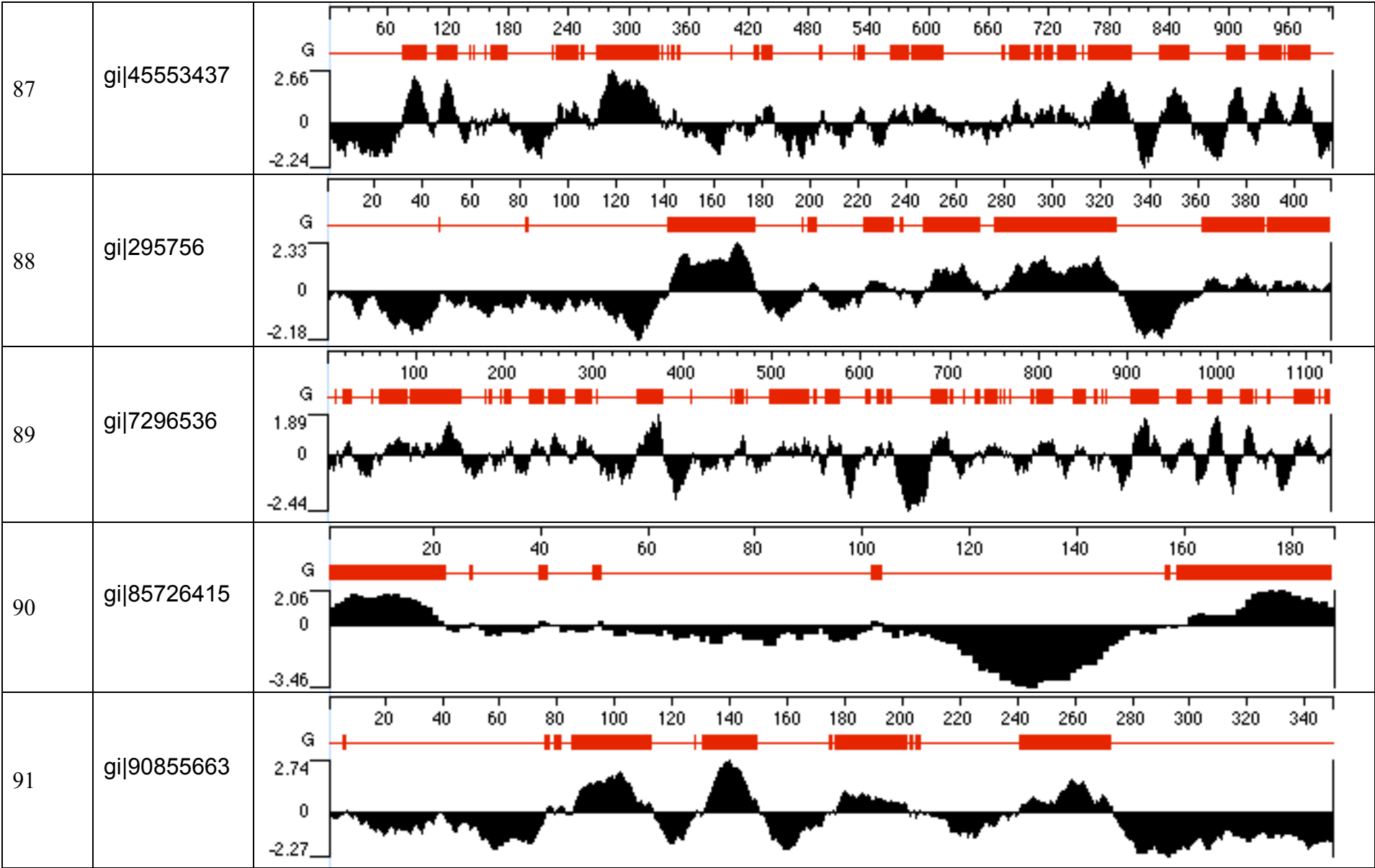

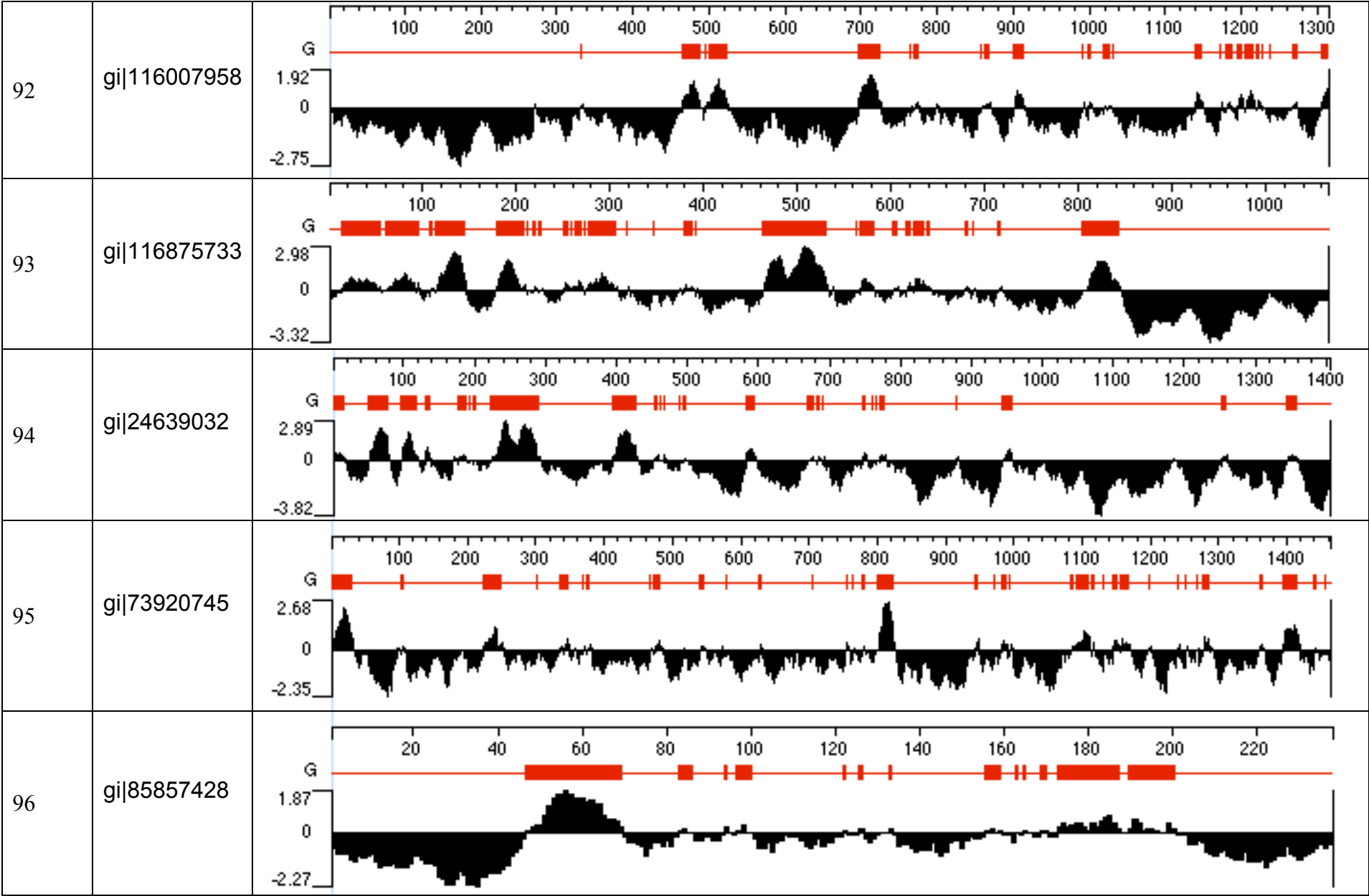

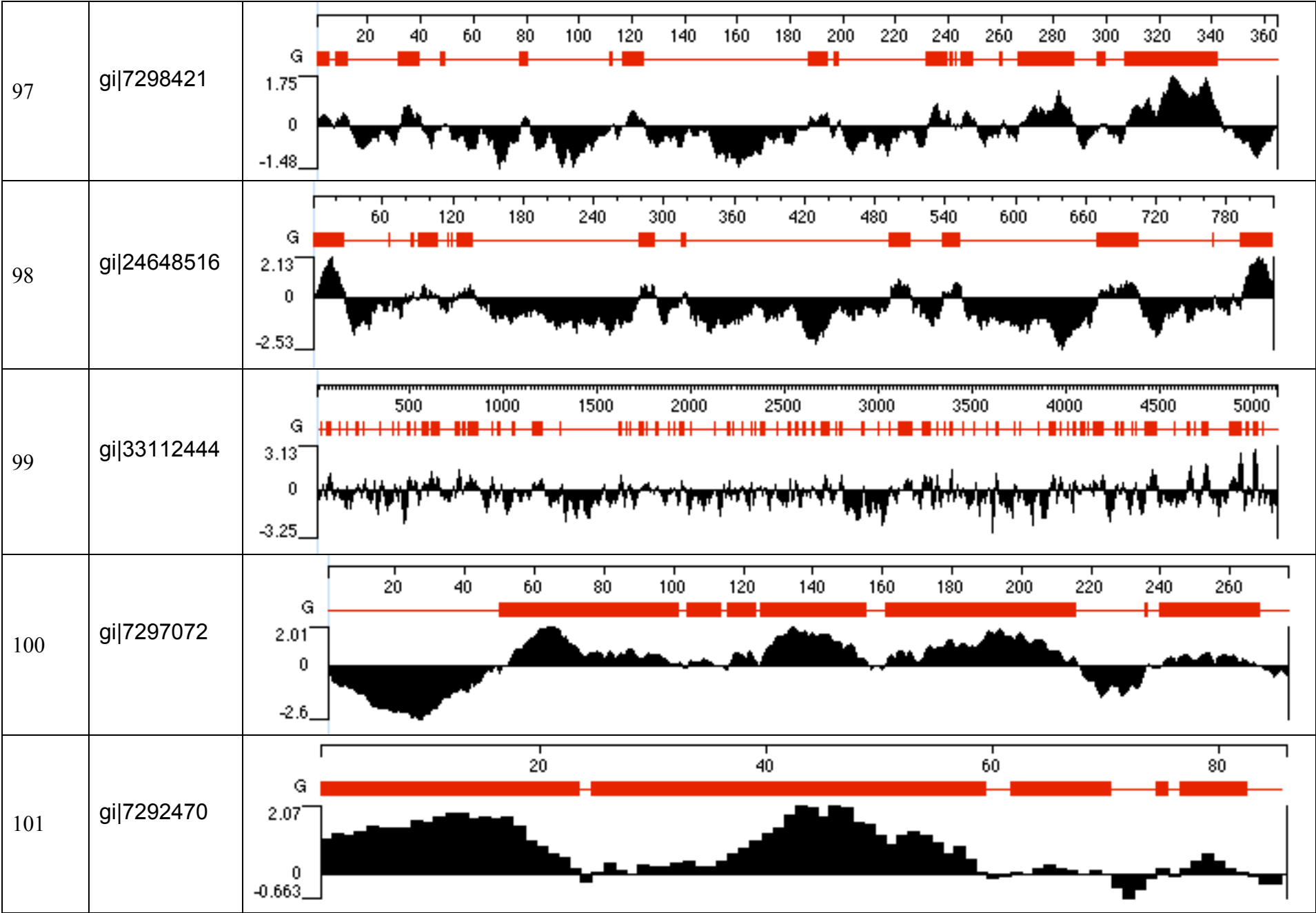

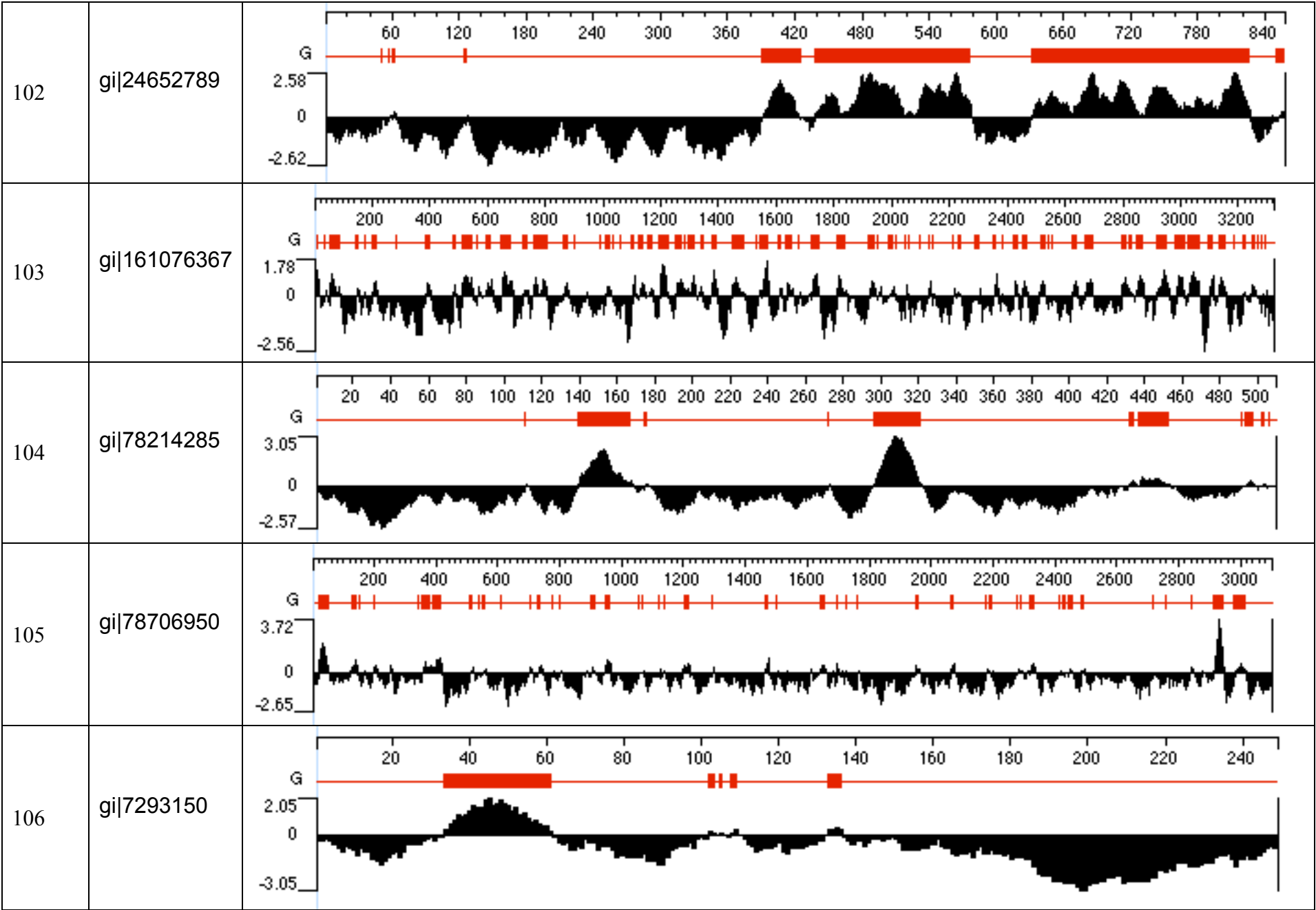

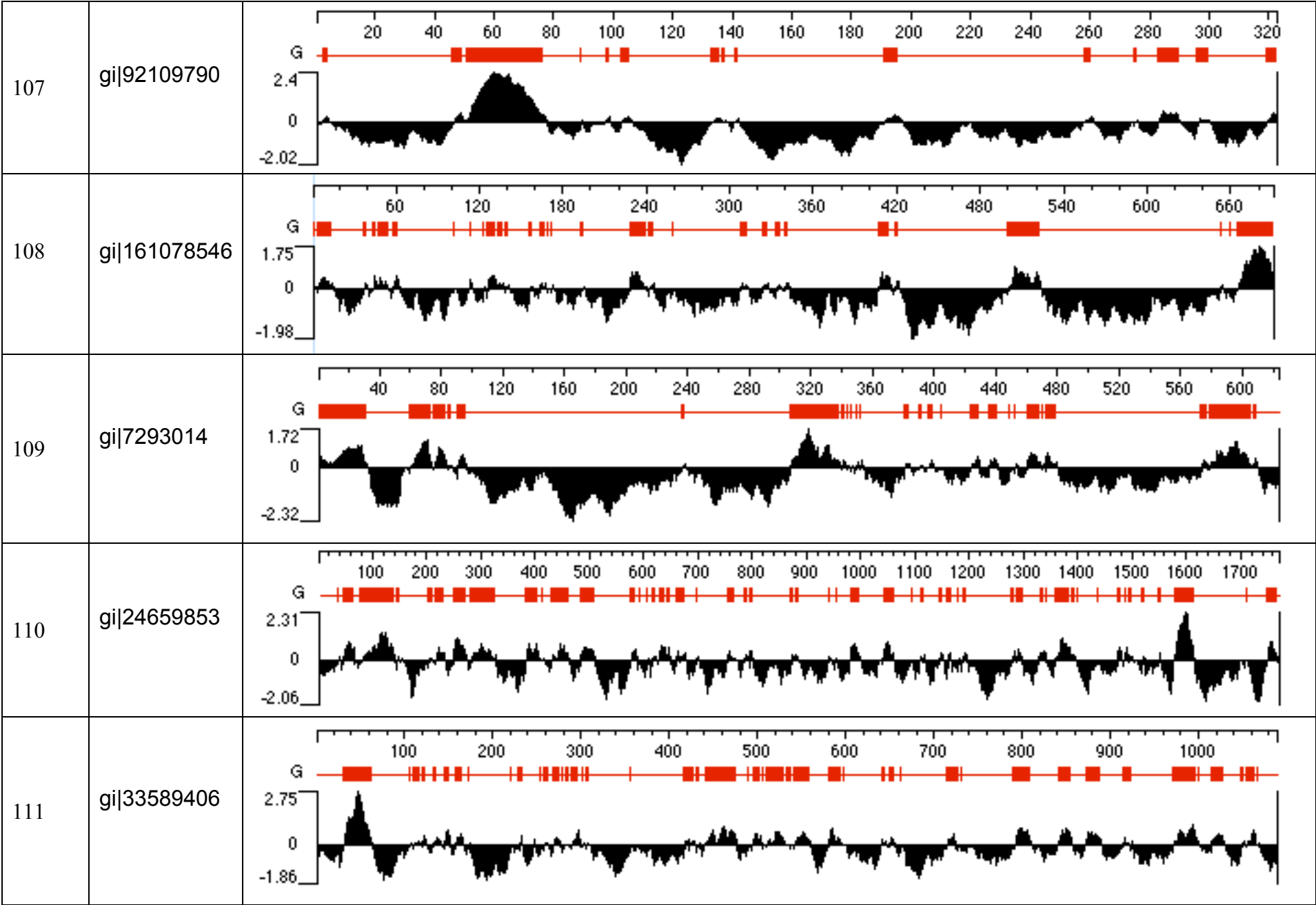

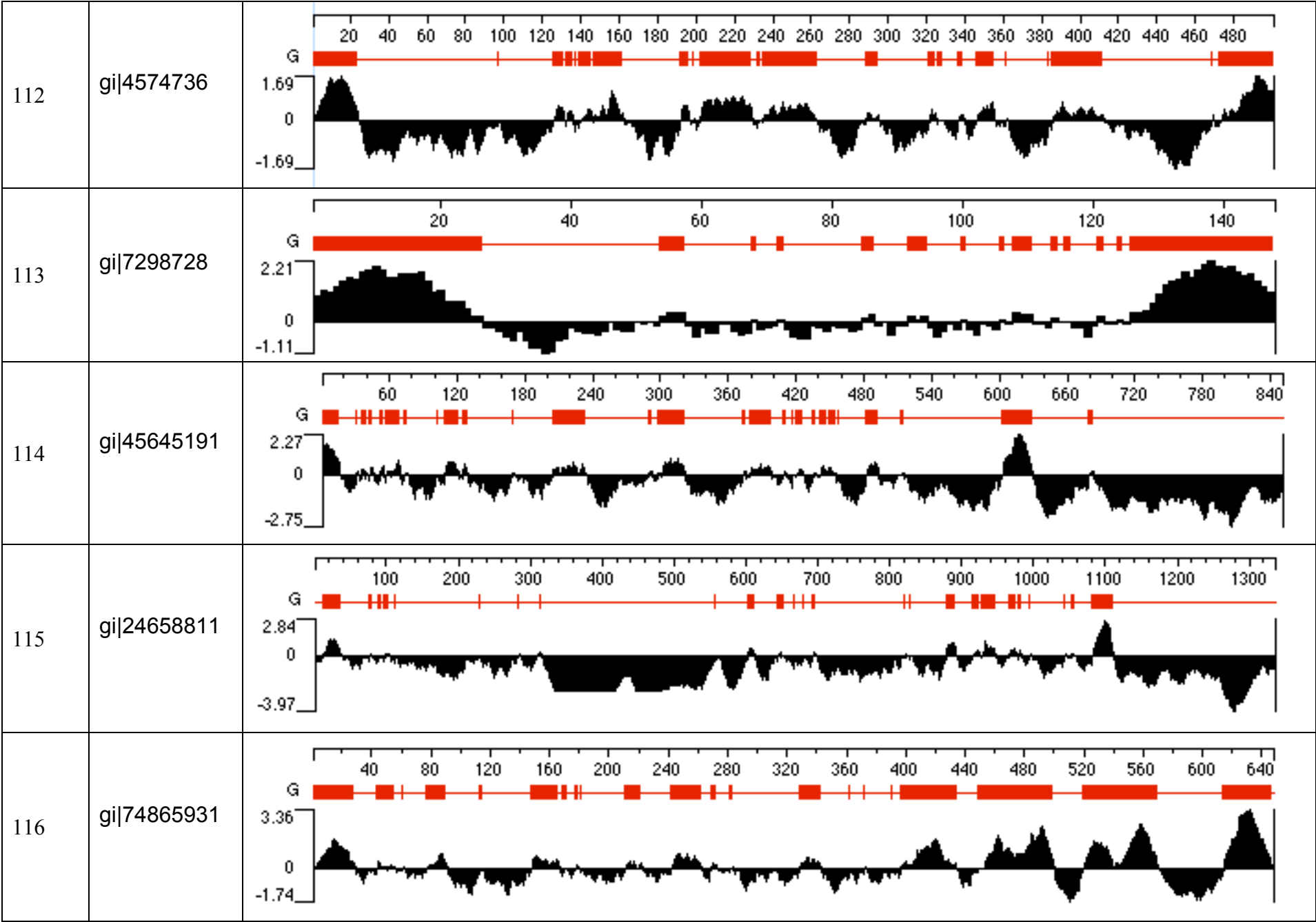

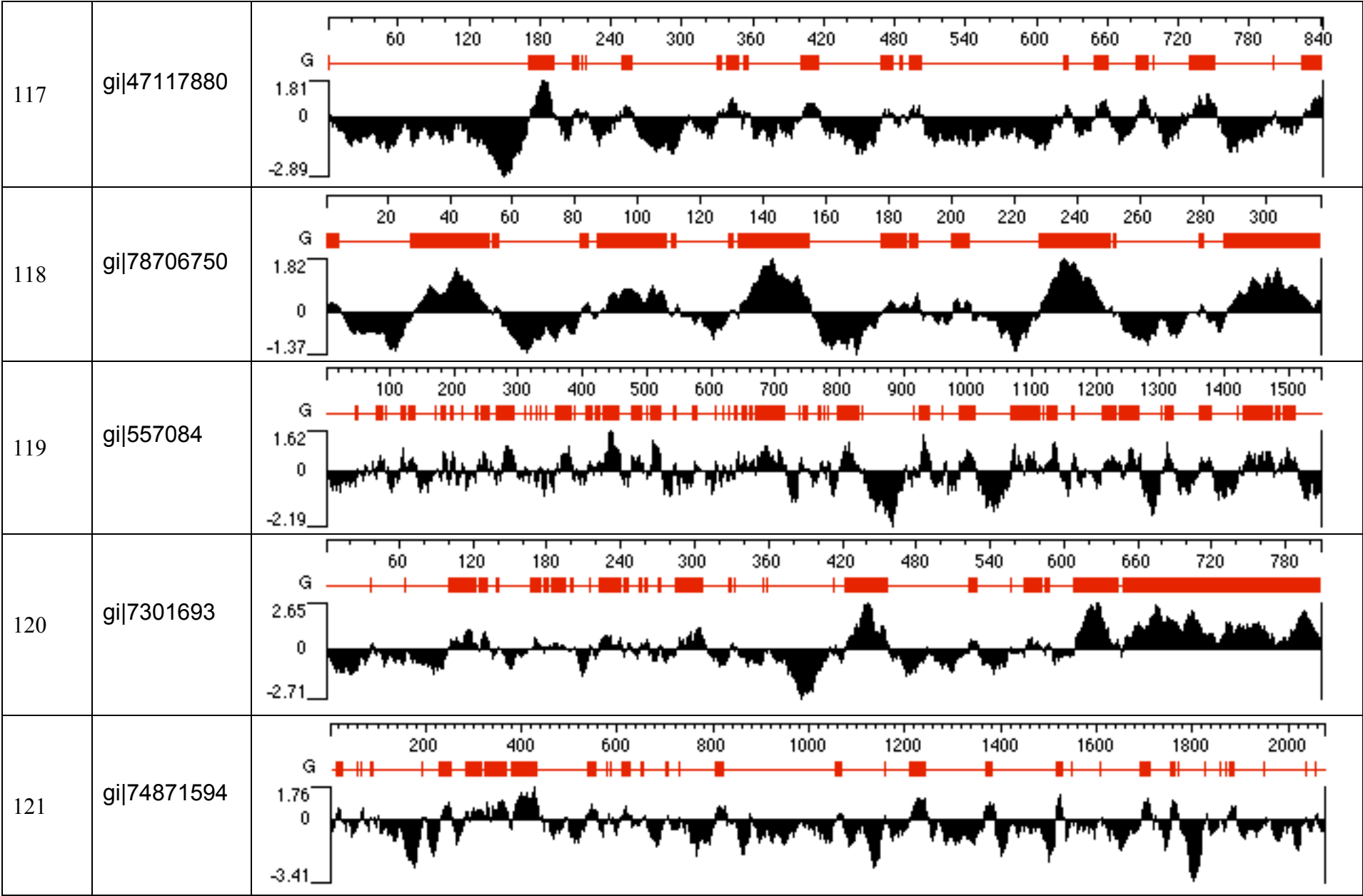

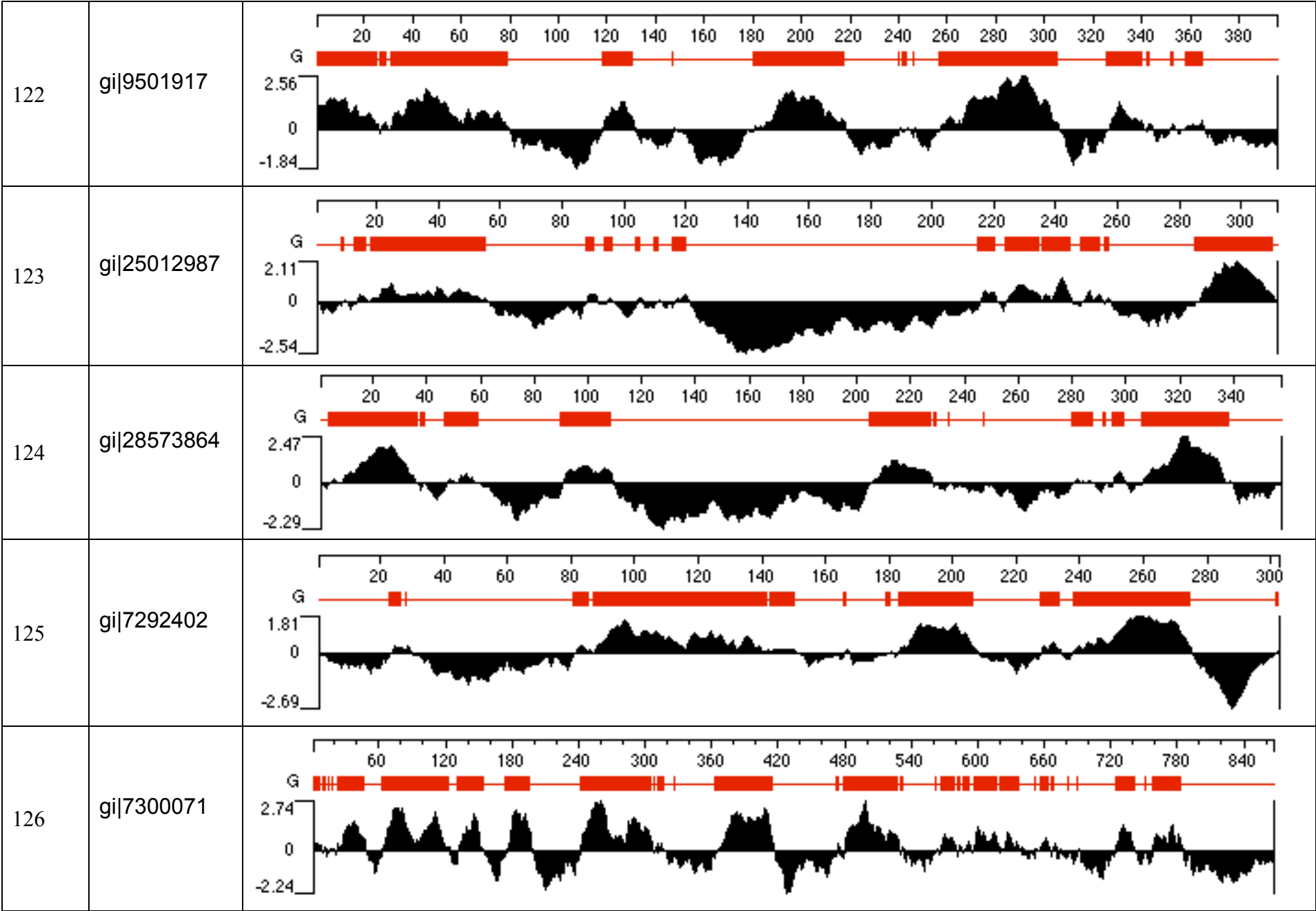

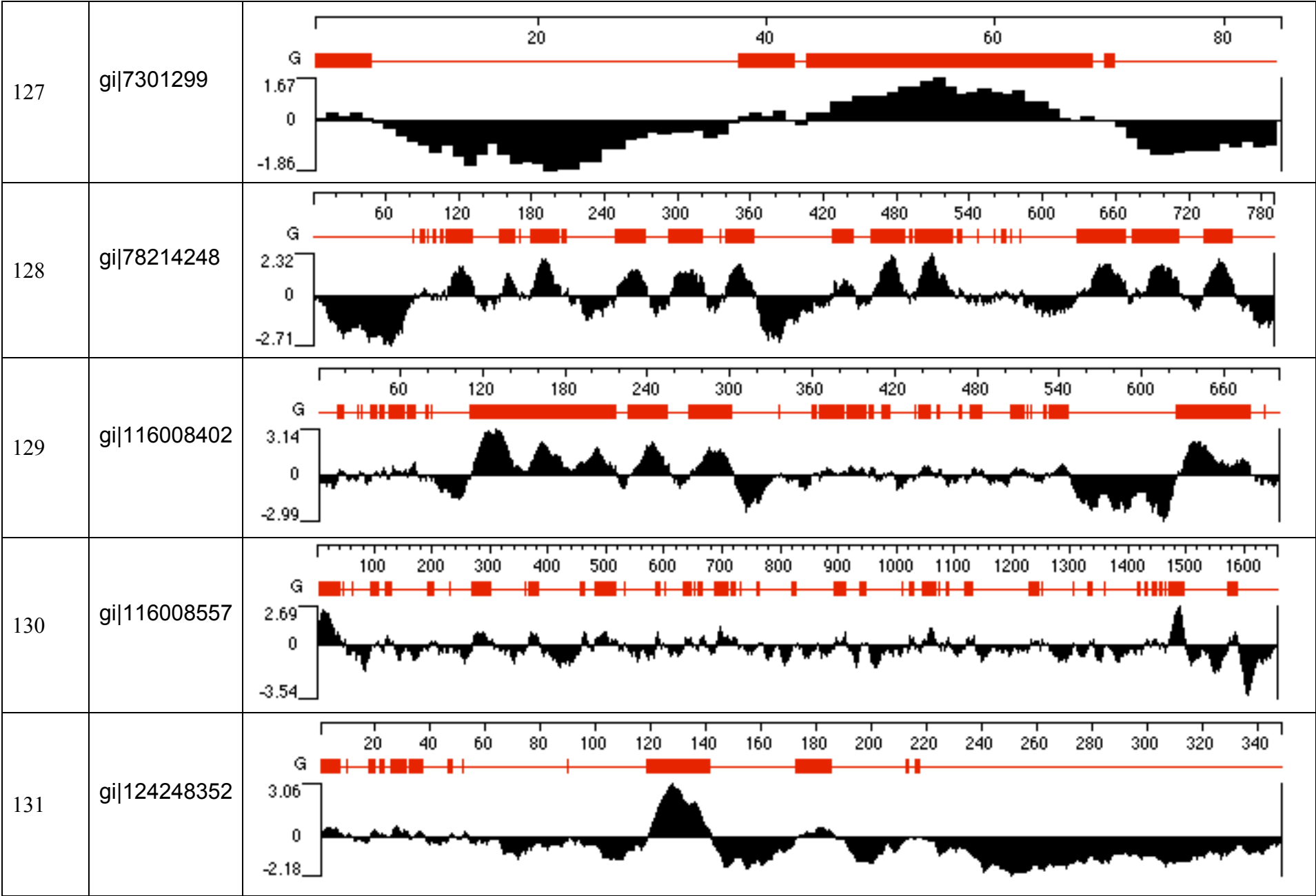

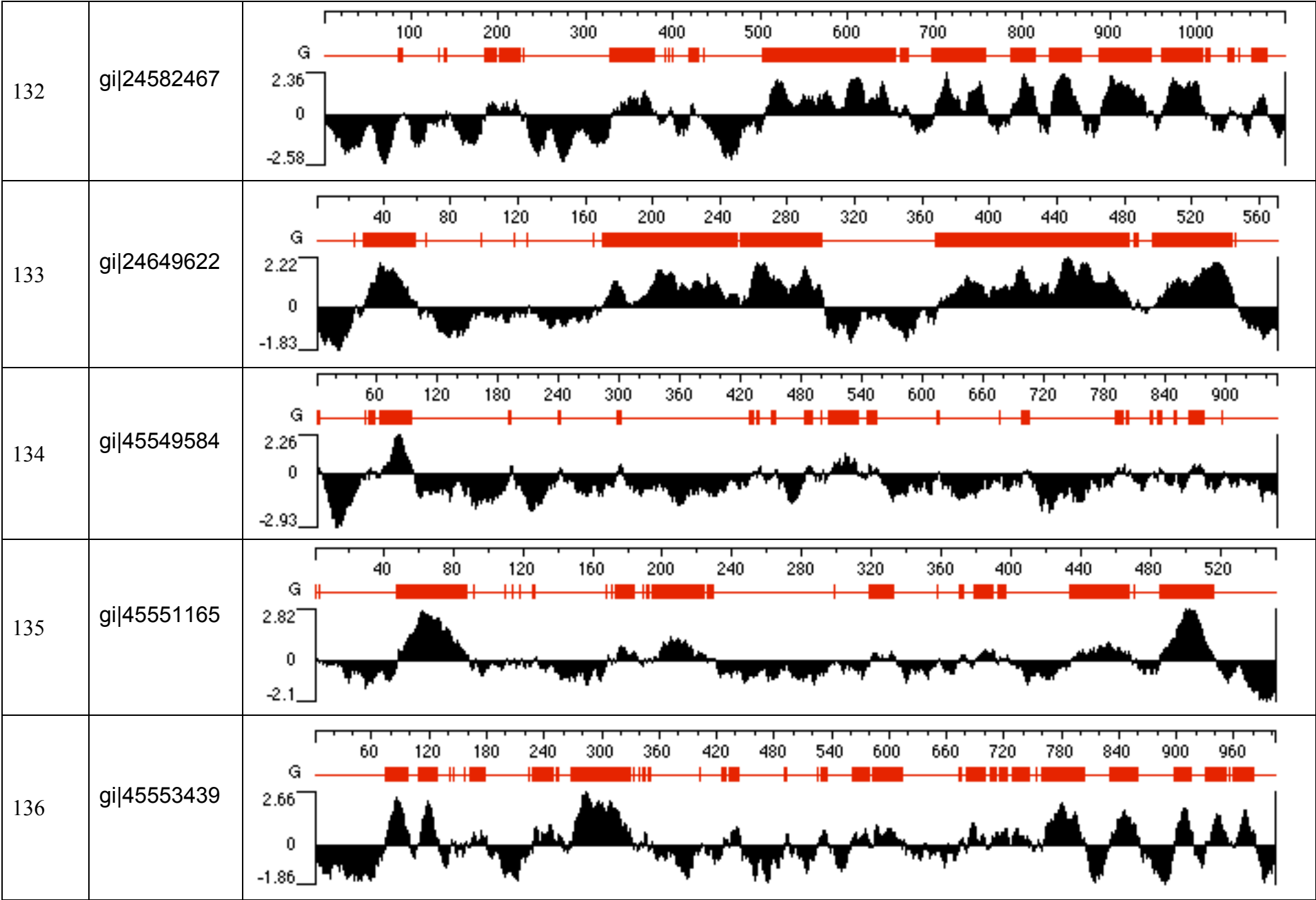

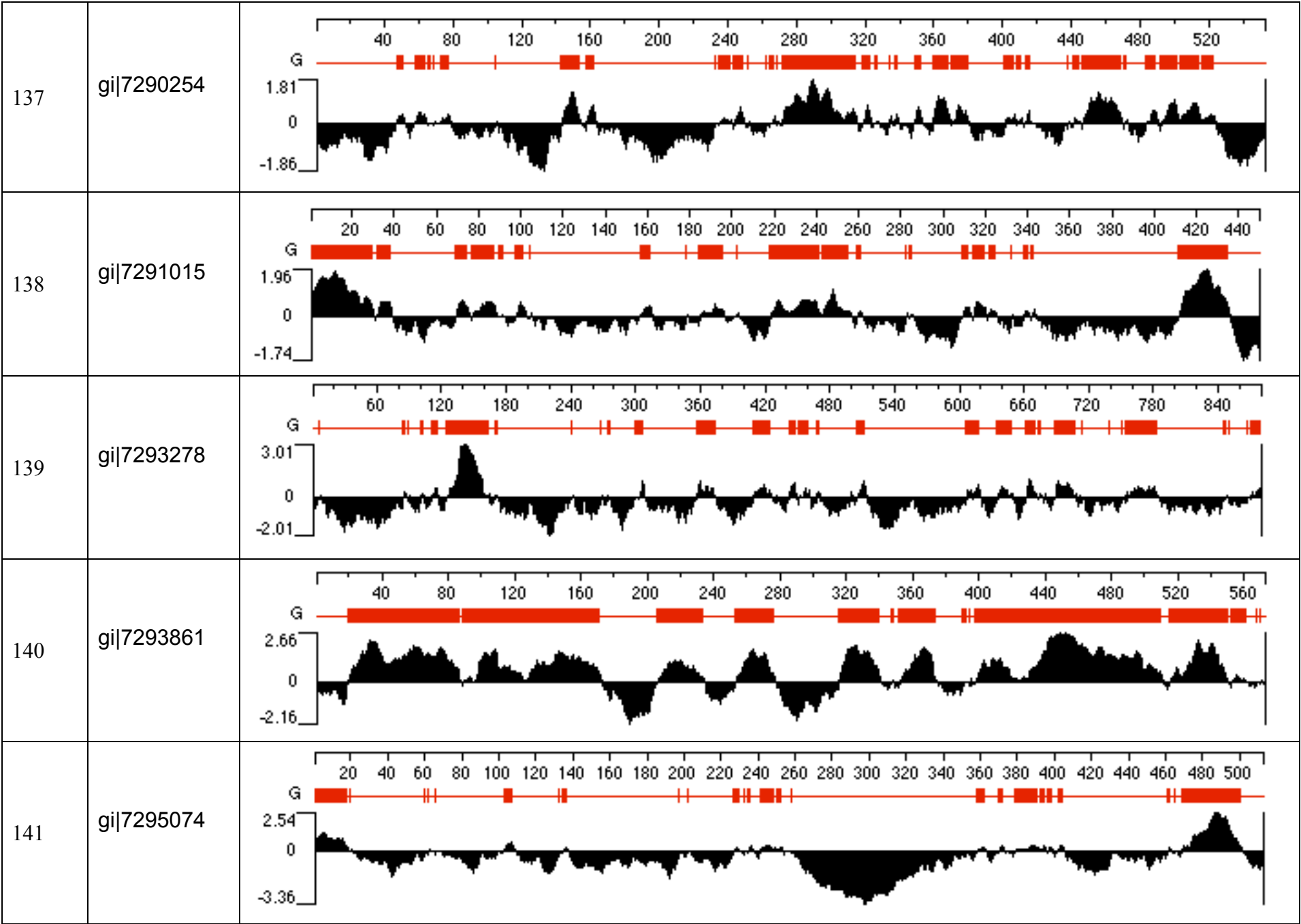

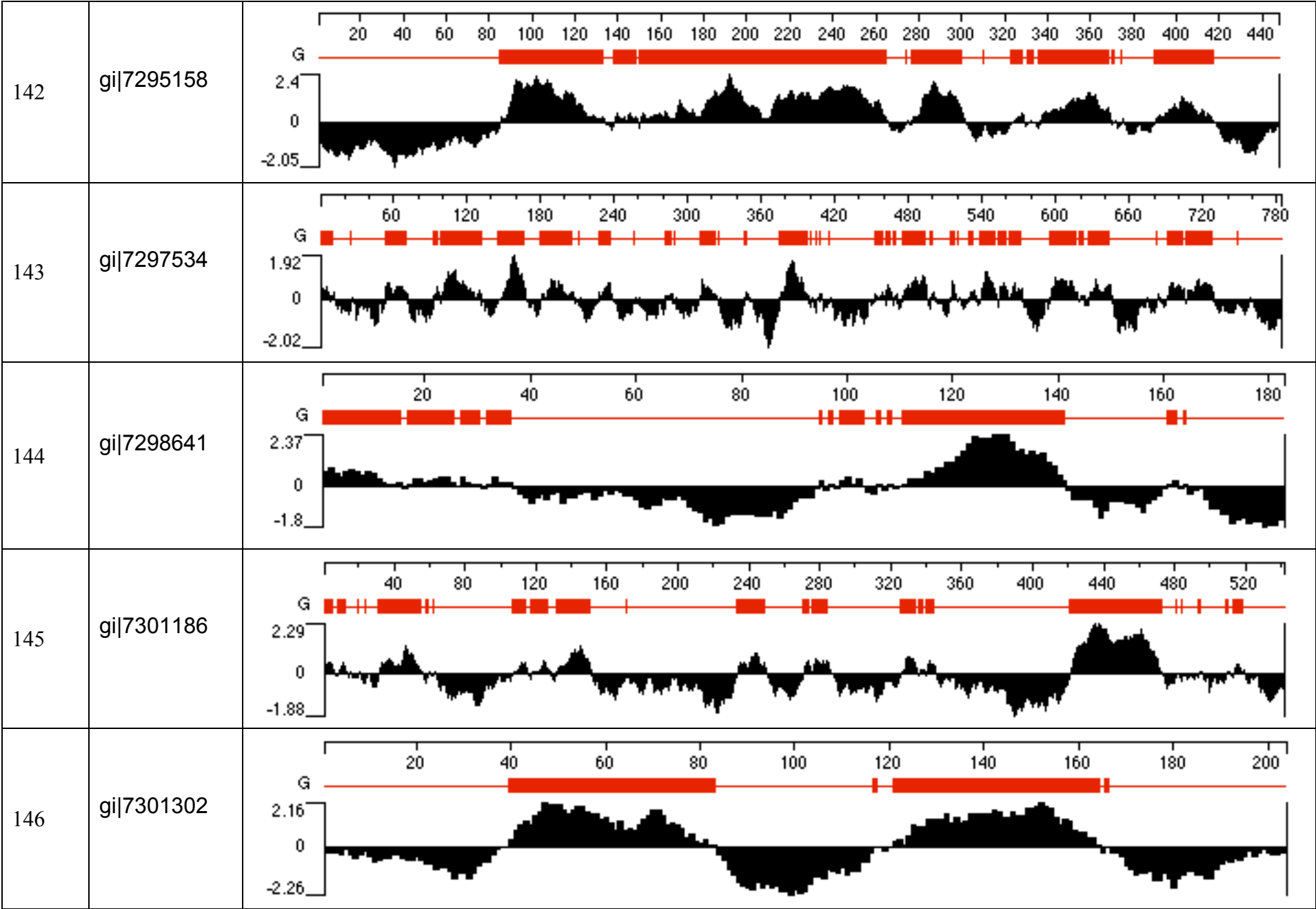

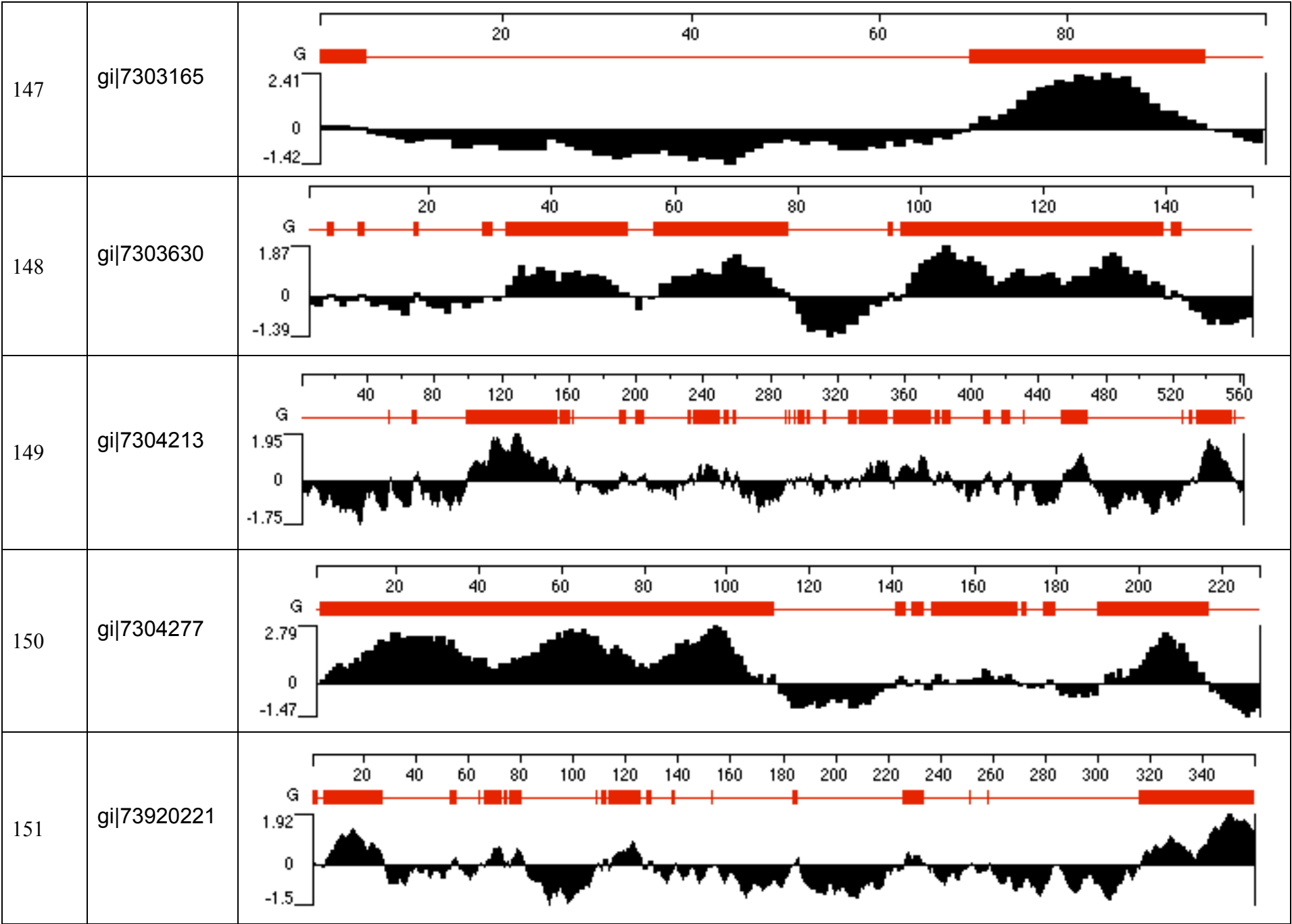

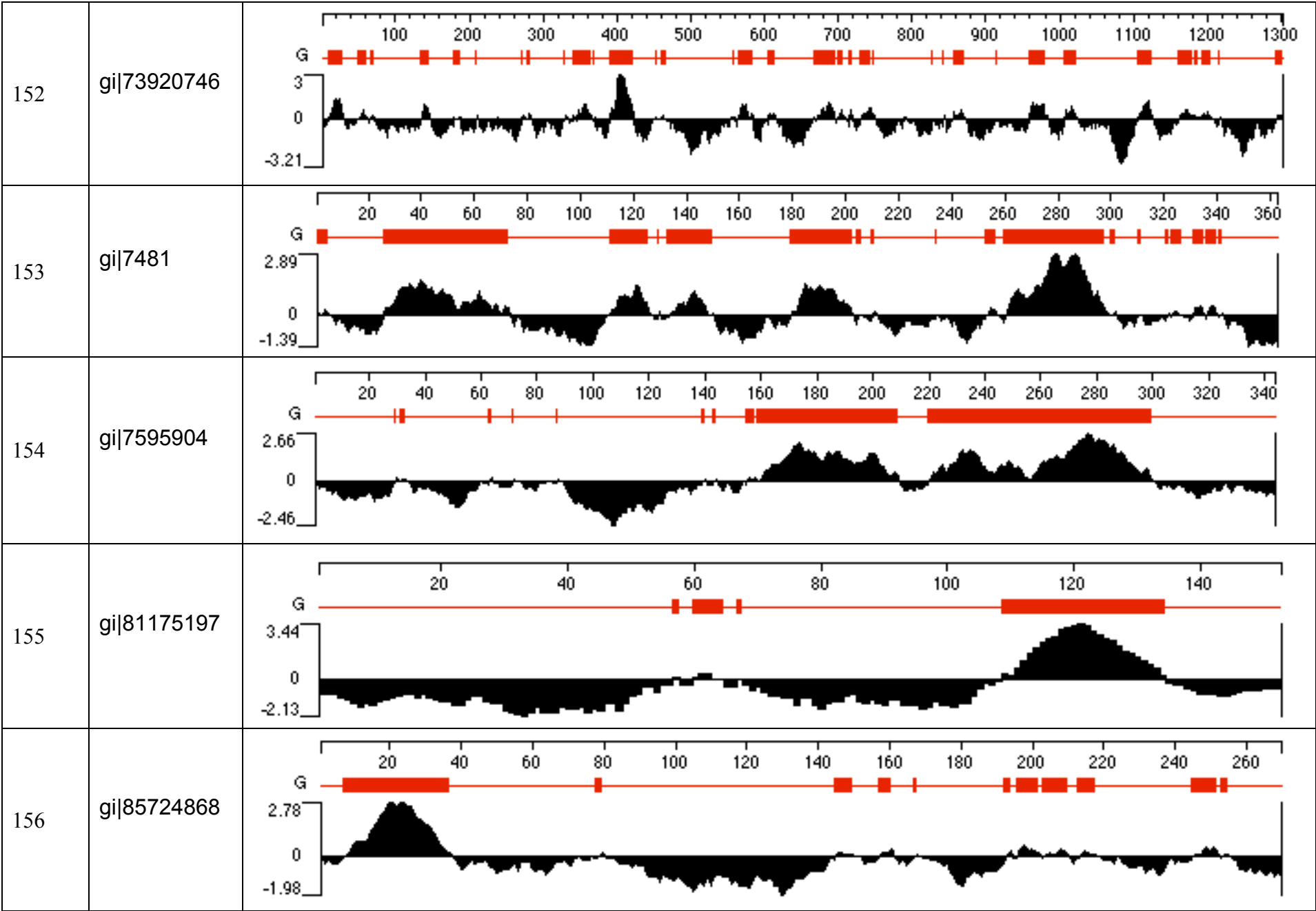

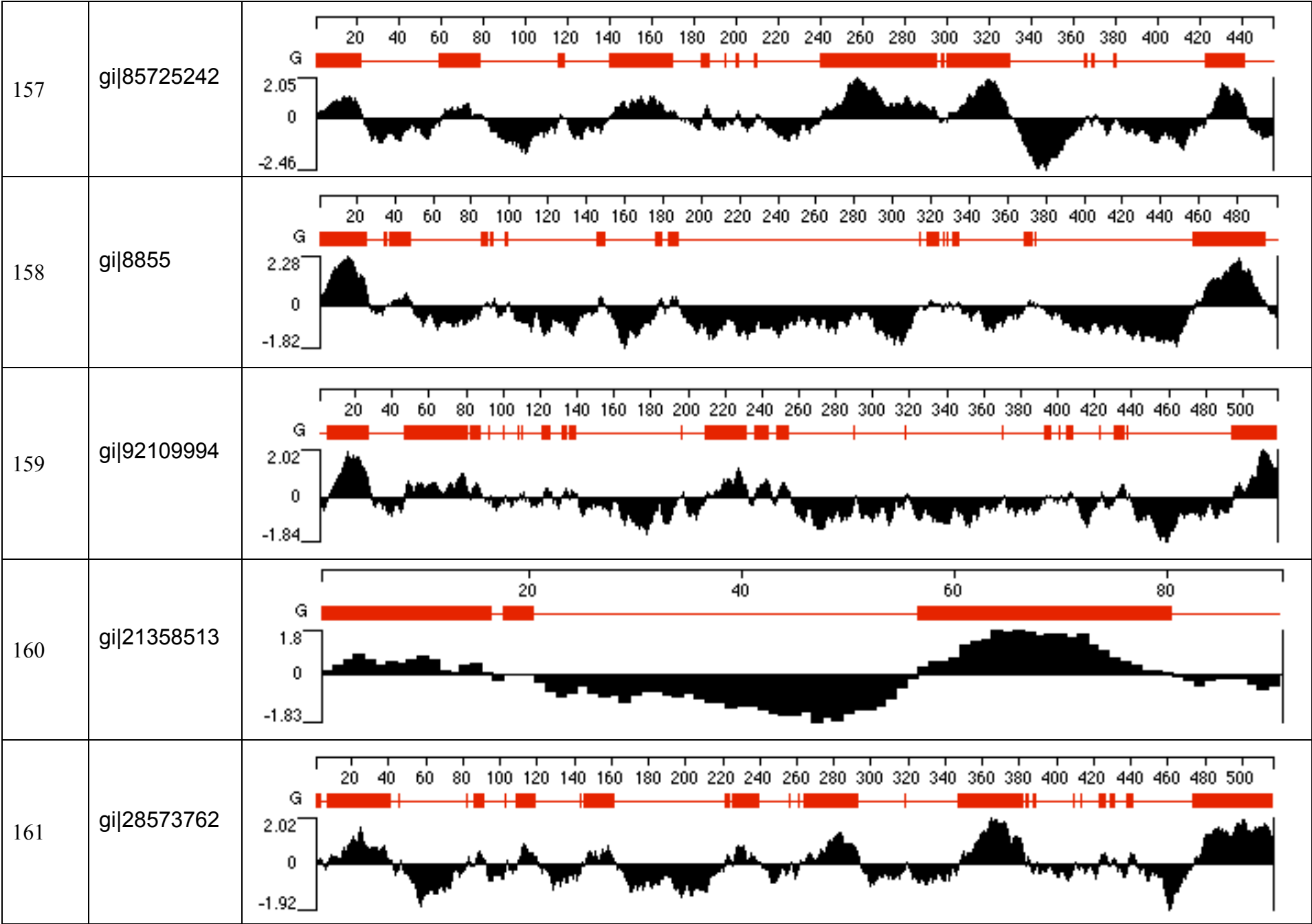

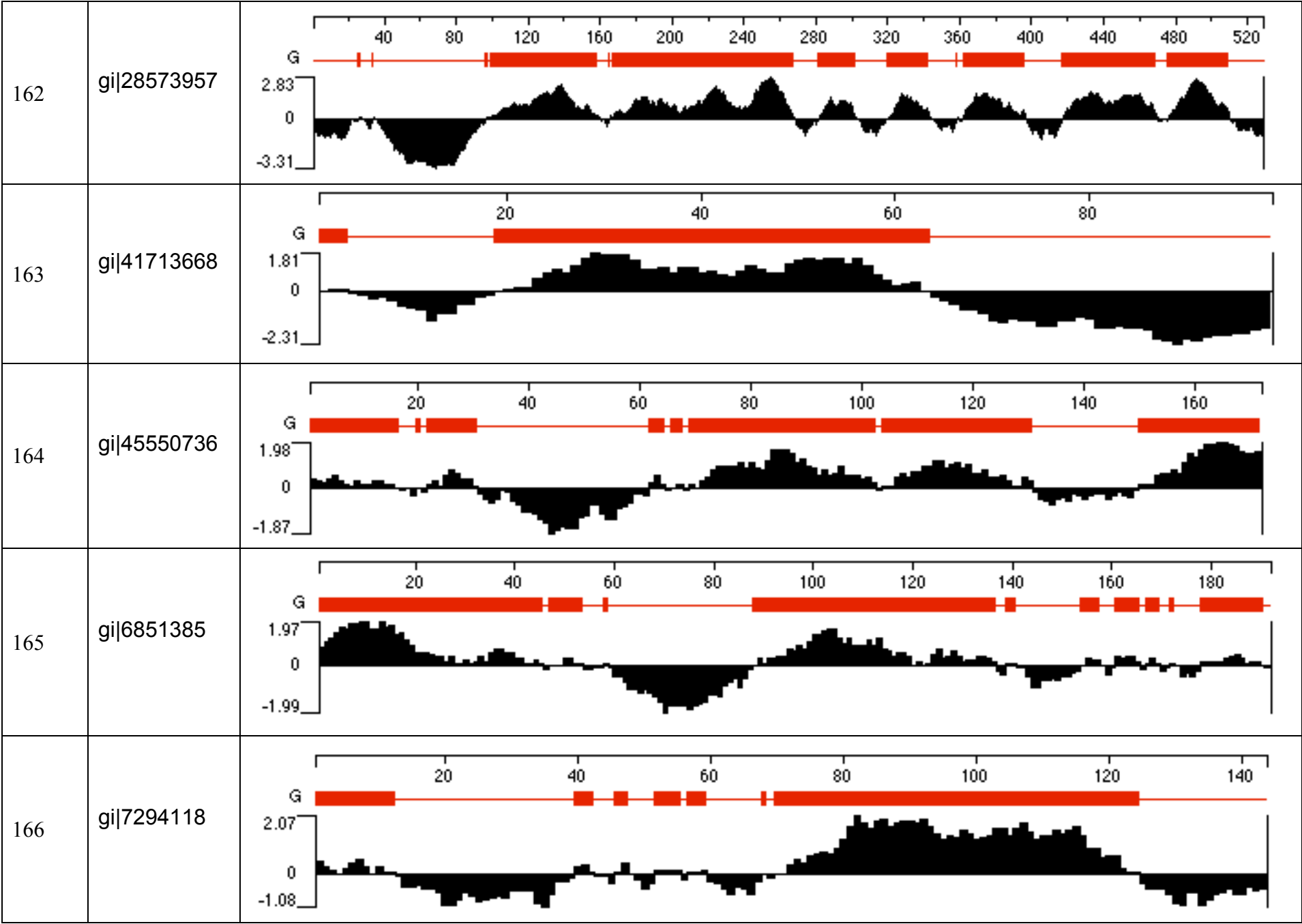

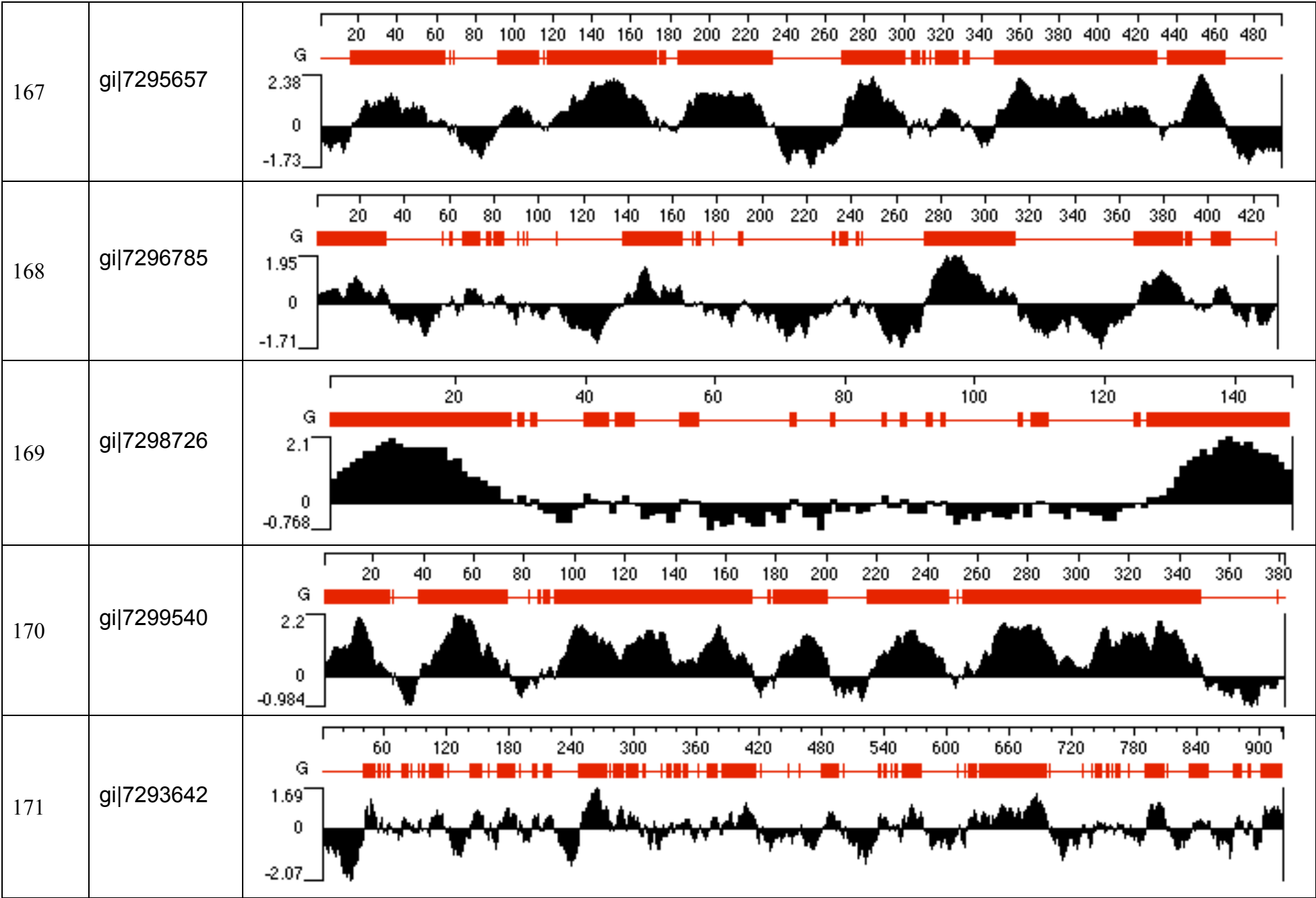

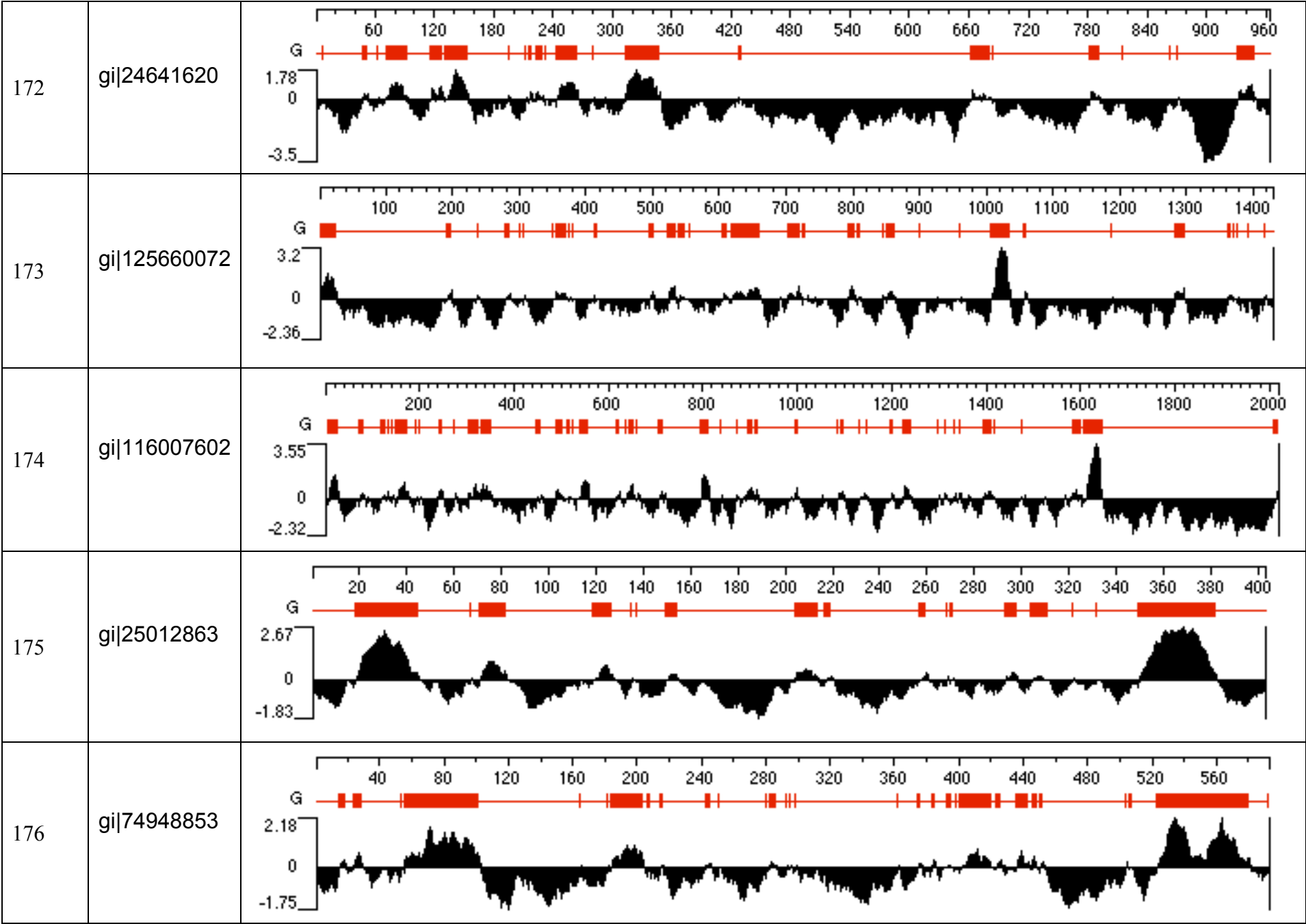

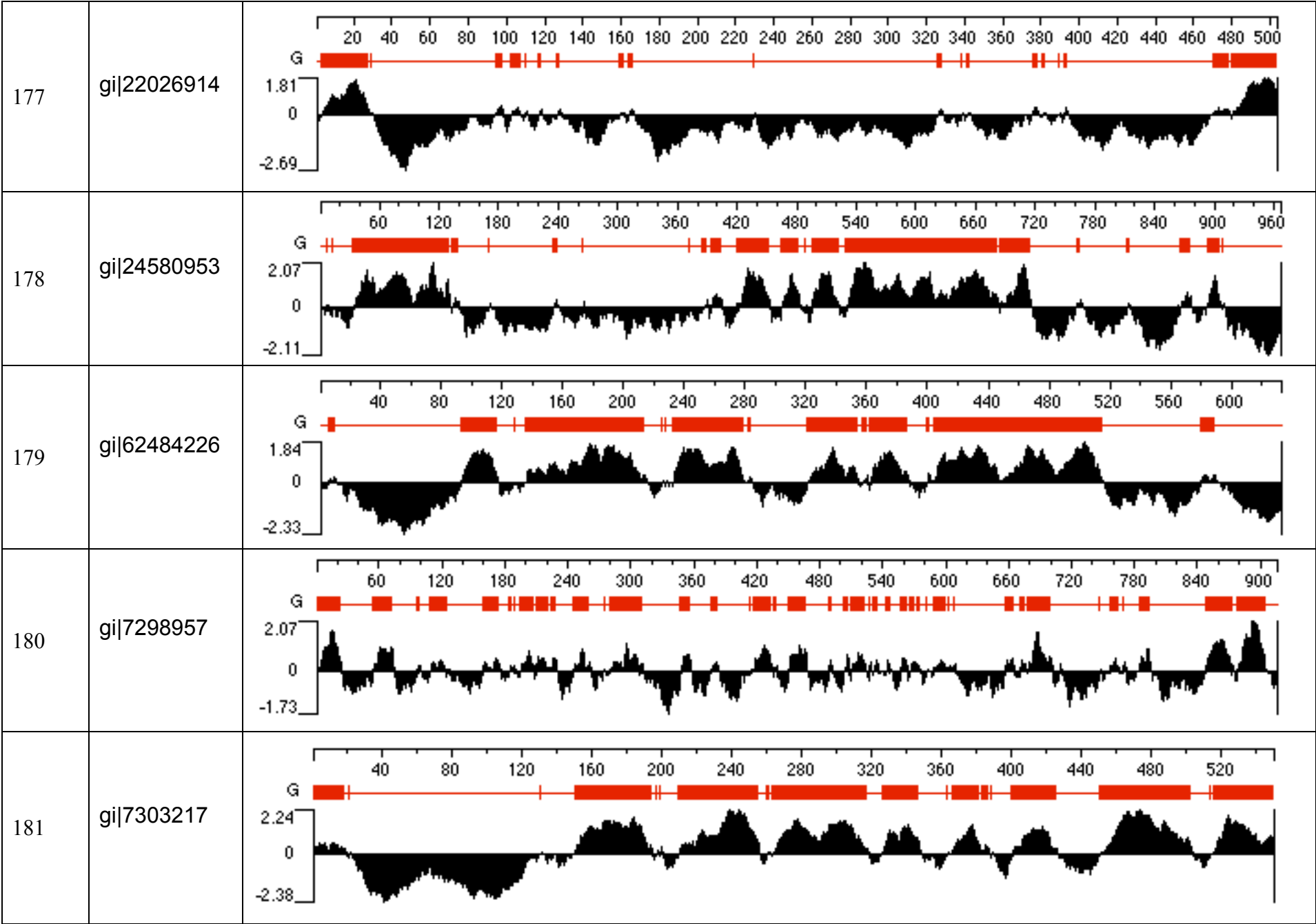

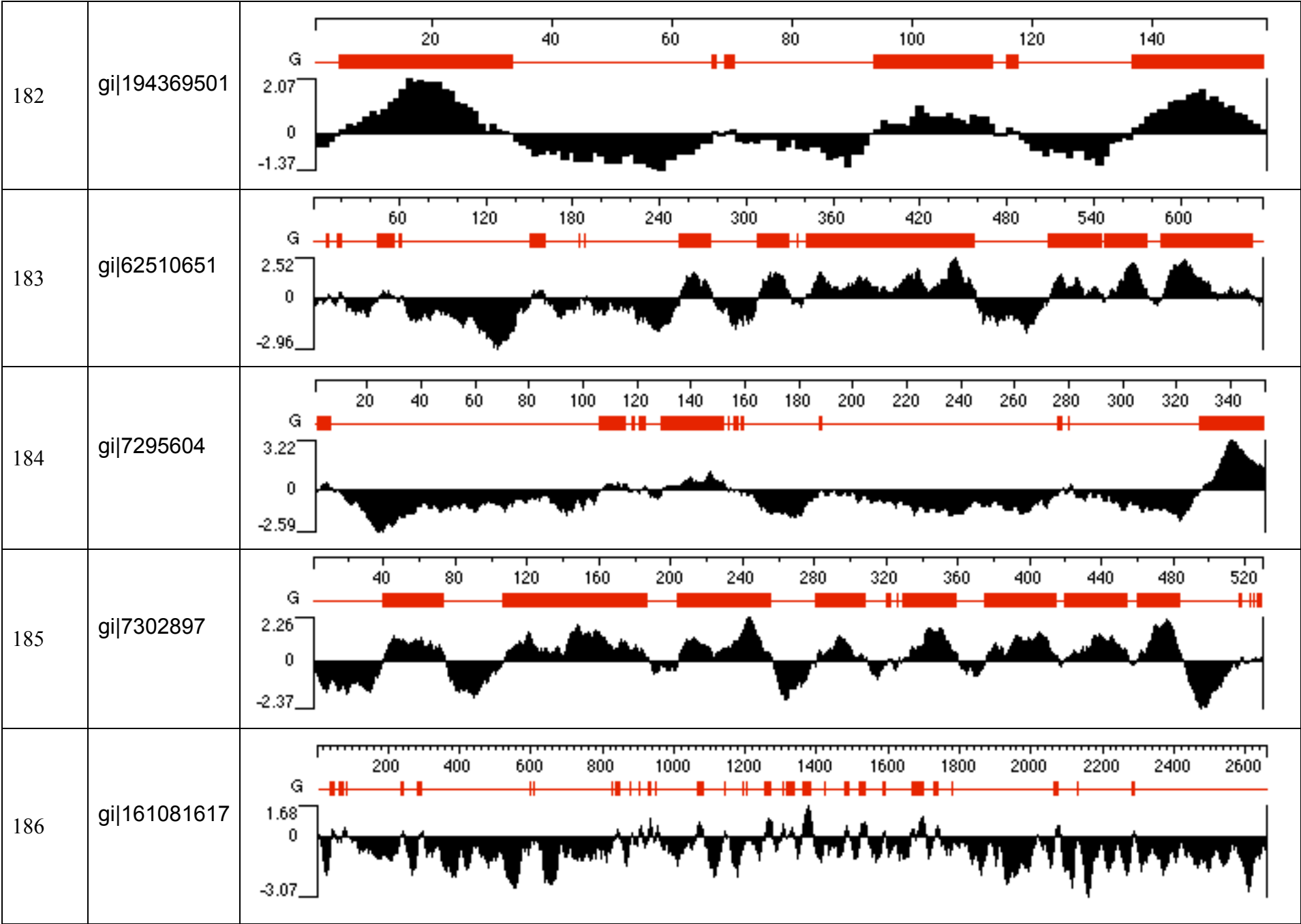

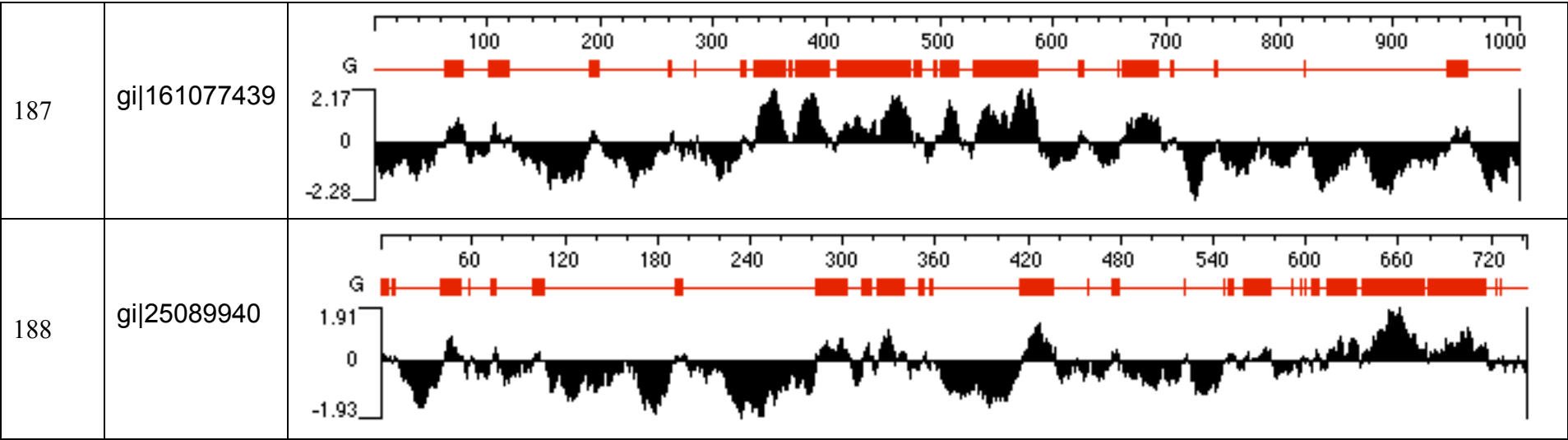

Supplement: Additional file 4 — Hydropathy plots for proteins predicted to have transmembrane domains: This table provides the hydropathy plots of all those proteins predicted to have transmembrane domains by the method of Kyte and Doolittle. [file 1471-2164-11-302-S4.PDF]
